# Identifying Patients with Atrioventricular Septal Defect in Down Syndrome Populations by Using Self-Normalizing Neural Networks and Feature Selection

**DOI:** 10.3390/genes9040208

**Published:** 2018-04-12

**Authors:** Xiaoyong Pan, Xiaohua Hu, Yu Hang Zhang, Kaiyan Feng, Shao Peng Wang, Lei Chen, Tao Huang, Yu Dong Cai

**Affiliations:** 1 College of Life Science, Shanghai University, Shanghai 200444, China; x.pan@erasmusmc.nl (X.P.); wsptfb@163.com (S.P.W.); 2 Department of Medical Informatics, Erasmus MC, 3015 CE Rotterdam, The Netherlands; 3 Department of Biostatistics and Computational Biology, School of Life Sciences, Fudan University, Shanghai 200438, China; xhhu@fudan.edu.cn; 4 Institute of Health Sciences, Shanghai Institutes for Biological Sciences, Chinese Academy of Sciences, Shanghai 200031, China; zhangyh825@163.com; 5 Department of Computer Science, Guangdong AIB Polytechnic, Guangzhou 510507, China; addland@126.com; 6 College of Information Engineering, Shanghai Maritime University, Shanghai 201306, China

**Keywords:** atrioventricular septal defect, Down syndrome, self-normalizing neural network, Monte Carlo feature selection, random forest

## Abstract

Atrioventricular septal defect (AVSD) is a clinically significant subtype of congenital heart disease (CHD) that severely influences the health of babies during birth and is associated with Down syndrome (DS). Thus, exploring the differences in functional genes in DS samples with and without AVSD is a critical way to investigate the complex association between AVSD and DS. In this study, we present a computational method to distinguish DS patients with AVSD from those without AVSD using the newly proposed self-normalizing neural network (SNN). First, each patient was encoded by using the copy number of probes on chromosome 21. The encoded features were ranked by the reliable Monte Carlo feature selection (MCFS) method to obtain a ranked feature list. Based on this feature list, we used a two-stage incremental feature selection to construct two series of feature subsets and applied SNNs to build classifiers to identify optimal features. Results show that 2737 optimal features were obtained, and the corresponding optimal SNN classifier constructed on optimal features yielded a Matthew’s correlation coefficient (MCC) value of 0.748. For comparison, random forest was also used to build classifiers and uncover optimal features. This method received an optimal MCC value of 0.582 when top 132 features were utilized. Finally, we analyzed some key features derived from the optimal features in SNNs found in literature support to further reveal their essential roles.

## 1. Introduction

Congenital heart disease (CHD) is a defect of the heart structure at birth [1] and is one of the most common birth defects in America with 8 out 1000 newborns affected with different severities [2]. In 2010, more than 35,000 babies in the United States are born with CHD [2,3]. With the advances in medical technologies, majority of these babies can have active, productive lives with proper medical interference. To date, more than 1 million adults live with CHD in the United States [3], implying that this disease is a widely distributed and significant threat to the health of human beings.

As a structural defect of the heart at birth, CHD has similar major signs and symptoms to cardiovascular diseases, including rapid breathing, cyanosis, fatigue, and poor blood circulation, but do not hold the typical symptoms of other cardiovascular diseases, such as chest pain [4,5]. As a group of heart structural problems at birth, CHD can be further categorized into various subtypes based on detailed pathogenesis, including different regions of the cardiovascular system and similar symptoms [6]. Atrioventricular septal defect (AVSD) was previously described as common atrioventricular canal defect or endocardial cushion defect, but is found to be a clinically significant subtype of CHD, that accounts for over 5% of all CHD cases [7]. To geneticists, ASVD is particularly important because of its strong association with Down syndrome (DS) [4,8,9]. Babies with DS are likely to have AVSD with an incidence 2000 times higher than that in the euploid population. According to clinical statistics, approximately 15% to 20% of newborns with DS also suffer from the AVSD, indicating the potential relationship between these two diseases [10,11].

DS, also known as trisomy 21, is caused by a genetic disorder induced by the presence of all or part of a third copy of chromosome 21. The genetic contributions of trisomy 21 to the concurrence of DS and AVSD are more significant compared with other factors, even environmental factors. In 2012, a specific study [12] on CHD confirmed that variants in *CRELD1*, encoding an epidermal growth factor-related gene, contribute to the concurrence of DS and AVSD. In addition, researchers identified a group of *CRELD1* variants that affect the susceptibility of AVSD in patients with DS, thus validating the genetic causative component of AVSD, especially in DS populations [12]. In 2015, two studies [13,14] confirmed that genes *NKX2-5*, *GATA4*, and *CREL1* participate in the pathogenesis of AVSD in DS. In 2016, another study [15] focusing on all types of genetic contribution on AVSD in DS showed that not only the genetic variants but also other regulatory variants (e.g., altered microRNA expression) may participate in such pathogenesis. As a specific form of genetic variants, copy number variants (CNVs) have been widely identified in DS according to many recent publications. In 2017, a novel study provided by the Gene Expression Omnibus (GEO) database (GSE93004) [16,17] describing the CNVs anchored in chromosome 21 in DS confirmed that such a type of genetic variant may also affect the susceptibility of AVSD in patients with DS. However, this study did not reveal that the detailed regions/genes located on chromosome 21 that may contribute to the pathogenesis of AVSD in patients with DS. Therefore, in this study, we tried to find out an applicable computational method to identify the key genomic regions/genes that contribute to such pathogenesis.

Machine learning models provide a powerful solution to identify the crucial regions/genes associated with AVSD in patients with DS. These models have been used to analyze the CNV data for cancers [18,19]. For example, Ding et al. apply feature selection to identify crucial genes associated with cancer; these genes are further coded into conventional machine learning models to distinguish cancer samples from other control samples. However, these models still do not achieve high performance.

Deep learning has recently achieved remarkable results in computer vision and computational biology [20,21,22,23,24,25,26,27]. Most successful applications use many layers of convolutional or recurrent neural networks but are limited to vision or sequential tasks. For data without sequential characteristics or local structure, the feed-forward neural network (FNN) can be applied. Successfully using FNN with more than four layers is rarely reported. For deep network, the variations in the distributions of the activations are known as internal covariate shift [28], resulting in slow training and poor generalization [29,30,31]. In addition, training deep networks easily suffers from vanishing or exploding gradient [32], which can be avoided by stabilizing variance of activations. Thus, batch normalization [28] and other normalization tricks are applied to ensure zero mean and unit variance of activations and thus robustly learn many layers. However, these tricks are easily perturbed by stochastic gradient descent and dropout regularization. Both the convolutional neural network (CNN) and the recurrent neural network can cope with this issue using Rectified Linear Unit, weight sharing, or skip connection (residual network ResNet [33]). On the contrary, deep FNNs are sensitive to these perturbations and have high variance even when using all these techniques [32]. Currently, there are no effective techniques that can be applied for training deep FNNs. The self-normalizing neural network (SNN) [32] is proposed to handle the effect of the perturbations and build deep FNNs.

SNNs open the door for deep network applications on general data and are not limited to sequential and image data. It has been evaluated on 121 UCI tasks, and the results reveal that SNNs are superior to FNNs in all tasks and have outperformed random forests (RFs) and support vector machine (SVM) when the data size is greater than 1000. Furthermore, SNNs outperformed all other methods, including ultradeep CNNs, such as ResNet, on the Tox21 and astronomy dataset. The winning SNNs are deep [32].

In this study, we present machine learning-based methods to analyze CNV data of 236 patients with both DS and AVSD and 290 patients with only DS provided by the study from GEO we have mentioned above (GSE93004) [16]. Considering the high dimension of CNVs, we first apply feature selection methods to identify the informative genes that may contribute to the pathogenesis of AVSD. Then, we train a deep SNN model on those informative genes to classify patients with DS and AVSD from those only with DS. As a comparison, the powerful classification algorithm, RF [34], is also used to build classifiers and estimate their prediction abilities on distinguishing the two types of patient samples. With both qualitative and quantitative analyses, this study not only facilitates the identification of pathogenic genes contributing to AVSD in DS populations, but also lays a statistical foundation for further studies on the relationship and detailed mechanisms of CHD and DS.

## 2. Materials and Methods

**Figure 1:**
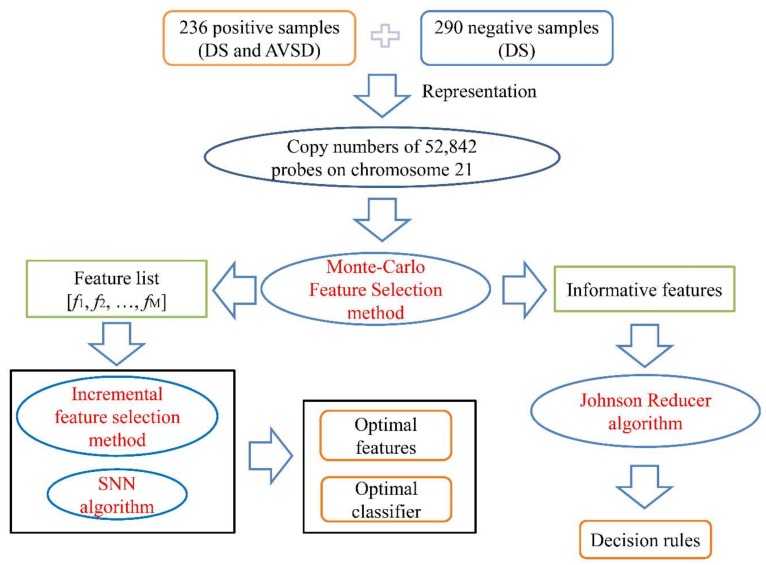
Flowchart of our proposed pipeline. 236 patients with both Down syndrome (DS) and atrioventricular septal defect (AVSD) (positive samples) and 290 patients had only DS (negative samples) were measured by the copy numbers of 52,842 probes on chromosome 21. Then, all features were evaluated by Monte Carlo feature selection method (MCFS), resulting in a feature list and several informative features. The feature list was used in the incremental feature selection method to construct an optimal self-normalizing neural network (SNN) classifier and extract optimal features. The informative features were feed into the Johnson Reducer algorithm to extract decision rules.

We presented a machine learning-based method to identify the pathogenic genes associated with AVSD in DS population (Figure 1). We first collect copy number data of total 526 samples. Then, we applied feature selection methods to yield the informative genes. These informative features are further fed into a deep SNN classifier to classify patients with DS and AVSD from those only with DS and fed into Johnson reducer algorithm to generate some decision rules with biological support.

### 2.1. Dataset

We downloaded the copy number data of 236 patients with both DS and AVSD and 290 patients who had only DS and did not have other simple forms of CHD [16] from GEO [35]. The copy numbers of 52,842 probes on chromosome 21 were measured using Agilent Comparative Genomic Hybridization arrays (Agilent, Santa Clara, CA, USA). By investigating the copy number difference between patients with both DS and AVSD and patients with only DS, we may find the key genomic regions or genes that trigger the AVSD for patients with DS.

### 2.2. Feature Analysis

In this study, each patient sample was represented by 52,842 features derived from the copy number of probes. To select some essential features that might contribute to discriminating patients with DS and AVSD from those only with DS, a reliable feature selection procedure was necessary to achieve the goal. To this end, we applied Monte Carlo feature selection (MCFS) [36] and incremental feature selection (IFS) methods. The details are described in the following sections.

#### 2.2.1. Monte Carlo Feature Selection Method

MCFS method is designed to rank informative features for supervised classifiers using sampling technique with replacement. In detail, a large number of decision tree classifiers are constructed, where each tree is grown from a bootstrapped dataset with a randomly selected feature subset. In addition, each feature *f* is assigned a score called relative importance (RI*_f_*). MCFS assigns greater RI*_f_* to feature *f* if it participates more in the classification using the tree classifiers. For each time, a feature subset is constructed with *m* features (*m* ≪ *M*, where *M* is the total number of features) and *t* tree classifiers are grown, wherein each of the *t* trees is trained by a random sampled training and test sets from the original training set. Repeating the abovementioned process *s* times, we obtained *s* feature subsets and a total of *t* × *s* tree classifiers. Then, for each feature, we calculate the score RI by estimating the overall number of splits involving this feature in all nodes of all constructed trees. Particularly, RI is estimated for feature *f* using the following equation:

(1) $${\mathrm{RI}}_{f}=\overset{s\times t}{\underset{\tau =1}{\sum }}{\left(\mathrm{wAcc}\right)}^{u}\underset{n_{f}\left(\tau \right)}{\sum }\mathrm{IG}\left(n_{f}\left(\tau \right)\right){\left(\frac{\mathrm{no}.\;\mathrm{in}\;n_{f}\left(\tau \right)}{\mathrm{no}.\;\mathrm{in}\;\tau }\right)}^{v},$$

where wAcc is the weighted accuracy for all samples, $\mathrm{IG}\left(n_{f}\left(\tau \right)\right)$ is the information gain of node $n_{f}\left(\tau \right)$, $\mathrm{no}.\;\mathrm{in}\;n_{f}\left(\tau \right)$ is the number of samples in $n_{f}\left(\tau \right)$, no. in τ is the number of samples in tree *τ*, and *u* and *v* are fixed real numbers. By default *u* and *v* are set to 1, and a detailed discussion on how to set parameters of MCFS method can be found in Draminski et al. study [36]. The wAcc is defined as follows:

(2) $$\mathrm{wAcc}=\frac{1}{c}\sum_{i=1}^{c}\frac{n_{ii}}{n_{i1}+n_{i2}+\ldots +n_{ic}},$$

where c reprents the number of classes and $n_{ij}$ denotes the number of samples from class i that are classified as class j. The $\mathrm{IG}\left(n_{f}\left(\tau \right)\right)$ is defined as follows:

(3) $$\mathrm{IG}\left(n_{f}\left(\tau \right)\right)=\mathrm{Entropy}\;\left(T\right)-\mathrm{Entropy}\;\left(T,f\right),$$

where T is the target variable (class label) of node $n_{f}\left(\tau \right)$, Entropy (T) is the entropy of the frequency table of variable T and Entropy (T,f) is the entropy of the frequency table of the two variables T and f. One way of building a decision tree is to repeatedly find the attribute that returns the highest information gain. Both *s* and *t* should be sufficiently large so that a feature has a great chance to appear in many randomly generated feature subsets. Even so, there is still a chance that a feature is totally ignored by the algorithm. However, the great majority of the features are properly ranked by the algorithm which is sufficient for the feature selection in our work.

By using MCFS software downloaded from home page of Dramiński [37], we ranked all features. As a result, we yielded a ranked feature list in descending order according to their RI values, which can be formulated as

(4) $$F=\left[f_{1},f_{2},\ldots ,f_{M}\right],$$

where *M* is the total 52,842 features.

#### 2.2.2. Incremental Feature Selection Method

Based on the ranked feature list yielded from MCFS, we attempted to further determine a group of optimal features with a supervised classifier, which would correctly distinguish the most of the two types of patient samples in our dataset. Many supervised classifiers can be used, such as SVM, RF, SNN, etc. In this study, we used SNN and RF (Section 2.3) as the supervised classifiers. Therefore, we performedIFS method on the ranked feature list. First, we constructed a series of feature subsets, formulated as $S_{1}^{1}$, $S_{2}^{1}$, …, $S_{l}^{1}$, where $S_{i}^{1}=\left[f_{1},f_{2},\ldots f_{i*k}\right]$, i.e., the *i*th feature subset contained the first *i* * *k* features in the original feature list. By using features in each feature subset to represent patient samples in dataset, one classifier was built. After testing all feature subsets, we would obtain good prediction performances in a feature interval represented as [*min*, *max*].

To exactly extract optimal features in this interval, another series of feature subsets, $S_{min}^{2}$, $S_{min+1}^{2}$, …, $S_{max}^{2}$, was also constructed. Similarly, we tested all feature subsets by building classifiers on them. We obtained a feature subset with the best performance. Finally, the features in this feature subset were denoted as optimal features for further utilization. We believed that the optimal features contained key chromatin segments, which can be used to distinguish different patient samples. Simultaneously, we obtained an optimal classifier built on these optimal features.

#### 2.2.3. Rule Extraction

**Figure 2:**
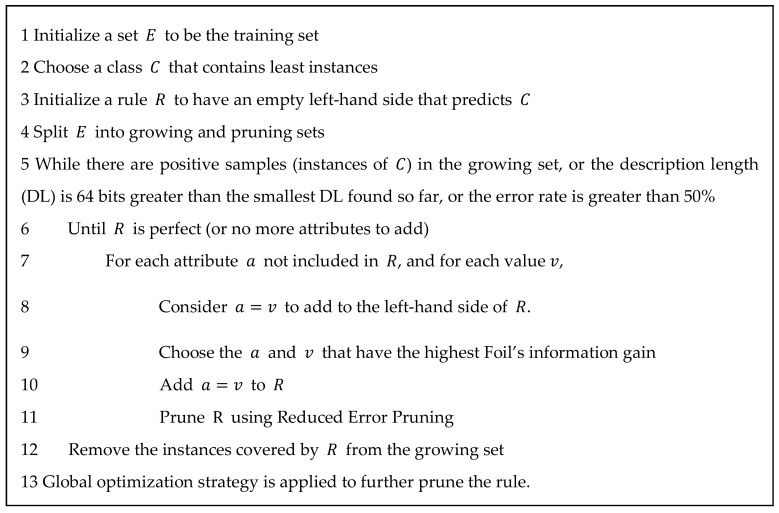
The procedures of RIPPER algorithm.

Based on the MCFS method, a feature list can be obtained as formulated in Equation (4). Informative features can be extracted from this list by selecting the top *p*% features in the list, where *p* is a predefined number. The Johnson Reducer algorithm [38] is then used to find a single reduction of the top *p*% features, which is a reduced subset of the features able to classify as well as using all of the features. Johnson Reducer algorithm is a greedy heuristic algorithm, which generates a reduction that is not guaranteed to have minimal size. Based on the reduction, Repeated Incremental Pruning to Produce Error Reduction (RIPPER) algorithm is applied to generate decision rules. The RIPPER, proposed by Cohen [39] in 1995, is a rule learning algorithm, which is capable of handling large noisy datasets effectively. It is an improved version of Incremental Reduced Error Pruning (IREP) [40], which combines both the separate-and-conquer technique used first in the relational learner FOIL, a system that learns Horn clauses from data expressed as relations [41], and the reduced error pruning strategy proposed by Brunk and Pazzani [42]. The RIPPER algorithm is described briefly in Figure 2.

In this study, the implementation of MCFS method from http://www.ipipan.eu/staff/m.draminski/mcfs.html integrates the rule extraction method mentioned above. By analyzing the extracted rules, a clearer comprehension can be achieved for DS population with AVSD. The rule extraction approach generates IF-THEN rules, which are more easily interpreted by a human being as they are similar to human language. In addition, it can narrow down the features to the most important ones which deserve special attention from related researchers.

### 2.3. Classification Algorithm

As described in Section 2.2.2, a classification algorithm is necessary to build classifiers on the feature subset derived from IFS method. In this study, a type of newly proposed neural network SNN and a widely used algorithm RF were applied. The mechanism on building classifiers is briefly listed as follows.

#### 2.3.1. Self-Normalizing Neural Network Algorithm

SNN [32] is proposed to solve the problems by keeping the mean (μ) and variance (v) of activations to a certain interval and making it (μ,v) converge to a fixed point, particularly to (0,1). Two techniques are adopted to achieve the self-normalizing properties as follows: (i) modify the activation functions; and (ii) initialize the weights of the network. The authors tweak an Exponential Linear Unit (ELU) activation function to obtain a Scale ELU (SELU) function.

(5) $$\mathrm{selu}\left(x\right)=\lambda \{\begin{array}{c} x\;\mathrm{if}\;x>0\\ \alpha e^{x}-\alpha \;\mathrm{if}\;x\leq 0 \end{array}$$

The authors [32] prove that after initializing the weights, SNN is indeed self-normalizing with the SELU function, and if v is too high and approaches the upper bound, it will decrease v, and if v is too low and approaches the lower bound, it will increase v.

In terms of initializing, Gaussian distribution with mean 0 and variance $\frac{1}{\sqrt{n}}$, where *n* is the number of weights, is applied. The authors prove that with such initializing and the fixed point being (0,1), SNN obtains an optimum solution λ=1.0507 and α=1.6733 in SELU function [32].

In addition, alpha dropout [32] is proposed to maintain the self-normalizing properties. Instead of using zero, which would perturb the mean and variance, the authors suggest using −αλ as the inputs of any dropout neurons. Then, the variance is preserved by applying an affine transformation ax+b where a,b can be computed relative to the dropout rate and the most negative activation.

In this study, all the constructed SNN classifiers have three hidden layers, each layer containing 200 hidden nodes.

#### 2.3.2. Random Forest Algorithm

RF is an ensemble classifier [43], which grows multiple decision trees. In the training stage, two statistical techniques including bootstrap method [44] and random feature subsets [45] are combined to build decision trees. In the procedure of bootstrap, a training dataset containing *N* samples is repeatedly sampled *B* times (*B* as a parameter representing the number of decision trees). For each decision tree, the randomly selected *N* samples (with replacement) comprise its training set, and a random feature subset is adopted to split the nodes of this decision tree. Eventually, *B* decision trees are grown. For a new sample, each decision tree provides a predicted result, and the predicted result of the RF was finally determined by majority voting. To date, it has been applied to tackle many biological problems [46,47,48,49,50,51,52,53,54,55].

The RF algorithm was implemented by the RandomForest classifier with default parameters in Weka [56] software, which contains some state-of-the-art machine learning algorithms.

### 2.4. Performance Measurements

To evaluate prediction ability of SNN classifier, we performed a 10-fold cross-validation [57,58,59,60]. Compared with jackknife cross validation test [61,62], the 10-fold test usually yielded a similar result.

As a binary classification problem, each positive or negative sample received a predicted class label from the constructed classifier. By comparing with their real labels, four values are calculated. They are true positive (TP), true negative (TN), false negative (FN), and false positive (FP) [63], where TP/FN is the number of positive samples that are predicted correctly/incorrectly, TN/FP is the number of negative samples that are predicted correctly/incorrectly. Based on the four values, a measurement called Matthew’s correlation coefficient (MCC) [64] is calculated to evaluate the prediction ability of classifier, defined as follows:

(6) $$MCC=\frac{TP\times TN-FP\times FN}{\sqrt{\left(TP+FP)(TP+FN)(TN+FP)(TN+FN\right)}}$$

The range of MCC is between −1 and 1. +1 represents a perfect prediction, 0 shows the prediction is close to random guessing and −1 indicates total disagreement between prediction and real labels. The predicted results were primarily evaluated using MCC because the two sample sizes in our dataset were slightly different.

In addition to MCC, we also employed the area under curve (AUC) for evaluating the performance of different classifiers. To calculate AUC, the receiver operating characteristic (ROC) curve should be plotted, which is defined by setting true positive rate (TPR) as its *Y*-axis and false positive rate (FPR) as its *X*-axis. Then, the AUC is defined as the value of area under the ROC curve. Generally, AUC is larger than 0.5 and a high AUC always implies good performance. 

## 3. Results

In this study, we used different copy numbers of probes of chromosome 21 as input features to distinguish patients with DS and AVSD from those with only DS. To evaluate these features on discriminating two types of patient samples, a MCFS method was used to rank all features in descending order according to their RI values using Monte Carlo method and decision trees. We selected the top 5000 features (listed in Supplementary Material S1) in feature list and executed IFS method on them because our goal was to extract important features and most of them are weakly correlated with patient samples.

### 3.1. Results from Self-Normalizing Neural Network Algorithm

**Figure 3:**
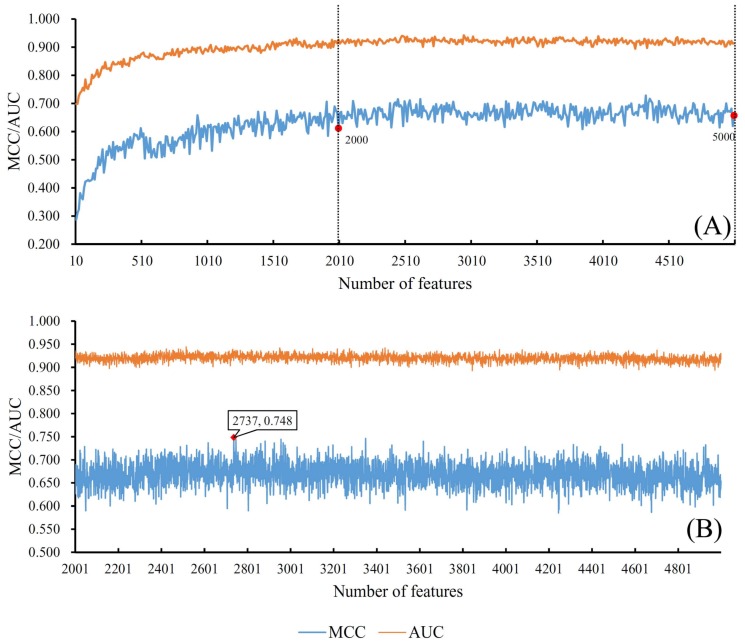
Incremental feature selection (IFS) curves derived from the IFS method and SNN algorithm. *X*-axis is the number of features participating in building classifiers in feature subsets. *Y*-axis is their corresponding Matthew’s correlation coefficient (MCC) or area under the curve (AUC) values. (**A**) IFS curve with X-values of 10 to 5000. The selected feature interval for SNN algorithm is [2001, 4999], which were marked with two vertical lines. (**B**) IFS curve with X-values of 2001 to 4999 for SNN algorithm. When first 2737 features in feature list were considered, the optimal MCC value reached 0.748, which is marked by a red diamond.

After the feature ranking procedure using MCFS, we obtained two series of feature subsets as introduced in Section 2.2.2. For the first series of feature subsets, the parameter *k* was set to 10. Thus, the *i*th feature subset contained top 10 * *i* features in the original feature list. Then, we constructed a SNN classifier on each feature subset, executed 10-fold cross validation test and calculated its MCC value. The obtained MCCs are listed in Supplementary Material S2. To provide a clear exhibition of these MCCs, we plotted an IFS curve using MCC as its *Y*-axis and the number used features as its *X*-axis, which is shown in Figure 3A. It can be observed that the IFS curve first follows a sharp increasing trend and then becomes stable. By careful checking, several good MCC values (most of them are larger than 0.600) were achieved by using number of features ranging from 2000 to 5000. Therefore, we determined a number interval [2001, 4999] for subsequent utilization.

The second series of feature subsets were built using the number of features in the number interval [2001, 4999]. Each feature subset had one more feature than the former one. Similarly, by testing all of them, we accessed several MCC values, which are also listed in Supplementary Material S2. Similarly, an IFS curve was also plotted to illustrate these values, which is shown in Figure 3B. The optimal MCC value (0.748) was yielded when top 2737 features were used to construct the SNN classifier (Table 1), which in turn demonstrated the discriminative ability of those top 2737 genes. In addition, we also ran the similar pipeline using RF as the classifier. The RF classifier yielded an optimum MCC 0.582 using optimum 132 features (see Section 3.2). RF yielded a much lower MCC than 0.748 of the SNN classifier, which demonstrated the power of deep SNN classifier. Accordingly, we obtained an optimal feature subset and an optimal SNN classifier.

**Figure 4:**
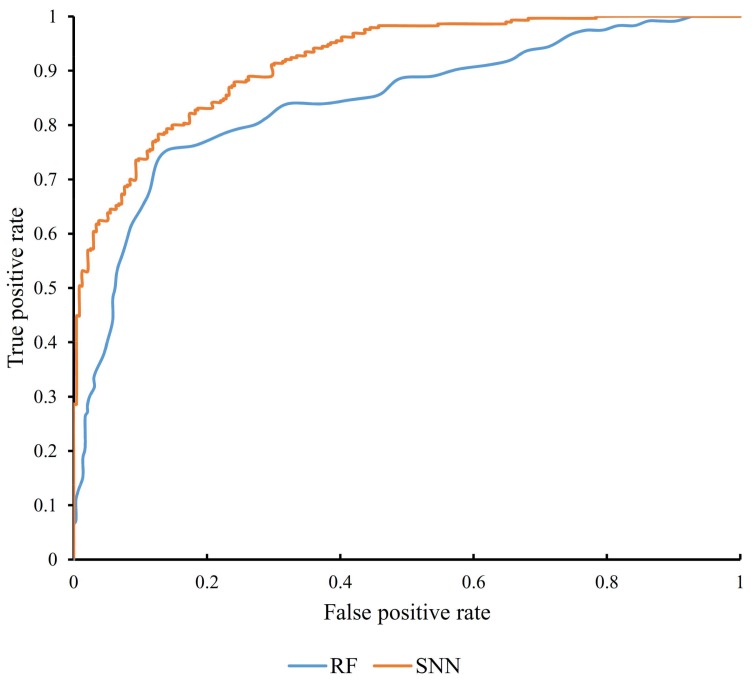
The receiver operating characteristic (ROC) curves for the optimal SNN and Random Forest classifier.

As mentioned in Section 2.4, to give a full evaluation of different classifiers, we also counted the AUCs for all SNN classifiers, which are provided in Supplementary Material S2. Also, we plotted the IFS curves for these AUCs, which are shown in Figure 3. It can be seen that the trends of these IFS curves are always similar to those for MCCs. In addition, for the optimal SNN classifier, its AUC (0.915) is listed in Table 1 and the corresponding ROC curve is illustrated in Figure 4.

### 3.2. Results from Random Forest Algorithm

**Figure 5:**
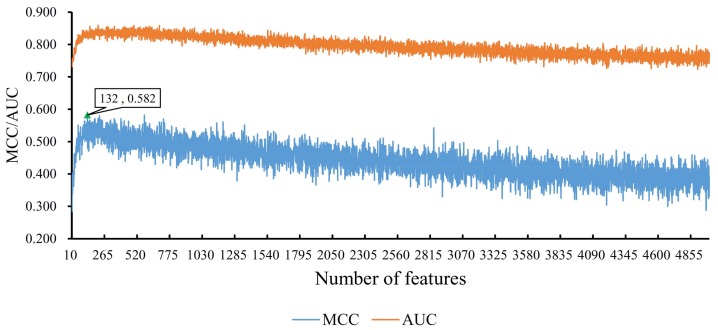
IFS curves derived from the IFS method and RF algorithm. *X*-axis is the number of features participating in building classifiers in feature subsets. *Y*-axis is their corresponding MCC or AUC values. When first 132 features in feature list were considered, the optimal MCC value reached 0.582, which is marked by a triangle.

Similar to SNN algorithm, we also applied RF algorithm to build classifiers on feature subsets derived from IFS method, and each classifier was evaluated by a 10-fold cross validation test. Because the RF algorithm is quite fast, all feature sets containing 10–5000 features were tested. The obtained MCC and AUC values are provided in Supplementary Material S3. Also, for a good observation, the IFS curves for MCC and AUC were plotted in Figure 5, from which we can see that the optimal MCC value is 0.582 when top 132 features in feature list were used (Table 1). Accordingly, the top 132 features and RF algorithm can construct the optimal RF classifier. The AUC yielded by this classifier was also calculated, it was 0.834 (Table 1). Obviously, the MCC and AUC obtained by the optimal RF classifier were much less than those obtained by the optimal SNN classifier. In addition, in most of the range of X-values, the corresponding MCC and AUC values of classifiers constructed using the RF algorithm were lower than those using the SNN algorithm (RF: majority of MCC values < 0.550 and all AUC values < 0.9, see Figure 5; SNN: majority of MCC values > 0.550 and all AUC values > 0.9, see Figure 3), indicating good prediction abilities of SNN classifiers on distinguishing complex biological samples.

### 3.3. Decision Rules

As mentioned in Section 2.2.3, IF-THEN rules were generated by the program of MCFS method, which are listed in Table 2. Two gene regions were involved in these rules: A_16_P41408273 and A_16_P03593084. Following these rules, the predicted results were measured as a MCC of 0.169. We also estimated an odds ratio for these rules of 2.01 (*p* = 2.62 × 10^−11^, 95% Confidence Interval (CI): lower, upper were 1.63, 2.48). These results also suggest that the predicted class from these rules was consistent with class of the observed disease groups. Although it is not very satisfactory, we can still find important clues that indicate the differences between DS populations with and without AVSD by analyzing A_16_P41408273 and A_16_P03593084, which is given below.

Three rules have been screened to identify two subgroups of DS populations with or without AVSD. In Rule 1, only one specific gene region named A_16_P41408273 in our reference database [16] has been screened out. According to this rule, patients with a reduction in copy number in A_16_P41408273 are more likely to have AVSD. Aside from our original database, minimal direct evidence confirmed the CNV of such region in patients with AVSD. Based on the GSE93004, GSE18152 from GEO database, and a specific publication [65], the copy number of such gene region is downregulated in most patients with AVSD compared with those without AVSD, validating our prediction. The Rule 2 of our quantitative screening involves two functional genes, *PDE9A* (A_16_P03593084) and A_16_P41408273 we mentioned above. The gene *PDE9A*, encoding a component of cGMP phosphodiesterase which further contributes to signal transduction by regulating the intracellular cyclic nucleotides’ concentration, has increased copy number in most patients with AVSD, consistent with our predicted rule (≥ −0.0164 and ≤ 0.075) [66,67]. As for transcript A_16_P41408273, we found that most patients with AVSD have a reduced copy number. However, a group of DS patients with AVSD who have an increasing copy number in A_16_P41408273 according to our reference dataset remains [65]. Combined with PDE9A, to speculate that all such samples screened out by Rule 2 are definitely DS patients with AVSD is quite reasonable and is consistent with our prediction. The successive application of Rule 1 and Rule 2 may identify patients with AVSD more accurately than either rule alone. Furthermore, according to our optimal rules, patients with DS with different copy number variant patterns compared with our previous two rules turn out to be patients without AVSD, corresponding with our original dataset.

## 4. Discussion

### 4.1. Why Use Self-Normalizing Neural Network as the Classifier

In this study, we integrated a SNN into feature selection method IFS, and achieved good prediction performance for classifying DS patients with or without AVSD. In addition, we also found some important genes and quantitative rules aligning with recent literature. However, there still exists some limitations: (i) the number of samples is much smaller than the number of input features, which easily leads to model overfitting; (ii) deep learning in general requires many more training samples, and currently we only trains SNN on a small dataset. Thus, if we can collect more samples, our method is expected to achieve better performance; (iii) SNN is still a black-box classifier, and currently we combine it with feature selection method to identify the important genes based on the discriminate performance; and (iv) training a deep learning model is still time-consuming.

As shown in Table 1, SNN performed much better than conventional machine learning classifier RF, which in general outperforms other conventional classifiers on many datasets. Meanwhile, we can train multiple classifiers to combine as a super learner, which is expected to perform better than any single classifier. However, it is time-consuming to train multiple classifiers. In particular, the classifier is integrated in IFS, which will run multiple times on feature subsets to select the optimum features. On the other hand, SNN itself outperforms other classifiers with a large margin. Thus, we just used SNN instead of a super learner.

### 4.2. Optimal Genes Associated with Atrioventricular Septal Defect in Patients with Down Syndrome 

Findings from our analysis suggest that patients with AVSD have specific copy number characteristics in DS populations compared with other patients with DS. Based on the copy number statistics provided in the GEO database, we apply our newly SNN-based method. Using our newly presented computational method, a group of functional genes (Table 3) with specific CNVs that may distinguish patients with AVSD in DS population were identified. The detailed analysis of screened important genes is listed below.

*PDE9A* (A_16_P03593084) has been predicted to have different copy number status in DS patients with or without AVSD. According to recent publications, *PDE9A* has been reported to contribute to the signaling transduction processes by specifically hydrolyzing the second messenger cyclic guanosine monophosphate (cGMP) [68,69]. In 2000 and 2011, two publications confirmed that in some patients with DS and in mouse models, *PDE9A*, predicted as an important gene in our model, has specific pathological CNV, validating the specific distribution of CNVs in such genes among DS populations [66,67]. *PDE9A* as a specific component of the cGMP signaling pathway has also been reported to participate in atrioventricular septal-associated diseases [70,71,72]. To identify patients with AVSD in DS populations by its specific atrioventricular septal associated functions is quite reasonable, further validating our prediction.

The gene *DOPEY2* (A_16_P03583086) is a known pathological gene of DS involved in the protein traffic between lately Golgi and early endosomes [73,74]. Recently, a comprehensive review [75] on the genome dosage imbalance in DS confirmed that the copy number alteration of this gene may be involved in the complications of disease, such as AVSD, aligning with our prediction. Considering that the CNVs of *DOPEY2* may participate in the pathogenesis of AVSD, to speculate that DS patients with or without AVSD may have different copy number status of gene *DOPEY2* is quite reasonable, validating the efficacy and accuracy of our prediction.

*LCA5L* (A_16_P03587947) encoding the ligand of *LCA5* has been widely reported to participate in centrosomal or ciliary functions according to recent publications [76,77]. Additionally, the interaction between *LCA5L* and *NDK1* has been confirmed to contribute to the pathogenesis of DS with specific CNVs [78,79,80]. Although no direct evidence revealed the specific contribution of *LCA5L* in patients with DS, a study [81] on the congenital heart defects confirmed that copy number variation of *LCA5L* also participated in the pathogenesis of congenital heart defects. In general, AVSD is a subtype of congenital heart defects. Therefore, considering that *LCA5L* simultaneously participate in the pathogenesis of both AVSD and DS, to conclude the potential relationship between LCA5L and DS patients with AVSD is quite reasonable.

*DSCR4* (A_16_P21251330) as a specific non-coding RNA gene that has been linked to the pathogenesis of DS is also predicted to contribute to distinguishing DS patients with or without AVSD [82]. The copy number alteration of this gene has also been reported to participate in AVSD-associated biological processes. In 2013, a specific study on children with DS implied that copy number alteration of *DSCR4* participate in the pathogenesis of DS patients with AVSD, functionally interacting with *CRELD1* [83]. In 2017, another study [84] also confirmed that in partial trisomy 21 cases, a specific gene region of *DSCR4* and its neighbor gene *KCNJ6* have been duplicated in patients with AVSD, compared with other patients without AVSD, validating our prediction.

*ITGB2* (A_16_P41466725) has also been predicted to contribute to AVSD in patients with DS. CNVs have been widely reported in *ITGB2* contributing to various subtypes of diseases, including DS [85], systemic lupus erythematosus [86], and lupus nephritis [86]. As a DS-associated gene, the CNV of *ITGB2* has also been reported to contribute to the specific complication of DS and CHD, indicating its complicated biological functions during such pathogenesis [87]. To consider *ITGB2* as a potential marker for the distinction of the two subgroups of DS is quite reasonable because the CNV of *ITGB2* has different distribution patterns in patients with and without AVSD [87], validating our prediction.

The last annotated gene in the top 10 predicted gene list is *U16296* (A_16_P41430034). As a specific annotated RNA isoform of gene *TIAM1*, the relationship between *U16296* and AVSD actually refers to the relationship between *TIAM1* and AVSD. Early in 2011, based on mouse embryo sequencing data, the CNVs of our predicted gene *TIAM1* has been reported to participate in the pathogenesis of DS-associated heart defects including AVSD, validating our prediction [88]. Furthermore, in 2004, a clinical study on a male infant with DS further confirmed the specific role of *TIAM1* translocation in the pathogenesis of CHD, aligning with our prediction [89]. Therefore, based on such literature supports, our predicted RNA isoform named U16296 derived from *TIAM1* may contribute to the identification of DS patients with AVSD.

Limited by the length of our manuscript, not all predicted genes/transcripts are discussed in detail. Filtering out the un-annotated RNA transcripts, many of the top predicted genes/transcripts have been reported to contribute to the distinction of DS patients with or without AVSD, providing a group of solid candidate biomarkers for further experimental confirmation and clinical detection.

## 5. Conclusions

Although lacking detailed copy number statistics for further validation, all screened rules have been confirmed to align with recent literature. Combined with the qualitative analysis of optimal distinctive genes we analyzed, our computational method successfully identified a group of functional biomarkers for the identification of patients with AVSD in patients with DS, which can help to understand why some DS patients develop AVSD. Although the model was still too complex to be applied in clinical practice, it included the candidate genes for further experimental validation, and the complex mechanism of AVSD and DS will be revealed one day.

## Figures and Tables

**Table 1:** Optimal number of features and MCC values yielded from the optimal SNN and RF classifiers.

Classification Algorithm	Number of Features	MCC	AUC
SNN	2737	0.748	0.915
Random forest	132	0.582	0.834

**Table 2:** Three decision rules extracted from the informative features.

Classification	Rules	Features	Criteria
With AVSD	Rule 1	A_16_P41408273	≤−0.00593
With AVSD	Rule 2	A_16_P03593084	≥−0.0164
		A_16_P03593084	≤0.075
		A_16_P41408273	≥0.0248
Without DS	Rule 3	Other conditions	

**Table 3:** Detailed analyzed optimal features in Section 4.2.

No.	Feature Name	Gene Name
1	A_16_P03593084	*PDE9A*
2	A_16_P03583086	*DOPEY2*
3	A_16_P03587947	*LCA5L*
4	A_16_P21251330	*DSCR4*
5	A_16_P41466725	*ITGB2*
6	A_16_P41430034	*U16296*

## Supplementary Materials

The following are available online at http://www.mdpi.com/2073-4425/9/4/208/s1, Supplementary Material S1: Output feature list derived from MCFS method with first 5000 features ranked by their RI values; Supplementary Material S2: Two series of MCCs and AUCs resulting from IFS method and SNN classifiers; Supplementary Material S3: MCCs and AUCs resulting from IFS method and RF classifiers.
