# Supplementary material for: Identifying Patients with Atrioventricular Septal Defect in Down Syndrome Populations by Using Self-Normalizing Neural Networks and Feature Selection
Source: Genes (Basel). 2018 Apr 12;9(4):208. doi: 10.3390/genes9040208 (PMC5924550; doi:10.3390/genes9040208)
Supplement: Supplementary file 1 [file genes-09-00208-s001.zip › Suppl_S1.pdf]

Supplementary Material S1. Output feature list derived from MCFS method with first 5,000 features ranked by their RI values.

Feature name	Projections	classifiers	Nodes	RI
GENE_A_16_P41408273	133	314	316	7.419
GENE_A_16_P03593084	145	241	242	7.798
GENE_A_16_P03583086	167	365	367	8.640
GENE_A_18_P13926919	164	271	278	8.064
GENE_A_16_P03587947	158	243	244	7.079
GENE_A_16_P21251330	150	271	276	5.804
GENE_A_16_P21200216	176	249	252	6.321
GENE_A_16_P41466725	139	210	214	4.795
GENE_A_16_P41430034	137	238	240	4.675
GENE_A_16_P58270038	141	215	216	4.664
GENE_A_16_P21272549	146	185	186	4.702
GENE_A_18_P13877404	144	183	184	4.506
GENE_A_16_P21221207	161	206	207	4.996
GENE_A_18_P13885289	139	196	196	4.172
GENE_A_18_P22508932	132	157	161	3.662
GENE_A_16_P41389893	145	164	173	3.955
GENE_A_16_P58266545	127	198	199	3.456
GENE_A_16_P58272455	162	132	132	4.131
GENE_A_18_P13894033	147	112	114	3.637
GENE_A_16_P58290676	143	131	132	3.530
GENE_A_18_P13885100	142	141	142	3.464
GENE_A_16_P03557267	135	141	142	3.160
GENE_A_16_P41439471	157	151	154	3.643
GENE_A_16_P58302227	179	153	154	4.071
GENE_A_16_P58297974	157	176	177	3.568
GENE_A_16_P58261137	152	128	129	3.389
GENE_A_16_P58275119	165	181	183	3.586
GENE_A_16_P03587490	155	112	112	3.361
GENE_A_16_P21253139	169	168	169	3.561
GENE_A_16_P03584079	161	157	158	3.340
GENE_A_18_P13914646	161	174	175	3.332
GENE_A_18_P13871234	170	136	137	3.505
GENE_A_16_P58281738	146	111	111	2.935
GENE_A_16_P41428190	146	109	109	2.929
GENE_A_16_P21199499	155	142	144	3.075
GENE_A_16_P58273213	166	143	145	3.167
GENE_A_16_P41466811	150	121	122	2.848
GENE_A_16_P21205029	162	170	172	3.052
GENE_A_16_P41407690	148	113	114	2.764
GENE_A_16_P58291425	147	121	123	2.673
GENE_A_16_P41414343	167	134	136	3.030
GENE_A_16_P21240676	147	100	103	2.664

GENE_A_16_P41453138	149	108	108	2.654
GENE_A_16_P58300439	150	115	118	2.662
GENE_A_16_P21243857	152	124	127	2.696
GENE_A_16_P34815756	158	103	103	2.801
GENE_A_16_P58295469	137	115	115	2.425
GENE_A_16_P41404890	153	115	116	2.690
GENE_A_16_P41460341	163	127	128	2.859
GENE_A_16_P03585823	134	109	111	2.343
GENE_A_16_P58308119	144	110	113	2.502
GENE_A_16_P41431008	144	115	116	2.501
GENE_A_16_P21263068	149	88	88	2.574
GENE_A_16_P03573977	155	109	110	2.631
GENE_A_16_P21194153	153	120	121	2.546
GENE_A_16_P03573323	144	113	113	2.292
GENE_A_18_P13926910	171	102	106	2.699
GENE_A_16_P21192551	154	105	105	2.399
GENE_A_18_P13944845	137	82	83	2.115
GENE_A_16_P58268657	149	94	95	2.253
GENE_A_16_P03579831	148	103	103	2.233
GENE_A_16_P58269185	140	89	90	2.063
GENE_A_18_P13934893	157	106	106	2.304
GENE_A_18_P13919845	145	103	105	2.118
GENE_A_16_P21237952	129	86	87	1.884
GENE_A_16_P21237367	162	86	88	2.357
GENE_A_16_P03586322	167	109	110	2.427
GENE_A_16_P03576985	153	72	73	2.207
GENE_A_16_P58274089	151	98	99	2.176
GENE_A_16_P21233544	142	98	98	2.040
GENE_A_16_P58300783	168	100	102	2.385
GENE_A_16_P58308874	148	76	76	2.097
GENE_A_16_P41400147	154	119	121	2.179
GENE_A_16_P58275223	154	81	82	2.171
GENE_A_16_P58287351	157	112	114	2.198
GENE_A_16_P58300641	143	89	91	2.000
GENE_A_16_P03584898	163	103	104	2.274
GENE_A_16_P03576542	145	81	81	2.020
GENE_A_16_P21233912	165	91	92	2.286
GENE_A_16_P21226562	141	96	96	1.949
GENE_A_16_P58287972	159	80	82	2.190
GENE_A_16_P58262537	154	77	77	2.101
GENE_A_16_P03577414	135	98	98	1.834
GENE_A_18_P13907430	159	93	94	2.135
GENE_A_16_P58289619	158	96	97	2.093
GENE_A_16_P41401278	153	102	102	2.011
GENE_A_16_P03579784	164	92	93	2.152
GENE_A_16_P21207547	154	68	68	2.019

GENE_A_16_P03588317	145	81	81	1.898
GENE_A_16_P21224887	134	75	79	1.749
GENE_A_16_P03580724	139	92	92	1.814
GENE_A_16_P41399222	159	79	82	2.070
GENE_A_16_P21246335	133	96	98	1.730
GENE_A_16_P03581646	145	77	78	1.882
GENE_A_16_P41426828	132	78	80	1.708
GENE_A_16_P58275881	140	90	93	1.808
GENE_A_16_P41399158	152	76	77	1.947
GENE_A_18_P22583234	161	92	93	2.059
GENE_A_16_P58278364	148	98	98	1.879
GENE_A_16_P21217005	135	87	87	1.713
GENE_A_16_P58277171	153	83	83	1.940
GENE_A_16_P21194939	152	112	112	1.922
GENE_A_18_P13933405	132	82	86	1.658
GENE_A_16_P58294940	149	98	99	1.858
GENE_A_16_P21245392	142	85	85	1.770
GENE_A_16_P21237178	164	104	105	2.044
GENE_A_16_P41398884	160	96	96	1.978
GENE_A_16_P21235365	148	79	81	1.817
GENE_A_18_P13904910	154	90	90	1.889
GENE_A_16_P58280694	141	58	58	1.725
GENE_A_16_P41398494	136	72	72	1.659
GENE_A_16_P21192533	140	75	75	1.676
GENE_A_16_P21240389	145	81	83	1.735
GENE_A_16_P03580926	132	58	59	1.574
GENE_A_16_P58283685	163	100	101	1.932
GENE_A_16_P03585115	143	65	66	1.689
GENE_A_16_P58287902	154	85	87	1.812
GENE_A_18_P22561155	153	77	78	1.798
GENE_A_16_P58305302	129	62	62	1.513
GENE_A_16_P21225031	146	69	70	1.706
GENE_A_16_P58286962	148	83	84	1.723
GENE_A_16_P58276798	159	84	84	1.848
GENE_A_16_P41442752	140	71	71	1.622
GENE_A_16_P41427810	148	76	77	1.699
GENE_A_16_P03575796	171	101	102	1.958
GENE_A_16_P03577776	162	80	80	1.849
GENE_A_16_P21253274	151	86	86	1.716
GENE_A_16_P58281088	128	60	60	1.452
GENE_A_18_P13885076	138	76	77	1.562
GENE_A_16_P58267505	143	73	74	1.617
GENE_A_16_P41423700	144	75	75	1.624
GENE_A_16_P21251340	143	69	70	1.605
GENE_A_18_P13870659	171	81	81	1.916
GENE_A_16_P03562873	157	94	94	1.759

GENE_A_16_P58265849	151	90	90	1.688
GENE_A_16_P58305388	152	95	96	1.680
GENE_A_16_P21271612	150	83	83	1.650
GENE_A_16_P03566324	162	75	75	1.765
GENE_A_18_P13887948	138	68	69	1.499
GENE_A_16_P03586959	159	65	65	1.713
GENE_A_16_P21214165	148	70	71	1.592
GENE_A_16_P21234994	150	72	72	1.612
GENE_A_16_P03575365	159	73	73	1.700
GENE_A_16_P03589228	164	93	95	1.743
GENE_A_16_P58262210	151	67	69	1.601
GENE_A_16_P21196628	132	60	60	1.395
GENE_A_16_P41469755	118	56	56	1.247
GENE_A_16_P03568027	169	89	89	1.775
GENE_A_16_P21219961	147	79	80	1.539
GENE_A_16_P21255646	157	77	77	1.636
GENE_A_16_P58265232	133	60	60	1.375
GENE_A_16_P21261458	136	54	55	1.398
GENE_A_16_P03555366	138	66	68	1.416
GENE_A_16_P21231234	161	80	80	1.650
GENE_A_16_P41427337	161	81	84	1.641
GENE_A_16_P21250588	169	66	67	1.723
GENE_A_16_P21240656	145	77	77	1.474
GENE_A_16_P03591209	157	92	92	1.588
GENE_A_16_P21213708	138	59	59	1.392
GENE_A_18_P13918493	145	69	69	1.461
GENE_A_16_P21244975	156	70	70	1.565
GENE_A_16_P41431790	155	79	80	1.555
GENE_A_16_P41406695	140	50	50	1.403
GENE_A_16_P03557380	155	75	75	1.536
GENE_A_16_P58295086	140	68	68	1.384
GENE_A_16_P58281394	152	71	71	1.502
GENE_A_16_P21224448	159	81	82	1.565
GENE_A_16_P21228578	153	79	79	1.504
GENE_A_16_P21246729	145	69	70	1.424
GENE_A_16_P21205844	123	46	46	1.207
GENE_A_18_P13914606	151	67	67	1.468
GENE_A_16_P21253880	174	92	93	1.689
GENE_A_18_P22556456	132	46	46	1.279
GENE_A_18_P22500830	151	65	66	1.452
GENE_A_16_P58290918	143	60	60	1.371
GENE_A_18_P22583269	186	63	63	1.773
GENE_A_16_P41454487	165	87	87	1.570
GENE_A_16_P21221113	159	69	70	1.498
GENE_A_18_P13889431	171	87	88	1.603
GENE_A_16_P21219964	169	94	94	1.577

GENE_A_16_P21248704	145	61	62	1.353
GENE_A_16_P41426129	147	77	78	1.364
GENE_A_16_P21248248	144	54	54	1.335
GENE_A_16_P21272296	154	69	70	1.421
GENE_A_16_P58273068	180	88	90	1.646
GENE_A_16_P41402565	133	58	58	1.208
GENE_A_16_P58271577	163	65	65	1.474
GENE_A_18_P13920176	174	91	92	1.570
GENE_A_16_P58278867	164	75	75	1.454
GENE_A_16_P58260068	160	72	73	1.416
GENE_A_16_P58275016	148	69	69	1.306
GENE_A_16_P21208955	149	62	62	1.311
GENE_A_16_P03595339	147	64	64	1.289
GENE_A_16_P21218979	177	83	83	1.552
GENE_A_16_P03588485	139	61	61	1.218
GENE_A_18_P13867158	149	63	65	1.303
GENE_A_16_P41391313	172	74	74	1.504
GENE_A_16_P03556074	178	70	71	1.553
GENE_A_16_P03580728	134	58	58	1.169
GENE_A_16_P21212542	150	62	62	1.301
GENE_A_16_P03576977	163	63	63	1.412
GENE_A_16_P21226041	161	67	67	1.389
GENE_A_16_P21190577	133	59	59	1.146
GENE_A_16_P58264653	139	66	66	1.196
GENE_A_16_P21253634	151	62	62	1.298
GENE_A_16_P03579786	171	58	59	1.469
GENE_A_16_P58290468	151	55	55	1.290
GENE_A_16_P58307144	154	55	56	1.314
GENE_A_16_P03556088	156	61	61	1.331
GENE_A_16_P03587253	155	62	62	1.322
GENE_A_16_P41401335	161	71	71	1.370
GENE_A_18_P13890273	147	54	54	1.250
GENE_A_16_P41419656	149	59	60	1.259
GENE_A_16_P21213745	134	50	51	1.131
GENE_A_16_P41413895	149	72	72	1.256
GENE_A_16_P21214786	147	67	67	1.231
GENE_A_16_P21232404	167	69	71	1.395
GENE_A_18_P13867296	157	73	74	1.310
GENE_A_16_P34811079	146	54	54	1.216
GENE_A_16_P58263772	150	68	68	1.243
GENE_A_18_P22573177	128	55	56	1.061
GENE_A_16_P58299604	150	60	60	1.236
GENE_A_16_P21224039	160	54	54	1.317
GENE_A_16_P03551407	142	49	49	1.165
GENE_A_16_P03584219	142	51	51	1.162
GENE_A_16_P41463628	130	51	51	1.060

GENE_A_16_P03576057	138	46	48	1.121
GENE_A_16_P41410453	168	62	62	1.364
GENE_A_18_P13904124	154	59	59	1.248
GENE_A_18_P13889749	147	57	57	1.190
GENE_A_16_P41401708	171	62	64	1.381
GENE_A_16_P03596152	149	60	61	1.203
GENE_A_18_P13884087	148	64	64	1.192
GENE_A_18_P13876058	152	59	59	1.223
GENE_A_16_P21248569	144	53	54	1.156
GENE_A_16_P03592779	152	54	54	1.220
GENE_A_16_P21192759	147	58	59	1.179
GENE_A_16_P21223519	161	63	64	1.290
GENE_A_16_P41416190	163	65	65	1.305
GENE_A_16_P21242783	163	62	63	1.303
GENE_A_16_P21245765	148	55	55	1.178
GENE_A_16_P21242947	133	58	58	1.058
GENE_A_16_P58261734	149	60	61	1.184
GENE_A_16_P21227071	137	43	45	1.088
GENE_A_16_P58307669	141	40	41	1.119
GENE_A_16_P58297009	159	54	57	1.262
GENE_A_16_P21219146	140	53	54	1.109
GENE_A_16_P41391412	154	61	61	1.219
GENE_A_16_P58266792	174	57	58	1.377
GENE_A_16_P41421471	143	51	52	1.127
GENE_A_16_P21222368	131	50	50	1.030
GENE_A_16_P58275950	170	66	67	1.333
GENE_A_16_P03597424	162	61	61	1.270
GENE_A_16_P21249299	142	50	50	1.113
GENE_A_18_P13875284	138	43	43	1.080
GENE_A_16_P58261804	149	60	60	1.165
GENE_A_16_P58262136	156	57	57	1.219
GENE_A_16_P21201076	153	40	40	1.194
GENE_A_18_P13918191	150	52	54	1.166
GENE_A_16_P21230892	143	64	66	1.111
GENE_A_16_P34772588	156	46	46	1.211
GENE_A_16_P58271576	161	59	59	1.250
GENE_A_16_P58308424	137	48	49	1.062
GENE_A_16_P21246644	140	59	59	1.082
GENE_A_16_P58298957	144	50	50	1.111
GENE_A_16_P21200062	153	53	54	1.176
GENE_A_18_P13864033	162	62	63	1.241
GENE_A_16_P58260114	174	59	59	1.326
GENE_A_16_P58299276	146	60	60	1.112
GENE_A_16_P03567576	157	56	57	1.188
GENE_A_16_P03597388	153	66	66	1.157
GENE_A_18_P13921978	163	51	51	1.231

GENE_A_16_P21253048	154	65	65	1.160
GENE_A_18_P13854404	160	45	46	1.201
GENE_A_16_P58298669	160	53	53	1.200
GENE_A_16_P41449184	155	53	54	1.162
GENE_A_16_P58294683	156	61	64	1.170
GENE_A_18_P22587799	150	61	61	1.125
GENE_A_16_P58283049	158	61	62	1.179
GENE_A_16_P34804678	164	45	45	1.220
GENE_A_18_P22549986	159	54	54	1.182
GENE_A_16_P41400113	154	57	57	1.145
GENE_A_18_P22573908	146	45	45	1.084
GENE_A_16_P03587037	153	56	56	1.129
GENE_A_16_P34777562	131	41	41	0.964
GENE_A_16_P58292004	155	48	48	1.140
GENE_A_16_P41407166	144	45	45	1.058
GENE_A_16_P41406320	138	49	49	1.012
GENE_A_16_P41416290	159	57	57	1.165
GENE_A_18_P13931607	184	54	54	1.343
GENE_A_16_P41418366	164	67	68	1.196
GENE_A_16_P03589361	160	53	53	1.167
GENE_A_16_P21228807	138	37	38	1.006
GENE_A_16_P41406429	146	57	57	1.063
GENE_A_16_P41400984	166	53	54	1.206
GENE_A_16_P58286851	153	43	43	1.111
GENE_A_16_P58266361	119	36	36	0.862
GENE_A_18_P13916728	159	63	64	1.151
GENE_A_16_P21201091	139	49	50	1.005
GENE_A_16_P21259494	131	43	43	0.947
GENE_A_16_P58292259	132	47	48	0.953
GENE_A_16_P03585686	148	55	56	1.068
GENE_A_18_P13864621	159	58	58	1.146
GENE_A_16_P58291858	154	67	67	1.110
GENE_A_16_P41417051	152	48	48	1.091
GENE_A_16_P41409477	157	60	60	1.127
GENE_A_18_P22577181	146	56	56	1.046
GENE_A_14_P104353	169	52	54	1.209
GENE_A_16_P03561251	167	59	59	1.194
GENE_A_16_P41458984	167	59	59	1.194
GENE_A_18_P22589356	180	68	69	1.284
GENE_A_18_P22486057	140	52	53	0.998
GENE_A_16_P58265935	155	53	53	1.104
GENE_A_16_P03552483	166	43	43	1.180
GENE_A_16_P03555312	176	63	64	1.250
GENE_A_16_P58293632	136	53	53	0.965
GENE_A_16_P58278858	170	52	52	1.206
GENE_A_16_P21198329	155	44	44	1.096

GENE_A_16_P58282515	155	53	54	1.093
GENE_A_16_P21239263	152	41	41	1.071
GENE_A_16_P58299911	137	46	46	0.965
GENE_A_16_P58290899	172	60	60	1.207
GENE_A_16_P41397930	158	54	56	1.108
GENE_A_16_P21231974	152	54	56	1.065
GENE_A_16_P21243819	163	69	71	1.135
GENE_A_16_P03572689	165	52	52	1.149
GENE_A_16_P41450867	143	50	50	0.996
GENE_A_16_P58301180	148	44	44	1.028
GENE_A_16_P21228472	148	49	50	1.028
GENE_A_16_P58280563	156	50	50	1.083
GENE_A_16_P58283470	166	50	50	1.151
GENE_A_16_P21262228	153	49	49	1.061
GENE_A_16_P21246008	144	35	35	0.996
GENE_A_18_P13893415	141	55	55	0.976
GENE_A_16_P21254345	142	37	37	0.980
GENE_A_16_P41408162	153	49	50	1.053
GENE_A_16_P03579477	140	54	55	0.963
GENE_A_16_P03590157	146	47	47	1.004
GENE_A_18_P13889721	156	50	51	1.072
GENE_A_14_P101341	143	48	49	0.978
GENE_A_18_P13864952	154	43	43	1.052
GENE_A_16_P21243847	140	49	49	0.954
GENE_A_18_P22582810	145	49	49	0.987
GENE_A_16_P58303800	161	51	51	1.096
GENE_A_16_P58304940	133	52	53	0.905
GENE_A_16_P58289867	136	46	46	0.923
GENE_A_16_P21202268	145	45	45	0.982
GENE_A_16_P03562798	140	40	40	0.945
GENE_A_18_P22496335	158	51	51	1.065
GENE_A_18_P13870780	163	42	44	1.099
GENE_A_16_P21219120	152	38	39	1.022
GENE_A_18_P22599388	153	39	42	1.027
GENE_A_16_P21244510	156	49	49	1.045
GENE_A_16_P58274139	147	46	46	0.983
GENE_A_16_P21232755	184	65	66	1.228
GENE_A_16_P58302201	142	55	56	0.948
GENE_A_16_P03589435	142	46	47	0.947
GENE_A_16_P21213648	136	44	45	0.905
GENE_A_18_P13897597	146	53	54	0.970
GENE_A_16_P58266506	145	50	50	0.961
GENE_A_16_P03567787	168	56	56	1.112
GENE_A_16_P03574364	168	50	50	1.111
GENE_A_16_P58294847	160	52	53	1.058
GENE_A_16_P41401596	125	37	37	0.825

GENE_A_16_P41421592	186	68	69	1.227
GENE_A_16_P41452968	137	44	45	0.903
GENE_A_16_P21205331	159	55	57	1.044
GENE_A_16_P21231205	158	47	47	1.037
GENE_A_16_P58265118	153	47	47	1.001
GENE_A_16_P58282345	142	43	43	0.929
GENE_A_16_P41395085	127	38	39	0.829
GENE_A_16_P41401383	140	39	39	0.912
GENE_A_16_P41398164	168	51	51	1.093
GENE_A_18_P22518689	154	43	43	1.002
GENE_A_16_P58296452	157	44	45	1.018
GENE_A_16_P21232586	161	53	53	1.044
GENE_A_16_P03579363	147	42	42	0.952
GENE_A_18_P22554603	162	45	45	1.047
GENE_A_18_P13938503	154	49	50	0.995
GENE_A_16_P41434481	161	50	53	1.040
GENE_A_16_P03585048	151	54	55	0.974
GENE_A_16_P41470093	143	50	51	0.922
GENE_A_16_P41416022	163	49	49	1.050
GENE_A_16_P21202932	150	50	50	0.966
GENE_A_16_P58304848	160	61	61	1.027
GENE_A_18_P13944852	149	50	50	0.956
GENE_A_16_P58262476	154	49	49	0.987
GENE_A_16_P21193937	154	50	51	0.987
GENE_A_16_P21212954	178	60	61	1.139
GENE_A_16_P03555984	156	51	51	0.994
GENE_A_16_P41464767	145	39	39	0.922
GENE_A_16_P58262610	144	49	49	0.915
GENE_A_16_P34793258	135	41	42	0.857
GENE_A_16_P41421283	149	45	46	0.945
GENE_A_16_P58301534	150	42	42	0.951
GENE_A_16_P03573216	156	44	44	0.986
GENE_A_16_P21255466	146	46	46	0.923
GENE_A_18_P13861999	146	42	42	0.917
GENE_A_18_P13855069	158	46	46	0.993
GENE_A_16_P58263165	124	49	49	0.778
GENE_A_16_P41414505	165	50	50	1.034
GENE_A_16_P03590560	136	42	42	0.852
GENE_A_16_P58261522	118	34	34	0.738
GENE_A_18_P13899148	141	43	43	0.881
GENE_A_16_P58271644	127	26	26	0.793
GENE_A_16_P21241049	150	45	45	0.935
GENE_A_16_P58275796	164	51	53	1.020
GENE_A_16_P41426414	170	59	59	1.056
GENE_A_16_P58307709	149	45	45	0.925
GENE_A_16_P21230094	147	48	49	0.910

GENE_A_16_P21231922	166	35	35	1.027
GENE_A_16_P21231177	130	36	37	0.804
GENE_A_16_P58280627	150	51	51	0.926
GENE_A_16_P41418597	153	49	50	0.944
GENE_A_16_P03583886	178	52	52	1.097
GENE_A_16_P58309612	144	42	43	0.886
GENE_A_16_P03592416	133	41	42	0.817
GENE_A_16_P03594858	154	46	47	0.944
GENE_A_16_P21200065	155	45	45	0.949
GENE_A_16_P21209798	134	39	39	0.820
GENE_A_18_P13916873	125	39	39	0.763
GENE_A_16_P03584273	166	55	56	1.009
GENE_A_16_P21217437	146	39	39	0.886
GENE_A_16_P21220831	132	47	47	0.800
GENE_A_16_P03578642	178	46	46	1.078
GENE_A_18_P13858353	166	44	45	1.005
GENE_A_18_P22566132	165	47	49	0.997
GENE_A_18_P13929854	152	45	45	0.917
GENE_A_16_P58268343	140	42	43	0.844
GENE_A_16_P21212724	146	42	43	0.880
GENE_A_16_P41442687	143	45	45	0.861
GENE_A_16_P58294660	153	40	40	0.921
GENE_A_18_P22505538	159	43	43	0.956
GENE_A_16_P58294426	152	39	39	0.914
GENE_A_18_P22538337	166	54	54	0.997
GENE_A_16_P41435949	168	41	41	1.008
GENE_A_16_P03590558	146	43	44	0.876
GENE_A_16_P58259782	134	36	36	0.803
GENE_A_16_P41389850	147	42	42	0.881
GENE_A_16_P41390427	151	44	46	0.904
GENE_A_16_P21206138	137	39	39	0.820
GENE_A_16_P41443419	158	52	52	0.942
GENE_A_16_P03590300	153	47	50	0.912
GENE_A_16_P03561293	157	43	46	0.936
GENE_A_18_P13938241	139	30	30	0.829
GENE_A_16_P03575839	149	30	30	0.888
GENE_A_18_P13865099	145	35	36	0.863
GENE_A_16_P58302866	145	42	43	0.863
GENE_A_16_P58274439	165	46	46	0.982
GENE_A_16_P58277275	138	35	35	0.820
GENE_A_18_P13860011	149	41	41	0.885
GENE_A_16_P58279801	153	51	51	0.907
GENE_A_16_P58260322	155	45	45	0.918
GENE_A_16_P21224036	145	39	39	0.858
GENE_A_16_P21251471	162	48	49	0.958
GENE_A_16_P21252158	154	43	45	0.910

GENE_A_16_P21251039	137	40	40	0.808
GENE_A_16_P58272968	142	45	45	0.837
GENE_A_16_P21250568	156	43	44	0.919
GENE_A_16_P21241136	129	26	26	0.759
GENE_A_16_P58272729	168	39	41	0.988
GENE_A_16_P21193882	141	38	39	0.828
GENE_A_16_P21202741	151	44	47	0.887
GENE_A_16_P21194469	144	35	37	0.845
GENE_A_16_P21216506	143	43	43	0.837
GENE_A_16_P41431380	175	51	51	1.022
GENE_A_16_P21209130	155	43	43	0.905
GENE_A_16_P58266631	137	32	32	0.799
GENE_A_18_P22493793	127	38	38	0.740
GENE_A_16_P58306985	153	45	45	0.891
GENE_A_16_P41455907	153	42	45	0.890
GENE_A_16_P58304027	142	31	31	0.825
GENE_A_16_P21232433	180	57	57	1.042
GENE_A_16_P03565058	149	46	46	0.862
GENE_A_16_P41468990	151	51	51	0.873
GENE_A_16_P58271362	147	43	44	0.850
GENE_A_16_P41423365	167	49	49	0.965
GENE_A_16_P21204615	172	44	45	0.993
GENE_A_16_P21218703	160	42	42	0.924
GENE_A_16_P21236080	152	40	41	0.877
GENE_A_16_P21210370	151	40	41	0.869
GENE_A_16_P58276945	127	38	39	0.731
GENE_A_16_P41437338	164	41	41	0.943
GENE_A_16_P03582742	124	39	39	0.712
GENE_A_16_P58301369	153	51	51	0.876
GENE_A_16_P58290406	154	39	39	0.881
GENE_A_16_P03592085	148	32	32	0.845
GENE_A_16_P58277400	151	40	40	0.861
GENE_A_16_P03555800	134	34	34	0.762
GENE_A_16_P21208702	139	29	29	0.789
GENE_A_16_P21203993	152	37	37	0.863
GENE_A_18_P22509056	181	49	50	1.027
GENE_A_16_P58294454	164	43	44	0.930
GENE_A_16_P03560104	158	42	44	0.894
GENE_A_16_P21204722	144	42	44	0.813
GENE_A_16_P03596809	132	36	37	0.745
GENE_A_16_P41409759	153	43	43	0.863
GENE_A_16_P03560741	146	38	38	0.821
GENE_A_16_P21193972	161	43	44	0.904
GENE_A_16_P58307810	153	42	42	0.858
GENE_A_16_P41436946	146	53	53	0.819
GENE_A_18_P13899404	148	41	42	0.829

GENE_A_16_P41390748	156	40	40	0.873
GENE_A_18_P22590255	184	56	56	1.028
GENE_A_18_P13902431	161	44	46	0.900
GENE_A_16_P41413316	149	44	46	0.832
GENE_A_16_P41456045	149	36	36	0.831
GENE_A_16_P03552982	163	58	59	0.908
GENE_A_16_P21235345	162	44	44	0.901
GENE_A_16_P41452892	161	42	43	0.895
GENE_A_16_P03587422	162	49	49	0.901
GENE_A_16_P03580001	138	38	40	0.767
GENE_A_16_P41416991	132	38	38	0.734
GENE_A_16_P21226905	147	41	41	0.816
GENE_A_16_P21209084	136	31	32	0.751
GENE_A_16_P03580477	158	32	32	0.873
GENE_A_16_P03565942	145	45	45	0.800
GENE_A_18_P13934433	161	41	42	0.887
GENE_A_16_P21212157	156	40	41	0.858
GENE_A_16_P58303981	153	53	53	0.841
GENE_A_16_P34801962	161	47	47	0.885
GENE_A_16_P41423393	160	49	50	0.878
GENE_A_16_P21218959	138	38	38	0.756
GENE_A_16_P03598246	161	41	42	0.880
GENE_A_16_P41441717	145	39	40	0.792
GENE_A_18_P13937934	156	39	39	0.851
GENE_A_16_P21243681	166	51	52	0.905
GENE_A_16_P58289031	157	42	42	0.852
GENE_A_16_P41463291	144	39	39	0.780
GENE_A_18_P13875252	145	37	37	0.785
GENE_A_16_P21200307	163	41	41	0.877
GENE_A_16_P21208993	137	38	39	0.737
GENE_A_16_P58267514	140	34	35	0.751
GENE_A_16_P58304623	142	41	43	0.760
GENE_A_16_P41410706	159	43	43	0.850
GENE_A_16_P58289981	138	37	39	0.737
GENE_A_16_P58294567	142	40	42	0.757
GENE_A_16_P21218049	138	40	40	0.735
GENE_A_16_P21193700	153	36	37	0.814
GENE_A_16_P41417766	145	45	46	0.771
GENE_A_18_P13889177	153	34	35	0.814
GENE_A_16_P21268256	155	44	44	0.824
GENE_A_16_P03573288	158	32	32	0.840
GENE_A_16_P58290383	154	39	40	0.818
GENE_A_16_P21201693	147	46	46	0.781
GENE_A_16_P58277848	161	41	41	0.855
GENE_A_16_P03562705	170	45	47	0.901
GENE_A_18_P13908378	138	36	36	0.730

GENE_A_18_P13917560	168	43	43	0.888
GENE_A_16_P58280030	154	34	37	0.812
GENE_A_16_P03590470	157	39	40	0.828
GENE_A_16_P03594058	156	42	44	0.820
GENE_A_16_P21205896	158	37	38	0.831
GENE_A_16_P41392945	188	46	46	0.987
GENE_A_16_P03584022	149	39	39	0.782
GENE_A_16_P21213714	142	34	35	0.745
GENE_A_16_P21202826	145	40	41	0.761
GENE_A_16_P58289122	161	38	42	0.843
GENE_A_18_P13881444	129	40	40	0.675
GENE_A_16_P58295598	141	46	46	0.737
GENE_A_16_P58295296	132	36	36	0.690
GENE_A_18_P13932424	153	44	45	0.799
GENE_A_16_P21212800	145	39	39	0.756
GENE_A_16_P21231086	143	35	36	0.745
GENE_A_16_P21204628	167	35	35	0.869
GENE_A_18_P13927711	164	44	45	0.853
GENE_A_16_P03573537	144	40	40	0.748
GENE_A_16_P41436428	168	43	44	0.873
GENE_A_16_P21270736	143	35	35	0.742
GENE_A_16_P41418310	162	33	33	0.840
GENE_A_16_P58272190	152	49	49	0.787
GENE_A_16_P58306598	164	32	33	0.848
GENE_A_16_P21196093	149	38	38	0.770
GENE_A_16_P58268615	160	42	42	0.826
GENE_A_16_P41408414	142	31	32	0.732
GENE_A_16_P58302265	158	35	35	0.814
GENE_A_16_P58290441	166	24	24	0.855
GENE_A_18_P22518176	184	46	46	0.947
GENE_A_18_P13883654	176	40	40	0.906
GENE_A_16_P58276227	153	38	38	0.786
GENE_A_16_P21221459	169	40	41	0.868
GENE_A_16_P58279342	137	34	34	0.704
GENE_A_16_P41444932	143	35	35	0.734
GENE_A_18_P13913528	148	36	36	0.759
GENE_A_16_P41400273	141	28	28	0.723
GENE_A_16_P41415646	157	37	37	0.802
GENE_A_16_P21265143	153	47	47	0.782
GENE_A_16_P58270816	158	38	39	0.806
GENE_A_16_P21247064	166	39	39	0.846
GENE_A_16_P03583504	138	30	30	0.704
GENE_A_16_P58296607	155	41	41	0.789
GENE_A_18_P22520638	142	35	36	0.722
GENE_A_16_P41420386	158	34	34	0.803
GENE_A_16_P03582129	148	40	40	0.750

GENE_A_16_P03564611	151	40	40	0.764
GENE_A_16_P21240042	169	41	41	0.855
GENE_A_18_P22526710	144	37	37	0.729
GENE_A_16_P41437504	163	41	42	0.823
GENE_A_16_P21257919	160	53	53	0.807
GENE_A_16_P21257582	153	29	29	0.771
GENE_A_16_P58259905	170	43	43	0.857
GENE_A_16_P03596344	165	47	47	0.831
GENE_A_18_P13927536	145	42	42	0.730
GENE_A_16_P03586380	138	37	37	0.693
GENE_A_18_P13894490	165	40	40	0.827
GENE_A_16_P21228461	151	41	42	0.757
GENE_A_16_P41408423	133	28	28	0.666
GENE_A_16_P03583677	159	31	31	0.796
GENE_A_16_P03575986	148	36	37	0.741
GENE_A_16_P21206222	147	29	29	0.735
GENE_A_16_P21252159	162	36	36	0.809
GENE_A_16_P21272543	158	33	34	0.788
GENE_A_16_P58285245	159	39	39	0.792
GENE_A_16_P58280924	165	37	37	0.822
GENE_A_16_P21197688	171	48	48	0.852
GENE_A_16_P03584645	175	41	41	0.870
GENE_A_16_P21205249	141	33	33	0.700
GENE_A_18_P13907111	130	35	35	0.643
GENE_A_18_P22520417	156	47	49	0.771
GENE_A_16_P58295099	151	42	42	0.745
GENE_A_16_P03580107	144	32	32	0.711
GENE_A_16_P58306120	159	43	43	0.784
GENE_A_16_P41409068	134	33	34	0.661
GENE_A_16_P21227082	143	32	32	0.705
GENE_A_16_P58271131	164	42	43	0.808
GENE_A_16_P21224524	155	36	36	0.763
GENE_A_16_P21251919	138	34	34	0.680
GENE_A_16_P41462796	143	39	39	0.704
GENE_A_16_P58284882	161	42	43	0.791
GENE_A_16_P41460613	149	38	39	0.731
GENE_A_18_P13924872	153	31	31	0.751
GENE_A_18_P13947514	165	41	42	0.810
GENE_A_16_P58275790	154	40	40	0.753
GENE_A_16_P34805273	159	43	44	0.777
GENE_A_16_P21243526	147	32	32	0.718
GENE_A_16_P21264119	146	33	33	0.712
GENE_A_16_P21214247	144	30	30	0.701
GENE_A_18_P22571299	154	40	41	0.750
GENE_A_16_P58285484	148	37	38	0.719
GENE_A_16_P58309498	138	31	31	0.669

GENE_A_16_P41402841	159	28	28	0.771
GENE_A_18_P13856612	125	30	30	0.606
GENE_A_16_P03556338	147	33	36	0.712
GENE_A_18_P22600376	165	44	44	0.799
GENE_A_18_P22593922	166	41	41	0.803
GENE_A_16_P21233422	149	39	39	0.720
GENE_A_16_P58292125	162	40	41	0.783
GENE_A_18_P13936268	147	27	27	0.710
GENE_A_16_P03577394	172	39	39	0.831
GENE_A_16_P58284189	144	42	43	0.695
GENE_A_16_P41416572	153	36	36	0.739
GENE_A_16_P21215041	139	31	31	0.670
GENE_A_16_P21242084	163	36	36	0.785
GENE_A_16_P03585327	175	31	31	0.843
GENE_A_18_P22508124	140	36	37	0.673
GENE_A_16_P58263039	141	34	35	0.677
GENE_A_18_P13934631	130	31	31	0.624
GENE_A_18_P22592771	153	31	31	0.734
GENE_A_16_P58264253	155	28	28	0.744
GENE_A_16_P58270036	153	41	41	0.733
GENE_A_16_P58261421	141	34	35	0.675
GENE_A_16_P21266407	158	37	37	0.756
GENE_A_16_P21246940	131	22	22	0.627
GENE_A_16_P03559922	133	29	30	0.636
GENE_A_16_P03597674	160	34	34	0.765
GENE_A_16_P58308238	161	38	38	0.770
GENE_A_16_P21250071	127	37	37	0.605
GENE_A_16_P58295094	157	36	36	0.748
GENE_A_16_P03585482	118	25	25	0.562
GENE_A_16_P03576536	142	34	34	0.676
GENE_A_18_P22500504	159	37	37	0.757
GENE_A_16_P58283125	154	33	34	0.733
GENE_A_16_P03582514	174	37	37	0.828
GENE_A_16_P03594829	172	39	39	0.818
GENE_A_16_P58272704	151	34	34	0.717
GENE_A_16_P03566518	153	28	29	0.726
GENE_A_16_P03598064	142	30	30	0.673
GENE_A_16_P21264322	172	42	42	0.814
GENE_A_18_P22551097	155	37	37	0.734
GENE_A_16_P03576956	163	37	38	0.770
GENE_A_16_P58300403	147	30	30	0.694
GENE_A_14_P102587	159	38	38	0.751
GENE_A_16_P58294128	141	28	29	0.666
GENE_A_16_P41427277	151	36	36	0.713
GENE_A_16_P58307907	142	29	29	0.670
GENE_A_16_P03572956	144	30	30	0.679

GENE_A_16_P41431764	159	34	34	0.749
GENE_A_16_P58266491	132	37	37	0.621
GENE_A_16_P58299551	142	32	32	0.668
GENE_A_16_P41393404	161	35	35	0.757
GENE_A_16_P58293775	150	38	40	0.704
GENE_A_18_P13858671	151	44	44	0.708
GENE_A_16_P21203947	155	41	41	0.726
GENE_A_16_P58281756	168	32	32	0.787
GENE_A_16_P03560316	154	44	44	0.721
GENE_A_16_P21239087	151	35	35	0.706
GENE_A_18_P13907242	155	37	38	0.723
GENE_A_16_P03555945	149	36	37	0.694
GENE_A_16_P58302782	146	27	27	0.680
GENE_A_16_P58289367	141	36	37	0.657
GENE_A_16_P03560797	147	40	40	0.684
GENE_A_16_P03576524	146	40	40	0.680
GENE_A_16_P58260200	136	36	36	0.633
GENE_A_18_P13888005	149	27	29	0.690
GENE_A_18_P22531988	150	38	38	0.695
GENE_A_16_P21272676	142	31	32	0.657
GENE_A_16_P41398267	143	37	39	0.662
GENE_A_16_P58260105	164	46	46	0.758
GENE_A_16_P21232617	147	32	32	0.679
GENE_A_16_P21194216	150	36	36	0.693
GENE_A_16_P21208710	138	30	30	0.637
GENE_A_16_P58274943	175	37	39	0.808
GENE_A_16_P03590159	167	38	39	0.770
GENE_A_18_P13909236	143	33	33	0.660
GENE_A_16_P03588873	146	31	32	0.673
GENE_A_16_P21203748	141	28	29	0.649
GENE_A_16_P58270327	149	27	27	0.686
GENE_A_16_P21263164	167	39	40	0.769
GENE_A_16_P21242607	148	30	30	0.681
GENE_A_16_P58282714	158	37	37	0.727
GENE_A_16_P41452163	146	26	27	0.671
GENE_A_16_P58309358	159	35	36	0.730
GENE_A_16_P03597308	161	38	38	0.739
GENE_A_18_P13863355	142	32	33	0.652
GENE_A_16_P58278941	173	30	30	0.794
GENE_A_16_P03594300	165	39	40	0.757
GENE_A_16_P21251766	172	39	39	0.789
GENE_A_16_P58293129	158	45	45	0.724
GENE_A_16_P21223681	142	31	32	0.650
GENE_A_16_P21226108	155	34	34	0.709
GENE_A_16_P58280274	173	37	38	0.791
GENE_A_16_P21219368	156	34	35	0.713

GENE_A_16_P21245793	187	42	42	0.854
GENE_A_16_P03591162	150	29	29	0.684
GENE_A_16_P21195507	142	29	31	0.645
GENE_A_16_P21242091	135	29	29	0.613
GENE_A_18_P13923540	144	46	46	0.653
GENE_A_16_P21231642	149	35	35	0.676
GENE_A_18_P13926939	158	37	38	0.716
GENE_A_18_P13892274	159	35	35	0.721
GENE_A_16_P21214594	139	30	31	0.630
GENE_A_16_P58277843	157	35	37	0.710
GENE_A_16_P41397623	175	43	43	0.790
GENE_A_16_P03579941	150	31	31	0.677
GENE_A_16_P03584662	148	39	39	0.668
GENE_A_16_P03551412	128	29	30	0.578
GENE_A_18_P13886579	147	35	35	0.663
GENE_A_16_P21208645	141	30	30	0.636
GENE_A_18_P13920628	160	31	31	0.722
GENE_A_16_P21192731	151	35	36	0.680
GENE_A_16_P21249898	148	30	30	0.666
GENE_A_18_P13898566	137	32	33	0.616
GENE_A_16_P03567060	152	38	38	0.683
GENE_A_18_P13878632	151	29	30	0.678
GENE_A_18_P22565982	148	29	29	0.664
GENE_A_16_P21216154	115	31	31	0.515
GENE_A_16_P21233194	156	34	34	0.697
GENE_A_16_P58304018	162	35	36	0.723
GENE_A_16_P03553040	145	32	33	0.647
GENE_A_16_P21266860	155	36	36	0.691
GENE_A_16_P58266039	144	29	29	0.641
GENE_A_16_P03596172	140	31	31	0.623
GENE_A_16_P58288996	136	28	28	0.605
GENE_A_16_P21272452	128	25	27	0.569
GENE_A_16_P58271530	145	31	31	0.644
GENE_A_16_P58286765	146	38	39	0.648
GENE_A_16_P03551810	145	34	34	0.644
GENE_A_16_P03585951	166	45	45	0.737
GENE_A_16_P21198962	129	30	30	0.572
GENE_A_18_P13934663	156	31	32	0.692
GENE_A_16_P58273592	166	37	37	0.735
GENE_A_16_P03581524	158	31	31	0.698
GENE_A_16_P03578549	150	32	33	0.662
GENE_A_18_P13854256	146	35	37	0.644
GENE_A_16_P03587307	163	33	33	0.719
GENE_A_16_P21202245	165	40	40	0.727
GENE_A_16_P03593661	152	31	32	0.670
GENE_A_18_P13882730	183	41	41	0.805

GENE_A_16_P03597696	152	33	33	0.669
GENE_A_16_P21220810	138	39	40	0.607
GENE_A_16_P58288173	143	32	33	0.629
GENE_A_16_P03575075	162	35	35	0.711
GENE_A_18_P13882357	166	33	33	0.729
GENE_A_16_P21193820	138	31	31	0.605
GENE_A_16_P03579382	150	38	38	0.658
GENE_A_18_P22587358	161	38	38	0.706
GENE_A_16_P03587526	158	26	26	0.692
GENE_A_16_P58304119	163	38	38	0.714
GENE_A_16_P21252051	158	28	28	0.691
GENE_A_16_P03567108	146	33	33	0.639
GENE_A_16_P41386924	137	37	37	0.599
GENE_A_16_P21200540	182	41	41	0.796
GENE_A_18_P22597408	145	32	32	0.634
GENE_A_16_P58303770	141	31	32	0.616
GENE_A_14_P121273	168	32	32	0.734
GENE_A_16_P41400501	159	33	33	0.694
GENE_A_16_P58306963	140	31	31	0.611
GENE_A_16_P03551604	140	30	31	0.611
GENE_A_16_P58308188	151	33	34	0.659
GENE_A_16_P41429541	126	32	32	0.549
GENE_A_16_P21217211	164	34	35	0.715
GENE_A_18_P13943653	140	27	28	0.610
GENE_A_16_P41405858	124	29	29	0.539
GENE_A_16_P41396052	157	27	27	0.681
GENE_A_16_P58284773	149	29	30	0.646
GENE_A_16_P03593767	159	34	34	0.690
GENE_A_16_P58297587	158	37	38	0.684
GENE_A_16_P41411581	147	36	37	0.636
GENE_A_16_P41437398	161	33	33	0.696
GENE_A_18_P22486729	146	33	34	0.632
GENE_A_16_P21225110	157	27	27	0.679
GENE_A_16_P21206759	137	24	24	0.592
GENE_A_16_P21208779	146	32	32	0.631
GENE_A_16_P03564200	169	36	37	0.730
GENE_A_16_P41466730	154	34	34	0.665
GENE_A_16_P41460653	134	23	23	0.579
GENE_A_18_P13892804	140	26	26	0.604
GENE_A_16_P58308018	156	29	29	0.673
GENE_A_16_P03558327	150	29	30	0.647
GENE_A_18_P22506518	158	36	37	0.681
GENE_A_16_P58301782	155	32	33	0.668
GENE_A_16_P58288557	175	36	36	0.753
GENE_A_16_P41426459	143	30	30	0.615
GENE_A_16_P58280697	176	40	40	0.757

GENE_A_16_P41415871	128	29	29	0.551
GENE_A_16_P03589793	144	32	33	0.620
GENE_A_16_P03573409	155	36	36	0.667
GENE_A_18_P13879556	150	31	31	0.645
GENE_A_16_P58274053	153	35	38	0.658
GENE_A_16_P21229783	146	36	37	0.627
GENE_A_16_P03582643	155	38	38	0.665
GENE_A_16_P21245176	130	27	27	0.558
GENE_A_16_P58272533	166	31	31	0.712
GENE_A_16_P21232828	154	28	28	0.660
GENE_A_16_P58297060	159	29	29	0.682
GENE_A_16_P58268902	164	35	36	0.703
GENE_A_16_P21208681	149	40	40	0.638
GENE_A_16_P21203812	173	38	40	0.740
GENE_A_16_P21202721	148	25	25	0.633
GENE_A_16_P03563255	146	30	30	0.624
GENE_A_18_P13946992	152	37	37	0.650
GENE_A_16_P03591764	146	36	36	0.623
GENE_A_16_P58268479	149	34	34	0.636
GENE_A_16_P58277130	134	29	29	0.572
GENE_A_16_P03594214	157	32	32	0.670
GENE_A_16_P03584840	147	33	34	0.626
GENE_A_16_P21255929	161	28	28	0.686
GENE_A_16_P21263643	148	32	32	0.630
GENE_A_18_P13911443	156	29	29	0.664
GENE_A_16_P58268544	148	34	34	0.629
GENE_A_16_P58262723	128	29	29	0.544
GENE_A_16_P58263433	156	34	35	0.663
GENE_A_16_P41395614	144	34	36	0.612
GENE_A_16_P34789507	140	32	33	0.594
GENE_A_16_P58263839	161	39	39	0.683
GENE_A_16_P58288656	147	33	33	0.623
GENE_A_16_P03573719	142	29	29	0.601
GENE_A_18_P22516155	153	31	31	0.648
GENE_A_16_P41390612	153	32	32	0.647
GENE_A_18_P22589094	143	33	33	0.604
GENE_A_18_P13865344	140	29	30	0.591
GENE_A_16_P41436292	153	35	36	0.646
GENE_A_16_P21256601	143	30	30	0.603
GENE_A_16_P58293814	140	24	24	0.591
GENE_A_16_P58277064	166	43	43	0.700
GENE_A_16_P41421103	129	25	25	0.544
GENE_A_16_P58276192	158	32	32	0.666
GENE_A_18_P22498529	149	39	40	0.627
GENE_A_16_P21240282	161	27	28	0.678
GENE_A_16_P03552396	160	35	35	0.674

GENE_A_16_P58257478	144	27	28	0.606
GENE_A_18_P22558062	158	34	35	0.665
GENE_A_16_P21213128	162	39	40	0.682
GENE_A_16_P58299299	166	23	23	0.698
GENE_A_16_P03575143	128	28	29	0.538
GENE_A_16_P21232301	160	36	36	0.672
GENE_A_16_P21211778	140	31	32	0.588
GENE_A_16_P41401855	134	31	32	0.563
GENE_A_16_P58262935	169	38	39	0.709
GENE_A_16_P58288482	158	35	35	0.663
GENE_A_16_P41400026	159	31	32	0.667
GENE_A_16_P21215180	184	35	36	0.772
GENE_A_16_P03573901	153	32	32	0.640
GENE_A_16_P21261955	172	36	37	0.719
GENE_A_16_P58305474	143	26	26	0.598
GENE_A_16_P58294009	138	21	21	0.576
GENE_A_16_P21236043	141	33	33	0.588
GENE_A_16_P58304292	157	34	34	0.655
GENE_A_16_P58261136	151	37	38	0.629
GENE_A_16_P21248473	167	26	27	0.696
GENE_A_16_P41453235	132	30	30	0.550
GENE_A_16_P41414046	143	26	26	0.596
GENE_A_16_P58305538	119	26	26	0.495
GENE_A_16_P21261795	153	32	32	0.636
GENE_A_16_P58285924	146	21	21	0.606
GENE_A_16_P21269143	139	26	26	0.577
GENE_A_16_P21214967	173	36	37	0.718
GENE_A_18_P13911361	143	31	32	0.593
GENE_A_16_P21245894	147	26	27	0.610
GENE_A_16_P58308062	151	31	31	0.626
GENE_A_16_P03557639	153	36	37	0.634
GENE_A_16_P21194019	149	35	35	0.616
GENE_A_18_P13872834	136	27	28	0.562
GENE_A_16_P03573713	136	30	30	0.561
GENE_A_16_P58284074	151	33	33	0.622
GENE_A_16_P21213168	147	26	28	0.606
GENE_A_16_P58298067	164	30	30	0.676
GENE_A_18_P13907727	149	33	33	0.614
GENE_A_18_P13906320	153	32	32	0.629
GENE_A_16_P21233381	132	27	27	0.543
GENE_A_16_P21248573	128	25	25	0.526
GENE_A_18_P13910213	152	31	33	0.625
GENE_A_16_P58289104	178	30	31	0.731
GENE_A_18_P22521072	153	29	29	0.627
GENE_A_18_P13904024	143	32	32	0.586
GENE_A_16_P03591765	150	33	33	0.614

GENE_A_16_P21202422	169	38	39	0.692
GENE_A_16_P41416286	158	31	32	0.647
GENE_A_16_P58298767	159	33	34	0.650
GENE_A_16_P58278802	139	29	29	0.568
GENE_A_16_P21250920	135	24	24	0.551
GENE_A_16_P58284871	171	37	37	0.698
GENE_A_16_P21231426	166	32	32	0.678
GENE_A_16_P58268773	140	27	27	0.571
GENE_A_16_P41404256	177	34	34	0.721
GENE_A_18_P13944535	164	34	34	0.668
GENE_A_16_P41432285	127	19	19	0.517
GENE_A_16_P03580945	142	30	30	0.578
GENE_A_16_P58302519	171	37	37	0.696
GENE_A_16_P58260330	155	35	35	0.630
GENE_A_16_P03566385	169	34	34	0.687
GENE_A_16_P03552160	146	30	30	0.594
GENE_A_16_P41423623	138	26	27	0.561
GENE_A_16_P03575343	146	26	27	0.593
GENE_A_16_P21203818	140	28	28	0.569
GENE_A_16_P58265348	137	27	28	0.557
GENE_A_16_P03567678	141	27	27	0.572
GENE_A_16_P58279654	143	26	26	0.580
GENE_A_18_P22576901	136	27	27	0.552
GENE_A_18_P13860165	150	29	29	0.608
GENE_A_16_P58287831	156	33	34	0.631
GENE_A_16_P03563744	151	30	31	0.611
GENE_A_16_P58260337	154	25	25	0.623
GENE_A_16_P21212249	174	36	36	0.704
GENE_A_16_P58279889	139	32	32	0.562
GENE_A_18_P13938995	146	24	24	0.590
GENE_A_16_P03591537	145	31	31	0.586
GENE_A_16_P41437069	142	25	25	0.574
GENE_A_18_P22548725	142	22	22	0.573
GENE_A_16_P03583117	162	34	35	0.653
GENE_A_16_P58298993	146	31	31	0.588
GENE_A_16_P58279594	144	28	28	0.580
GENE_A_16_P03587137	138	21	21	0.556
GENE_A_18_P13858328	195	38	38	0.785
GENE_A_16_P21246969	154	25	26	0.620
GENE_A_16_P21264815	144	21	21	0.580
GENE_A_16_P03582332	164	35	35	0.660
GENE_A_16_P58266570	147	31	31	0.591
GENE_A_18_P22485147	131	32	35	0.527
GENE_A_16_P21246737	146	30	30	0.587
GENE_A_16_P03583531	146	32	32	0.586
GENE_A_16_P21234324	164	31	31	0.659

GENE_A_18_P13884056	148	29	29	0.593
GENE_A_16_P58288209	137	30	30	0.549
GENE_A_16_P21193170	154	35	35	0.617
GENE_A_16_P21204437	166	27	27	0.665
GENE_A_18_P22513215	142	35	36	0.569
GENE_A_16_P21208708	146	31	31	0.585
GENE_A_16_P41429539	154	31	31	0.617
GENE_A_16_P34815721	141	24	24	0.564
GENE_A_18_P13923262	140	28	28	0.560
GENE_A_16_P21211323	155	33	33	0.620
GENE_A_16_P58276217	153	31	31	0.612
GENE_A_18_P13880141	164	30	30	0.656
GENE_A_16_P21197741	147	26	26	0.588
GENE_A_16_P03573860	143	28	29	0.571
GENE_A_16_P21221605	148	25	27	0.591
GENE_A_16_P21230831	159	37	37	0.634
GENE_A_16_P41401397	169	35	35	0.674
GENE_A_16_P03569743	164	34	34	0.654
GENE_A_16_P03595822	151	22	22	0.602
GENE_A_16_P21218859	152	28	28	0.606
GENE_A_18_P13858470	144	34	34	0.574
GENE_A_16_P58298857	159	25	25	0.633
GENE_A_16_P03574443	167	37	37	0.665
GENE_A_16_P34792067	146	28	28	0.581
GENE_A_16_P03598311	148	32	32	0.588
GENE_A_16_P58281573	170	36	36	0.675
GENE_A_16_P58264996	152	25	25	0.603
GENE_A_16_P41417983	161	38	38	0.639
GENE_A_16_P03576112	146	29	29	0.579
GENE_A_16_P21230870	157	28	28	0.622
GENE_A_16_P58291130	142	36	36	0.563
GENE_A_16_P41421617	150	31	31	0.594
GENE_A_16_P21242098	153	27	28	0.606
GENE_A_18_P13899178	172	35	35	0.680
GENE_A_16_P03585192	162	36	36	0.640
GENE_A_18_P13877545	169	35	35	0.668
GENE_A_18_P22591074	157	35	36	0.620
GENE_A_16_P21259430	143	24	25	0.565
GENE_A_18_P22501294	146	33	33	0.576
GENE_A_16_P03583200	135	32	32	0.533
GENE_A_16_P03577137	144	26	26	0.568
GENE_A_16_P21257018	175	38	38	0.689
GENE_A_16_P58267465	177	33	33	0.697
GENE_A_18_P22491874	140	27	27	0.551
GENE_A_16_P03562833	153	38	38	0.602
GENE_A_16_P58263492	129	22	22	0.507

GENE_A_16_P41468736	152	31	31	0.598
GENE_A_16_P21270278	154	35	35	0.605
GENE_A_16_P58260377	148	31	32	0.580
GENE_A_16_P58277167	168	31	32	0.658
GENE_A_16_P58294745	177	38	38	0.692
GENE_A_16_P21210544	173	39	39	0.677
GENE_A_16_P21253423	142	27	27	0.555
GENE_A_16_P21207598	139	28	28	0.543
GENE_A_18_P13887892	157	28	28	0.613
GENE_A_18_P13911410	146	25	26	0.570
GENE_A_18_P22594890	161	24	24	0.629
GENE_A_16_P58267950	142	31	31	0.554
GENE_A_16_P58289736	149	19	19	0.581
GENE_A_16_P41396047	168	39	39	0.655
GENE_A_16_P58261450	149	27	27	0.581
GENE_A_16_P41438938	163	28	28	0.636
GENE_A_16_P21263686	130	27	27	0.506
GENE_A_16_P58260968	148	30	30	0.576
GENE_A_16_P03581453	162	36	38	0.630
GENE_A_16_P58269380	157	35	35	0.610
GENE_A_16_P58271753	147	30	30	0.571
GENE_A_16_P21214268	147	30	30	0.571
GENE_A_16_P58265561	148	27	27	0.575
GENE_A_16_P21250808	154	33	33	0.598
GENE_A_16_P03583592	143	29	29	0.555
GENE_A_16_P41425226	170	36	37	0.659
GENE_A_16_P58299913	161	34	35	0.624
GENE_A_16_P58300528	148	32	32	0.574
GENE_A_16_P21207623	148	29	30	0.574
GENE_A_16_P21206207	148	32	32	0.573
GENE_A_16_P41411245	171	25	25	0.662
GENE_A_16_P21218244	151	27	27	0.585
GENE_A_16_P21215392	161	25	25	0.623
GENE_A_16_P21218381	136	28	29	0.526
GENE_A_16_P21231105	147	27	27	0.568
GENE_A_16_P58276816	161	27	27	0.622
GENE_A_16_P58295876	182	28	28	0.703
GENE_A_16_P58287755	171	32	33	0.660
GENE_A_18_P13870720	136	21	21	0.525
GENE_A_16_P58281591	147	27	27	0.567
GENE_A_16_P58270779	150	27	27	0.578
GENE_A_16_P21235066	140	30	32	0.540
GENE_A_16_P21199425	155	25	25	0.597
GENE_A_18_P22556607	163	35	35	0.627
GENE_A_18_P13928501	143	28	28	0.550
GENE_A_16_P21226520	119	24	24	0.458

GENE_A_18_P13866623	158	26	26	0.607
GENE_A_16_P41455506	163	37	37	0.626
GENE_A_16_P21196657	166	33	34	0.638
GENE_A_16_P58309417	151	31	32	0.580
GENE_A_16_P21202952	153	25	25	0.587
GENE_A_16_P41449486	158	29	29	0.607
GENE_A_18_P13884463	133	22	22	0.510
GENE_A_16_P58289978	152	27	27	0.583
GENE_A_18_P13890545	133	26	26	0.510
GENE_A_16_P58281800	147	25	26	0.563
GENE_A_16_P21250257	169	29	31	0.648
GENE_A_18_P22589351	146	28	29	0.559
GENE_A_16_P58299381	169	24	25	0.647
GENE_A_16_P41398237	140	29	29	0.536
GENE_A_16_P58279287	145	28	28	0.554
GENE_A_18_P13872723	142	29	29	0.543
GENE_A_16_P21209280	127	27	29	0.485
GENE_A_16_P03574265	138	29	29	0.527
GENE_A_16_P21215243	157	30	30	0.600
GENE_A_16_P21202966	134	30	30	0.512
GENE_A_16_P58272447	141	22	23	0.538
GENE_A_16_P41441024	153	25	25	0.584
GENE_A_16_P03580971	156	30	30	0.595
GENE_A_18_P13860216	131	25	25	0.500
GENE_A_18_P22597196	163	30	30	0.622
GENE_A_16_P58298489	159	27	27	0.605
GENE_A_16_P03577139	152	33	33	0.578
GENE_A_16_P58268279	163	30	30	0.620
GENE_A_16_P21228415	180	32	32	0.684
GENE_A_16_P21250901	158	31	31	0.600
GENE_A_16_P41392482	162	32	32	0.615
GENE_A_16_P58297321	125	23	23	0.474
GENE_A_16_P21198100	142	26	26	0.539
GENE_A_16_P58285405	187	36	36	0.709
GENE_A_16_P58289569	142	20	21	0.538
GENE_A_16_P03597051	150	29	29	0.569
GENE_A_16_P58262968	145	24	25	0.550
GENE_A_16_P21200621	151	30	30	0.572
GENE_A_16_P58285950	147	25	25	0.557
GENE_A_16_P21212355	155	29	29	0.587
GENE_A_18_P22539499	130	27	27	0.492
GENE_A_16_P03585118	153	24	24	0.579
GENE_A_16_P58263261	145	32	32	0.548
GENE_A_16_P03553144	144	26	27	0.543
GENE_A_16_P58269632	138	23	23	0.520
GENE_A_16_P21252225	153	29	29	0.577

GENE_A_16_P03576912	166	31	31	0.625
GENE_A_16_P21221092	144	25	26	0.542
GENE_A_16_P03567696	137	19	19	0.515
GENE_A_16_P21216993	145	26	26	0.545
GENE_A_16_P21267889	166	31	32	0.624
GENE_A_16_P58283728	166	31	31	0.624
GENE_A_16_P21219713	144	29	29	0.541
GENE_A_16_P03587856	151	24	24	0.568
GENE_A_16_P41448771	155	29	29	0.582
GENE_A_16_P21268254	154	29	29	0.578
GENE_A_18_P13948564	155	30	30	0.582
GENE_A_16_P21207436	156	28	28	0.585
GENE_A_18_P22540839	158	33	33	0.592
GENE_A_16_P58262600	145	29	29	0.544
GENE_A_18_P22599703	151	33	33	0.566
GENE_A_16_P21247203	153	25	25	0.573
GENE_A_18_P22494891	152	24	24	0.569
GENE_A_18_P13857267	145	26	26	0.543
GENE_A_16_P21214385	146	23	26	0.547
GENE_A_16_P58302523	155	32	32	0.580
GENE_A_16_P58276818	152	25	26	0.569
GENE_A_16_P58290242	129	24	24	0.483
GENE_A_16_P03588733	149	29	29	0.557
GENE_A_18_P22568748	159	29	29	0.595
GENE_A_16_P03580110	161	25	25	0.602
GENE_A_18_P13891202	158	27	28	0.591
GENE_A_16_P58267002	144	22	22	0.538
GENE_A_16_P58272728	158	21	21	0.591
GENE_A_16_P03582904	137	31	31	0.512
GENE_A_16_P03582933	167	33	33	0.624
GENE_A_16_P03551624	136	22	22	0.508
GENE_A_16_P21262501	143	29	30	0.534
GENE_A_16_P03587267	138	25	25	0.515
GENE_A_16_P21261614	138	23	25	0.515
GENE_A_16_P21243325	164	32	32	0.611
GENE_A_18_P13900585	151	26	27	0.563
GENE_A_16_P21258998	161	27	27	0.600
GENE_A_16_P41389109	147	33	33	0.547
GENE_A_16_P55129880	147	28	28	0.547
GENE_A_16_P58283570	156	29	29	0.580
GENE_A_16_P21218965	170	35	37	0.632
GENE_A_16_P03590467	150	28	29	0.558
GENE_A_16_P03592678	164	31	31	0.610
GENE_A_16_P58260356	135	29	30	0.502
GENE_A_16_P03557591	136	29	29	0.505
GENE_A_16_P03584435	147	30	30	0.545

GENE_A_18_P13931139	177	35	35	0.656
GENE_A_16_P03578439	164	29	29	0.608
GENE_A_16_P58292204	133	27	28	0.493
GENE_A_16_P03594707	181	25	25	0.670
GENE_A_16_P03590987	152	24	24	0.562
GENE_A_16_P41442506	152	26	26	0.562
GENE_A_16_P41413762	141	29	29	0.521
GENE_A_16_P58266609	176	29	29	0.650
GENE_A_16_P58287327	162	31	31	0.598
GENE_A_16_P03556252	142	24	24	0.524
GENE_A_16_P21220871	157	33	33	0.579
GENE_A_16_P58266321	156	26	26	0.575
GENE_A_16_P21233182	146	30	30	0.538
GENE_A_18_P13880482	150	26	26	0.553
GENE_A_16_P03553097	152	25	25	0.560
GENE_A_16_P03595771	142	26	26	0.523
GENE_A_16_P58289698	154	28	29	0.567
GENE_A_18_P13898658	159	27	27	0.585
GENE_A_16_P21267910	143	26	26	0.526
GENE_A_16_P58264888	159	27	28	0.585
GENE_A_18_P13876046	149	29	29	0.548
GENE_A_16_P41392273	162	31	31	0.595
GENE_A_16_P58302977	156	26	26	0.573
GENE_A_16_P21230747	144	23	23	0.529
GENE_A_16_P58301326	162	29	29	0.594
GENE_A_16_P41429911	137	21	21	0.502
GENE_A_16_P41418755	138	22	23	0.506
GENE_A_16_P03583649	140	26	26	0.513
GENE_A_16_P58272894	167	29	29	0.612
GENE_A_16_P58293285	142	24	25	0.520
GENE_A_16_P41431270	153	31	31	0.561
GENE_A_16_P58296920	153	30	30	0.561
GENE_A_16_P41415245	139	27	27	0.509
GENE_A_16_P21257058	148	38	38	0.542
GENE_A_16_P03596544	151	29	29	0.552
GENE_A_16_P41412651	164	26	26	0.600
GENE_A_16_P21263666	134	27	28	0.490
GENE_A_16_P21249848	149	30	30	0.545
GENE_A_16_P41398628	157	28	28	0.574
GENE_A_16_P03576973	157	27	27	0.574
GENE_A_18_P13914151	162	33	33	0.592
GENE_A_16_P41412367	158	25	25	0.576
GENE_A_18_P13936866	170	34	34	0.619
GENE_A_16_P58297431	152	30	30	0.554
GENE_A_16_P03566062	146	36	38	0.532
GENE_A_16_P21238254	162	30	30	0.589

GENE_A_18_P13921025	139	25	25	0.506
GENE_A_16_P03571275	167	31	31	0.607
GENE_A_16_P21227489	148	29	29	0.538
GENE_A_16_P03578115	165	32	32	0.599
GENE_A_16_P03568906	150	25	25	0.545
GENE_A_18_P13920729	153	32	32	0.555
GENE_A_16_P21248228	170	32	34	0.617
GENE_A_16_P58269875	168	27	27	0.609
GENE_A_18_P13919468	138	25	25	0.500
GENE_A_18_P13858759	140	35	35	0.507
GENE_A_16_P41423030	134	26	26	0.485
GENE_A_16_P41428798	141	27	27	0.510
GENE_A_16_P58288358	146	21	21	0.528
GENE_A_16_P34804819	150	31	32	0.542
GENE_A_16_P21234238	127	18	18	0.459
GENE_A_16_P03591207	165	29	29	0.596
GENE_A_16_P21221017	151	25	27	0.545
GENE_A_16_P03571968	151	32	33	0.545
GENE_A_16_P58308293	157	28	29	0.565
GENE_A_16_P41438267	142	27	27	0.511
GENE_A_16_P21251288	157	23	23	0.565
GENE_A_16_P21224797	145	29	30	0.522
GENE_A_18_P22557682	173	33	33	0.622
GENE_A_16_P21233227	150	24	24	0.539
GENE_A_16_P41427057	174	32	32	0.626
GENE_A_18_P13906913	154	28	28	0.554
GENE_A_16_P58295063	158	28	28	0.568
GENE_A_16_P58260977	157	33	33	0.564
GENE_A_16_P21234093	151	30	30	0.542
GENE_A_16_P21207377	150	31	31	0.539
GENE_A_16_P21203979	153	31	31	0.549
GENE_A_18_P13947240	159	28	29	0.571
GENE_A_16_P03579518	137	24	25	0.492
GENE_A_18_P13889715	164	32	32	0.588
GENE_A_16_P03581410	148	26	26	0.531
GENE_A_16_P03592864	146	23	23	0.523
GENE_A_16_P21199043	146	25	25	0.523
GENE_A_16_P58297586	171	30	30	0.612
GENE_A_16_P58275255	142	29	32	0.508
GENE_A_16_P03585341	140	27	27	0.501
GENE_A_16_P58271391	149	28	29	0.533
GENE_A_16_P21205413	151	28	28	0.540
GENE_A_16_P58307034	157	28	28	0.561
GENE_A_16_P41415981	150	23	23	0.536
GENE_A_16_P58305622	157	31	32	0.561
GENE_A_18_P13932574	140	22	22	0.500

GENE_A_16_P41413845	156	31	31	0.557
GENE_A_18_P13920798	152	29	30	0.543
GENE_A_16_P58300062	158	26	26	0.564
GENE_A_16_P21230800	126	24	24	0.450
GENE_A_16_P41455098	149	26	26	0.532
GENE_A_16_P58305378	155	26	27	0.553
GENE_A_16_P58267626	153	31	31	0.545
GENE_A_16_P41392054	150	25	25	0.534
GENE_A_16_P21206380	165	34	34	0.587
GENE_A_16_P41442171	144	28	28	0.512
GENE_A_16_P21202152	139	26	26	0.495
GENE_A_16_P21203933	144	27	27	0.512
GENE_A_16_P03551565	145	29	29	0.515
GENE_A_18_P13930293	139	21	21	0.494
GENE_A_16_P41429513	160	27	27	0.568
GENE_A_16_P21261686	146	28	28	0.518
GENE_A_16_P21194324	176	28	28	0.624
GENE_A_16_P21270985	123	21	21	0.436
GENE_A_16_P58305602	164	25	26	0.582
GENE_A_16_P03578655	162	30	30	0.574
GENE_A_16_P03588823	158	27	27	0.560
GENE_A_16_P58281478	161	31	31	0.570
GENE_A_16_P58269192	143	31	31	0.506
GENE_A_16_P58262432	136	26	26	0.481
GENE_A_16_P03575580	147	25	26	0.520
GENE_A_16_P41451553	140	24	24	0.495
GENE_A_16_P21248222	159	29	29	0.562
GENE_A_16_P21211034	136	22	23	0.480
GENE_A_18_P13902826	157	30	30	0.554
GENE_A_18_P13909687	126	24	24	0.445
GENE_A_18_P13884878	158	29	29	0.558
GENE_A_16_P21233549	151	26	26	0.532
GENE_A_16_P58278122	146	22	25	0.515
GENE_A_16_P58295196	149	27	27	0.525
GENE_A_16_P41443736	144	31	31	0.508
GENE_A_18_P13899982	135	24	24	0.476
GENE_A_16_P03556197	135	22	22	0.475
GENE_A_16_P21222737	148	27	28	0.520
GENE_A_16_P58274180	161	29	29	0.565
GENE_A_18_P13914406	153	29	29	0.537
GENE_A_16_P21192804	143	31	31	0.502
GENE_A_16_P58271532	157	20	20	0.551
GENE_A_16_P41415407	136	21	21	0.477
GENE_A_16_P58291141	146	27	27	0.512
GENE_A_16_P58298052	169	24	24	0.593
GENE_A_16_P58262451	140	31	31	0.491

GENE_A_16_P41408104	147	20	22	0.515
GENE_A_16_P58281323	168	31	31	0.588
GENE_A_16_P58275669	150	25	25	0.525
GENE_A_16_P41437313	160	27	27	0.559
GENE_A_16_P41444561	148	27	27	0.517
GENE_A_16_P41442297	149	28	28	0.520
GENE_A_16_P21208999	155	30	30	0.541
GENE_A_16_P41406347	138	21	21	0.482
GENE_A_14_P133284	140	19	19	0.489
GENE_A_16_P58302827	171	29	29	0.596
GENE_A_16_P03589678	130	22	23	0.453
GENE_A_16_P03582759	168	28	29	0.586
GENE_A_16_P41457187	147	23	23	0.512
GENE_A_16_P21209379	180	38	38	0.627
GENE_A_16_P21202642	146	27	27	0.508
GENE_A_16_P03592593	150	24	24	0.522
GENE_A_16_P41422035	164	26	26	0.571
GENE_A_16_P21200243	161	32	33	0.560
GENE_A_16_P41416367	165	30	30	0.574
GENE_A_16_P21210967	161	31	31	0.560
GENE_A_16_P58260743	146	26	27	0.508
GENE_A_16_P21197945	168	25	25	0.584
GENE_A_16_P03577015	147	23	23	0.511
GENE_A_16_P03592252	123	23	23	0.427
GENE_A_16_P58283988	155	28	28	0.538
GENE_A_16_P58299827	126	25	25	0.437
GENE_A_16_P03596593	148	26	27	0.513
GENE_A_16_P21220122	158	31	31	0.548
GENE_A_16_P03552541	178	31	31	0.617
GENE_A_16_P03550652	159	27	28	0.551
GENE_A_16_P21259740	141	17	17	0.489
GENE_A_14_P137127	142	25	27	0.492
GENE_A_16_P41389416	166	28	28	0.575
GENE_A_16_P21229247	138	25	25	0.477
GENE_A_16_P21209876	142	21	21	0.491
GENE_A_16_P21212306	152	25	27	0.526
GENE_A_16_P58279518	142	26	26	0.491
GENE_A_16_P58298665	150	27	27	0.518
GENE_A_16_P41425139	140	30	30	0.484
GENE_A_16_P21209414	167	34	34	0.577
GENE_A_16_P03562133	134	21	21	0.463
GENE_A_16_P21231031	153	19	19	0.529
GENE_A_16_P21255991	144	24	24	0.498
GENE_A_16_P41391458	140	24	24	0.483
GENE_A_18_P13870052	148	27	27	0.511
GENE_A_18_P13891424	157	33	33	0.542

GENE_A_16_P41446979	163	27	27	0.563
GENE_A_16_P21227313	145	24	25	0.500
GENE_A_16_P58287593	134	23	23	0.462
GENE_A_16_P41411065	170	33	34	0.586
GENE_A_16_P21223162	136	20	21	0.469
GENE_A_16_P58266645	163	31	31	0.562
GENE_A_16_P41468222	154	25	25	0.530
GENE_A_16_P21233329	166	30	30	0.571
GENE_A_16_P58267193	135	25	26	0.464
GENE_A_18_P22488092	142	27	29	0.488
GENE_A_16_P41416371	161	35	36	0.553
GENE_A_16_P03582115	150	28	28	0.515
GENE_A_16_P58293363	165	28	28	0.567
GENE_A_16_P03593015	154	26	26	0.529
GENE_A_16_P34767649	137	21	21	0.470
GENE_A_16_P58271076	142	23	23	0.488
GENE_A_18_P13884121	137	21	21	0.470
GENE_A_16_P58287650	151	28	28	0.518
GENE_A_18_P13857750	144	22	22	0.494
GENE_A_16_P58264294	138	23	23	0.473
GENE_A_16_P58284729	117	21	21	0.401
GENE_A_16_P03596757	159	28	28	0.545
GENE_A_18_P22574579	144	27	27	0.494
GENE_A_18_P13936279	144	29	29	0.493
GENE_A_18_P13873178	135	22	22	0.462
GENE_A_16_P03553016	156	31	31	0.534
GENE_A_16_P03592685	132	20	20	0.452
GENE_A_16_P58271292	141	29	29	0.483
GENE_A_16_P58284985	159	26	27	0.544
GENE_A_16_P03592134	156	26	26	0.533
GENE_A_16_P21210783	148	28	29	0.506
GENE_A_16_P21257332	155	25	25	0.530
GENE_A_16_P58274209	148	22	22	0.505
GENE_A_16_P21214936	174	28	29	0.594
GENE_A_16_P21201252	145	21	21	0.495
GENE_A_16_P58299453	137	23	23	0.468
GENE_A_18_P22585659	150	24	24	0.512
GENE_A_16_P58306891	158	29	29	0.539
GENE_A_16_P21240719	172	38	38	0.587
GENE_A_16_P03587932	164	32	32	0.559
GENE_A_16_P21208026	154	25	25	0.525
GENE_A_16_P58300945	136	22	22	0.464
GENE_A_16_P58296454	138	26	26	0.470
GENE_A_16_P21220463	154	28	29	0.525
GENE_A_16_P03582496	151	22	22	0.514
GENE_A_16_P58260082	151	27	28	0.514

GENE_A_18_P13942945	149	27	27	0.507
GENE_A_16_P58289437	147	25	25	0.500
GENE_A_18_P22569928	139	21	21	0.473
GENE_A_16_P41393696	130	22	22	0.442
GENE_A_16_P41435283	161	32	32	0.548
GENE_A_16_P58275429	154	25	26	0.524
GENE_A_16_P03582837	145	24	24	0.493
GENE_A_18_P13876104	132	20	20	0.449
GENE_A_16_P58268180	138	25	25	0.469
GENE_A_16_P58262325	135	20	20	0.458
GENE_A_16_P58289234	165	28	28	0.560
GENE_A_18_P22599316	160	23	23	0.543
GENE_A_16_P58302748	159	21	21	0.539
GENE_A_16_P03597446	144	25	25	0.488
GENE_A_16_P58264037	143	24	25	0.485
GENE_A_18_P13876639	163	20	20	0.553
GENE_A_16_P58305764	154	28	28	0.521
GENE_A_16_P58304003	139	25	25	0.470
GENE_A_16_P58293144	143	22	22	0.484
GENE_A_16_P21198989	170	31	31	0.575
GENE_A_18_P13864065	141	19	21	0.476
GENE_A_16_P03552494	152	27	27	0.514
GENE_A_18_P13938788	147	25	25	0.496
GENE_A_16_P58296129	156	27	27	0.527
GENE_A_16_P21198675	161	26	28	0.544
GENE_A_16_P03594778	131	22	22	0.442
GENE_A_16_P03592817	143	30	31	0.482
GENE_A_16_P41423478	153	25	26	0.516
GENE_A_16_P03575216	136	22	22	0.459
GENE_A_16_P03572018	145	26	26	0.489
GENE_A_16_P58261934	140	26	26	0.472
GENE_A_16_P58298037	169	27	27	0.570
GENE_A_16_P03591062	149	25	25	0.502
GENE_A_18_P13925319	140	18	18	0.472
GENE_A_16_P21224351	133	24	24	0.448
GENE_A_16_P41393825	142	24	24	0.478
GENE_A_18_P13866759	156	24	24	0.525
GENE_A_16_P58290636	175	23	23	0.589
GENE_A_16_P21199342	150	26	26	0.505
GENE_A_16_P21235319	153	29	29	0.515
GENE_A_16_P58279643	155	21	22	0.521
GENE_A_16_P21251691	159	23	23	0.535
GENE_A_16_P34772513	137	22	22	0.461
GENE_A_16_P03559559	146	29	29	0.491
GENE_A_16_P21194373	159	25	25	0.534
GENE_A_16_P03556521	149	27	27	0.501

GENE_A_18_P13905044	142	25	25	0.477
GENE_A_16_P21213518	162	27	27	0.544
GENE_A_16_P21246440	147	25	25	0.493
GENE_A_16_P58286262	155	27	28	0.520
GENE_A_16_P41425993	133	21	21	0.446
GENE_A_16_P58283387	150	24	24	0.503
GENE_A_16_P58280326	153	30	30	0.513
GENE_A_16_P58277554	165	27	28	0.553
GENE_A_16_P21206744	150	27	27	0.503
GENE_A_16_P21221242	132	24	24	0.442
GENE_A_16_P41466933	156	24	24	0.523
GENE_A_16_P21239005	161	27	27	0.539
GENE_A_16_P21223901	153	22	22	0.512
GENE_A_16_P03580035	149	23	23	0.498
GENE_A_16_P58306418	177	31	31	0.591
GENE_A_18_P13908923	133	24	24	0.444
GENE_A_18_P13887260	130	21	21	0.434
GENE_A_18_P22551754	148	24	24	0.494
GENE_A_16_P58270151	151	25	26	0.504
GENE_A_16_P03567429	141	21	21	0.470
GENE_A_18_P13915572	135	19	19	0.450
GENE_A_16_P21252937	138	24	24	0.460
GENE_A_16_P58278426	137	24	24	0.456
GENE_A_16_P41414181	156	27	27	0.520
GENE_A_16_P58263525	164	24	24	0.546
GENE_A_16_P21268289	158	27	27	0.526
GENE_A_18_P13898702	159	26	26	0.529
GENE_A_18_P13925082	147	32	32	0.489
GENE_A_18_P13899935	141	26	26	0.469
GENE_A_16_P21255471	167	32	33	0.555
GENE_A_16_P34774137	130	24	24	0.432
GENE_A_18_P22528457	142	23	24	0.472
GENE_A_16_P58291591	163	30	30	0.541
GENE_A_16_P58260592	146	25	26	0.485
GENE_A_16_P21208712	152	24	24	0.504
GENE_A_16_P21262792	147	22	22	0.488
GENE_A_16_P41406657	160	31	32	0.531
GENE_A_16_P41427222	161	29	29	0.534
GENE_A_16_P58300928	153	29	29	0.507
GENE_A_18_P13874230	153	27	28	0.507
GENE_A_16_P21239137	162	26	26	0.536
GENE_A_18_P13856062	147	27	28	0.487
GENE_A_16_P58270530	151	21	21	0.500
GENE_A_16_P21192749	164	30	31	0.543
GENE_A_16_P21270771	143	30	31	0.473
GENE_A_16_P58294164	139	26	26	0.460

GENE_A_16_P41407766	142	23	23	0.470
GENE_A_18_P22596174	135	26	26	0.446
GENE_A_16_P41439167	144	21	21	0.476
GENE_A_16_P21196607	170	29	29	0.561
GENE_A_16_P21244157	135	25	25	0.446
GENE_A_16_P03585696	145	28	28	0.479
GENE_A_16_P21255876	142	15	15	0.468
GENE_A_16_P21215884	164	27	27	0.541
GENE_A_18_P22499941	161	28	28	0.531
GENE_A_16_P03597218	151	25	25	0.498
GENE_A_16_P03577618	164	29	29	0.541
GENE_A_16_P03579906	156	23	23	0.514
GENE_A_16_P03593190	159	30	30	0.524
GENE_A_16_P58304924	160	28	28	0.527
GENE_A_16_P21229245	163	28	28	0.537
GENE_A_16_P21202292	140	18	18	0.461
GENE_A_16_P21217905	128	18	18	0.421
GENE_A_18_P13868336	134	23	24	0.441
GENE_A_16_P41437039	133	22	22	0.437
GENE_A_16_P58273080	125	21	21	0.411
GENE_A_18_P13916933	148	26	27	0.486
GENE_A_16_P03561027	145	26	27	0.477
GENE_A_16_P21223918	127	18	18	0.417
GENE_A_16_P21193807	127	25	26	0.417
GENE_A_16_P41428096	171	27	27	0.561
GENE_A_16_P03572780	150	24	24	0.492
GENE_A_18_P13858284	142	21	21	0.466
GENE_A_16_P58308123	139	26	26	0.456
GENE_A_16_P58309375	137	22	22	0.449
GENE_A_16_P03586675	144	29	29	0.472
GENE_A_16_P03583254	135	23	23	0.443
GENE_A_16_P03581527	154	24	24	0.505
GENE_A_16_P21215048	173	28	28	0.567
GENE_A_16_P58304912	172	28	28	0.563
GENE_A_16_P21224067	128	22	22	0.419
GENE_A_16_P58309247	157	25	25	0.514
GENE_A_16_P58294692	139	22	22	0.454
GENE_A_16_P21215087	155	28	28	0.506
GENE_A_16_P58296535	125	21	21	0.408
GENE_A_18_P22598081	166	26	26	0.542
GENE_A_16_P03583446	151	25	25	0.493
GENE_A_16_P21240233	165	34	34	0.538
GENE_A_16_P21196824	146	24	24	0.476
GENE_A_16_P58279667	154	24	24	0.502
GENE_A_18_P13938584	145	23	23	0.473
GENE_A_16_P21220627	135	23	23	0.440

GENE_A_18_P13908810	136	27	27	0.444
GENE_A_16_P58292688	153	29	29	0.499
GENE_A_16_P58261920	165	23	23	0.538
GENE_A_16_P03580259	155	25	25	0.505
GENE_A_18_P13890905	141	21	22	0.460
GENE_A_16_P58261261	153	18	18	0.498
GENE_A_18_P13879535	158	23	23	0.514
GENE_A_16_P03569065	155	19	19	0.505
GENE_A_16_P58267612	152	28	28	0.495
GENE_A_16_P58269140	169	24	24	0.550
GENE_A_16_P21198317	164	32	32	0.533
GENE_A_16_P21198343	135	22	22	0.439
GENE_A_16_P03580543	143	22	23	0.465
GENE_A_16_P41441291	166	23	23	0.540
GENE_A_16_P21199022	159	27	27	0.517
GENE_A_16_P21251912	145	22	22	0.471
GENE_A_18_P13895795	145	26	26	0.471
GENE_A_16_P58288474	157	26	26	0.510
GENE_A_16_P21223443	150	21	21	0.487
GENE_A_16_P41452059	138	22	22	0.448
GENE_A_16_P41433352	152	25	25	0.493
GENE_A_16_P03590277	157	34	34	0.510
GENE_A_16_P41404210	159	26	26	0.515
GENE_A_16_P41409163	157	25	25	0.509
GENE_A_16_P21202014	119	19	20	0.385
GENE_A_18_P13923334	178	27	27	0.576
GENE_A_16_P21210136	150	24	24	0.486
GENE_A_16_P34810023	140	22	22	0.453
GENE_A_16_P03577644	139	23	23	0.450
GENE_A_16_P58293847	150	24	25	0.485
GENE_A_16_P58293094	156	28	28	0.505
GENE_A_16_P21244663	161	27	28	0.521
GENE_A_16_P58292081	156	29	30	0.504
GENE_A_16_P03565395	148	24	25	0.478
GENE_A_16_P41398800	154	25	26	0.498
GENE_A_16_P21210001	179	24	24	0.578
GENE_A_16_P41413338	172	32	32	0.556
GENE_A_18_P22542396	154	26	27	0.497
GENE_A_16_P03566562	157	26	26	0.507
GENE_A_16_P21252635	147	23	23	0.474
GENE_A_16_P58269324	160	27	27	0.516
GENE_A_18_P22547761	144	23	25	0.464
GENE_A_16_P21205679	138	24	24	0.445
GENE_A_16_P58307282	158	25	25	0.509
GENE_A_16_P58295610	159	25	25	0.512
GENE_A_16_P03558501	143	23	23	0.460

GENE_A_16_P58296419	137	23	23	0.440
GENE_A_16_P58284589	127	21	21	0.408
GENE_A_16_P03570574	113	18	18	0.363
GENE_A_16_P58261844	154	22	22	0.494
GENE_A_18_P13881524	154	27	27	0.494
GENE_A_16_P03557568	149	22	23	0.478
GENE_A_18_P13927162	149	24	25	0.477
GENE_A_16_P58299315	143	22	22	0.458
GENE_A_18_P13913921	160	23	23	0.512
GENE_A_16_P58282736	167	24	25	0.534
GENE_A_18_P13912518	168	33	33	0.537
GENE_A_16_P21202023	161	26	26	0.515
GENE_A_16_P03586887	158	25	25	0.505
GENE_A_16_P58286190	146	22	22	0.466
GENE_A_16_P03576283	158	31	31	0.505
GENE_A_16_P03595852	181	28	28	0.578
GENE_A_16_P21231723	151	27	27	0.482
GENE_A_16_P58264804	143	20	21	0.456
GENE_A_16_P58265523	151	29	29	0.482
GENE_A_16_P21262183	139	20	20	0.443
GENE_A_16_P21268805	164	28	28	0.523
GENE_A_16_P58278379	137	21	22	0.437
GENE_A_16_P58286110	142	22	22	0.453
GENE_A_16_P58278259	155	26	26	0.494
GENE_A_16_P21206264	170	24	24	0.541
GENE_A_16_P58275375	147	26	26	0.468
GENE_A_16_P58273811	155	25	26	0.494
GENE_A_16_P03586147	157	19	19	0.500
GENE_A_16_P03594037	161	23	23	0.512
GENE_A_18_P22567521	168	27	27	0.535
GENE_A_16_P03588885	163	27	27	0.518
GENE_A_16_P58291427	151	24	24	0.480
GENE_A_18_P22562193	155	32	32	0.493
GENE_A_16_P21198232	165	27	27	0.524
GENE_A_16_P21248558	159	25	25	0.505
GENE_A_16_P21230286	132	24	24	0.419
GENE_A_16_P58309507	143	26	26	0.454
GENE_A_16_P21199947	153	25	25	0.485
GENE_A_16_P58272450	141	23	23	0.447
GENE_A_18_P22572447	138	24	24	0.438
GENE_A_18_P13898073	140	24	24	0.444
GENE_A_16_P41453995	149	24	24	0.472
GENE_A_16_P41424462	130	26	26	0.412
GENE_A_18_P13926746	157	20	20	0.497
GENE_A_16_P03595772	157	26	26	0.497
GENE_A_16_P21272433	148	27	27	0.468

GENE_A_16_P21203552	146	27	28	0.461
GENE_A_18_P13878037	143	22	22	0.452
GENE_A_16_P03585655	163	24	24	0.515
GENE_A_16_P41397302	161	30	30	0.508
GENE_A_16_P21239130	147	25	25	0.464
GENE_A_16_P03588331	155	25	25	0.489
GENE_A_16_P21227172	135	19	19	0.426
GENE_A_16_P58269220	158	27	27	0.498
GENE_A_16_P58272758	155	26	26	0.488
GENE_A_16_P21199853	130	20	20	0.409
GENE_A_18_P13907579	144	24	24	0.453
GENE_A_16_P41469248	143	23	23	0.450
GENE_A_18_P13891702	158	24	25	0.497
GENE_A_16_P58301206	165	22	22	0.519
GENE_A_16_P03597344	149	25	25	0.469
GENE_A_16_P21210703	158	26	26	0.497
GENE_A_16_P21204457	178	30	30	0.559
GENE_A_16_P21221633	167	25	25	0.525
GENE_A_16_P03566935	123	21	21	0.386
GENE_A_16_P03594485	157	19	19	0.493
GENE_A_16_P03553870	160	33	34	0.502
GENE_A_16_P58263801	160	25	25	0.502
GENE_A_16_P41396525	158	27	27	0.495
GENE_A_16_P21263786	159	25	27	0.498
GENE_A_16_P21206011	148	23	23	0.464
GENE_A_16_P58295843	159	31	31	0.498
GENE_A_16_P21229872	149	25	26	0.467
GENE_A_16_P21206103	148	22	22	0.464
GENE_A_18_P22572469	149	26	26	0.467
GENE_A_16_P21224567	169	27	28	0.529
GENE_A_16_P03561941	163	29	29	0.510
GENE_A_16_P03561928	166	30	30	0.519
GENE_A_16_P41402747	174	29	29	0.544
GENE_A_16_P58267790	174	26	26	0.544
GENE_A_16_P21251294	156	27	27	0.488
GENE_A_16_P58265877	160	22	22	0.500
GENE_A_16_P58289771	140	18	18	0.438
GENE_A_16_P58306237	156	21	21	0.488
GENE_A_16_P58298402	140	23	24	0.437
GENE_A_16_P21261709	154	20	20	0.481
GENE_A_18_P13894741	139	21	21	0.434
GENE_A_18_P13941274	159	24	24	0.496
GENE_A_18_P13907481	148	21	22	0.462
GENE_A_16_P21234463	148	24	25	0.462
GENE_A_16_P03558043	149	26	26	0.465
GENE_A_16_P21222413	149	21	21	0.465

GENE_A_16_P21244806	157	24	24	0.489
GENE_A_18_P13933964	160	26	26	0.499
GENE_A_16_P41400538	141	18	18	0.439
GENE_A_16_P41403655	149	25	25	0.463
GENE_A_16_P03568981	127	20	20	0.395
GENE_A_16_P41415935	171	25	25	0.532
GENE_A_16_P21233617	147	24	24	0.457
GENE_A_16_P21230169	132	21	22	0.410
GENE_A_16_P41458780	153	28	28	0.475
GENE_A_16_P41435050	134	19	19	0.416
GENE_A_16_P41427069	160	24	24	0.496
GENE_A_16_P03565790	133	23	23	0.412
GENE_A_16_P58274954	137	21	21	0.425
GENE_A_16_P03581783	150	24	24	0.465
GENE_A_18_P13935793	160	24	24	0.496
GENE_A_18_P22576670	164	24	25	0.508
GENE_A_18_P13899347	121	17	18	0.375
GENE_A_16_P21193834	154	25	26	0.477
GENE_A_16_P21252168	159	24	25	0.493
GENE_A_16_P41459589	145	22	22	0.449
GENE_A_16_P58287967	138	21	21	0.427
GENE_A_18_P13919923	162	25	26	0.502
GENE_A_16_P58288385	141	25	25	0.437
GENE_A_16_P21230976	152	25	26	0.471
GENE_A_16_P58278002	143	23	23	0.443
GENE_A_16_P03575422	145	22	23	0.449
GENE_A_16_P21200049	147	24	24	0.455
GENE_A_16_P03565816	119	27	28	0.368
GENE_A_18_P22486534	167	34	34	0.517
GENE_A_16_P58268460	153	22	22	0.473
GENE_A_16_P58299506	145	26	26	0.449
GENE_A_16_P03563307	153	26	26	0.473
GENE_A_16_P21261654	127	18	18	0.393
GENE_A_16_P21223892	138	19	19	0.426
GENE_A_16_P41455148	138	22	22	0.426
GENE_A_16_P58271911	152	25	26	0.470
GENE_A_16_P58273220	132	22	22	0.407
GENE_A_16_P03583523	144	25	26	0.444
GENE_A_16_P58280914	153	23	23	0.472
GENE_A_16_P21195014	157	25	25	0.484
GENE_A_18_P13910711	139	23	23	0.428
GENE_A_16_P21212145	153	26	27	0.471
GENE_A_16_P03580109	133	16	16	0.409
GENE_A_18_P13911885	147	27	27	0.452
GENE_A_16_P58282452	146	23	23	0.449
GENE_A_16_P03597270	149	24	24	0.458

GENE_A_16_P41400701	157	24	24	0.483
GENE_A_16_P58264117	157	24	24	0.483
GENE_A_16_P03584915	145	23	24	0.445
GENE_A_18_P13859369	171	27	27	0.525
GENE_A_16_P03551905	145	19	19	0.445
GENE_A_18_P13865723	137	19	20	0.421
GENE_A_16_P03580713	161	25	25	0.494
GENE_A_16_P41437807	153	25	25	0.470
GENE_A_16_P58263727	152	29	30	0.466
GENE_A_16_P41401709	139	20	20	0.426
GENE_A_16_P58309424	137	22	22	0.420
GENE_A_16_P21223908	154	20	20	0.472
GENE_A_16_P03558273	149	24	25	0.456
GENE_A_18_P13943866	141	24	24	0.432
GENE_A_16_P41428879	155	25	25	0.475
GENE_A_18_P13889048	177	24	24	0.542
GENE_A_16_P58307494	159	29	29	0.487
GENE_A_16_P41416533	154	26	26	0.471
GENE_A_16_P58293115	148	22	22	0.453
GENE_A_16_P41466226	141	22	22	0.431
GENE_A_16_P41453106	153	23	23	0.468
GENE_A_16_P03588138	174	31	31	0.532
GENE_A_16_P41418558	162	25	25	0.495
GENE_A_18_P13914365	139	19	20	0.425
GENE_A_18_P22595763	172	30	30	0.526
GENE_A_16_P58302687	162	25	26	0.495
GENE_A_16_P58267437	147	27	27	0.449
GENE_A_16_P21218462	132	21	21	0.403
GENE_A_16_P58273178	162	25	25	0.495
GENE_A_16_P03589021	135	20	21	0.412
GENE_A_16_P21233661	150	25	25	0.458
GENE_A_16_P03575720	145	23	24	0.442
GENE_A_18_P13869814	155	28	28	0.473
GENE_A_16_P58281105	147	21	21	0.448
GENE_A_16_P21214507	156	23	23	0.476
GENE_A_16_P58283396	154	27	27	0.469
GENE_A_16_P21253416	156	25	26	0.475
GENE_A_16_P41414600	154	22	22	0.469
GENE_A_16_P03585347	160	25	25	0.487
GENE_A_16_P21212577	164	28	28	0.499
GENE_A_16_P21233474	164	24	24	0.499
GENE_A_16_P21242570	166	16	16	0.505
GENE_A_18_P13874402	153	27	27	0.465
GENE_A_18_P13877361	163	29	29	0.495
GENE_A_16_P41458505	146	24	24	0.443
GENE_A_16_P03586674	163	23	23	0.495

GENE_A_16_P21227570	154	21	21	0.467
GENE_A_18_P22594426	158	23	23	0.479
GENE_A_16_P58267300	149	19	19	0.452
GENE_A_16_P03562593	164	27	27	0.497
GENE_A_16_P58288994	153	21	21	0.464
GENE_A_16_P41402592	158	23	24	0.479
GENE_A_16_P58264769	160	25	25	0.485
GENE_A_18_P13906353	154	24	24	0.466
GENE_A_16_P58265436	132	22	22	0.400
GENE_A_16_P21228204	142	21	21	0.430
GENE_A_16_P41456031	152	25	25	0.460
GENE_A_18_P13889958	148	22	22	0.448
GENE_A_16_P03583674	155	25	25	0.469
GENE_A_18_P13907378	150	24	24	0.454
GENE_A_18_P13906089	171	29	29	0.517
GENE_A_16_P21218492	147	24	24	0.444
GENE_A_16_P21251099	153	26	26	0.462
GENE_A_16_P21215036	130	18	18	0.393
GENE_A_16_P21192956	128	16	16	0.387
GENE_A_18_P22596119	151	24	24	0.456
GENE_A_16_P21258142	151	24	24	0.456
GENE_A_16_P41391300	156	26	27	0.471
GENE_A_16_P21247461	148	14	14	0.447
GENE_A_16_P41403112	145	19	19	0.437
GENE_A_16_P34790005	135	19	19	0.407
GENE_A_16_P03578350	145	24	24	0.437
GENE_A_16_P21203642	155	22	22	0.467
GENE_A_18_P22549757	148	20	20	0.446
GENE_A_16_P21195218	142	23	24	0.428
GENE_A_16_P21249424	172	27	27	0.518
GENE_A_16_P58265781	167	24	24	0.503
GENE_A_16_P58268909	172	22	22	0.518
GENE_A_18_P13882790	139	22	22	0.418
GENE_A_18_P13941527	138	21	21	0.415
GENE_A_16_P58282497	148	24	24	0.445
GENE_A_16_P41464715	155	22	23	0.466
GENE_A_16_P41416129	165	26	26	0.496
GENE_A_16_P41417211	133	17	17	0.400
GENE_A_16_P58288407	132	20	20	0.397
GENE_A_16_P03593430	154	25	25	0.463
GENE_A_16_P21232888	147	21	22	0.442
GENE_A_18_P13872724	147	23	23	0.442
GENE_A_16_P21258057	163	22	22	0.489
GENE_A_16_P58296364	153	22	22	0.459
GENE_A_16_P21219431	165	31	31	0.495
GENE_A_16_P21212554	160	24	24	0.480

GENE_A_18_P13885037	172	27	28	0.515
GENE_A_16_P21244103	146	22	22	0.437
GENE_A_16_P58274863	146	21	22	0.437
GENE_A_18_P13873408	154	25	25	0.461
GENE_A_16_P58278765	153	22	22	0.458
GENE_A_18_P13915838	138	16	16	0.413
GENE_A_16_P21202487	173	33	33	0.517
GENE_A_16_P34803463	144	18	19	0.430
GENE_A_16_P03596748	156	23	23	0.466
GENE_A_16_P21231512	148	21	22	0.442
GENE_A_16_P21215761	151	22	23	0.451
GENE_A_16_P34770226	191	27	27	0.570
GENE_A_16_P03597821	152	24	24	0.454
GENE_A_16_P21248733	167	23	23	0.499
GENE_A_16_P03571892	127	18	18	0.379
GENE_A_16_P58259872	125	21	22	0.373
GENE_A_16_P21226971	163	25	25	0.486
GENE_A_16_P03574478	177	26	26	0.528
GENE_A_18_P13881350	154	25	25	0.459
GENE_A_16_P03582844	147	20	20	0.438
GENE_A_16_P58302198	144	21	22	0.429
GENE_A_18_P13873849	174	29	29	0.518
GENE_A_16_P21233143	166	26	26	0.494
GENE_A_16_P03593299	116	14	14	0.345
GENE_A_16_P21198148	160	22	22	0.476
GENE_A_16_P41405679	142	22	22	0.422
GENE_A_16_P58303768	147	23	23	0.437
GENE_A_16_P03550677	153	32	33	0.454
GENE_A_16_P03572075	157	22	22	0.466
GENE_A_16_P58280151	157	24	24	0.466
GENE_A_18_P22516446	154	29	29	0.457
GENE_A_16_P58293753	137	20	21	0.407
GENE_A_16_P21253512	147	21	21	0.436
GENE_A_18_P13875766	164	27	28	0.486
GENE_A_16_P03556255	170	26	26	0.504
GENE_A_16_P03573338	164	27	28	0.486
GENE_A_16_P41415181	143	20	22	0.424
GENE_A_16_P58299690	151	23	23	0.447
GENE_A_16_P41395748	154	24	25	0.456
GENE_A_18_P13914347	140	26	26	0.414
GENE_A_16_P41417094	128	21	21	0.379
GENE_A_18_P13912914	144	21	21	0.426
GENE_A_18_P13868083	148	24	25	0.438
GENE_A_16_P41403349	149	22	22	0.440
GENE_A_16_P58259907	144	22	22	0.425
GENE_A_18_P13913617	161	24	25	0.476

GENE_A_16_P41412876	139	24	24	0.411
GENE_A_16_P21209942	158	24	24	0.467
GENE_A_16_P03597517	169	21	23	0.499
GENE_A_16_P58306354	142	22	22	0.419
GENE_A_16_P58278674	147	23	24	0.434
GENE_A_18_P13892259	164	24	24	0.484
GENE_A_16_P58275945	163	29	29	0.481
GENE_A_18_P22542084	163	23	23	0.480
GENE_A_18_P13903329	139	19	19	0.410
GENE_A_16_P58271074	166	25	25	0.489
GENE_A_16_P41413560	152	21	21	0.448
GENE_A_16_P58264116	154	22	22	0.454
GENE_A_16_P21212704	141	19	19	0.415
GENE_A_16_P41429618	146	20	20	0.430
GENE_A_16_P21231649	137	20	20	0.403
GENE_A_16_P03576831	164	30	30	0.482
GENE_A_16_P41398583	165	21	21	0.485
GENE_A_16_P58305298	152	22	22	0.447
GENE_A_16_P58307995	139	19	19	0.409
GENE_A_16_P03563140	135	22	22	0.397
GENE_A_16_P58282710	150	23	23	0.440
GENE_A_16_P21232057	154	21	21	0.452
GENE_A_16_P03597455	157	24	24	0.461
GENE_A_16_P21263945	148	19	19	0.434
GENE_A_16_P03592440	134	24	24	0.393
GENE_A_16_P41444343	127	17	17	0.372
GENE_A_16_P21259048	146	19	19	0.428
GENE_A_16_P03578589	159	24	25	0.466
GENE_A_16_P58268015	177	29	29	0.519
GENE_A_16_P03595204	145	18	18	0.425
GENE_A_16_P21196796	161	25	25	0.472
GENE_A_16_P03596599	169	22	23	0.495
GENE_A_16_P21202708	156	23	24	0.457
GENE_A_16_P58307100	157	21	23	0.460
GENE_A_16_P58305655	135	20	20	0.395
GENE_A_16_P21244253	147	24	24	0.430
GENE_A_16_P21247895	160	28	29	0.468
GENE_A_16_P41418768	170	26	26	0.497
GENE_A_16_P03594879	156	19	19	0.456
GENE_A_16_P41407451	144	20	21	0.421
GENE_A_16_P34798412	139	23	23	0.406
GENE_A_16_P03559871	143	25	25	0.418
GENE_A_16_P21227535	153	24	24	0.447
GENE_A_16_P58290017	151	16	16	0.441
GENE_A_18_P13868415	132	21	21	0.386
GENE_A_16_P58296002	151	22	22	0.441

GENE_A_16_P58290391	143	15	15	0.418
GENE_A_16_P21234323	148	23	23	0.432
GENE_A_16_P58265412	131	24	24	0.382
GENE_A_16_P58284763	146	20	20	0.426
GENE_A_16_P41446594	159	24	25	0.463
GENE_A_16_P58272780	145	20	20	0.423
GENE_A_16_P03597981	138	24	24	0.402
GENE_A_16_P58263848	159	26	26	0.463
GENE_A_16_P03589814	151	22	22	0.439
GENE_A_16_P21213891	142	16	16	0.413
GENE_A_16_P58297409	164	25	27	0.477
GENE_A_18_P22531294	173	26	26	0.503
GENE_A_16_P41388386	149	25	25	0.433
GENE_A_16_P03578557	161	26	26	0.467
GENE_A_16_P21263159	162	23	23	0.470
GENE_A_16_P21240842	133	19	20	0.386
GENE_A_16_P58281824	134	14	15	0.389
GENE_A_16_P58268145	152	24	24	0.441
GENE_A_16_P21213250	153	22	23	0.444
GENE_A_16_P21194031	135	22	23	0.391
GENE_A_18_P13879380	140	23	23	0.405
GENE_A_16_P21196795	149	22	22	0.431
GENE_A_18_P13922905	154	23	23	0.446
GENE_A_16_P21241883	154	26	26	0.445
GENE_A_16_P21222845	144	18	18	0.417
GENE_A_16_P21218172	159	22	22	0.460
GENE_A_16_P41412093	156	20	20	0.451
GENE_A_16_P03575272	166	25	26	0.480
GENE_A_16_P03575958	133	24	24	0.384
GENE_A_18_P13913824	141	23	23	0.408
GENE_A_16_P58277448	162	25	25	0.468
GENE_A_16_P03553224	141	20	20	0.407
GENE_A_16_P03585060	140	21	21	0.404
GENE_A_16_P58305107	164	25	25	0.474
GENE_A_16_P21254551	151	25	25	0.436
GENE_A_18_P13935671	162	24	24	0.468
GENE_A_16_P21210688	156	30	30	0.450
GENE_A_16_P41427483	166	25	25	0.479
GENE_A_18_P13900806	147	21	21	0.424
GENE_A_16_P21204725	137	21	22	0.395
GENE_A_16_P03572623	149	26	26	0.429
GENE_A_16_P03576630	141	23	23	0.406
GENE_A_16_P58289956	175	27	27	0.504
GENE_A_16_P21223635	152	19	20	0.438
GENE_A_16_P21233401	158	26	26	0.455
GENE_A_16_P03573802	161	28	28	0.464

GENE_A_16_P21214053	148	24	24	0.426
GENE_A_16_P21209159	164	25	25	0.472
GENE_A_16_P03575882	178	25	25	0.512
GENE_A_16_P03583493	156	21	21	0.449
GENE_A_16_P58285498	151	25	25	0.435
GENE_A_16_P03586984	146	20	23	0.420
GENE_A_16_P03565869	154	21	21	0.443
GENE_A_16_P21200210	138	20	20	0.397
GENE_A_16_P21203178	155	30	30	0.446
GENE_A_16_P21193997	154	26	26	0.443
GENE_A_16_P21197349	171	25	26	0.491
GENE_A_16_P58261722	173	28	28	0.497
GENE_A_16_P41437663	149	24	24	0.428
GENE_A_16_P03551984	171	24	24	0.491
GENE_A_16_P21222535	159	28	28	0.455
GENE_A_16_P21242419	137	20	20	0.392
GENE_A_18_P13943903	153	23	23	0.438
GENE_A_16_P21228458	165	21	21	0.472
GENE_A_16_P03571975	137	20	21	0.392
GENE_A_16_P21194292	156	24	24	0.446
GENE_A_16_P21219671	165	25	25	0.472
GENE_A_18_P22560387	165	22	22	0.472
GENE_A_18_P13889364	150	25	25	0.429
GENE_A_16_P03583770	152	23	23	0.435
GENE_A_16_P03592550	147	21	21	0.420
GENE_A_16_P58285180	129	21	21	0.369
GENE_A_16_P58263988	160	23	23	0.457
GENE_A_16_P03591656	189	25	25	0.540
GENE_A_16_P58308586	144	21	22	0.411
GENE_A_16_P58264652	155	25	25	0.443
GENE_A_14_P118035	167	24	24	0.477
GENE_A_16_P21236827	148	20	22	0.422
GENE_A_16_P41470124	175	30	31	0.499
GENE_A_16_P58265256	165	25	26	0.471
GENE_A_16_P41446137	144	22	22	0.411
GENE_A_18_P13864506	138	20	21	0.394
GENE_A_16_P03572503	149	22	23	0.425
GENE_A_16_P58297956	155	25	25	0.442
GENE_A_16_P34795183	164	20	21	0.467
GENE_A_16_P58307731	146	23	23	0.416
GENE_A_16_P21244959	169	25	25	0.481
GENE_A_16_P58292231	126	20	21	0.359
GENE_A_16_P03555791	176	24	24	0.501
GENE_A_16_P03596459	166	23	23	0.473
GENE_A_16_P21242412	145	20	21	0.413
GENE_A_16_P58282572	146	19	19	0.416

GENE_A_18_P13874241	157	24	25	0.447
GENE_A_16_P03594432	154	21	21	0.438
GENE_A_16_P03596978	148	23	23	0.421
GENE_A_18_P13895960	136	22	22	0.387
GENE_A_16_P21256513	157	21	21	0.446
GENE_A_16_P58294686	148	17	17	0.420
GENE_A_16_P58284916	145	20	20	0.412
GENE_A_16_P58266888	137	17	17	0.389
GENE_A_16_P41405998	169	23	23	0.480
GENE_A_16_P58266760	169	24	24	0.479
GENE_A_16_P58308897	145	23	23	0.411
GENE_A_16_P41419253	168	21	21	0.476
GENE_A_16_P41447179	152	20	20	0.431
GENE_A_16_P03589830	139	18	20	0.394
GENE_A_16_P58293223	170	20	20	0.482
GENE_A_18_P13926029	149	22	23	0.422
GENE_A_16_P58274848	145	20	21	0.411
GENE_A_16_P58300449	163	23	23	0.461
GENE_A_16_P21193896	147	22	22	0.416
GENE_A_16_P41432723	148	23	23	0.419
GENE_A_16_P03585155	150	19	19	0.425
GENE_A_18_P13940936	161	24	24	0.456
GENE_A_18_P13883753	134	21	21	0.379
GENE_A_16_P58262416	156	21	21	0.441
GENE_A_16_P21220009	158	23	23	0.447
GENE_A_16_P21240794	156	20	20	0.441
GENE_A_16_P58265406	166	23	23	0.469
GENE_A_16_P41431314	157	22	22	0.444
GENE_A_16_P03593075	159	25	26	0.449
GENE_A_18_P13873685	144	20	20	0.407
GENE_A_16_P58294001	138	19	19	0.390
GENE_A_16_P21199672	165	25	25	0.466
GENE_A_16_P21220786	164	25	25	0.463
GENE_A_16_P41463146	161	15	15	0.454
GENE_A_16_P58262790	168	28	29	0.474
GENE_A_16_P21253912	167	19	19	0.471
GENE_A_16_P21260734	150	23	23	0.423
GENE_A_16_P41439379	186	30	30	0.524
GENE_A_16_P21198338	155	30	30	0.437
GENE_A_16_P58295727	131	23	23	0.369
GENE_A_16_P03590974	177	25	25	0.499
GENE_A_16_P58290385	136	20	20	0.383
GENE_A_16_P03589097	162	22	22	0.456
GENE_A_16_P03575951	160	19	20	0.450
GENE_A_16_P58300748	160	23	23	0.450
GENE_A_16_P58309657	162	23	23	0.456

GENE_A_16_P58278769	154	24	24	0.433
GENE_A_16_P21262441	142	22	22	0.399
GENE_A_16_P21204740	142	20	20	0.399
GENE_A_18_P13880870	161	22	22	0.452
GENE_A_18_P13888523	154	22	22	0.433
GENE_A_16_P58306805	119	21	22	0.334
GENE_A_16_P58305582	150	21	21	0.421
GENE_A_16_P21197899	136	18	18	0.382
GENE_A_16_P41457992	138	22	22	0.388
GENE_A_16_P03566071	149	19	19	0.418
GENE_A_16_P58270901	153	22	22	0.430
GENE_A_16_P41397875	168	28	28	0.472
GENE_A_16_P03590768	134	17	17	0.376
GENE_A_16_P41446811	120	19	19	0.336
GENE_A_16_P21268833	134	15	15	0.376
GENE_A_16_P03586986	135	19	19	0.378
GENE_A_16_P03567399	146	20	20	0.409
GENE_A_16_P03576749	156	20	20	0.437
GENE_A_18_P22504070	148	23	23	0.415
GENE_A_18_P13912994	178	28	28	0.499
GENE_A_16_P41401488	158	19	19	0.442
GENE_A_16_P21247239	151	25	25	0.423
GENE_A_16_P21199955	161	24	24	0.451
GENE_A_16_P58308636	134	20	20	0.375
GENE_A_16_P58300866	143	23	23	0.400
GENE_A_16_P58263737	152	20	20	0.425
GENE_A_16_P21212802	150	25	26	0.419
GENE_A_16_P21258832	133	20	20	0.372
GENE_A_16_P41411001	134	20	20	0.375
GENE_A_16_P41438283	132	20	20	0.369
GENE_A_16_P41425026	146	18	18	0.408
GENE_A_16_P41427059	156	23	23	0.436
GENE_A_16_P58285997	134	19	19	0.374
GENE_A_16_P03581149	165	23	23	0.460
GENE_A_16_P58272925	139	20	20	0.388
GENE_A_16_P21211169	136	20	20	0.379
GENE_A_16_P21258435	169	23	23	0.471
GENE_A_16_P41445638	158	22	22	0.441
GENE_A_16_P21254874	137	20	20	0.382
GENE_A_16_P03572613	170	26	26	0.474
GENE_A_18_P13868365	145	25	25	0.404
GENE_A_16_P21202218	163	22	23	0.454
GENE_A_16_P41430234	142	18	18	0.396
GENE_A_18_P13935484	174	21	21	0.485
GENE_A_16_P41424805	143	19	19	0.398
GENE_A_16_P03564560	140	19	19	0.390

GENE_A_16_P41415911	147	20	20	0.409
GENE_A_16_P58299102	142	18	18	0.395
GENE_A_16_P58279377	147	20	21	0.409
GENE_A_16_P21204581	147	21	21	0.409
GENE_A_16_P41449684	153	23	23	0.425
GENE_A_16_P21250783	159	21	22	0.442
GENE_A_16_P58287467	148	23	23	0.411
GENE_A_16_P58270314	142	19	19	0.395
GENE_A_16_P58274271	144	20	20	0.400
GENE_A_16_P41436637	147	20	20	0.408
GENE_A_16_P21239094	160	25	25	0.444
GENE_A_16_P58294388	153	19	19	0.425
GENE_A_16_P03595340	131	20	22	0.364
GENE_A_16_P58305286	135	19	19	0.375
GENE_A_16_P58269255	142	23	23	0.394
GENE_A_16_P58302275	174	26	27	0.483
GENE_A_18_P13869311	131	18	18	0.363
GENE_A_18_P22582368	149	24	24	0.413
GENE_A_16_P21202234	151	22	22	0.418
GENE_A_18_P13888708	162	23	23	0.449
GENE_A_16_P21203228	148	22	22	0.410
GENE_A_16_P58294052	159	29	29	0.440
GENE_A_16_P21213356	135	18	18	0.374
GENE_A_16_P03575921	140	19	20	0.387
GENE_A_16_P41421278	159	21	22	0.440
GENE_A_16_P58278964	149	25	26	0.412
GENE_A_16_P03577897	161	17	17	0.445
GENE_A_16_P21217742	132	18	18	0.365
GENE_A_16_P21223919	148	21	21	0.409
GENE_A_16_P21213871	128	18	18	0.354
GENE_A_16_P58272280	150	24	24	0.415
GENE_A_18_P13938905	144	20	20	0.398
GENE_A_16_P21206053	155	23	25	0.428
GENE_A_18_P13903612	139	20	20	0.384
GENE_A_16_P03575182	145	21	21	0.401
GENE_A_16_P58290354	142	23	23	0.392
GENE_A_18_P22541156	143	19	21	0.395
GENE_A_16_P21231823	154	23	23	0.425
GENE_A_18_P22561041	150	17	17	0.414
GENE_A_16_P03554879	154	22	22	0.425
GENE_A_16_P03564763	153	25	25	0.422
GENE_A_16_P58291754	168	18	18	0.463
GENE_A_18_P22495431	129	18	18	0.356
GENE_A_18_P13927550	132	19	19	0.364
GENE_A_16_P41407782	166	21	21	0.457
GENE_A_18_P13870197	160	22	22	0.441

GENE_A_16_P41398335	138	18	18	0.380
GENE_A_16_P41425762	132	21	21	0.364
GENE_A_16_P21267516	168	23	23	0.462
GENE_A_16_P58291796	138	18	18	0.380
GENE_A_16_P58278106	144	20	20	0.396
GENE_A_16_P58298262	175	25	25	0.482
GENE_A_16_P58283961	155	23	24	0.426
GENE_A_18_P13852245	159	23	23	0.437
GENE_A_16_P21213299	162	26	26	0.445
GENE_A_16_P41413718	155	25	25	0.426
GENE_A_16_P58270509	141	20	20	0.388
GENE_A_16_P21204599	126	16	16	0.346
GENE_A_16_P03582295	157	25	27	0.431
GENE_A_18_P13879257	160	25	25	0.440
GENE_A_16_P21254447	139	21	21	0.382
GENE_A_16_P21211400	161	24	25	0.442
GENE_A_16_P03559557	146	22	22	0.401
GENE_A_18_P13883065	149	23	23	0.409
GENE_A_18_P13884129	126	19	19	0.346
GENE_A_16_P58297571	169	26	26	0.464
GENE_A_16_P58291397	149	20	21	0.409
GENE_A_16_P58259568	153	23	24	0.419
GENE_A_18_P13891636	138	19	19	0.378
GENE_A_16_P41412397	152	21	21	0.416
GENE_A_18_P13893549	167	24	24	0.458
GENE_A_16_P58273301	145	23	23	0.397
GENE_A_16_P41414925	156	21	22	0.427
GENE_A_16_P58281222	120	17	17	0.329
GENE_A_16_P21236547	133	20	20	0.364
GENE_A_16_P41439341	158	23	23	0.432
GENE_A_16_P41449300	154	22	22	0.421
GENE_A_16_P41429870	163	26	26	0.446
GENE_A_16_P03585845	153	20	21	0.418
GENE_A_16_P03587396	166	21	21	0.454
GENE_A_16_P21193603	147	20	21	0.402
GENE_A_16_P58303621	147	23	23	0.402
GENE_A_16_P41391128	180	26	26	0.491
GENE_A_16_P58275156	151	21	21	0.412
GENE_A_18_P13946179	167	26	26	0.456
GENE_A_16_P03554196	168	24	24	0.458
GENE_A_16_P21230922	148	18	18	0.404
GENE_A_16_P03554344	123	18	18	0.335
GENE_A_16_P21198413	175	21	22	0.477
GENE_A_16_P21216127	161	21	21	0.439
GENE_A_16_P41391454	151	21	21	0.412
GENE_A_16_P03595115	167	19	20	0.455

GENE_A_16_P21237209	144	16	16	0.392
GENE_A_16_P03577974	163	22	22	0.444
GENE_A_16_P58302842	168	24	24	0.458
GENE_A_16_P58276367	121	16	16	0.329
GENE_A_16_P21222616	161	23	23	0.438
GENE_A_16_P58277674	150	18	18	0.408
GENE_A_16_P21191052	158	26	26	0.430
GENE_A_16_P21215348	173	19	19	0.471
GENE_A_16_P58280450	168	25	25	0.457
GENE_A_16_P21259755	140	20	20	0.381
GENE_A_16_P03578730	150	18	18	0.408
GENE_A_16_P03566488	160	23	23	0.435
GENE_A_16_P03591652	157	22	22	0.427
GENE_A_16_P03595561	160	16	16	0.435
GENE_A_16_P21196144	145	20	20	0.394
GENE_A_16_P21244427	134	18	18	0.364
GENE_A_16_P58274555	143	18	18	0.389
GENE_A_18_P13886359	147	21	21	0.399
GENE_A_16_P41458099	168	24	24	0.456
GENE_A_18_P13924079	146	21	21	0.397
GENE_A_18_P22560875	170	25	25	0.462
GENE_A_16_P21271152	161	21	21	0.437
GENE_A_16_P21208438	139	18	19	0.377
GENE_A_18_P13893232	170	20	20	0.462
GENE_A_16_P21222047	148	23	23	0.402
GENE_A_16_P58270295	143	19	19	0.388
GENE_A_16_P21196770	136	22	22	0.369
GENE_A_16_P21214857	159	23	23	0.431
GENE_A_16_P03597379	145	23	23	0.393
GENE_A_16_P41461392	134	20	20	0.363
GENE_A_16_P03552860	159	20	20	0.431
GENE_A_16_P03587959	136	17	18	0.369
GENE_A_16_P21248140	147	20	21	0.398
GENE_A_16_P21196358	153	23	23	0.415
GENE_A_16_P58275250	131	18	18	0.355
GENE_A_16_P21260603	145	23	23	0.393
GENE_A_16_P03574560	168	21	21	0.455
GENE_A_16_P58305357	159	22	23	0.431
GENE_A_16_P03586724	154	19	19	0.417
GENE_A_16_P58300266	138	17	17	0.374
GENE_A_16_P21256143	139	20	20	0.376
GENE_A_16_P58270001	162	20	21	0.438
GENE_A_16_P03583595	155	25	26	0.419
GENE_A_18_P13926791	165	28	28	0.446
GENE_A_16_P03580881	155	23	23	0.419
GENE_A_16_P21243725	178	28	28	0.481

GENE_A_16_P03585093	170	24	24	0.460
GENE_A_16_P58286941	148	20	20	0.400
GENE_A_14_P116756	139	19	19	0.376
GENE_A_16_P21244480	173	24	24	0.468
GENE_A_18_P22596734	156	24	24	0.422
GENE_A_16_P58276110	155	23	23	0.419
GENE_A_16_P21258552	151	24	24	0.408
GENE_A_16_P41391399	166	25	25	0.449
GENE_A_16_P21205661	152	21	21	0.411
GENE_A_18_P13909872	146	21	21	0.394
GENE_A_16_P58274186	169	25	25	0.456
GENE_A_16_P03580699	151	19	19	0.407
GENE_A_16_P21202806	141	20	20	0.381
GENE_A_16_P21254583	172	21	21	0.464
GENE_A_16_P58283542	161	24	24	0.434
GENE_A_16_P58272822	143	19	19	0.386
GENE_A_16_P58309662	142	22	22	0.383
GENE_A_16_P58286598	159	20	21	0.429
GENE_A_16_P03578214	152	23	23	0.410
GENE_A_18_P22568826	161	19	19	0.434
GENE_A_16_P03582109	142	20	20	0.383
GENE_A_16_P58285847	165	23	23	0.445
GENE_A_16_P58308951	162	20	20	0.437
GENE_A_16_P58289989	147	21	21	0.396
GENE_A_16_P21206058	163	22	22	0.439
GENE_A_16_P03553369	163	25	26	0.439
GENE_A_16_P41450319	153	14	14	0.412
GENE_A_16_P03570059	147	18	18	0.396
GENE_A_16_P58288587	157	22	22	0.422
GENE_A_16_P03586749	162	23	26	0.436
GENE_A_16_P21196312	127	15	18	0.341
GENE_A_16_P03596866	152	21	21	0.409
GENE_A_16_P34820638	132	19	19	0.355
GENE_A_16_P58271239	128	19	19	0.344
GENE_A_16_P58297094	146	17	17	0.392
GENE_A_16_P03582678	150	24	24	0.403
GENE_A_18_P13883199	151	22	22	0.406
GENE_A_16_P41454929	163	25	25	0.438
GENE_A_16_P41429156	149	21	21	0.400
GENE_A_16_P03576925	162	26	26	0.435
GENE_A_16_P03581209	147	23	23	0.395
GENE_A_16_P34768499	161	23	24	0.432
GENE_A_16_P58277331	168	23	24	0.451
GENE_A_16_P58275080	152	23	24	0.408
GENE_A_16_P58281501	146	18	18	0.392
GENE_A_16_P58265443	160	22	22	0.429

GENE_A_16_P03591426	163	21	21	0.437
GENE_A_16_P21251434	146	18	18	0.391
GENE_A_16_P21251535	144	20	21	0.386
GENE_A_16_P58273957	157	24	24	0.420
GENE_A_18_P13944666	142	20	20	0.380
GENE_A_16_P41441651	137	16	16	0.367
GENE_A_16_P41440963	144	22	22	0.385
GENE_A_16_P21212981	152	16	17	0.407
GENE_A_16_P03594555	140	22	22	0.375
GENE_A_16_P58271382	170	23	23	0.455
GENE_A_18_P13880535	156	19	19	0.417
GENE_A_16_P41426429	145	22	22	0.388
GENE_A_16_P21269363	156	25	25	0.417
GENE_A_16_P21232642	131	16	16	0.350
GENE_A_16_P03555200	150	24	25	0.401
GENE_A_16_P21202552	148	22	22	0.396
GENE_A_16_P41431207	151	24	24	0.403
GENE_A_16_P03578687	152	23	23	0.406
GENE_A_18_P13873632	160	24	24	0.427
GENE_A_16_P21249093	156	23	24	0.417
GENE_A_18_P13904389	163	24	24	0.435
GENE_A_16_P58300451	149	21	21	0.398
GENE_A_16_P03576693	174	26	26	0.464
GENE_A_16_P21262885	137	14	14	0.366
GENE_A_16_P58265603	144	17	17	0.384
GENE_A_18_P22560556	135	19	19	0.360
GENE_A_16_P41423787	159	17	17	0.424
GENE_A_18_P22517109	141	21	21	0.376
GENE_A_18_P22585026	169	25	26	0.451
GENE_A_18_P13861366	155	24	24	0.413
GENE_A_16_P58279317	138	16	17	0.368
GENE_A_16_P58307535	153	15	15	0.408
GENE_A_16_P21216259	120	15	15	0.320
GENE_A_16_P21215796	124	17	17	0.330
GENE_A_16_P34781085	146	20	20	0.389
GENE_A_16_P21230564	128	18	18	0.341
GENE_A_18_P13881841	151	21	21	0.402
GENE_A_16_P58279196	153	21	21	0.407
GENE_A_16_P03582466	140	16	16	0.373
GENE_A_16_P03578552	144	20	20	0.383
GENE_A_16_P58281711	168	21	21	0.447
GENE_A_16_P03564441	131	22	22	0.349
GENE_A_16_P58273243	150	22	22	0.399
GENE_A_16_P41427686	137	19	19	0.365
GENE_A_16_P21268979	158	21	21	0.420
GENE_A_16_P58296881	154	13	13	0.410

GENE_A_16_P58288195	142	19	19	0.378
GENE_A_16_P58287243	145	18	18	0.386
GENE_A_16_P58275775	137	18	18	0.364
GENE_A_16_P58274065	133	18	18	0.354
GENE_A_16_P58307722	166	20	20	0.441
GENE_A_16_P58281975	156	19	20	0.415
GENE_A_16_P21210400	152	21	22	0.404
GENE_A_16_P58289066	147	21	21	0.391
GENE_A_16_P58282918	146	23	23	0.388
GENE_A_18_P22551217	135	17	17	0.359
GENE_A_16_P03584520	154	23	23	0.409
GENE_A_16_P58307098	143	20	21	0.380
GENE_A_16_P21224717	157	22	22	0.417
GENE_A_16_P03580664	167	25	25	0.443
GENE_A_16_P21265097	151	22	22	0.401
GENE_A_16_P21261797	159	16	17	0.422
GENE_A_16_P03580238	163	22	24	0.433
GENE_A_18_P13922744	166	21	21	0.441
GENE_A_18_P13918653	147	23	23	0.390
GENE_A_16_P41440717	144	18	18	0.382
GENE_A_16_P21203590	147	15	16	0.390
GENE_A_16_P03578289	143	21	21	0.379
GENE_A_16_P03578600	146	20	20	0.387
GENE_A_16_P03555444	148	19	19	0.392
GENE_A_16_P41416384	163	27	27	0.432
GENE_A_16_P58272487	188	31	31	0.498
GENE_A_16_P03576077	147	22	22	0.389
GENE_A_16_P21250796	155	20	20	0.410
GENE_A_16_P21238821	134	19	19	0.355
GENE_A_16_P58281760	155	21	21	0.410
GENE_A_16_P58281332	155	20	20	0.410
GENE_A_16_P03583226	138	22	22	0.365
GENE_A_18_P22605914	125	17	18	0.331
GENE_A_16_P03572429	151	18	19	0.399
GENE_A_16_P21197362	153	19	19	0.405
GENE_A_16_P58269631	149	20	20	0.394
GENE_A_18_P13886776	164	22	22	0.433
GENE_A_16_P03566806	170	20	20	0.449
GENE_A_16_P58272019	176	20	21	0.465
GENE_A_16_P03587565	147	19	19	0.388
GENE_A_16_P41467325	135	19	19	0.357
GENE_A_16_P21224075	136	16	16	0.359
GENE_A_16_P41415657	134	20	20	0.354
GENE_A_16_P41396468	162	20	20	0.428
GENE_A_18_P13892343	158	21	21	0.417
GENE_A_16_P34809936	154	26	26	0.407

GENE_A_16_P41426199	151	16	16	0.398
GENE_A_18_P22526973	153	24	24	0.404
GENE_A_16_P58297522	148	13	13	0.390
GENE_A_18_P13933707	167	25	25	0.440
GENE_A_16_P21209002	150	23	23	0.396
GENE_A_16_P21205468	154	19	19	0.406
GENE_A_16_P41437267	157	22	22	0.414
GENE_A_16_P21265434	163	22	22	0.429
GENE_A_16_P58270146	139	17	17	0.366
GENE_A_18_P22568443	152	20	20	0.400
GENE_A_16_P58273942	159	20	20	0.419
GENE_A_16_P21256406	162	19	19	0.427
GENE_A_16_P41452124	154	24	24	0.405
GENE_A_16_P21245280	146	22	22	0.384
GENE_A_16_P58288886	162	22	25	0.426
GENE_A_18_P13947900	131	18	18	0.344
GENE_A_16_P58269384	158	18	18	0.415
GENE_A_16_P03589123	167	21	21	0.439
GENE_A_16_P03573712	157	27	27	0.412
GENE_A_16_P58294844	135	22	22	0.355
GENE_A_16_P58307012	165	23	24	0.433
GENE_A_18_P22545857	154	20	20	0.404
GENE_A_18_P13924130	131	19	19	0.344
GENE_A_16_P03595918	143	20	20	0.375
GENE_A_16_P03584097	162	25	25	0.425
GENE_A_18_P13893407	162	23	23	0.425
GENE_A_16_P21208740	149	17	17	0.391
GENE_A_16_P21259402	145	18	18	0.380
GENE_A_16_P21219811	168	21	21	0.440
GENE_A_16_P21219513	151	19	19	0.395
GENE_A_16_P41398406	149	20	21	0.390
GENE_A_18_P13888478	149	20	20	0.390
GENE_A_16_P58269063	159	25	26	0.416
GENE_A_16_P41411619	157	20	21	0.410
GENE_A_16_P03552072	155	26	26	0.405
GENE_A_16_P03556629	148	23	23	0.387
GENE_A_16_P41438780	155	21	21	0.405
GENE_A_16_P21255966	168	23	23	0.439
GENE_A_16_P58292229	171	25	26	0.446
GENE_A_18_P13871196	152	20	20	0.397
GENE_A_16_P03583303	139	21	21	0.363
GENE_A_16_P03580319	151	23	23	0.394
GENE_A_16_P21240020	173	18	18	0.451
GENE_A_16_P58280995	135	18	18	0.352
GENE_A_16_P58300003	141	19	19	0.368
GENE_A_16_P03588752	149	23	23	0.388

GENE_A_16_P03579962	152	22	22	0.396
GENE_A_16_P21206150	140	17	17	0.365
GENE_A_16_P21196382	158	22	22	0.412
GENE_A_16_P21204874	169	21	21	0.440
GENE_A_16_P21252748	152	14	14	0.396
GENE_A_16_P58300837	150	21	21	0.391
GENE_A_16_P58284076	171	27	27	0.445
GENE_A_14_P133103	151	18	20	0.393
GENE_A_16_P41390296	139	21	21	0.362
GENE_A_16_P21261692	151	21	21	0.393
GENE_A_18_P13923053	151	17	17	0.393
GENE_A_16_P21216635	167	23	23	0.434
GENE_A_16_P21255691	134	18	19	0.348
GENE_A_18_P13868391	138	14	14	0.359
GENE_A_16_P58265362	145	19	19	0.377
GENE_A_16_P58305701	146	19	19	0.379
GENE_A_16_P21237608	158	19	19	0.410
GENE_A_16_P21204222	169	26	26	0.439
GENE_A_16_P03571183	157	22	22	0.407
GENE_A_16_P41415626	151	22	22	0.392
GENE_A_18_P13911534	143	18	19	0.371
GENE_A_16_P41435207	164	23	23	0.425
GENE_A_16_P58270873	180	24	24	0.467
GENE_A_16_P58298891	142	15	15	0.368
GENE_A_16_P03551140	150	17	17	0.389
GENE_A_18_P22484284	146	19	20	0.378
GENE_A_16_P03581029	150	18	18	0.389
GENE_A_16_P58270534	164	24	26	0.425
GENE_A_16_P21206442	160	22	22	0.415
GENE_A_16_P41398265	172	23	23	0.446
GENE_A_16_P58266040	148	19	19	0.383
GENE_A_16_P58300406	159	23	23	0.412
GENE_A_16_P21197849	142	22	23	0.367
GENE_A_16_P21232142	124	15	15	0.320
GENE_A_16_P41443086	164	23	24	0.423
GENE_A_18_P13870886	142	19	19	0.367
GENE_A_16_P21222988	157	18	18	0.405
GENE_A_16_P41446166	134	17	17	0.346
GENE_A_18_P13933103	140	19	19	0.361
GENE_A_16_P58294533	149	17	17	0.384
GENE_A_16_P58302123	158	22	22	0.407
GENE_A_18_P13865410	151	16	16	0.389
GENE_A_16_P03557696	140	19	19	0.361
GENE_A_18_P13869481	147	22	22	0.379
GENE_A_16_P03577061	175	22	22	0.451
GENE_A_16_P58297811	169	23	23	0.435

GENE_A_16_P21230217	152	22	22	0.392
GENE_A_16_P58281492	151	25	25	0.389
GENE_A_18_P13874291	151	16	16	0.389
GENE_A_18_P13879455	141	18	18	0.363
GENE_A_16_P58285410	138	17	17	0.355
GENE_A_16_P03588693	151	20	20	0.388
GENE_A_16_P58299071	140	22	22	0.360
GENE_A_16_P58266630	151	19	20	0.388
GENE_A_16_P03564246	153	24	24	0.393
GENE_A_16_P58296910	164	23	23	0.422
GENE_A_16_P21224855	143	19	19	0.368
GENE_A_16_P58277533	147	15	16	0.378
GENE_A_16_P03583428	140	19	19	0.360
GENE_A_16_P58264696	154	18	18	0.396
GENE_A_16_P21202964	182	27	27	0.468
GENE_A_16_P21270487	161	17	17	0.414
GENE_A_16_P58302534	143	20	20	0.367
GENE_A_16_P21223749	154	23	24	0.395
GENE_A_16_P58290577	152	20	20	0.390
GENE_A_16_P58293406	157	19	19	0.403
GENE_A_16_P58292611	161	24	24	0.413
GENE_A_16_P58259923	147	19	19	0.377
GENE_A_18_P13877044	154	20	20	0.395
GENE_A_16_P58303278	167	22	22	0.429
GENE_A_18_P13875775	157	25	25	0.403
GENE_A_18_P13881416	144	19	20	0.369
GENE_A_16_P21215013	155	22	22	0.397
GENE_A_16_P58285230	158	18	19	0.405
GENE_A_16_P58260904	141	16	16	0.362
GENE_A_16_P21232291	161	21	21	0.413
GENE_A_16_P58268726	150	17	17	0.384
GENE_A_16_P03585964	129	17	17	0.331
GENE_A_16_P58269450	162	23	23	0.415
GENE_A_16_P03556413	137	15	15	0.351
GENE_A_16_P58260793	149	21	21	0.382
GENE_A_16_P58270542	152	15	15	0.389
GENE_A_18_P13886201	154	19	19	0.394
GENE_A_16_P58300368	143	17	17	0.366
GENE_A_16_P03572230	143	17	17	0.366
GENE_A_16_P58277825	157	21	21	0.402
GENE_A_18_P13892407	147	20	20	0.376
GENE_A_16_P03563678	161	19	20	0.412
GENE_A_16_P03573242	152	18	18	0.389
GENE_A_16_P58272386	149	20	20	0.381
GENE_A_16_P03596338	158	17	17	0.404
GENE_A_16_P21206437	136	18	18	0.348

GENE_A_16_P41442870	160	20	20	0.409
GENE_A_16_P03597328	160	22	22	0.409
GENE_A_16_P41413622	128	17	17	0.327
GENE_A_16_P34807386	141	19	19	0.361
GENE_A_16_P21251750	147	23	23	0.376
GENE_A_16_P21205654	139	17	17	0.355
GENE_A_16_P21212258	148	18	18	0.378
GENE_A_16_P21253347	141	17	17	0.360
GENE_A_16_P21224139	142	19	21	0.363
GENE_A_16_P03576453	156	23	23	0.399
GENE_A_16_P03576592	170	23	23	0.434
GENE_A_18_P13889309	152	18	20	0.388
GENE_A_16_P41424263	170	22	22	0.434
GENE_A_16_P03591916	130	19	19	0.332
GENE_A_16_P41432049	138	16	16	0.352
GENE_A_16_P21254192	133	19	19	0.339
GENE_A_18_P22561105	146	17	17	0.372
GENE_A_16_P03579670	156	24	24	0.398
GENE_A_16_P21205414	145	15	15	0.369
GENE_A_16_P34812841	167	22	22	0.425
GENE_A_16_P21217107	162	21	21	0.413
GENE_A_16_P21195599	135	17	17	0.344
GENE_A_16_P21193790	151	18	18	0.385
GENE_A_18_P22553526	137	17	17	0.349
GENE_A_16_P21215727	151	17	18	0.384
GENE_A_16_P21254951	148	18	19	0.377
GENE_A_16_P03570639	145	21	21	0.369
GENE_A_18_P22591147	152	19	19	0.387
GENE_A_16_P03595527	151	20	21	0.384
GENE_A_16_P03593080	139	20	20	0.354
GENE_A_16_P41401033	177	24	24	0.450
GENE_A_16_P58293880	140	20	20	0.356
GENE_A_18_P13909554	143	22	23	0.364
GENE_A_18_P22493591	160	16	16	0.407
GENE_A_16_P03588120	118	18	18	0.300
GENE_A_16_P58289492	162	24	24	0.412
GENE_A_16_P41412822	150	19	19	0.381
GENE_A_18_P22504589	147	21	21	0.373
GENE_A_16_P41400269	154	17	17	0.391
GENE_A_16_P34767287	152	19	20	0.386
GENE_A_16_P41409964	143	19	19	0.363
GENE_A_16_P58291907	160	20	20	0.406
GENE_A_16_P21234847	152	18	19	0.386
GENE_A_18_P13897221	149	19	19	0.378
GENE_A_18_P13868253	157	22	22	0.398
GENE_A_16_P41440680	158	23	23	0.401

GENE_A_16_P58270121	152	20	20	0.386
GENE_A_16_P58262904	173	26	26	0.439
GENE_A_16_P58265674	150	22	22	0.380
GENE_A_16_P03570914	131	23	24	0.332
GENE_A_18_P22559707	151	19	19	0.383
GENE_A_16_P58296597	155	20	20	0.393
GENE_A_16_P58273233	151	17	18	0.383
GENE_A_16_P58260545	146	19	19	0.370
GENE_A_16_P41392117	160	22	23	0.405
GENE_A_16_P41399335	149	21	21	0.377
GENE_A_16_P21214698	153	19	19	0.387
GENE_A_16_P58279220	152	20	20	0.385
GENE_A_16_P21252926	137	19	19	0.347
GENE_A_16_P58308287	158	21	21	0.400
GENE_A_16_P21204631	131	17	17	0.331
GENE_A_16_P58263249	147	20	20	0.372
GENE_A_18_P13902760	159	23	23	0.402
GENE_A_16_P58273303	139	13	13	0.351
GENE_A_18_P13859250	157	20	20	0.397
GENE_A_18_P13940957	106	14	14	0.268
GENE_A_16_P41426972	139	18	18	0.351
GENE_A_16_P03552048	147	18	18	0.371
GENE_A_16_P03589755	150	17	17	0.379
GENE_A_18_P13926454	152	24	24	0.384
GENE_A_18_P13864539	133	19	19	0.336
GENE_A_16_P58276680	152	22	22	0.384
GENE_A_16_P41429496	149	21	21	0.376
GENE_A_16_P03581529	160	24	24	0.404
GENE_A_16_P03597472	150	18	18	0.378
GENE_A_16_P21197100	159	23	23	0.401
GENE_A_18_P13874770	155	24	24	0.391
GENE_A_16_P58295031	129	14	14	0.325
GENE_A_16_P21237554	147	21	21	0.371
GENE_A_16_P41448552	136	17	17	0.343
GENE_A_16_P41459430	140	16	16	0.353
GENE_A_16_P03579676	159	21	22	0.400
GENE_A_16_P21229490	156	20	20	0.393
GENE_A_16_P58261611	130	21	21	0.327
GENE_A_16_P58268529	146	17	17	0.368
GENE_A_16_P41449806	149	18	19	0.375
GENE_A_16_P58268023	153	17	19	0.385
GENE_A_16_P21197991	161	21	21	0.405
GENE_A_18_P13910078	145	21	21	0.365
GENE_A_18_P13916552	161	20	20	0.405
GENE_A_18_P13904282	169	20	21	0.425
GENE_A_16_P21257445	142	18	18	0.357

GENE_A_16_P03575950	146	21	21	0.367
GENE_A_16_P58304378	163	22	22	0.410
GENE_A_16_P58280087	143	17	17	0.359
GENE_A_16_P58303806	152	19	19	0.382
GENE_A_16_P03589267	139	19	19	0.349
GENE_A_16_P58268506	156	23	24	0.392
GENE_A_16_P21231186	150	18	18	0.377
GENE_A_16_P58293819	148	21	21	0.372
GENE_A_16_P58271623	141	17	17	0.354
GENE_A_16_P21221847	158	19	19	0.397
GENE_A_16_P21244232	146	19	20	0.366
GENE_A_16_P41420789	142	17	17	0.356
GENE_A_18_P13872415	135	18	18	0.339
GENE_A_16_P03564797	147	18	18	0.369
GENE_A_16_P58282658	142	24	24	0.356
GENE_A_16_P21214926	158	21	21	0.396
GENE_A_16_P03596226	167	18	18	0.419
GENE_A_16_P58295326	158	22	22	0.396
GENE_A_16_P41443368	178	25	25	0.446
GENE_A_16_P21210891	145	18	19	0.363
GENE_A_16_P58298983	157	20	20	0.393
GENE_A_16_P41431239	135	19	19	0.338
GENE_A_18_P22485831	162	22	22	0.406
GENE_A_16_P58281894	177	24	24	0.443
GENE_A_16_P58299389	148	22	22	0.371
GENE_A_16_P58306921	156	22	23	0.391
GENE_A_16_P03572133	154	14	14	0.385
GENE_A_16_P21220119	167	18	18	0.418
GENE_A_16_P41406842	141	16	16	0.353
GENE_A_16_P41444656	141	16	16	0.353
GENE_A_16_P21240087	160	19	20	0.400
GENE_A_16_P03573884	172	22	22	0.430
GENE_A_16_P58273535	147	18	18	0.368
GENE_A_16_P21198414	160	21	22	0.400
GENE_A_16_P58289912	154	20	20	0.385
GENE_A_16_P21211838	142	17	17	0.355
GENE_A_16_P03583708	160	19	19	0.399
GENE_A_16_P58274200	166	21	21	0.414
GENE_A_16_P21226713	132	19	19	0.329
GENE_A_16_P41433237	152	16	16	0.379
GENE_A_16_P41446085	159	20	20	0.397
GENE_A_16_P03568275	166	21	21	0.414
GENE_A_16_P41396466	163	21	21	0.407
GENE_A_16_P21211590	158	20	20	0.394
GENE_A_16_P21200909	167	22	22	0.416
GENE_A_16_P58293098	140	19	19	0.349

GENE_A_16_P21205180	137	20	20	0.341
GENE_A_16_P21220702	157	17	17	0.391
GENE_A_16_P21239076	153	22	22	0.381
GENE_A_18_P13926118	180	20	20	0.448
GENE_A_16_P34779847	149	19	20	0.371
GENE_A_16_P58278187	136	14	14	0.338
GENE_A_16_P21227195	170	21	21	0.423
GENE_A_16_P41454063	152	20	20	0.378
GENE_A_16_P58269464	128	16	16	0.318
GENE_A_16_P21221381	156	21	22	0.388
GENE_A_16_P41404033	157	18	18	0.390
GENE_A_16_P03558031	156	20	21	0.388
GENE_A_16_P21233249	164	21	21	0.407
GENE_A_16_P41393928	145	15	15	0.360
GENE_A_16_P34808019	134	19	19	0.333
GENE_A_16_P41445302	176	23	23	0.437
GENE_A_16_P21226542	154	23	23	0.382
GENE_A_16_P58265417	148	17	17	0.368
GENE_A_16_P58305291	138	16	16	0.343
GENE_A_16_P58276337	150	18	18	0.372
GENE_A_16_P21223807	155	21	21	0.385
GENE_A_16_P03591574	167	24	24	0.415
GENE_A_16_P58271040	150	23	23	0.372
GENE_A_16_P03555027	157	20	20	0.390
GENE_A_16_P03572648	134	18	18	0.333
GENE_A_16_P03582516	171	26	27	0.424
GENE_A_16_P41414134	153	19	19	0.380
GENE_A_16_P58269773	140	19	19	0.347
GENE_A_16_P03590546	145	16	17	0.360
GENE_A_16_P58297671	177	23	23	0.439
GENE_A_16_P03573120	155	18	18	0.384
GENE_A_16_P21193575	170	19	19	0.421
GENE_A_16_P21242108	162	20	20	0.402
GENE_A_16_P58282680	151	20	20	0.374
GENE_A_16_P58275460	147	19	19	0.364
GENE_A_16_P21231398	144	18	18	0.357
GENE_A_16_P58296870	138	12	12	0.342
GENE_A_16_P21242053	157	16	16	0.389
GENE_A_16_P58294924	142	18	18	0.352
GENE_A_16_P41398166	133	18	18	0.329
GENE_A_16_P03550895	136	16	16	0.337
GENE_A_16_P58287887	153	16	16	0.379
GENE_A_16_P58268973	143	18	18	0.354
GENE_A_16_P41462527	150	20	20	0.371
GENE_A_18_P13921433	148	20	20	0.366
GENE_A_16_P21208143	134	19	19	0.331

GENE_A_16_P58294883	172	22	22	0.425
GENE_A_16_P58294471	146	20	20	0.361
GENE_A_16_P03580532	149	15	15	0.368
GENE_A_18_P13864558	144	18	18	0.356
GENE_A_18_P13914955	157	22	22	0.388
GENE_A_16_P58307772	153	16	16	0.378
GENE_A_16_P03552108	134	20	20	0.331
GENE_A_16_P21246557	142	17	17	0.351
GENE_A_16_P21204315	156	23	23	0.385
GENE_A_16_P41451241	140	19	19	0.345
GENE_A_16_P21243647	141	19	19	0.348
GENE_A_16_P58263393	166	20	20	0.409
GENE_A_16_P58279942	169	18	18	0.416
GENE_A_18_P22522733	194	24	26	0.478
GENE_A_16_P03558628	146	18	18	0.360
GENE_A_16_P21220404	126	16	17	0.310
GENE_A_16_P58264575	150	14	14	0.369
GENE_A_16_P21216384	161	15	15	0.397
GENE_A_18_P13867675	159	23	23	0.391
GENE_A_16_P21214578	144	18	19	0.355
GENE_A_16_P41407544	164	19	19	0.404
GENE_A_16_P21221426	137	15	15	0.337
GENE_A_16_P21231965	161	23	23	0.396
GENE_A_16_P58290616	144	20	20	0.354
GENE_A_16_P21221175	135	20	20	0.332
GENE_A_16_P58267745	153	20	20	0.376
GENE_A_18_P13936833	128	18	18	0.315
GENE_A_16_P03554136	139	18	18	0.342
GENE_A_16_P21195227	153	19	19	0.376
GENE_A_16_P58285189	164	19	20	0.403
GENE_A_16_P21258159	145	16	16	0.356
GENE_A_16_P21197260	165	20	20	0.406
GENE_A_16_P58304024	145	15	15	0.356
GENE_A_16_P58297236	155	23	23	0.381
GENE_A_16_P58281506	151	18	19	0.371
GENE_A_16_P21231084	147	17	17	0.361
GENE_A_16_P21200897	152	17	17	0.373
GENE_A_16_P03560476	144	18	18	0.354
GENE_A_16_P58278195	158	20	21	0.388
GENE_A_16_P58262716	145	15	15	0.356
GENE_A_16_P58270832	154	21	21	0.378
GENE_A_16_P03560366	130	19	19	0.319
GENE_A_16_P03573538	151	21	21	0.370
GENE_A_18_P22504090	153	22	22	0.375
GENE_A_16_P58260150	168	23	23	0.412
GENE_A_16_P21214831	147	19	19	0.360

GENE_A_16_P41447259	154	23	23	0.377
GENE_A_16_P41469255	145	20	20	0.355
GENE_A_16_P21206410	122	20	20	0.299
GENE_A_18_P13877060	156	19	19	0.382
GENE_A_16_P41394529	155	20	20	0.379
GENE_A_16_P21270624	160	21	21	0.392
GENE_A_16_P58309509	114	16	16	0.279
GENE_A_16_P03582184	137	18	18	0.335
GENE_A_16_P58285570	141	19	19	0.345
GENE_A_16_P21238645	161	22	22	0.394
GENE_A_16_P03585815	160	16	17	0.391
GENE_A_16_P03580487	161	18	19	0.394
GENE_A_16_P58279898	169	19	19	0.413
GENE_A_16_P03592485	150	19	19	0.367
GENE_A_18_P22555207	160	22	22	0.391
GENE_A_16_P58270992	159	19	19	0.389
GENE_A_16_P41467565	164	19	19	0.401
GENE_A_16_P21246261	153	26	26	0.374
GENE_A_16_P58269388	149	20	20	0.364
GENE_A_16_P41400299	143	20	20	0.349
GENE_A_16_P41410665	162	22	22	0.395
GENE_A_16_P21252958	157	19	19	0.383
GENE_A_16_P03558299	154	18	18	0.376
GENE_A_16_P21207899	140	19	19	0.342
GENE_A_16_P58269480	155	20	21	0.378
GENE_A_16_P03581750	148	15	15	0.361
GENE_A_16_P58271402	148	16	17	0.361
GENE_A_16_P58299186	156	24	24	0.380
GENE_A_16_P03573937	172	21	21	0.419
GENE_A_16_P03580695	166	22	22	0.405
GENE_A_16_P21271588	148	20	21	0.361
GENE_A_16_P41443495	137	18	18	0.334
GENE_A_16_P03595009	161	22	23	0.392
GENE_A_16_P21205566	131	16	16	0.319
GENE_A_16_P03589237	167	28	28	0.406
GENE_A_16_P58298656	140	19	19	0.340
GENE_A_16_P58286379	157	16	16	0.382
GENE_A_16_P21210512	147	20	20	0.357
GENE_A_16_P21235826	133	17	17	0.323
GENE_A_16_P41412658	139	17	17	0.338
GENE_A_16_P03598310	160	15	15	0.389
GENE_A_16_P03564860	148	18	18	0.360
GENE_A_16_P58273953	171	22	22	0.415
GENE_A_18_P13905355	146	23	23	0.355
GENE_A_16_P58292627	161	15	15	0.391
GENE_A_16_P03557405	158	21	21	0.384

GENE_A_16_P58288969	164	22	23	0.398
GENE_A_16_P58299923	169	23	23	0.410
GENE_A_16_P03595672	175	15	15	0.425
GENE_A_16_P58273402	158	17	17	0.383
GENE_A_16_P03577141	145	18	18	0.352
GENE_A_16_P21256293	133	18	18	0.323
GENE_A_16_P58306607	156	19	19	0.379
GENE_A_16_P21197291	183	24	24	0.444
GENE_A_18_P13875313	140	18	18	0.340
GENE_A_18_P13938222	135	17	17	0.327
GENE_A_16_P21239088	129	15	15	0.313
GENE_A_16_P58295259	147	16	16	0.356
GENE_A_16_P03586857	131	14	14	0.317
GENE_A_16_P21262273	177	25	25	0.429
GENE_A_16_P03572655	154	22	22	0.373
GENE_A_16_P41417453	119	14	14	0.288
GENE_A_16_P21262405	149	21	22	0.361
GENE_A_16_P41389105	158	20	20	0.382
GENE_A_16_P58285187	168	19	19	0.407
GENE_A_16_P58272215	158	18	18	0.382
GENE_A_16_P58276352	133	15	15	0.322
GENE_A_16_P58304674	134	16	16	0.324
GENE_A_16_P21197324	152	21	21	0.368
GENE_A_18_P22515885	136	18	18	0.329
GENE_A_16_P41429538	144	17	17	0.348
GENE_A_16_P58271578	153	15	15	0.370
GENE_A_18_P22536193	140	21	21	0.339
GENE_A_16_P41411500	158	17	17	0.382
GENE_A_16_P21205665	156	20	20	0.377
GENE_A_16_P41396592	141	19	20	0.341
GENE_A_16_P58285760	145	16	16	0.350
GENE_A_16_P58278164	159	19	19	0.384
GENE_A_16_P58276357	151	16	16	0.365
GENE_A_16_P41439686	146	19	20	0.353
GENE_A_16_P03587355	145	16	17	0.350
GENE_A_16_P21240035	141	21	21	0.340
GENE_A_16_P03568673	153	18	18	0.369
GENE_A_16_P03570617	157	17	17	0.379
GENE_A_16_P03591419	142	22	22	0.343
GENE_A_16_P21215070	142	18	18	0.343
GENE_A_16_P41445985	148	14	15	0.357
GENE_A_16_P58290384	133	15	15	0.321
GENE_A_18_P22531144	138	19	19	0.333
GENE_A_16_P03586961	140	10	10	0.337
GENE_A_16_P58302780	162	20	20	0.390
GENE_A_16_P21248693	158	22	22	0.381

GENE_A_16_P58306218	180	21	21	0.434
GENE_A_16_P58294412	165	22	23	0.397
GENE_A_16_P41413986	165	19	19	0.397
GENE_A_16_P58298452	151	16	16	0.364
GENE_A_16_P41389675	137	18	19	0.330
GENE_A_16_P03567109	135	18	18	0.325
GENE_A_16_P21220805	154	19	19	0.371
GENE_A_16_P58268193	127	15	15	0.306
GENE_A_16_P58302122	125	15	15	0.301
GENE_A_16_P03575241	143	19	19	0.344
GENE_A_16_P41448272	160	22	22	0.385
GENE_A_16_P21207768	169	24	24	0.407
GENE_A_16_P21268683	147	21	21	0.354
GENE_A_18_P13938521	154	16	16	0.370
GENE_A_16_P21244031	130	15	15	0.313
GENE_A_16_P03554301	144	17	17	0.346
GENE_A_16_P21269655	167	23	23	0.401
GENE_A_16_P58309271	129	14	14	0.310
GENE_A_16_P21243746	152	18	18	0.365
GENE_A_16_P58269962	138	14	14	0.331
GENE_A_16_P41390127	158	18	18	0.379
GENE_A_16_P58305824	149	21	22	0.358
GENE_A_16_P58279431	170	14	14	0.408
GENE_A_16_P58263787	168	20	21	0.403
GENE_A_16_P41449342	152	14	14	0.365
GENE_A_16_P58286948	152	21	21	0.365
GENE_A_18_P22500183	130	18	18	0.312
GENE_A_16_P58301202	159	20	21	0.381
GENE_A_16_P58306530	167	16	16	0.400
GENE_A_18_P13898920	136	16	16	0.326
GENE_A_16_P03584659	138	17	17	0.331
GENE_A_16_P58280651	162	19	19	0.388
GENE_A_16_P21230312	155	19	20	0.371
GENE_A_16_P21212535	173	21	21	0.414
GENE_A_16_P03592152	147	19	19	0.352
GENE_A_16_P21197052	164	20	20	0.393
GENE_A_18_P13868512	140	19	19	0.335
GENE_A_16_P58287291	136	21	21	0.326
GENE_A_16_P03596641	162	14	14	0.388
GENE_A_16_P58301873	123	15	15	0.294
GENE_A_16_P21266679	141	16	19	0.337
GENE_A_16_P21217217	134	15	15	0.320
GENE_A_16_P21221845	147	20	20	0.352
GENE_A_16_P58291529	141	15	15	0.337
GENE_A_16_P41414509	175	23	23	0.418
GENE_A_16_P58295701	126	18	18	0.301

GENE_A_16_P41412516	134	19	19	0.320
GENE_A_16_P21213849	154	23	23	0.368
GENE_A_16_P41437779	143	17	18	0.342
GENE_A_18_P13916693	154	17	17	0.368
GENE_A_18_P13876489	151	20	20	0.361
GENE_A_16_P21217872	146	19	19	0.349
GENE_A_16_P58289027	146	18	18	0.349
GENE_A_18_P13890008	152	19	19	0.363
GENE_A_16_P41406054	153	18	18	0.365
GENE_A_16_P41396240	142	16	16	0.339
GENE_A_16_P21215686	146	16	16	0.348
GENE_A_16_P34819312	167	23	23	0.399
GENE_A_16_P21252542	155	21	21	0.370
GENE_A_16_P03585661	141	21	21	0.336
GENE_A_16_P21222108	143	14	14	0.341
GENE_A_16_P41416766	158	21	21	0.377
GENE_A_18_P22587163	164	16	16	0.391
GENE_A_16_P41393158	139	15	15	0.331
GENE_A_16_P58277739	135	19	19	0.322
GENE_A_16_P21204090	138	20	20	0.329
GENE_A_16_P03580473	158	18	18	0.377
GENE_A_16_P58267085	176	22	22	0.420
GENE_A_18_P22575808	160	17	18	0.381
GENE_A_18_P22603243	152	21	21	0.362
GENE_A_16_P41398063	144	16	17	0.343
GENE_A_16_P58294483	124	15	15	0.295
GENE_A_16_P21255508	141	17	17	0.336
GENE_A_14_P104721	166	22	22	0.395
GENE_A_16_P58297826	139	12	12	0.331
GENE_A_18_P22502015	132	15	15	0.314
GENE_A_16_P21256745	142	19	19	0.338
GENE_A_18_P13871376	158	21	21	0.376
GENE_A_16_P58271291	127	15	15	0.302
GENE_A_14_P110524	151	18	18	0.359
GENE_A_16_P58292551	137	16	16	0.326
GENE_A_16_P03587931	157	17	17	0.373
GENE_A_16_P41434341	149	16	16	0.354
GENE_A_16_P41403664	142	16	16	0.337
GENE_A_18_P13856476	140	18	19	0.333
GENE_A_18_P13856324	138	18	18	0.328
GENE_A_16_P03572757	136	16	16	0.323
GENE_A_16_P58277867	158	19	19	0.375
GENE_A_16_P58266574	146	19	19	0.346
GENE_A_16_P58287272	146	16	16	0.346
GENE_A_16_P21214276	138	16	16	0.327
GENE_A_18_P13857061	149	19	20	0.353

GENE_A_18_P13938870	148	17	17	0.351
GENE_A_16_P21260529	154	16	17	0.365
GENE_A_16_P58283649	143	12	12	0.339
GENE_A_16_P58284071	136	24	25	0.322
GENE_A_18_P13925416	157	19	19	0.372
GENE_A_16_P58265179	152	20	20	0.360
GENE_A_16_P21214072	153	20	20	0.362
GENE_A_16_P58274929	134	17	17	0.317
GENE_A_16_P58262471	151	15	15	0.358
GENE_A_18_P22584558	163	21	21	0.386
GENE_A_16_P21205412	157	21	21	0.372
GENE_A_16_P58298018	146	20	20	0.346
GENE_A_16_P58277006	161	22	22	0.381
GENE_A_16_P58261848	132	17	17	0.312
GENE_A_16_P21220979	145	19	19	0.343
GENE_A_16_P21257809	154	18	19	0.364
GENE_A_16_P58304359	162	18	19	0.383
GENE_A_18_P13881338	127	16	17	0.300
GENE_A_16_P58279396	138	15	16	0.326
GENE_A_16_P58300142	160	18	18	0.378
GENE_A_16_P58264522	166	23	23	0.392
GENE_A_16_P41392448	150	18	18	0.354
GENE_A_16_P03597299	166	22	22	0.392
GENE_A_16_P41425688	149	19	19	0.352
GENE_A_16_P58286807	136	18	19	0.321
GENE_A_16_P55114876	150	19	19	0.354
GENE_A_16_P58272133	179	21	21	0.422
GENE_A_16_P21226553	133	17	17	0.314
GENE_A_16_P21201087	143	18	18	0.337
GENE_A_16_P03590282	162	19	19	0.382
GENE_A_18_P13925487	161	18	18	0.380
GENE_A_16_P03594012	140	15	15	0.330
GENE_A_16_P41412949	152	20	20	0.358
GENE_A_16_P21193305	171	19	19	0.403
GENE_A_18_P22581359	161	23	23	0.379
GENE_A_18_P22550519	175	20	20	0.412
GENE_A_16_P58263464	150	19	19	0.353
GENE_A_16_P41460386	137	16	17	0.323
GENE_A_16_P21245782	126	16	16	0.297
GENE_A_16_P34778028	137	17	18	0.322
GENE_A_16_P21209315	157	24	24	0.369
GENE_A_16_P41450223	126	16	16	0.296
GENE_A_16_P03556704	129	15	16	0.303
GENE_A_16_P41399149	152	20	20	0.357
GENE_A_16_P03598303	167	21	22	0.392
GENE_A_18_P13944080	138	18	18	0.324

GENE_A_16_P41456459	148	16	16	0.348
GENE_A_16_P58308467	151	17	18	0.355
GENE_A_16_P41401992	157	24	24	0.369
GENE_A_16_P21217526	152	18	18	0.357
GENE_A_16_P58305630	128	14	14	0.301
GENE_A_18_P22568772	152	15	15	0.357
GENE_A_18_P13855650	138	16	16	0.324
GENE_A_16_P41418790	173	21	21	0.406
GENE_A_16_P58304025	161	21	22	0.378
GENE_A_16_P21205718	148	16	18	0.347
GENE_A_16_P21192980	155	20	22	0.364
GENE_A_18_P22593126	144	17	17	0.338
GENE_A_16_P03574547	167	20	21	0.392
GENE_A_16_P41410472	151	16	16	0.354
GENE_A_16_P03596031	148	15	16	0.347
GENE_A_16_P58268932	167	22	23	0.391
GENE_A_16_P03592772	146	20	20	0.342
GENE_A_16_P21216394	135	13	15	0.316
GENE_A_16_P03575914	159	21	21	0.373
GENE_A_16_P03594489	163	20	20	0.382
GENE_A_18_P13914067	165	18	19	0.387
GENE_A_16_P21204928	148	18	18	0.347
GENE_A_16_P03592966	138	18	18	0.323
GENE_A_18_P13901200	146	17	17	0.342
GENE_A_18_P13890426	141	16	17	0.330
GENE_A_16_P41433398	157	20	20	0.368
GENE_A_16_P34805453	143	14	15	0.335
GENE_A_16_P58286411	156	16	16	0.365
GENE_A_16_P58260003	130	16	16	0.304
GENE_A_18_P13905063	156	21	22	0.365
GENE_A_16_P58261208	146	20	20	0.341
GENE_A_16_P21217567	154	19	19	0.360
GENE_A_16_P58289782	133	13	13	0.311
GENE_A_16_P03597506	179	22	22	0.418
GENE_A_16_P58282825	170	22	22	0.397
GENE_A_16_P03556312	150	19	19	0.350
GENE_A_16_P21259333	155	20	20	0.362
GENE_A_16_P58302149	150	18	18	0.350
GENE_A_16_P21233725	131	14	14	0.306
GENE_A_16_P58266827	149	14	14	0.348
GENE_A_16_P03587273	166	26	27	0.387
GENE_A_16_P41403996	164	23	23	0.383
GENE_A_16_P58291081	167	20	20	0.390
GENE_A_16_P58269467	134	14	14	0.313
GENE_A_18_P22576885	147	20	20	0.343
GENE_A_16_P21246169	134	15	15	0.313

GENE_A_16_P58299167	156	23	23	0.364
GENE_A_14_P105919	156	23	23	0.364
GENE_A_16_P58262961	167	16	16	0.389
GENE_A_16_P58277111	150	17	17	0.350
GENE_A_18_P13871146	155	18	18	0.361
GENE_A_16_P03577853	142	17	17	0.331
GENE_A_14_P103534	145	14	14	0.338
GENE_A_16_P21201568	161	21	21	0.375
GENE_A_16_P41402421	169	20	21	0.394
GENE_A_16_P41429648	156	16	16	0.363
GENE_A_16_P41416864	138	18	18	0.322
GENE_A_16_P03587366	127	10	10	0.296
GENE_A_16_P58305717	151	21	21	0.352
GENE_A_18_P13872534	160	20	20	0.373
GENE_A_18_P13885305	130	14	14	0.303
GENE_A_16_P21192599	142	18	18	0.331
GENE_A_16_P21261083	138	19	19	0.321
GENE_A_16_P03588428	155	20	20	0.361
GENE_A_16_P03575503	155	18	18	0.360
GENE_A_16_P21211115	137	17	17	0.319
GENE_A_16_P41455846	167	19	19	0.388
GENE_A_16_P21230587	156	18	18	0.363
GENE_A_16_P03589771	143	21	21	0.332
GENE_A_16_P21207758	143	16	16	0.332
GENE_A_18_P13869729	140	16	16	0.325
GENE_A_16_P21203809	167	18	18	0.388
GENE_A_16_P21260655	155	17	17	0.360
GENE_A_16_P21229023	152	18	18	0.353
GENE_A_16_P58267367	146	19	19	0.339
GENE_A_16_P41391328	153	18	18	0.355
GENE_A_16_P58305066	148	20	20	0.344
GENE_A_16_P21235486	137	15	15	0.318
GENE_A_16_P21238678	141	13	13	0.327
GENE_A_16_P21189857	145	16	18	0.336
GENE_A_16_P41451363	136	15	17	0.315
GENE_A_16_P21193165	148	21	21	0.343
GENE_A_16_P21232226	184	23	23	0.426
GENE_A_16_P21216710	170	21	21	0.394
GENE_A_16_P21203096	161	17	17	0.373
GENE_A_16_P58290546	170	23	23	0.394
GENE_A_16_P03562471	143	16	16	0.331
GENE_A_16_P21201308	172	22	22	0.398
GENE_A_16_P21205631	156	20	20	0.361
GENE_A_16_P03572614	146	16	17	0.338
GENE_A_16_P58267652	150	18	18	0.347
GENE_A_16_P41401018	157	17	19	0.363

GENE_A_18_P13904656	147	20	20	0.340
GENE_A_18_P22600191	160	24	24	0.370
GENE_A_16_P41417367	144	18	18	0.333
GENE_A_16_P21249341	150	14	14	0.347
GENE_A_16_P03589472	157	17	17	0.363
GENE_A_16_P58296281	146	15	15	0.337
GENE_A_16_P21240412	148	21	21	0.342
GENE_A_16_P21268994	149	19	20	0.344
GENE_A_16_P58283030	167	25	25	0.385
GENE_A_18_P13906687	172	17	17	0.397
GENE_A_16_P21265800	149	16	16	0.344
GENE_A_16_P41391610	148	17	17	0.341
GENE_A_16_P21224070	159	18	18	0.367
GENE_A_18_P22485544	154	20	20	0.355
GENE_A_16_P03589808	155	22	22	0.357
GENE_A_16_P21218787	163	19	19	0.375
GENE_A_16_P41426298	153	21	21	0.352
GENE_A_16_P41425403	148	17	17	0.341
GENE_A_16_P58302947	146	15	15	0.336
GENE_A_16_P21221662	149	19	19	0.343
GENE_A_16_P41461118	135	13	13	0.311
GENE_A_16_P58278424	157	21	21	0.361
GENE_A_16_P03575653	163	19	19	0.375
GENE_A_16_P21256438	170	22	22	0.391
GENE_A_16_P58303042	182	19	19	0.418
GENE_A_18_P13914023	155	19	20	0.356
GENE_A_16_P58297428	161	18	18	0.370
GENE_A_16_P58267962	165	20	20	0.379
GENE_A_16_P58302088	182	23	23	0.418
GENE_A_16_P41394032	152	18	18	0.349
GENE_A_16_P21196702	175	20	20	0.402
GENE_A_16_P41402407	161	20	20	0.370
GENE_A_16_P41426347	154	19	19	0.354
GENE_A_16_P41398932	169	19	19	0.388
GENE_A_16_P21233148	149	22	22	0.342
GENE_A_16_P58264709	152	15	15	0.349
GENE_A_16_P58271112	167	20	20	0.383
GENE_A_16_P41439282	159	16	16	0.365
GENE_A_16_P41398317	148	19	19	0.339
GENE_A_16_P21227260	158	18	18	0.362
GENE_A_16_P41426970	147	18	19	0.337
GENE_A_16_P58274420	158	16	17	0.362
GENE_A_18_P13933200	126	17	17	0.289
GENE_A_18_P22520130	149	22	22	0.341
GENE_A_18_P22599306	136	16	16	0.312
GENE_A_16_P03594760	167	18	19	0.383

GENE_A_16_P03595844	144	16	17	0.330
GENE_A_16_P21239403	123	16	16	0.282
GENE_A_18_P13898722	151	15	15	0.346
GENE_A_16_P58262145	137	17	17	0.314
GENE_A_18_P22554425	146	17	17	0.334
GENE_A_16_P03590383	149	19	20	0.341
GENE_A_16_P58296596	142	20	20	0.325
GENE_A_16_P21249470	147	22	23	0.336
GENE_A_16_P58286600	174	19	19	0.398
GENE_A_16_P58288676	155	16	16	0.354
GENE_A_16_P21260003	156	17	17	0.357
GENE_A_16_P21249641	144	17	17	0.329
GENE_A_16_P21224511	135	16	17	0.309
GENE_A_16_P03555863	158	20	20	0.361
GENE_A_16_P03585758	125	15	15	0.286
GENE_A_16_P03588421	157	16	16	0.359
GENE_A_16_P21214710	166	21	21	0.379
GENE_A_16_P58307956	147	15	16	0.336
GENE_A_16_P21226708	145	14	15	0.331
GENE_A_18_P13887506	162	19	19	0.370
GENE_A_16_P21202117	167	20	21	0.381
GENE_A_16_P21195529	121	14	14	0.276
GENE_A_16_P41410182	140	19	19	0.320
GENE_A_18_P13893204	150	17	18	0.342
GENE_A_18_P22598169	154	16	16	0.352
GENE_A_16_P41387094	139	20	21	0.317
GENE_A_16_P21219578	156	18	18	0.356
GENE_A_16_P21230951	126	16	16	0.288
GENE_A_16_P58280740	149	16	16	0.340
GENE_A_16_P21217718	146	16	16	0.333
GENE_A_16_P21242319	139	18	18	0.317
GENE_A_16_P21227792	155	20	20	0.353
GENE_A_16_P58285167	128	13	13	0.292
GENE_A_16_P21196612	147	21	21	0.335
GENE_A_16_P21224143	149	17	17	0.340
GENE_A_16_P03590290	169	21	21	0.385
GENE_A_16_P58293401	154	18	18	0.351
GENE_A_16_P58274421	142	15	15	0.323
GENE_A_16_P58281578	135	16	16	0.307
GENE_A_18_P13878111	149	19	19	0.339
GENE_A_16_P21208506	148	15	16	0.337
GENE_A_16_P41392344	138	17	17	0.314
GENE_A_16_P21223166	139	19	19	0.316
GENE_A_16_P21272461	127	16	18	0.289
GENE_A_16_P21203550	149	21	21	0.339
GENE_A_16_P21193752	139	17	17	0.316

GENE_A_16_P03592397	130	13	13	0.295
GENE_A_18_P13863810	152	17	17	0.345
GENE_A_16_P21244069	160	25	25	0.364
GENE_A_16_P58290974	170	22	22	0.386
GENE_A_16_P21194056	142	18	18	0.323
GENE_A_16_P21212338	156	21	21	0.354
GENE_A_16_P21230953	136	13	13	0.309
GENE_A_16_P58273357	153	18	18	0.347
GENE_A_16_P03558626	174	16	16	0.395
GENE_A_18_P13876157	140	19	19	0.318
GENE_A_16_P58279118	153	17	18	0.347
GENE_A_16_P58301578	151	19	19	0.343
GENE_A_16_P21216520	138	14	14	0.313
GENE_A_16_P21210182	147	14	14	0.333
GENE_A_16_P41406927	129	16	16	0.293
GENE_A_16_P21231308	160	21	21	0.363
GENE_A_16_P03583758	170	15	15	0.385
GENE_A_16_P58297641	146	19	19	0.331
GENE_A_18_P13897959	167	20	20	0.378
GENE_A_16_P58272735	145	17	17	0.329
GENE_A_16_P03553980	165	18	18	0.374
GENE_A_16_P21228887	149	19	19	0.338
GENE_A_16_P41436064	144	20	20	0.326
GENE_A_16_P03585100	153	19	19	0.346
GENE_A_18_P22530530	148	17	17	0.335
GENE_A_16_P41393256	151	18	18	0.342
GENE_A_16_P41440857	147	20	20	0.333
GENE_A_16_P03596686	137	13	13	0.310
GENE_A_16_P03566214	152	17	17	0.344
GENE_A_16_P21266690	150	18	18	0.339
GENE_A_16_P58260207	152	20	20	0.344
GENE_A_16_P58296625	148	19	19	0.335
GENE_A_16_P41396045	146	15	15	0.330
GENE_A_16_P21260133	158	21	21	0.357
GENE_A_16_P21254193	152	20	20	0.344
GENE_A_16_P03577273	158	15	16	0.357
GENE_A_16_P58299332	129	17	17	0.292
GENE_A_18_P22536002	151	16	16	0.341
GENE_A_16_P41423495	144	16	16	0.326
GENE_A_16_P41416652	155	16	16	0.350
GENE_A_16_P41396480	146	15	15	0.330
GENE_A_16_P58273398	154	18	18	0.348
GENE_A_16_P03555543	152	19	19	0.344
GENE_A_16_P58277995	147	20	20	0.332
GENE_A_16_P21258393	154	18	18	0.348
GENE_A_18_P13893086	153	14	14	0.346

GENE_A_16_P03572482	150	12	12	0.339
GENE_A_16_P21202443	150	18	18	0.339
GENE_A_16_P58295081	153	20	20	0.345
GENE_A_16_P21242314	148	18	18	0.334
GENE_A_16_P58272826	155	18	18	0.350
GENE_A_16_P58305446	132	17	17	0.298
GENE_A_16_P03588580	149	18	20	0.336
GENE_A_16_P03551152	146	19	19	0.330
GENE_A_16_P58285468	147	17	17	0.332
GENE_A_16_P58281679	152	19	20	0.343
GENE_A_18_P22487331	163	23	23	0.368
GENE_A_16_P21194684	150	21	21	0.338
GENE_A_16_P21197140	152	20	21	0.342
GENE_A_18_P13918085	158	18	18	0.356
GENE_A_16_P55119400	140	13	13	0.315
GENE_A_16_P58271378	134	16	16	0.302
GENE_A_16_P21221247	157	20	20	0.353
GENE_A_16_P21212426	133	15	15	0.299
GENE_A_16_P21233588	161	21	21	0.362
GENE_A_16_P58278402	157	18	18	0.353
GENE_A_18_P13939828	164	18	18	0.369
GENE_A_16_P58305712	145	15	15	0.326
GENE_A_18_P13858481	165	20	20	0.371
GENE_A_16_P03553740	168	23	23	0.378
GENE_A_18_P13868075	153	20	21	0.344
GENE_A_16_P21263030	161	21	21	0.362
GENE_A_16_P21229662	142	16	18	0.319
GENE_A_16_P03588129	130	13	14	0.292
GENE_A_16_P41411120	149	18	18	0.335
GENE_A_16_P41449881	142	16	16	0.319
GENE_A_16_P41460279	150	17	17	0.337
GENE_A_16_P03570257	171	20	20	0.384
GENE_A_16_P58292254	163	19	19	0.366
GENE_A_16_P21226677	164	19	19	0.368
GENE_A_16_P21250331	161	18	19	0.361
GENE_A_16_P58288074	156	19	19	0.350
GENE_A_16_P03596511	172	17	17	0.386
GENE_A_18_P13940331	149	16	16	0.334
GENE_A_16_P58261517	148	19	19	0.332
GENE_A_18_P13893456	163	21	21	0.366
GENE_A_16_P21197089	131	18	18	0.294
GENE_A_16_P03572572	139	17	17	0.312
GENE_A_16_P58297500	141	16	16	0.316
GENE_A_16_P58305503	161	23	23	0.361
GENE_A_16_P03556598	156	19	19	0.350
GENE_A_16_P21224137	136	17	17	0.305

GENE_A_16_P03585821	156	17	18	0.350
GENE_A_16_P41426480	166	18	18	0.372
GENE_A_16_P21246200	132	18	18	0.296
GENE_A_16_P03580333	136	17	17	0.305
GENE_A_16_P03593070	151	15	15	0.338
GENE_A_16_P58277680	149	18	18	0.334
GENE_A_16_P21209262	162	20	21	0.363
GENE_A_18_P13888344	161	19	19	0.360
GENE_A_16_P41432671	140	15	15	0.313
GENE_A_16_P21253464	155	21	21	0.347
GENE_A_16_P58277803	154	17	17	0.345
GENE_A_16_P03597800	172	15	16	0.385
GENE_A_18_P13898510	158	19	19	0.353
GENE_A_18_P13877285	145	16	16	0.324
GENE_A_18_P22541558	148	16	16	0.331
GENE_A_16_P21250285	172	21	21	0.385
GENE_A_16_P21240712	169	21	21	0.378
GENE_A_18_P13908664	160	17	18	0.358
GENE_A_16_P41442135	160	19	19	0.358
GENE_A_16_P03575306	142	15	15	0.317
GENE_A_16_P21238078	159	19	19	0.355
GENE_A_16_P21197901	154	18	18	0.344
GENE_A_16_P21227505	143	15	15	0.319
GENE_A_18_P22552974	146	14	14	0.326
GENE_A_16_P58290643	145	17	17	0.324
GENE_A_16_P03591135	144	20	20	0.322
GENE_A_18_P13857890	144	17	17	0.322
GENE_A_16_P58274118	144	17	17	0.322
GENE_A_18_P13917910	153	17	17	0.342
GENE_A_16_P21208381	159	19	19	0.355
GENE_A_16_P21207182	151	20	20	0.337
GENE_A_16_P41458995	140	17	17	0.312
GENE_A_18_P13888392	156	17	17	0.348
GENE_A_16_P41391046	155	17	17	0.346
GENE_A_16_P58280050	146	21	21	0.326
GENE_A_16_P03596284	125	14	14	0.279
GENE_A_16_P03574454	165	20	20	0.368
GENE_A_16_P34819799	176	19	19	0.393
GENE_A_16_P21243653	154	18	18	0.344
GENE_A_16_P03595577	148	16	16	0.330
GENE_A_16_P21215078	135	14	14	0.301
GENE_A_16_P03592547	146	16	16	0.326
GENE_A_16_P58309144	157	22	22	0.350
GENE_A_16_P58280104	153	21	21	0.341
GENE_A_16_P41422139	143	15	15	0.319
GENE_A_16_P21197048	131	13	14	0.292

GENE_A_16_P03553121	132	16	16	0.294
GENE_A_16_P03589197	171	23	23	0.381
GENE_A_16_P41430991	146	17	17	0.325
GENE_A_18_P13859015	171	18	18	0.381
GENE_A_16_P03590924	144	17	18	0.321
GENE_A_18_P22558154	143	15	15	0.318
GENE_A_16_P58293890	157	19	19	0.349
GENE_A_16_P58267929	139	19	19	0.309
GENE_A_16_P21205314	175	19	19	0.389
GENE_A_16_P21204042	165	20	20	0.367
GENE_A_16_P03593234	165	19	19	0.367
GENE_A_18_P13905259	135	14	14	0.300
GENE_A_16_P58272834	162	21	22	0.360
GENE_A_16_P21240976	163	19	19	0.362
GENE_A_16_P03584822	143	17	17	0.317
GENE_A_16_P03551036	159	19	19	0.353
GENE_A_16_P41415362	140	17	17	0.311
GENE_A_16_P03578747	153	23	23	0.340
GENE_A_16_P03579066	162	17	17	0.359
GENE_A_16_P58270588	149	18	18	0.331
GENE_A_16_P58276776	144	16	16	0.320
GENE_A_16_P41396988	188	25	25	0.417
GENE_A_16_P03592161	163	20	20	0.362
GENE_A_16_P41457254	170	22	22	0.377
GENE_A_16_P21193693	158	17	17	0.350
GENE_A_16_P58283918	149	17	17	0.330
GENE_A_18_P13886106	140	15	15	0.310
GENE_A_16_P03591685	156	19	19	0.346
GENE_A_16_P58309403	173	18	18	0.383
GENE_A_16_P03589502	146	15	15	0.324
GENE_A_16_P21219185	163	22	22	0.361
GENE_A_16_P03588801	148	18	18	0.328
GENE_A_18_P22550077	155	16	16	0.343
GENE_A_18_P13877653	142	15	15	0.315
GENE_A_16_P34778299	138	15	15	0.306
GENE_A_16_P03583014	153	19	19	0.339
GENE_A_16_P58302809	153	15	16	0.339
GENE_A_16_P03586390	156	16	17	0.345
GENE_A_16_P58276499	158	19	19	0.350
GENE_A_16_P58279038	154	17	17	0.341
GENE_A_16_P58285510	132	19	19	0.292
GENE_A_16_P58283176	151	16	17	0.334
GENE_A_16_P21263746	140	13	13	0.310
GENE_A_18_P22527564	139	14	14	0.307
GENE_A_16_P21219924	161	20	20	0.356
GENE_A_18_P13923502	150	16	16	0.332

GENE_A_16_P41410658	133	15	15	0.294
GENE_A_16_P21236391	160	16	17	0.354
GENE_A_16_P58278534	175	19	19	0.387
GENE_A_16_P41436793	149	18	18	0.329
GENE_A_16_P21194848	134	15	15	0.296
GENE_A_16_P03581400	158	18	18	0.349
GENE_A_16_P41441764	163	24	24	0.360
GENE_A_16_P58304846	162	19	19	0.358
GENE_A_18_P22598259	161	17	17	0.355
GENE_A_16_P58296197	181	20	20	0.400
GENE_A_16_P41456286	151	22	23	0.333
GENE_A_16_P41447635	147	17	17	0.324
GENE_A_16_P58279285	148	15	17	0.327
GENE_A_16_P34774546	173	25	25	0.382
GENE_A_16_P58276897	150	19	19	0.331
GENE_A_16_P58266665	141	13	14	0.311
GENE_A_16_P41463230	145	19	19	0.320
GENE_A_16_P21211700	163	16	16	0.359
GENE_A_16_P41449876	147	19	19	0.324
GENE_A_16_P03555228	129	15	15	0.284
GENE_A_16_P58274156	163	20	20	0.359
GENE_A_16_P21258135	166	22	22	0.366
GENE_A_18_P13876861	128	13	13	0.282
GENE_A_16_P21261826	133	14	14	0.293
GENE_A_18_P13890533	144	19	19	0.317
GENE_A_16_P21232816	168	18	18	0.370
GENE_A_16_P58304934	148	17	18	0.326
GENE_A_16_P58264556	151	16	16	0.332
GENE_A_18_P13910750	166	22	22	0.365
GENE_A_16_P03575407	154	18	18	0.339
GENE_A_18_P22541164	145	17	17	0.319
GENE_A_16_P21270374	147	19	19	0.323
GENE_A_16_P21208570	152	13	13	0.334
GENE_A_16_P21236987	149	18	18	0.328
GENE_A_16_P41394752	141	19	19	0.310
GENE_A_16_P03595741	155	17	17	0.341
GENE_A_16_P03591150	149	16	16	0.328
GENE_A_16_P58281745	144	16	16	0.317
GENE_A_16_P41464020	140	15	16	0.308
GENE_A_16_P41414024	129	17	17	0.284
GENE_A_16_P21240024	144	16	16	0.316
GENE_A_16_P41413963	138	19	19	0.303
GENE_A_14_P109866	151	16	16	0.332
GENE_A_16_P58277713	150	18	19	0.329
GENE_A_18_P22576198	152	17	18	0.334
GENE_A_16_P58270097	141	13	13	0.309

GENE_A_18_P13877180	137	13	13	0.301
GENE_A_16_P58268807	166	20	20	0.364
GENE_A_16_P41414646	154	17	17	0.338
GENE_A_16_P03556176	173	21	21	0.379
GENE_A_18_P22481454	157	17	17	0.344
GENE_A_16_P03588543	130	15	15	0.285
GENE_A_16_P21197084	129	17	17	0.283
GENE_A_16_P41409189	150	16	16	0.329
GENE_A_16_P58272604	145	17	17	0.318
GENE_A_16_P21213637	156	17	17	0.342
GENE_A_16_P58279314	157	18	18	0.344
GENE_A_16_P41408795	141	19	19	0.309
GENE_A_16_P03585249	130	12	12	0.285
GENE_A_18_P13872551	132	13	13	0.289
GENE_A_16_P58275380	144	17	17	0.315
GENE_A_16_P21208979	156	17	17	0.342
GENE_A_16_P03589716	127	14	14	0.278
GENE_A_16_P21239195	162	19	20	0.355
GENE_A_16_P21237444	147	19	19	0.322
GENE_A_16_P41427814	139	14	15	0.304
GENE_A_18_P22493961	154	17	17	0.337
GENE_A_16_P03598032	155	18	18	0.339
GENE_A_16_P21196782	156	19	19	0.341
GENE_A_16_P58266499	142	19	19	0.311
GENE_A_16_P21198226	164	16	16	0.359
GENE_A_16_P58287675	148	12	12	0.324
GENE_A_16_P58300832	134	15	15	0.293
GENE_A_16_P58266703	152	16	16	0.332
GENE_A_16_P58307941	142	16	16	0.310
GENE_A_16_P34819962	136	17	17	0.297
GENE_A_16_P21218847	154	18	18	0.337
GENE_A_16_P21229162	166	16	17	0.363
GENE_A_16_P58273835	129	17	17	0.282
GENE_A_18_P13879334	159	15	15	0.347
GENE_A_16_P41437435	149	15	15	0.325
GENE_A_16_P21226980	146	15	16	0.319
GENE_A_16_P58307216	151	17	17	0.330
GENE_A_18_P13914998	159	14	14	0.347
GENE_A_16_P41408607	133	13	13	0.290
GENE_A_16_P03596010	144	16	16	0.314
GENE_A_16_P58290442	149	19	19	0.325
GENE_A_16_P03590329	152	17	17	0.332
GENE_A_16_P21242013	153	14	14	0.334
GENE_A_16_P41408063	144	18	18	0.314
GENE_A_18_P22565302	143	13	13	0.312
GENE_A_16_P21223453	177	19	19	0.386

GENE_A_16_P03588787	175	18	18	0.382
GENE_A_18_P13943675	142	17	17	0.310
GENE_A_16_P58261251	134	16	17	0.292
GENE_A_16_P58270763	134	14	14	0.292
GENE_A_16_P21234101	162	18	18	0.353
GENE_A_18_P13916376	129	12	12	0.281
GENE_A_16_P21248144	144	17	17	0.314
GENE_A_16_P03595666	175	18	18	0.381
GENE_A_16_P03571822	136	16	16	0.296
GENE_A_16_P58295544	147	17	17	0.320
GENE_A_16_P03559312	161	21	21	0.351
GENE_A_16_P58268201	168	14	14	0.366
GENE_A_16_P21214758	147	17	17	0.320
GENE_A_16_P03553770	137	17	17	0.298
GENE_A_16_P58280012	168	15	15	0.366
GENE_A_16_P03578000	169	20	20	0.368
GENE_A_16_P58275125	138	16	16	0.300
GENE_A_16_P21231891	159	21	21	0.346
GENE_A_16_P58291155	148	18	18	0.322
GENE_A_16_P58292482	143	17	17	0.311
GENE_A_18_P13928348	145	18	18	0.315
GENE_A_18_P13867586	149	20	20	0.324
GENE_A_16_P58305875	144	16	16	0.313
GENE_A_16_P58301720	135	16	16	0.293
GENE_A_16_P21204538	166	20	21	0.361
GENE_A_16_P41435813	157	16	16	0.341
GENE_A_16_P21201503	160	19	19	0.348
GENE_A_16_P03584216	145	19	19	0.315
GENE_A_16_P21253033	150	16	16	0.326
GENE_A_16_P58268476	149	17	17	0.324
GENE_A_16_P58295321	150	16	16	0.326
GENE_A_16_P41391234	150	15	15	0.326
GENE_A_16_P21262097	172	22	22	0.373
GENE_A_18_P13920172	142	18	18	0.308
GENE_A_16_P41403602	128	14	14	0.278
GENE_A_16_P21212527	164	17	17	0.356
GENE_A_16_P21262657	155	18	18	0.336
GENE_A_16_P03578883	129	14	14	0.280
GENE_A_16_P21231987	165	19	19	0.358
GENE_A_16_P21217215	166	20	20	0.360
GENE_A_18_P22598912	155	17	18	0.336
GENE_A_16_P03593487	146	16	17	0.316
GENE_A_16_P58301394	160	18	18	0.347
GENE_A_16_P21218674	160	20	20	0.347
GENE_A_16_P03577934	155	18	18	0.336
GENE_A_16_P41453451	142	14	14	0.308

GENE_A_16_P58283050	154	18	18	0.334
GENE_A_16_P03566478	142	13	13	0.308
GENE_A_16_P58294621	169	19	19	0.366
GENE_A_16_P58278341	158	19	19	0.342
GENE_A_16_P21263557	152	14	14	0.329
GENE_A_18_P13895636	138	17	17	0.299
GENE_A_16_P03580534	137	16	16	0.296
GENE_A_16_P21204098	164	16	16	0.355
GENE_A_16_P41457752	138	18	19	0.298
GENE_A_16_P58281304	156	21	21	0.337
GENE_A_16_P41438750	131	15	15	0.283
GENE_A_16_P21243272	119	13	13	0.257
GENE_A_18_P22543306	152	17	17	0.329
GENE_A_16_P21266234	144	19	19	0.311
GENE_A_18_P13860427	172	20	20	0.372
GENE_A_16_P21227385	129	13	13	0.279
GENE_A_16_P03593132	154	16	16	0.333
GENE_A_16_P58261757	151	17	17	0.326
GENE_A_16_P34812867	155	19	19	0.335
GENE_A_18_P22602960	145	16	16	0.313
GENE_A_16_P34767466	139	15	15	0.300
GENE_A_16_P58277863	154	19	19	0.333
GENE_A_16_P03587087	138	16	16	0.298
GENE_A_16_P21219906	155	20	20	0.335
GENE_A_16_P58260802	168	19	19	0.363
GENE_A_16_P03579183	166	20	20	0.358
GENE_A_14_P130577	152	17	17	0.328
GENE_A_18_P22567727	138	15	15	0.298
GENE_A_16_P58263454	167	18	18	0.360
GENE_A_16_P58268243	128	13	14	0.276
GENE_A_16_P58260926	134	17	17	0.289
GENE_A_16_P21249115	170	24	24	0.367
GENE_A_16_P58291518	159	12	13	0.343
GENE_A_16_P21201489	168	21	21	0.362
GENE_A_16_P03594174	146	10	11	0.315
GENE_A_16_P58272207	142	15	15	0.306
GENE_A_16_P41391890	152	21	22	0.328
GENE_A_16_P21244455	154	15	15	0.332
GENE_A_16_P21195172	151	19	19	0.325
GENE_A_16_P58308257	147	16	16	0.317
GENE_A_16_P21217999	146	14	14	0.315
GENE_A_16_P41446516	137	17	17	0.295
GENE_A_16_P21255463	149	18	18	0.321
GENE_A_18_P13870710	166	18	18	0.358
GENE_A_16_P21255388	128	15	15	0.276
GENE_A_18_P22596989	146	16	16	0.314

GENE_A_16_P58300795	150	15	15	0.323
GENE_A_16_P58268579	191	20	20	0.411
GENE_A_16_P03591715	158	20	20	0.340
GENE_A_16_P58308950	166	23	23	0.357
GENE_A_16_P21200136	148	17	17	0.318
GENE_A_18_P13924043	153	17	17	0.329
GENE_A_16_P34814272	148	15	15	0.318
GENE_A_16_P21254865	142	17	18	0.305
GENE_A_16_P55111816	137	18	18	0.295
GENE_A_16_P58278712	144	17	17	0.310
GENE_A_16_P58297392	144	16	16	0.310
GENE_A_16_P58274040	147	17	17	0.316
GENE_A_16_P34768213	144	18	18	0.309
GENE_A_16_P03576514	162	17	17	0.348
GENE_A_16_P21213962	170	18	19	0.365
GENE_A_16_P41442935	142	16	16	0.305
GENE_A_18_P13899592	152	18	18	0.327
GENE_A_16_P21272434	158	16	16	0.339
GENE_A_16_P41445275	160	17	18	0.344
GENE_A_16_P03581810	146	15	15	0.314
GENE_A_16_P41437294	161	19	20	0.346
GENE_A_18_P13868822	160	15	15	0.343
GENE_A_16_P03566908	143	17	17	0.307
GENE_A_16_P58299195	147	14	14	0.315
GENE_A_16_P58304344	155	19	19	0.333
GENE_A_16_P41409262	158	18	18	0.339
GENE_A_16_P03595802	146	19	19	0.313
GENE_A_18_P22525936	139	14	14	0.298
GENE_A_16_P21260929	146	18	18	0.313
GENE_A_16_P41450521	137	14	14	0.294
GENE_A_16_P21226731	157	23	23	0.337
GENE_A_16_P58280565	144	16	16	0.309
GENE_A_16_P03581791	158	18	18	0.339
GENE_A_16_P41409923	173	16	16	0.371
GENE_A_16_P58304284	141	14	14	0.302
GENE_A_16_P03594111	159	17	17	0.341
GENE_A_16_P41426423	157	15	15	0.336
GENE_A_16_P03566923	169	20	20	0.362
GENE_A_16_P21212322	162	17	17	0.347
GENE_A_16_P03591073	184	17	17	0.394
GENE_A_16_P58302626	155	20	20	0.332
GENE_A_16_P41415759	159	21	21	0.341
GENE_A_16_P41388737	165	18	19	0.353
GENE_A_16_P58299654	157	18	18	0.336
GENE_A_16_P34784150	156	16	16	0.334
GENE_A_16_P58262028	123	12	12	0.263

GENE_A_16_P58293828	165	18	18	0.353
GENE_A_16_P58265752	153	19	19	0.327
GENE_A_16_P03594112	162	22	22	0.347
GENE_A_16_P58278764	132	14	14	0.282
GENE_A_16_P03595808	164	17	17	0.351
GENE_A_16_P58293926	145	17	17	0.310
GENE_A_16_P21221610	146	18	18	0.312
GENE_A_16_P03552992	167	18	18	0.357
GENE_A_16_P03586607	131	13	13	0.280
GENE_A_16_P21271879	160	16	16	0.342
GENE_A_16_P21245012	152	16	16	0.325
GENE_A_16_P21240347	158	14	14	0.338
GENE_A_16_P03597735	146	16	16	0.312
GENE_A_18_P13935156	159	20	20	0.340
GENE_A_18_P13927025	155	17	17	0.331
GENE_A_16_P58265306	142	16	16	0.303
GENE_A_16_P21214654	137	17	17	0.292
GENE_A_16_P21195717	153	17	17	0.327
GENE_A_16_P21221447	141	18	18	0.301
GENE_A_16_P03552415	150	18	18	0.320
GENE_A_16_P03590935	148	18	18	0.316
GENE_A_16_P03576449	143	14	14	0.305
GENE_A_16_P58266766	149	18	18	0.318
GENE_A_16_P03579877	168	20	20	0.358
GENE_A_16_P21265023	178	20	20	0.379
GENE_A_16_P03591059	156	13	13	0.332
GENE_A_16_P03592055	142	15	15	0.303
GENE_A_16_P41400099	134	15	15	0.286
GENE_A_18_P13867333	163	18	19	0.347
GENE_A_16_P58294304	157	16	16	0.334
GENE_A_16_P41447797	135	17	18	0.288
GENE_A_16_P03591738	136	14	14	0.290
GENE_A_16_P21263474	150	18	18	0.319
GENE_A_16_P58282960	150	16	16	0.319
GENE_A_16_P58283683	134	14	14	0.285
GENE_A_18_P13930019	147	17	17	0.313
GENE_A_16_P03582499	134	17	18	0.285
GENE_A_16_P41431289	165	18	18	0.351
GENE_A_18_P22589321	157	19	19	0.334
GENE_A_14_P112018	132	15	15	0.281
GENE_A_16_P41455458	152	21	21	0.323
GENE_A_16_P58275801	154	15	15	0.327
GENE_A_16_P58300158	142	16	17	0.302
GENE_A_16_P21200774	148	19	19	0.314
GENE_A_16_P58272187	156	18	18	0.331
GENE_A_16_P41465427	149	15	15	0.316

GENE_A_16_P03588814	152	17	17	0.323
GENE_A_16_P58279446	173	20	20	0.367
GENE_A_16_P58291242	158	18	18	0.335
GENE_A_16_P21194105	147	13	13	0.312
GENE_A_16_P03569300	144	17	17	0.305
GENE_A_16_P58297827	143	15	15	0.303
GENE_A_16_P41445745	143	13	13	0.303
GENE_A_18_P22582138	143	15	15	0.303
GENE_A_16_P03553810	140	16	16	0.297
GENE_A_16_P21246748	165	18	18	0.350
GENE_A_16_P58278755	155	18	18	0.328
GENE_A_16_P21237795	151	15	15	0.320
GENE_A_16_P58269617	157	19	19	0.333
GENE_A_16_P21223659	150	18	18	0.318
GENE_A_16_P41416335	152	18	18	0.322
GENE_A_16_P21213289	130	16	16	0.275
GENE_A_16_P34797772	180	19	19	0.381
GENE_A_16_P41440696	162	17	17	0.343
GENE_A_16_P03597178	153	18	18	0.324
GENE_A_16_P58285457	160	17	18	0.339
GENE_A_16_P58294957	135	13	13	0.286
GENE_A_16_P21239099	148	18	18	0.313
GENE_A_16_P21259916	141	13	13	0.298
GENE_A_16_P21209989	139	15	16	0.294
GENE_A_16_P58261699	152	19	19	0.321
GENE_A_16_P41395004	173	20	20	0.366
GENE_A_16_P21211720	149	16	16	0.315
GENE_A_18_P13900868	138	16	16	0.292
GENE_A_16_P55109259	142	20	20	0.300
GENE_A_16_P03594960	139	11	11	0.294
GENE_A_16_P58272556	138	17	18	0.292
GENE_A_16_P58276895	155	16	16	0.328
GENE_A_16_P21237451	152	16	16	0.321
GENE_A_16_P21218159	133	13	13	0.281
GENE_A_16_P21201711	141	15	15	0.298
GENE_A_16_P03577164	158	18	18	0.334
GENE_A_16_P21212840	146	17	18	0.308
GENE_A_16_P03571789	135	17	17	0.285
GENE_A_16_P58300882	158	16	16	0.334
GENE_A_16_P34820844	136	14	14	0.287
GENE_A_16_P41430807	146	15	15	0.308
GENE_A_16_P21236747	141	18	18	0.298
GENE_A_16_P41426590	130	14	14	0.275
GENE_A_16_P03582737	139	13	13	0.294
GENE_A_16_P21224403	148	16	16	0.313
GENE_A_16_P21227098	154	16	16	0.325

GENE_A_16_P58303276	161	16	16	0.340
GENE_A_16_P58271921	137	14	14	0.289
GENE_A_16_P58282876	155	16	17	0.327
GENE_A_16_P21245534	150	19	19	0.316
GENE_A_16_P58301598	176	21	21	0.371
GENE_A_16_P34811673	148	15	15	0.312
GENE_A_18_P22550128	154	16	16	0.325
GENE_A_16_P58290470	153	18	18	0.322
GENE_A_16_P03578406	167	19	19	0.352
GENE_A_16_P21193146	171	19	19	0.360
GENE_A_16_P41407610	173	21	21	0.364
GENE_A_16_P58277590	167	18	18	0.352
GENE_A_16_P41389566	149	12	12	0.314
GENE_A_16_P21261554	141	12	12	0.297
GENE_A_18_P13943946	145	16	16	0.305
GENE_A_16_P03591071	152	16	16	0.320
GENE_A_16_P58288032	153	18	18	0.322
GENE_A_16_P58266670	146	17	17	0.307
GENE_A_16_P41451019	128	17	17	0.269
GENE_A_16_P21239492	148	21	21	0.311
GENE_A_16_P58267216	142	16	16	0.298
GENE_A_16_P03591625	159	19	19	0.334
GENE_A_18_P22591639	151	18	19	0.317
GENE_A_16_P58271947	125	13	13	0.263
GENE_A_16_P41402664	141	16	16	0.296
GENE_A_16_P21222058	153	14	14	0.321
GENE_A_16_P41445691	144	17	17	0.302
GENE_A_16_P21234536	170	20	20	0.357
GENE_A_16_P58269520	161	17	17	0.338
GENE_A_18_P13943120	139	16	16	0.292
GENE_A_16_P58270240	150	16	16	0.315
GENE_A_16_P21211660	150	16	16	0.315
GENE_A_16_P41420592	151	16	16	0.317
GENE_A_16_P41429047	160	16	16	0.336
GENE_A_16_P21217594	139	19	19	0.292
GENE_A_16_P41404448	162	18	18	0.340
GENE_A_16_P03557564	142	15	15	0.298
GENE_A_16_P03582877	156	18	18	0.327
GENE_A_16_P03552540	179	19	20	0.376
GENE_A_16_P03575131	140	16	16	0.294
GENE_A_16_P41423587	144	16	16	0.302
GENE_A_18_P13870830	166	19	19	0.348
GENE_A_16_P21237863	142	14	14	0.298
GENE_A_16_P21201138	161	17	17	0.338
GENE_A_18_P13878092	142	18	18	0.298
GENE_A_16_P58292178	152	16	16	0.319

GENE_A_16_P58276210	175	20	20	0.367
GENE_A_16_P58274843	151	18	18	0.317
GENE_A_16_P58280077	144	16	16	0.302
GENE_A_18_P13856861	156	17	17	0.327
GENE_A_16_P21231716	121	14	14	0.253
GENE_A_16_P21249434	146	13	13	0.306
GENE_A_16_P58285323	153	18	18	0.320
GENE_A_16_P03557432	150	18	18	0.314
GENE_A_16_P03574375	154	18	18	0.322
GENE_A_16_P21249009	152	17	17	0.318
GENE_A_16_P03584626	153	17	17	0.320
GENE_A_18_P22549434	136	16	16	0.285
GENE_A_16_P58260924	153	16	16	0.320
GENE_A_16_P41411506	133	18	18	0.278
GENE_A_16_P21250639	148	19	19	0.310
GENE_A_16_P03567675	148	18	18	0.310
GENE_A_16_P58286582	134	14	14	0.280
GENE_A_16_P03553318	179	20	20	0.374
GENE_A_18_P22586736	149	12	13	0.311
GENE_A_16_P21239949	155	16	16	0.324
GENE_A_16_P58269544	153	19	20	0.320
GENE_A_16_P03591020	144	15	15	0.301
GENE_A_16_P41426463	149	18	18	0.311
GENE_A_16_P03579976	160	20	20	0.334
GENE_A_16_P21265493	145	18	18	0.303
GENE_A_16_P21231455	160	17	17	0.334
GENE_A_16_P21259813	149	20	20	0.311
GENE_A_16_P03597833	160	17	17	0.334
GENE_A_16_P21213933	150	14	14	0.313
GENE_A_18_P22583852	124	14	14	0.259
GENE_A_16_P58266974	167	15	15	0.349
GENE_A_16_P58266343	131	13	14	0.274
GENE_A_16_P58307376	162	19	19	0.338
GENE_A_16_P03592982	169	17	17	0.353
GENE_A_16_P21236941	155	11	11	0.324
GENE_A_18_P13862977	175	18	18	0.365
GENE_A_16_P03553461	162	18	18	0.338
GENE_A_16_P21195101	160	19	19	0.334
GENE_A_16_P21196708	149	13	13	0.311
GENE_A_16_P41435727	152	14	15	0.317
GENE_A_16_P21213067	148	19	19	0.309
GENE_A_16_P21209282	143	17	17	0.298
GENE_A_18_P22484985	153	19	19	0.319
GENE_A_16_P03587787	142	15	15	0.296
GENE_A_16_P21248533	151	15	15	0.315
GENE_A_16_P21220895	157	20	20	0.327

GENE_A_16_P21203485	149	15	15	0.310
GENE_A_16_P03573254	140	18	18	0.292
GENE_A_16_P58274130	155	19	20	0.323
GENE_A_16_P21271886	141	16	16	0.294
GENE_A_16_P41411561	131	14	14	0.273
GENE_A_16_P21238712	181	19	19	0.377
GENE_A_16_P03562240	169	19	19	0.352
GENE_A_16_P21196986	153	17	17	0.318
GENE_A_16_P21208095	167	19	19	0.348
GENE_A_16_P58270931	138	16	16	0.287
GENE_A_16_P58302187	146	15	15	0.304
GENE_A_16_P58283083	171	20	20	0.356
GENE_A_16_P58300029	156	16	17	0.325
GENE_A_16_P58268561	147	16	16	0.306
GENE_A_18_P22505420	147	14	14	0.306
GENE_A_16_P03592425	142	15	15	0.295
GENE_A_16_P21234559	161	16	16	0.335
GENE_A_18_P13936452	158	16	17	0.328
GENE_A_16_P58304574	144	16	17	0.299
GENE_A_16_P03581767	133	14	14	0.276
GENE_A_16_P03551413	145	15	15	0.301
GENE_A_18_P13895970	156	19	19	0.324
GENE_A_16_P21218692	153	15	15	0.318
GENE_A_18_P13918467	142	18	18	0.295
GENE_A_16_P03578247	148	16	16	0.307
GENE_A_16_P03573185	146	16	16	0.303
GENE_A_16_P21246560	139	15	15	0.288
GENE_A_16_P21205440	150	13	13	0.311
GENE_A_16_P58260043	138	12	12	0.286
GENE_A_18_P13872063	143	16	16	0.297
GENE_A_18_P13919417	160	18	18	0.332
GENE_A_16_P58299908	150	16	16	0.311
GENE_A_16_P03573199	159	17	17	0.330
GENE_A_16_P21202169	145	15	15	0.301
GENE_A_18_P22537778	155	13	13	0.321
GENE_A_16_P03577145	135	14	15	0.280
GENE_A_18_P13882243	157	15	15	0.325
GENE_A_16_P58270085	156	17	18	0.323
GENE_A_16_P41408464	150	17	17	0.311
GENE_A_16_P03594388	141	15	15	0.292
GENE_A_16_P03592557	166	18	18	0.344
GENE_A_16_P58295489	156	13	13	0.323
GENE_A_16_P21249584	143	14	14	0.296
GENE_A_16_P41426885	150	15	15	0.310
GENE_A_16_P41409150	136	17	17	0.281
GENE_A_16_P21236036	146	21	21	0.302

GENE_A_16_P03595232	157	13	13	0.325
GENE_A_16_P21192916	155	16	16	0.321
GENE_A_16_P21255426	151	16	16	0.312
GENE_A_16_P03597529	150	16	16	0.310
GENE_A_16_P41417882	147	15	15	0.304
GENE_A_16_P21204578	163	18	18	0.337
GENE_A_16_P03587122	144	15	15	0.298
GENE_A_16_P58283745	142	15	15	0.293
GENE_A_16_P58292266	150	11	11	0.310
GENE_A_18_P13873179	151	20	20	0.312
GENE_A_16_P21257464	162	19	19	0.335
GENE_A_16_P21224367	159	16	16	0.328
GENE_A_16_P58265387	152	17	19	0.314
GENE_A_16_P21252143	157	17	17	0.324
GENE_A_16_P03597810	156	16	16	0.322
GENE_A_16_P21249484	134	12	12	0.277
GENE_A_16_P21231383	152	16	16	0.314
GENE_A_18_P13871055	146	15	15	0.301
GENE_A_16_P58287151	148	13	13	0.306
GENE_A_16_P58307022	165	18	19	0.341
GENE_A_16_P58283395	163	19	19	0.336
GENE_A_16_P21262611	161	9	9	0.332
GENE_A_16_P03568279	164	17	17	0.338
GENE_A_16_P21196645	152	15	16	0.314
GENE_A_16_P58307760	131	11	11	0.270
GENE_A_16_P34766157	148	15	15	0.305
GENE_A_16_P03573114	151	17	17	0.311
GENE_A_16_P03568846	158	16	16	0.326
GENE_A_16_P03579337	155	16	16	0.320
GENE_A_16_P03553246	166	18	18	0.342
GENE_A_16_P58283452	146	17	17	0.301
GENE_A_16_P58275244	148	17	17	0.305
GENE_A_16_P58308457	158	18	18	0.326
GENE_A_16_P21259602	136	14	14	0.280
GENE_A_16_P41392708	146	18	18	0.301
GENE_A_16_P58285212	151	16	16	0.311
GENE_A_18_P13922210	147	16	16	0.303
GENE_A_18_P13923197	143	15	15	0.294
GENE_A_16_P21248190	160	14	15	0.329
GENE_A_18_P13922240	162	17	18	0.333
GENE_A_16_P03554067	138	17	17	0.284
GENE_A_16_P58303896	165	17	18	0.339
GENE_A_16_P03557359	170	20	20	0.350
GENE_A_16_P41440102	144	16	16	0.296
GENE_A_16_P21224784	171	18	18	0.352
GENE_A_16_P41461399	132	16	16	0.271

GENE_A_18_P22567908	155	17	17	0.319
GENE_A_16_P21200039	148	14	14	0.304
GENE_A_18_P22572758	152	18	18	0.312
GENE_A_16_P21211887	149	14	14	0.306
GENE_A_16_P41417244	157	15	15	0.322
GENE_A_16_P21268345	170	14	14	0.349
GENE_A_16_P21266469	179	19	19	0.368
GENE_A_16_P21210881	158	17	17	0.324
GENE_A_16_P03588307	138	15	15	0.283
GENE_A_16_P03589184	154	16	16	0.316
GENE_A_18_P13897733	153	18	18	0.314
GENE_A_16_P58265419	190	21	21	0.390
GENE_A_16_P41453491	154	16	16	0.316
GENE_A_18_P22511989	152	18	18	0.312
GENE_A_16_P03562714	156	17	18	0.320
GENE_A_16_P03579988	162	18	18	0.332
GENE_A_16_P58300874	151	17	17	0.310
GENE_A_16_P21245073	169	16	16	0.347
GENE_A_18_P22556339	151	19	20	0.310
GENE_A_16_P03588533	149	15	15	0.306
GENE_A_18_P13929328	139	11	11	0.285
GENE_A_18_P22552496	164	18	18	0.336
GENE_A_16_P58284149	142	16	16	0.291
GENE_A_16_P58291310	133	11	11	0.273
GENE_A_16_P21256714	175	21	21	0.359
GENE_A_16_P21222208	165	16	16	0.338
GENE_A_16_P21227902	148	17	17	0.303
GENE_A_16_P58294156	173	17	17	0.354
GENE_A_16_P41400991	140	16	16	0.287
GENE_A_16_P03590210	155	11	11	0.317
GENE_A_16_P58268489	133	16	16	0.272
GENE_A_16_P21208535	131	16	16	0.268
GENE_A_16_P58288574	162	15	15	0.332
GENE_A_16_P58263925	141	16	16	0.289
GENE_A_18_P22561004	146	17	17	0.299
GENE_A_16_P58276781	160	18	18	0.327
GENE_A_16_P03569746	136	13	14	0.278
GENE_A_16_P21212112	136	12	12	0.278
GENE_A_16_P21226133	159	17	17	0.325
GENE_A_16_P21226149	144	17	17	0.294
GENE_A_16_P58294661	160	18	18	0.327
GENE_A_16_P58285334	155	19	19	0.317
GENE_A_16_P41424072	177	21	21	0.362
GENE_A_16_P58268542	138	16	16	0.282
GENE_A_16_P21206345	153	16	17	0.313
GENE_A_16_P41463681	147	17	17	0.300

GENE_A_16_P58293989	155	14	14	0.317
GENE_A_18_P13899427	163	19	19	0.333
GENE_A_16_P03561858	127	15	15	0.259
GENE_A_16_P58259865	150	15	15	0.306
GENE_A_16_P03596340	164	14	14	0.335
GENE_A_16_P03557092	160	18	18	0.327
GENE_A_16_P21238976	147	16	16	0.300
GENE_A_16_P03556117	146	15	15	0.298
GENE_A_18_P13869802	168	14	14	0.343
GENE_A_16_P03562953	155	15	15	0.316
GENE_A_16_P41405595	137	14	15	0.279
GENE_A_16_P21255357	153	16	16	0.312
GENE_A_16_P58260613	154	16	16	0.314
GENE_A_16_P58288342	162	19	19	0.330
GENE_A_16_P21232539	165	18	18	0.336
GENE_A_16_P58291186	138	15	15	0.281
GENE_A_16_P58259840	148	15	15	0.302
GENE_A_18_P22552809	154	18	18	0.314
GENE_A_18_P22525949	159	16	16	0.324
GENE_A_16_P21245871	146	17	17	0.297
GENE_A_16_P58286709	137	14	15	0.279
GENE_A_16_P21205044	158	17	17	0.322
GENE_A_16_P58285439	148	16	16	0.301
GENE_A_16_P58260194	157	19	19	0.320
GENE_A_16_P21227116	152	18	18	0.310
GENE_A_18_P13918630	157	16	16	0.320
GENE_A_16_P41394445	152	19	19	0.310
GENE_A_16_P58280337	136	18	18	0.277
GENE_A_16_P03572717	155	17	17	0.316
GENE_A_16_P03572791	171	17	18	0.348
GENE_A_16_P21210414	163	16	16	0.332
GENE_A_16_P21223059	142	14	14	0.289
GENE_A_16_P21220265	166	20	20	0.338
GENE_A_16_P21246056	142	13	13	0.289
GENE_A_18_P13885118	156	17	17	0.317
GENE_A_18_P13911293	160	17	17	0.326
GENE_A_18_P13858484	169	19	19	0.344
GENE_A_18_P13927762	153	12	12	0.311
GENE_A_16_P41414828	138	14	14	0.281
GENE_A_16_P58273667	153	11	12	0.311
GENE_A_16_P41395035	194	19	20	0.395
GENE_A_16_P03583076	164	15	15	0.334
GENE_A_16_P21200994	146	15	15	0.297
GENE_A_16_P21221515	145	16	16	0.295
GENE_A_16_P41448311	163	18	18	0.331
GENE_A_16_P58286332	139	15	15	0.283

GENE_A_18_P22560895	137	13	13	0.278
GENE_A_16_P58300844	136	14	14	0.276
GENE_A_16_P58302258	159	15	16	0.323
GENE_A_16_P21201158	154	16	16	0.313
GENE_A_16_P03564473	141	16	16	0.286
GENE_A_18_P22511969	148	14	14	0.300
GENE_A_16_P41421209	157	14	15	0.319
GENE_A_16_P21268568	136	13	14	0.276
GENE_A_16_P58267179	165	18	18	0.335
GENE_A_16_P03582356	145	14	15	0.294
GENE_A_16_P21220349	154	18	18	0.312
GENE_A_18_P13888988	170	21	21	0.345
GENE_A_16_P58266963	141	14	14	0.286
GENE_A_18_P13923730	153	12	12	0.310
GENE_A_16_P03583588	141	19	19	0.286
GENE_A_16_P41414466	171	16	17	0.347
GENE_A_16_P03561790	160	17	17	0.324
GENE_A_18_P13933770	151	14	14	0.306
GENE_A_16_P58270761	161	18	18	0.326
GENE_A_16_P03582613	157	18	18	0.318
GENE_A_16_P21229150	140	16	16	0.284
GENE_A_16_P03562438	142	16	16	0.288
GENE_A_16_P58294934	164	15	16	0.332
GENE_A_16_P41397890	166	17	17	0.336
GENE_A_16_P58288177	146	18	19	0.296
GENE_A_16_P03585634	155	14	14	0.314
GENE_A_16_P03592181	164	14	16	0.332
GENE_A_16_P41390066	127	15	15	0.257
GENE_A_18_P22592802	136	15	15	0.275
GENE_A_18_P22601092	143	13	13	0.290
GENE_A_16_P21259812	155	15	15	0.314
GENE_A_16_P58267793	147	18	18	0.298
GENE_A_16_P34770019	155	16	16	0.314
GENE_A_16_P03578908	159	17	17	0.322
GENE_A_16_P03593300	147	13	13	0.297
GENE_A_16_P21229495	138	18	18	0.279
GENE_A_16_P58305300	143	15	15	0.289
GENE_A_16_P41458241	166	10	10	0.336
GENE_A_16_P03569620	159	19	20	0.322
GENE_A_16_P41423266	155	17	17	0.313
GENE_A_16_P03569481	154	15	15	0.311
GENE_A_16_P21263712	181	21	21	0.366
GENE_A_16_P21203805	163	18	19	0.330
GENE_A_16_P21202820	158	17	17	0.319
GENE_A_16_P58262049	159	13	13	0.321
GENE_A_16_P58271332	155	15	15	0.313

GENE_A_16_P03577823	134	14	14	0.271
GENE_A_18_P22590859	149	17	17	0.301
GENE_A_16_P41388824	146	16	16	0.295
GENE_A_16_P58298573	129	14	15	0.261
GENE_A_16_P41417233	141	17	17	0.285
GENE_A_16_P41432046	154	13	13	0.311
GENE_A_18_P13938709	177	20	20	0.357
GENE_A_16_P58273302	121	12	12	0.244
GENE_A_16_P03567264	125	15	15	0.252
GENE_A_16_P21249626	130	13	13	0.262
GENE_A_16_P03550816	160	15	16	0.323
GENE_A_16_P21254862	155	17	17	0.313
GENE_A_16_P41435038	149	18	18	0.301
GENE_A_16_P21205150	144	13	13	0.290
GENE_A_16_P21203046	126	9	9	0.254
GENE_A_16_P21213697	129	16	16	0.260
GENE_A_16_P21258498	155	19	20	0.313
GENE_A_16_P03556044	159	16	16	0.321
GENE_A_16_P58275405	172	18	18	0.347
GENE_A_16_P58297819	137	13	13	0.276
GENE_A_16_P41428775	153	17	17	0.308
GENE_A_16_P21212090	157	20	20	0.316
GENE_A_16_P41448573	161	15	16	0.324
GENE_A_16_P21257322	155	17	17	0.312
GENE_A_16_P34822335	162	19	19	0.326
GENE_A_18_P13909216	154	10	10	0.310
GENE_A_16_P21214718	150	15	15	0.302
GENE_A_16_P41403163	165	17	17	0.332
GENE_A_18_P13901120	145	18	18	0.292
GENE_A_16_P21210326	152	18	18	0.306
GENE_A_16_P21210210	150	16	16	0.302
GENE_A_18_P13864015	132	16	16	0.266
GENE_A_16_P03593511	160	18	18	0.322
GENE_A_16_P58268135	149	16	16	0.300
GENE_A_16_P58282648	155	17	17	0.312
GENE_A_16_P41423753	153	15	15	0.308
GENE_A_16_P21239578	146	15	15	0.294
GENE_A_16_P58286134	137	16	16	0.276
GENE_A_16_P58263939	160	16	16	0.322
GENE_A_16_P21212352	162	16	16	0.326
GENE_A_18_P13860015	171	18	18	0.344
GENE_A_16_P58273718	150	16	16	0.302
GENE_A_16_P21241228	172	24	24	0.346
GENE_A_16_P58266833	171	17	17	0.344
GENE_A_16_P21232208	146	13	13	0.294
GENE_A_16_P21260875	162	19	19	0.326

GENE_A_18_P22598923	149	14	14	0.299
GENE_A_16_P41444059	161	18	18	0.324
GENE_A_18_P13868554	138	13	13	0.277
GENE_A_18_P13898247	131	14	14	0.263
GENE_A_16_P41385902	148	16	17	0.297
GENE_A_16_P58309501	138	16	16	0.277
GENE_A_18_P13907362	142	11	11	0.285
GENE_A_16_P21247680	137	15	15	0.275
GENE_A_16_P21227222	143	15	15	0.287
GENE_A_16_P58285233	153	12	12	0.307
GENE_A_16_P58297960	134	13	13	0.269
GENE_A_16_P41455641	135	15	15	0.271
GENE_A_16_P21249896	143	15	15	0.287
GENE_A_18_P22530307	147	14	14	0.295
GENE_A_16_P21193286	136	14	14	0.273
GENE_A_16_P21259212	141	14	14	0.283
GENE_A_16_P58286564	152	14	14	0.305
GENE_A_18_P13936962	163	20	20	0.327
GENE_A_16_P41426372	159	17	17	0.319
GENE_A_16_P21215924	148	15	15	0.297
GENE_A_16_P58304209	134	9	9	0.269
GENE_A_18_P13940598	159	19	19	0.319
GENE_A_16_P41454781	128	14	14	0.257
GENE_A_16_P58269413	119	13	13	0.239
GENE_A_16_P03556337	148	15	15	0.297
GENE_A_16_P58271303	158	19	19	0.317
GENE_A_16_P21244272	148	14	14	0.296
GENE_A_16_P21261236	164	18	18	0.328
GENE_A_16_P58272213	167	19	19	0.334
GENE_A_16_P58296847	168	15	15	0.336
GENE_A_16_P34811062	150	20	20	0.300
GENE_A_16_P03578770	139	15	15	0.278
GENE_A_18_P13865258	154	15	16	0.308
GENE_A_16_P58301745	154	15	16	0.308
GENE_A_16_P21226580	141	11	11	0.282
GENE_A_16_P21268969	156	19	19	0.312
GENE_A_16_P21259376	148	18	18	0.296
GENE_A_16_P21259910	148	16	16	0.296
GENE_A_16_P58305678	154	16	16	0.308
GENE_A_14_P124831	158	18	18	0.316
GENE_A_16_P58288846	154	17	17	0.308
GENE_A_16_P21207449	148	14	14	0.296
GENE_A_16_P03590564	160	16	16	0.320
GENE_A_16_P21239969	154	18	18	0.308
GENE_A_16_P21240295	153	16	16	0.306
GENE_A_16_P21222250	147	15	15	0.294

GENE_A_16_P21210573	141	14	14	0.282
GENE_A_16_P58290667	134	11	12	0.268
GENE_A_16_P58304724	145	14	15	0.290
GENE_A_16_P03598044	140	14	14	0.280
GENE_A_16_P58301605	161	13	13	0.322
GENE_A_18_P13867460	129	14	14	0.258
GENE_A_16_P41457647	150	18	18	0.300
GENE_A_16_P58283421	162	13	14	0.323
GENE_A_16_P21246289	148	18	18	0.296
GENE_A_16_P41454091	139	14	14	0.278
GENE_A_16_P21233929	134	14	14	0.268
GENE_A_16_P58306647	166	19	19	0.331
GENE_A_16_P41414059	151	13	14	0.301
GENE_A_16_P58260496	139	17	17	0.277
GENE_A_18_P13868369	140	17	17	0.279
GENE_A_16_P58306487	144	18	18	0.287
GENE_A_16_P58297877	125	13	13	0.249
GENE_A_16_P21219790	147	15	15	0.293
GENE_A_16_P58281889	141	16	16	0.281
GENE_A_16_P58266037	173	20	20	0.345
GENE_A_16_P21209589	147	16	16	0.293
GENE_A_16_P21199839	143	17	17	0.285
GENE_A_16_P41409635	169	14	14	0.337
GENE_A_18_P13905483	144	13	13	0.287
GENE_A_16_P41416319	143	16	16	0.285
GENE_A_16_P21244977	150	17	17	0.299
GENE_A_16_P58292252	151	16	16	0.301
GENE_A_18_P13944040	151	16	16	0.301
GENE_A_16_P58300566	160	18	18	0.319
GENE_A_16_P03579601	138	17	17	0.275
GENE_A_18_P22538585	150	17	17	0.298
GENE_A_16_P58262660	158	17	17	0.314
GENE_A_16_P21197726	145	16	16	0.288
GENE_A_18_P13908856	155	14	14	0.308
GENE_A_16_P41455748	153	17	17	0.304
GENE_A_16_P58278070	144	16	16	0.286
GENE_A_16_P21232292	149	15	16	0.296
GENE_A_16_P03580465	157	16	18	0.312
GENE_A_16_P03581040	148	16	16	0.294
GENE_A_16_P03580828	149	16	16	0.296
GENE_A_16_P03593910	144	14	14	0.286
GENE_A_16_P03589664	164	17	17	0.326
GENE_A_16_P58305227	149	17	17	0.296
GENE_A_18_P22543939	137	15	15	0.272
GENE_A_16_P41404104	144	14	14	0.286
GENE_A_16_P21255709	151	15	15	0.300

GENE_A_16_P58276755	129	15	15	0.256
GENE_A_16_P58292923	157	13	14	0.312
GENE_A_16_P03559584	147	12	12	0.292
GENE_A_16_P21228706	159	16	17	0.315
GENE_A_16_P03571743	155	16	16	0.307
GENE_A_16_P03592819	133	11	11	0.264
GENE_A_16_P41410941	147	17	17	0.292
GENE_A_16_P58293277	146	16	16	0.290
GENE_A_14_P134351	154	17	18	0.305
GENE_A_16_P21235805	150	13	13	0.297
GENE_A_16_P58290674	131	12	13	0.260
GENE_A_16_P41413774	147	12	12	0.291
GENE_A_16_P21217721	159	19	19	0.315
GENE_A_18_P13929437	158	14	14	0.313
GENE_A_16_P03573565	150	15	15	0.297
GENE_A_16_P21239440	151	17	17	0.299
GENE_A_16_P41417704	149	16	16	0.295
GENE_A_16_P03557312	169	15	15	0.335
GENE_A_16_P58307686	157	17	17	0.311
GENE_A_16_P58267165	141	15	16	0.279
GENE_A_16_P03582856	142	17	17	0.281
GENE_A_16_P21193963	139	14	14	0.275
GENE_A_16_P58283438	129	13	13	0.255
GENE_A_16_P21193182	151	15	15	0.299
GENE_A_16_P58280620	156	16	16	0.309
GENE_A_16_P58290105	147	17	17	0.291
GENE_A_16_P21254427	148	15	15	0.293
GENE_A_16_P58266806	148	14	14	0.293
GENE_A_16_P03589053	138	12	12	0.273
GENE_A_16_P58269487	152	16	16	0.301
GENE_A_18_P13919782	141	16	16	0.279
GENE_A_18_P13898009	161	15	15	0.318
GENE_A_16_P21238597	151	16	16	0.299
GENE_A_16_P21251689	161	14	14	0.318
GENE_A_16_P21240133	158	13	14	0.312
GENE_A_16_P58296259	141	13	13	0.279
GENE_A_16_P21204531	138	13	13	0.273
GENE_A_16_P41440528	144	14	14	0.284
GENE_A_16_P21223468	146	14	14	0.288
GENE_A_16_P21207646	137	14	14	0.271
GENE_A_16_P41402317	158	18	18	0.312
GENE_A_16_P21201198	146	16	16	0.288
GENE_A_16_P34780136	155	16	16	0.306
GENE_A_16_P21243474	146	15	15	0.288
GENE_A_16_P21231407	156	16	16	0.308
GENE_A_18_P22545057	140	16	17	0.276

GENE_A_16_P21219452	147	16	16	0.290
GENE_A_18_P22582929	138	15	16	0.272
GENE_A_16_P03589854	183	19	19	0.361
GENE_A_16_P21232309	171	18	18	0.337
GENE_A_16_P21265272	155	16	16	0.306
GENE_A_16_P58268764	133	16	16	0.262
GENE_A_16_P21219672	136	14	14	0.268
GENE_A_18_P13945051	152	16	16	0.300
GENE_A_16_P58286043	158	13	13	0.312
GENE_A_16_P41443723	138	13	13	0.272
GENE_A_16_P21217921	150	15	15	0.296
GENE_A_16_P58277212	152	17	17	0.300
GENE_A_16_P21263640	138	14	14	0.272
GENE_A_16_P03591535	139	13	13	0.274
GENE_A_16_P03592878	140	13	15	0.276
GENE_A_16_P21248095	143	13	14	0.282
GENE_A_16_P21268232	134	15	16	0.264
GENE_A_18_P13911306	161	17	17	0.317
GENE_A_16_P21240086	156	15	16	0.307
GENE_A_16_P58264679	140	14	14	0.276
GENE_A_16_P58296149	159	14	14	0.313
GENE_A_18_P22517117	135	17	17	0.266
GENE_A_16_P21250311	141	16	16	0.277
GENE_A_16_P21216595	141	11	11	0.277
GENE_A_16_P03556575	148	14	14	0.291
GENE_A_18_P13887916	154	18	18	0.303
GENE_A_16_P03585769	141	10	11	0.277
GENE_A_16_P03575554	135	9	9	0.265
GENE_A_16_P58291584	169	16	16	0.332
GENE_A_16_P58297914	144	16	17	0.283
GENE_A_16_P41447293	137	15	15	0.269
GENE_A_16_P21254315	141	15	15	0.277
GENE_A_18_P13882684	139	17	17	0.273
GENE_A_16_P21199617	153	17	17	0.301
GENE_A_16_P58290693	156	15	15	0.307
GENE_A_16_P41432912	161	14	14	0.316
GENE_A_16_P41428892	142	15	17	0.279
GENE_A_18_P22573846	149	12	12	0.293
GENE_A_16_P58265131	160	20	20	0.314
GENE_A_16_P58301030	163	14	14	0.320
GENE_A_18_P22599612	149	17	17	0.293
GENE_A_16_P21244673	150	13	13	0.295
GENE_A_16_P21232493	138	16	16	0.271
GENE_A_16_P58268073	132	14	15	0.259
GENE_A_16_P03579935	144	17	17	0.283
GENE_A_16_P21229474	160	17	18	0.314

GENE_A_16_P21246010	133	13	13	0.261
GENE_A_18_P13874655	156	14	14	0.306
GENE_A_16_P41441835	138	13	13	0.271
GENE_A_16_P21251232	161	20	21	0.316
GENE_A_16_P03597094	170	14	14	0.333
GENE_A_16_P03572861	141	14	14	0.276
GENE_A_16_P41389199	156	16	16	0.306
GENE_A_16_P03583423	138	13	13	0.271
GENE_A_16_P58276096	140	15	15	0.274
GENE_A_16_P41418740	149	16	16	0.292
GENE_A_18_P13901192	165	14	14	0.323
GENE_A_16_P58269901	154	17	17	0.302
GENE_A_18_P22505546	139	14	14	0.272
GENE_A_16_P58273337	157	14	14	0.308
GENE_A_18_P13917396	149	16	16	0.292
GENE_A_16_P58280666	147	13	13	0.288
GENE_A_16_P21195911	168	16	16	0.329
GENE_A_16_P03551673	133	14	14	0.261
GENE_A_16_P41392780	146	18	18	0.286
GENE_A_16_P21206177	140	14	14	0.274
GENE_A_16_P41431703	141	13	13	0.276
GENE_A_18_P22592209	146	15	15	0.286
GENE_A_16_P03570992	139	13	16	0.272
GENE_A_16_P58282425	138	15	15	0.270
GENE_A_16_P58283942	150	18	18	0.293
GENE_A_16_P41421755	168	15	15	0.329
GENE_A_16_P58261043	146	13	13	0.286
GENE_A_16_P03584721	165	13	13	0.323
GENE_A_18_P22555505	144	16	17	0.282
GENE_A_16_P58264078	151	14	14	0.295
GENE_A_16_P03565582	135	11	11	0.264
GENE_A_16_P41417111	148	15	15	0.289
GENE_A_16_P41448544	157	14	14	0.307
GENE_A_16_P58280020	160	16	16	0.313
GENE_A_16_P03557162	157	18	18	0.307
GENE_A_18_P13899689	151	14	14	0.295
GENE_A_16_P21249418	137	16	16	0.268
GENE_A_16_P21204728	170	19	19	0.332
GENE_A_18_P13872087	154	14	14	0.301
GENE_A_18_P13916942	143	15	15	0.279
GENE_A_16_P21199375	174	19	19	0.340
GENE_A_16_P41427926	163	17	17	0.318
GENE_A_18_P13867997	156	16	16	0.305
GENE_A_16_P58285238	144	13	13	0.281
GENE_A_16_P21216185	161	15	15	0.314
GENE_A_16_P58274840	140	15	15	0.273

GENE_A_16_P03586040	147	14	14	0.287
GENE_A_18_P13903395	151	15	15	0.294
GENE_A_16_P58298881	141	14	14	0.275
GENE_A_18_P13914051	135	17	17	0.263
GENE_A_16_P21269695	138	13	14	0.269
GENE_A_16_P41432507	146	17	17	0.285
GENE_A_16_P03551783	141	18	18	0.275
GENE_A_16_P03589677	179	16	16	0.349
GENE_A_16_P03592954	132	12	12	0.257
GENE_A_16_P21212483	154	14	14	0.300
GENE_A_16_P03596873	131	11	11	0.255
GENE_A_16_P58292133	141	14	14	0.275
GENE_A_16_P21214522	157	17	17	0.306
GENE_A_16_P21194377	142	13	13	0.277
GENE_A_16_P58268701	147	13	13	0.286
GENE_A_18_P22533023	150	16	17	0.292
GENE_A_16_P03553733	152	17	18	0.296
GENE_A_16_P03550799	158	17	17	0.307
GENE_A_16_P21209817	152	18	18	0.296
GENE_A_16_P03591818	152	15	15	0.296
GENE_A_16_P41447531	156	19	19	0.303
GENE_A_16_P21210103	146	16	16	0.284
GENE_A_16_P03582898	160	19	19	0.311
GENE_A_18_P13937520	145	12	13	0.282
GENE_A_16_P03590834	144	11	11	0.280
GENE_A_16_P21240430	140	15	15	0.272
GENE_A_16_P03585075	148	19	19	0.287
GENE_A_16_P58264304	146	16	16	0.284
GENE_A_18_P13875175	152	12	12	0.295
GENE_A_16_P58289987	153	15	15	0.297
GENE_A_16_P21225366	149	16	16	0.289
GENE_A_18_P13861054	154	13	13	0.299
GENE_A_16_P21211678	143	13	13	0.278
GENE_A_16_P58294441	144	15	16	0.279
GENE_A_16_P03585204	148	12	12	0.287
GENE_A_16_P03594231	136	12	12	0.264
GENE_A_16_P21239755	170	19	20	0.330
GENE_A_16_P58287611	147	14	14	0.285
GENE_A_16_P21237523	151	17	17	0.293
GENE_A_16_P41396828	147	15	15	0.285
GENE_A_16_P21194716	175	21	21	0.340
GENE_A_16_P21247414	154	15	15	0.299
GENE_A_16_P58284383	150	10	10	0.291
GENE_A_18_P13893405	135	15	15	0.262
GENE_A_18_P13904320	150	17	17	0.291
GENE_A_16_P03582857	140	14	14	0.271

GENE_A_16_P21210675	152	15	15	0.295
GENE_A_16_P58273356	136	15	15	0.264
GENE_A_16_P41394982	176	21	21	0.341
GENE_A_16_P41450343	154	17	17	0.298
GENE_A_16_P58295513	142	15	15	0.275
GENE_A_16_P58277619	157	19	19	0.304
GENE_A_18_P13861839	166	19	19	0.322
GENE_A_16_P34784705	176	18	18	0.341
GENE_A_16_P41440115	137	11	11	0.265
GENE_A_16_P03584250	156	15	15	0.302
GENE_A_14_P125555	143	13	14	0.277
GENE_A_16_P21214639	146	15	15	0.283
GENE_A_18_P13926906	164	19	19	0.318
GENE_A_16_P58262449	143	12	12	0.277
GENE_A_16_P41453930	152	19	19	0.294
GENE_A_16_P21224637	160	15	15	0.310
GENE_A_18_P13919794	179	19	19	0.347
GENE_A_18_P13886616	162	14	14	0.314
GENE_A_18_P13903378	169	16	16	0.327
GENE_A_18_P13896832	151	15	15	0.292
GENE_A_16_P58263278	172	17	17	0.333
GENE_A_16_P21217635	137	15	15	0.265
GENE_A_16_P41395738	174	19	19	0.337
GENE_A_16_P21216843	123	14	14	0.238
GENE_A_14_P128267	153	16	16	0.296
GENE_A_18_P13933883	132	11	11	0.255
GENE_A_16_P03558787	157	13	13	0.304
GENE_A_16_P58260417	162	17	18	0.313
GENE_A_16_P21224162	158	21	21	0.306
GENE_A_18_P13862930	165	18	19	0.319
GENE_A_18_P13905290	172	15	15	0.333
GENE_A_16_P03580489	154	16	16	0.298
GENE_A_16_P58301612	147	16	16	0.284
GENE_A_16_P58309668	158	16	16	0.305
GENE_A_18_P13865697	139	12	12	0.269
GENE_A_16_P41414501	160	14	14	0.309
GENE_A_16_P21259727	168	16	16	0.325
GENE_A_16_P03552654	177	17	17	0.342
GENE_A_16_P03555994	142	13	13	0.274
GENE_A_16_P41429128	156	13	13	0.301
GENE_A_16_P58267700	149	11	11	0.288
GENE_A_16_P03585317	164	16	16	0.316
GENE_A_16_P58272106	143	14	14	0.276
GENE_A_16_P21200869	152	14	14	0.293
GENE_A_16_P58276714	145	13	13	0.280
GENE_A_16_P58302614	149	16	16	0.287

GENE_A_16_P58306485	138	13	13	0.266
GENE_A_16_P58277828	144	14	15	0.278
GENE_A_16_P21251297	157	15	15	0.303
GENE_A_16_P21241290	165	15	15	0.318
GENE_A_16_P58297097	168	15	16	0.324
GENE_A_18_P13863005	147	13	13	0.283
GENE_A_16_P58296144	135	14	14	0.260
GENE_A_16_P41391627	159	16	16	0.307
GENE_A_16_P21228914	158	18	18	0.305
GENE_A_16_P58278377	164	16	16	0.316
GENE_A_16_P21200689	172	16	16	0.331
GENE_A_18_P13931428	145	12	12	0.279
GENE_A_16_P03553613	126	10	10	0.243
GENE_A_16_P21235755	166	19	19	0.320
GENE_A_16_P58275444	144	17	17	0.277
GENE_A_16_P21212820	143	14	14	0.275
GENE_A_16_P58301531	165	18	19	0.318
GENE_A_16_P21260943	162	16	16	0.312
GENE_A_16_P21209122	163	17	17	0.314
GENE_A_16_P21215768	147	16	16	0.283
GENE_A_16_P58294075	148	15	15	0.285
GENE_A_16_P03577442	148	16	16	0.285
GENE_A_16_P58306377	162	18	18	0.312
GENE_A_16_P03590184	145	13	13	0.279
GENE_A_16_P58261365	165	17	17	0.318
GENE_A_18_P13862827	170	17	17	0.327
GENE_A_16_P58291798	143	16	16	0.275
GENE_A_16_P21193389	125	14	14	0.240
GENE_A_16_P41410572	145	13	14	0.279
GENE_A_18_P22573453	148	14	14	0.285
GENE_A_16_P21255954	134	13	14	0.258
GENE_A_16_P21193612	148	20	20	0.285
GENE_A_16_P34802886	152	16	16	0.292
GENE_A_16_P41424587	134	15	15	0.258
GENE_A_16_P58303439	168	14	15	0.323
GENE_A_16_P41409394	154	17	17	0.296
GENE_A_16_P21219136	169	17	17	0.325
GENE_A_16_P41428149	140	15	15	0.269
GENE_A_16_P58283081	150	13	13	0.288
GENE_A_18_P13858374	166	16	16	0.319
GENE_A_16_P03588785	155	16	16	0.298
GENE_A_16_P03550683	155	19	19	0.298
GENE_A_18_P22493199	150	14	14	0.288
GENE_A_16_P41407114	145	13	13	0.278
GENE_A_18_P22596354	147	10	10	0.282
GENE_A_16_P21231691	174	17	17	0.334

GENE_A_16_P58291846	146	13	13	0.280
GENE_A_16_P58298789	159	16	16	0.305
GENE_A_16_P03588246	125	9	9	0.240
GENE_A_16_P41396819	153	17	17	0.294
GENE_A_16_P21218896	140	13	13	0.269
GENE_A_16_P58305784	165	17	17	0.317
GENE_A_16_P21196413	147	11	11	0.282
GENE_A_18_P13911899	141	12	12	0.270
GENE_A_16_P21256176	135	14	14	0.259
GENE_A_16_P41392791	171	19	19	0.328
GENE_A_16_P03595706	130	11	11	0.249
GENE_A_16_P21241857	146	14	14	0.280
GENE_A_16_P58298542	139	15	16	0.267
GENE_A_16_P34777489	190	19	19	0.364
GENE_A_16_P21262584	147	13	13	0.282
GENE_A_16_P58299504	143	13	13	0.274
GENE_A_18_P13889013	153	16	16	0.293
GENE_A_16_P58297485	150	17	17	0.288
GENE_A_16_P21192926	141	15	15	0.270
GENE_A_18_P22592927	153	15	15	0.293
GENE_A_18_P22542297	144	13	13	0.276
GENE_A_16_P03598013	155	16	16	0.297
GENE_A_16_P41401037	121	13	13	0.232
GENE_A_16_P58268635	162	18	18	0.310
GENE_A_16_P41458542	146	14	14	0.280
GENE_A_16_P21234931	170	17	17	0.326
GENE_A_16_P58301069	169	18	18	0.324
GENE_A_16_P58266370	147	16	17	0.282
GENE_A_16_P58309041	150	13	13	0.287
GENE_A_16_P41419087	137	13	13	0.262
GENE_A_16_P21256409	136	13	13	0.260
GENE_A_16_P41415011	140	16	16	0.268
GENE_A_16_P58281499	148	14	14	0.283
GENE_A_16_P03576133	147	14	14	0.281
GENE_A_16_P58299616	156	13	13	0.299
GENE_A_16_P58270532	155	17	17	0.297
GENE_A_16_P21241761	131	9	9	0.251
GENE_A_18_P13935234	149	13	15	0.285
GENE_A_16_P03560039	155	12	12	0.296
GENE_A_16_P21217631	159	15	15	0.304
GENE_A_16_P21241002	145	13	13	0.277
GENE_A_16_P21245900	153	15	15	0.293
GENE_A_18_P22585995	146	15	16	0.279
GENE_A_16_P21218105	148	17	17	0.283
GENE_A_16_P58287739	150	13	13	0.287
GENE_A_16_P03574640	146	17	17	0.279

GENE_A_16_P03594942	160	17	17	0.306
GENE_A_16_P03575213	164	17	17	0.313
GENE_A_18_P13869718	155	13	13	0.296
GENE_A_16_P21215137	137	13	14	0.262
GENE_A_16_P21253630	147	17	17	0.281
GENE_A_18_P22595596	158	16	16	0.302
GENE_A_16_P41439555	150	16	17	0.286
GENE_A_16_P03591471	157	17	17	0.300
GENE_A_16_P03572706	160	20	20	0.305
GENE_A_16_P21206998	149	16	16	0.284
GENE_A_16_P21208699	130	11	11	0.248
GENE_A_16_P21245387	129	12	12	0.246
GENE_A_16_P03580842	138	13	13	0.263
GENE_A_16_P21268886	161	20	20	0.307
GENE_A_16_P21231055	145	14	14	0.277
GENE_A_16_P41440952	166	14	14	0.317
GENE_A_16_P41415668	152	15	16	0.290
GENE_A_16_P21198849	148	14	15	0.282
GENE_A_16_P58263153	147	14	14	0.280
GENE_A_18_P22527134	153	17	17	0.292
GENE_A_16_P58300255	139	14	14	0.265
GENE_A_16_P03582849	141	11	11	0.269
GENE_A_16_P41409505	161	13	14	0.307
GENE_A_16_P03580838	140	14	14	0.267
GENE_A_16_P21226277	151	13	13	0.288
GENE_A_16_P21207666	170	17	17	0.324
GENE_A_16_P41437871	147	14	14	0.280
GENE_A_16_P21235033	172	15	15	0.328
GENE_A_16_P58304314	157	11	11	0.299
GENE_A_18_P13861046	159	16	16	0.303
GENE_A_18_P13905840	154	17	17	0.293
GENE_A_16_P21228095	152	14	14	0.289
GENE_A_16_P21235205	170	20	20	0.324
GENE_A_16_P03583080	151	14	15	0.287
GENE_A_16_P41391332	132	14	15	0.251
GENE_A_16_P41437431	138	18	18	0.263
GENE_A_16_P58263955	159	16	16	0.303
GENE_A_16_P03593500	149	16	16	0.284
GENE_A_16_P58274449	142	15	16	0.270
GENE_A_18_P13937933	173	20	20	0.329
GENE_A_16_P21272684	145	15	15	0.276
GENE_A_16_P34811436	136	11	11	0.259
GENE_A_18_P13894235	160	18	18	0.304
GENE_A_16_P41410907	157	15	15	0.299
GENE_A_16_P58267591	132	12	13	0.251
GENE_A_16_P41448160	148	15	16	0.281

GENE_A_16_P58272825	143	12	12	0.272
GENE_A_16_P58276003	136	13	13	0.258
GENE_A_16_P58287590	147	17	17	0.279
GENE_A_16_P21203688	149	15	15	0.283
GENE_A_16_P41397044	142	13	13	0.270
GENE_A_16_P03596477	151	15	16	0.287
GENE_A_16_P21205731	151	17	17	0.287
GENE_A_16_P03578196	161	15	15	0.306
GENE_A_18_P22562837	149	17	17	0.283
GENE_A_16_P21261453	150	15	16	0.285
GENE_A_16_P58279013	147	14	14	0.279
GENE_A_18_P13943300	134	14	14	0.254
GENE_A_16_P21206371	148	16	16	0.281
GENE_A_16_P21234873	155	15	15	0.294
GENE_A_16_P41408684	144	12	12	0.273
GENE_A_16_P41399253	156	18	18	0.296
GENE_A_16_P34779448	145	12	12	0.275
GENE_A_16_P03594314	119	11	11	0.226
GENE_A_16_P03576022	136	13	13	0.258
GENE_A_16_P58277355	161	16	16	0.305
GENE_A_18_P13941777	160	15	15	0.303
GENE_A_16_P21260956	148	13	13	0.281
GENE_A_16_P58266580	132	14	14	0.250
GENE_A_18_P13858708	151	13	13	0.286
GENE_A_16_P03592694	147	14	14	0.279
GENE_A_16_P58295148	151	15	15	0.286
GENE_A_16_P21249380	138	14	14	0.262
GENE_A_16_P21210987	155	15	15	0.294
GENE_A_18_P13881454	153	11	11	0.290
GENE_A_16_P03576684	151	16	16	0.286
GENE_A_16_P03596011	162	15	15	0.307
GENE_A_16_P21227670	141	15	16	0.267
GENE_A_18_P13884593	133	12	13	0.252
GENE_A_16_P58265822	154	15	15	0.292
GENE_A_16_P58280601	139	15	15	0.263
GENE_A_16_P21242430	139	15	15	0.263
GENE_A_16_P58263718	161	15	15	0.305
GENE_A_16_P21223056	144	13	13	0.273
GENE_A_16_P58285984	165	18	18	0.312
GENE_A_16_P03554694	130	13	13	0.246
GENE_A_16_P21257934	156	17	17	0.295
GENE_A_16_P03587646	135	14	14	0.255
GENE_A_16_P58259847	161	16	16	0.305
GENE_A_16_P58283835	154	13	14	0.291
GENE_A_16_P21266300	132	10	10	0.250
GENE_A_18_P13944730	147	15	15	0.278

GENE_A_16_P21228410	167	13	13	0.316
GENE_A_16_P58302401	145	14	14	0.274
GENE_A_16_P58268996	164	15	15	0.310
GENE_A_16_P21238973	146	13	13	0.276
GENE_A_16_P21220060	144	16	16	0.272
GENE_A_16_P58295703	159	15	15	0.300
GENE_A_16_P21258011	138	13	13	0.261
GENE_A_16_P21212118	153	15	15	0.289
GENE_A_16_P21260928	149	17	17	0.281
GENE_A_16_P58283078	126	13	13	0.238
GENE_A_16_P03594026	134	10	10	0.253
GENE_A_16_P41391476	140	13	13	0.264
GENE_A_18_P13924279	159	16	16	0.300
GENE_A_16_P21232137	148	15	15	0.279
GENE_A_16_P58291772	156	16	16	0.294
GENE_A_16_P21198602	136	15	15	0.257
GENE_A_16_P58277837	131	13	13	0.247
GENE_A_16_P58308276	161	14	14	0.304
GENE_A_16_P21259585	129	14	14	0.243
GENE_A_16_P21215589	169	17	17	0.319
GENE_A_16_P21226916	150	13	13	0.283
GENE_A_18_P13898981	151	16	17	0.285
GENE_A_16_P21247706	126	13	13	0.238
GENE_A_16_P41423982	155	14	14	0.292
GENE_A_18_P22509448	152	13	13	0.287
GENE_A_16_P21248285	136	12	14	0.256
GENE_A_16_P58271606	153	16	16	0.288
GENE_A_16_P58281753	156	17	18	0.294
GENE_A_16_P41447031	165	13	13	0.311
GENE_A_16_P58271418	157	21	21	0.296
GENE_A_16_P03563943	156	19	19	0.294
GENE_A_16_P03591509	145	15	15	0.273
GENE_A_18_P13936334	162	18	18	0.305
GENE_A_16_P58283271	162	17	17	0.305
GENE_A_16_P21226783	140	14	14	0.263
GENE_A_18_P22484408	151	15	15	0.284
GENE_A_16_P21244254	152	14	14	0.286
GENE_A_18_P22488715	142	14	14	0.267
GENE_A_16_P58308250	176	18	18	0.331
GENE_A_16_P21222971	158	14	14	0.297
GENE_A_16_P41393905	161	17	17	0.303
GENE_A_16_P03587153	165	12	12	0.310
GENE_A_16_P58267769	152	13	13	0.286
GENE_A_16_P41391700	136	13	13	0.256
GENE_A_16_P21209015	157	15	15	0.295
GENE_A_16_P21218621	151	14	14	0.284

GENE_A_16_P03571815	159	15	16	0.299
GENE_A_16_P58300753	141	14	14	0.265
GENE_A_16_P21200573	153	16	16	0.287
GENE_A_16_P21218955	167	18	18	0.314
GENE_A_14_P133410	127	13	13	0.239
GENE_A_16_P58301016	143	14	14	0.269
GENE_A_16_P58262743	135	14	14	0.254
GENE_A_16_P34821182	171	18	18	0.321
GENE_A_16_P03591168	149	18	18	0.280
GENE_A_18_P13943343	164	18	18	0.308
GENE_A_16_P58260796	166	18	18	0.311
GENE_A_16_P21240058	152	17	17	0.285
GENE_A_16_P58277709	136	10	10	0.255
GENE_A_16_P03594455	137	11	11	0.257
GENE_A_18_P22594002	145	14	14	0.272
GENE_A_16_P58302192	141	14	14	0.264
GENE_A_16_P58277618	135	12	12	0.253
GENE_A_16_P58272372	135	16	16	0.253
GENE_A_16_P21265275	147	13	13	0.276
GENE_A_16_P21194656	136	15	15	0.255
GENE_A_16_P21236338	149	17	17	0.279
GENE_A_16_P58284058	142	15	15	0.266
GENE_A_18_P13891792	130	13	14	0.244
GENE_A_16_P21203294	149	15	15	0.279
GENE_A_16_P03594999	136	11	11	0.255
GENE_A_16_P03580794	154	15	15	0.289
GENE_A_16_P41408475	146	13	13	0.274
GENE_A_16_P03579149	142	13	13	0.266
GENE_A_16_P58309004	149	14	14	0.279
GENE_A_16_P21216923	140	13	13	0.262
GENE_A_16_P41425573	156	17	17	0.292
GENE_A_16_P58276731	148	14	14	0.277
GENE_A_16_P03576412	141	13	14	0.264
GENE_A_18_P13905945	129	11	11	0.241
GENE_A_16_P03581850	171	16	16	0.320
GENE_A_16_P21272218	153	16	16	0.286
GENE_A_18_P13861002	148	15	15	0.277
GENE_A_16_P21227758	166	16	17	0.311
GENE_A_18_P13859660	161	15	15	0.301
GENE_A_18_P22594182	135	13	13	0.253
GENE_A_16_P58280885	141	16	16	0.264
GENE_A_16_P21203723	130	15	15	0.243
GENE_A_16_P21259949	138	14	14	0.258
GENE_A_16_P58266640	169	18	18	0.316
GENE_A_18_P22543933	130	11	11	0.243
GENE_A_16_P58269400	148	16	17	0.277

GENE_A_16_P21272636	148	16	16	0.277
GENE_A_16_P03565165	165	16	16	0.308
GENE_A_16_P21251587	173	19	19	0.323
GENE_A_16_P21209150	146	12	12	0.273
GENE_A_16_P58289048	154	16	16	0.288
GENE_A_16_P03561378	161	16	16	0.301
GENE_A_16_P58277684	155	14	14	0.290
GENE_A_18_P13887412	160	16	16	0.299
GENE_A_16_P58286081	132	14	14	0.247
GENE_A_16_P03595524	169	12	12	0.316
GENE_A_16_P03554013	166	17	17	0.310
GENE_A_16_P58264830	153	15	15	0.286
GENE_A_16_P21240665	149	17	17	0.278
GENE_A_16_P21220190	154	15	15	0.288
GENE_A_16_P21232420	155	16	16	0.289
GENE_A_16_P21235325	143	14	14	0.267
GENE_A_16_P21223675	139	12	12	0.259
GENE_A_16_P21249513	158	12	12	0.295
GENE_A_16_P21235099	153	16	16	0.286
GENE_A_16_P58287513	175	16	16	0.327
GENE_A_16_P41394080	155	14	14	0.289
GENE_A_16_P41430386	159	17	17	0.297
GENE_A_16_P41398443	144	13	13	0.269
GENE_A_16_P03584126	143	14	14	0.267
GENE_A_16_P03595830	152	17	17	0.283
GENE_A_16_P03575315	151	16	16	0.281
GENE_A_16_P21255379	136	15	15	0.253
GENE_A_16_P58291585	153	14	14	0.285
GENE_A_16_P58274481	145	17	17	0.270
GENE_A_18_P13864840	157	16	16	0.292
GENE_A_16_P58286459	143	14	14	0.266
GENE_A_16_P21239932	151	13	13	0.281
GENE_A_16_P58261088	168	17	17	0.313
GENE_A_16_P21229977	146	16	16	0.272
GENE_A_16_P41442448	152	15	15	0.283
GENE_A_16_P21215156	137	14	14	0.255
GENE_A_16_P58293734	163	19	19	0.303
GENE_A_16_P41419226	133	17	17	0.248
GENE_A_18_P13889255	145	17	17	0.270
GENE_A_16_P41406477	143	15	15	0.266
GENE_A_16_P58289553	152	13	13	0.283
GENE_A_16_P58265559	162	16	16	0.301
GENE_A_16_P58305718	143	16	16	0.266
GENE_A_16_P58291287	151	13	13	0.281
GENE_A_16_P21218685	161	16	16	0.299
GENE_A_18_P13919382	126	14	14	0.234

GENE_A_16_P03572716	126	12	12	0.234
GENE_A_16_P21238770	169	18	18	0.314
GENE_A_16_P03594042	161	19	19	0.299
GENE_A_16_P41392544	155	16	16	0.288
GENE_A_16_P21224825	153	16	16	0.284
GENE_A_16_P21232351	130	10	10	0.242
GENE_A_16_P58305765	136	12	12	0.253
GENE_A_18_P22524488	148	20	20	0.275
GENE_A_16_P03594925	137	13	13	0.254
GENE_A_16_P21239027	161	17	17	0.299
GENE_A_16_P03560792	149	17	17	0.277
GENE_A_16_P21194000	148	16	17	0.275
GENE_A_18_P13861419	141	12	12	0.262
GENE_A_16_P41413868	149	15	15	0.277
GENE_A_16_P41460381	160	19	19	0.297
GENE_A_18_P13927433	157	14	14	0.292
GENE_A_16_P58292654	150	16	16	0.278
GENE_A_16_P41408109	162	16	16	0.301
GENE_A_16_P21258684	143	11	11	0.265
GENE_A_18_P13923041	141	14	15	0.262
GENE_A_16_P58274896	166	15	15	0.308
GENE_A_16_P21205866	128	12	12	0.237
GENE_A_16_P21215694	161	14	14	0.299
GENE_A_16_P58276047	147	14	15	0.273
GENE_A_18_P22501450	142	17	17	0.263
GENE_A_16_P58264403	155	17	17	0.287
GENE_A_16_P21212333	142	14	14	0.263
GENE_A_16_P41389844	150	16	16	0.278
GENE_A_16_P41416700	154	14	14	0.286
GENE_A_16_P03556096	133	17	17	0.247
GENE_A_16_P41442103	129	11	11	0.239
GENE_A_16_P41437066	145	14	14	0.269
GENE_A_16_P03591158	139	15	15	0.258
GENE_A_16_P41425285	164	16	16	0.304
GENE_A_18_P22584083	122	13	14	0.226
GENE_A_18_P22594299	138	12	13	0.256
GENE_A_16_P41415372	138	14	14	0.256
GENE_A_16_P58269522	142	15	15	0.263
GENE_A_16_P03586623	162	15	15	0.300
GENE_A_18_P22532610	162	16	17	0.300
GENE_A_16_P34775145	150	14	14	0.278
GENE_A_18_P13920309	159	15	16	0.294
GENE_A_16_P41442253	138	13	13	0.255
GENE_A_16_P58267203	149	15	15	0.276
GENE_A_18_P22488985	153	18	18	0.283
GENE_A_16_P41390357	143	16	16	0.265

GENE_A_18_P13946050	148	16	16	0.274
GENE_A_18_P13946153	153	16	16	0.283
GENE_A_16_P41445864	135	12	12	0.250
GENE_A_16_P41436850	139	12	12	0.257
GENE_A_18_P13891532	138	13	13	0.255
GENE_A_16_P58305938	137	12	12	0.253
GENE_A_16_P21228688	150	15	15	0.277
GENE_A_16_P58269230	152	15	15	0.281
GENE_A_16_P21242301	140	16	16	0.259
GENE_A_16_P21214975	155	15	15	0.286
GENE_A_16_P41431965	162	19	19	0.299
GENE_A_16_P03556317	155	17	17	0.286
GENE_A_16_P21271949	153	16	16	0.283
GENE_A_16_P21201469	163	18	18	0.301
GENE_A_16_P21246328	166	16	16	0.307
GENE_A_16_P03569308	162	15	16	0.299
GENE_A_16_P03582161	133	12	12	0.246
GENE_A_16_P41467135	142	15	15	0.262
GENE_A_16_P21250866	134	12	12	0.247
GENE_A_16_P21234720	156	15	15	0.288
GENE_A_16_P41432184	152	17	17	0.281
GENE_A_16_P58287643	137	14	14	0.253
GENE_A_16_P21248589	145	13	13	0.268
GENE_A_16_P21202879	138	13	13	0.255
GENE_A_16_P03563148	156	16	16	0.288
GENE_A_16_P21252539	148	14	14	0.273
GENE_A_16_P58260380	154	16	16	0.284
GENE_A_16_P21218535	139	14	14	0.256
GENE_A_16_P21258406	140	11	11	0.258
GENE_A_16_P58276984	136	15	15	0.251
GENE_A_16_P03580678	172	17	18	0.317
GENE_A_16_P21265364	165	16	16	0.304
GENE_A_16_P21202943	151	13	13	0.278
GENE_A_16_P41417349	153	16	16	0.282
GENE_A_16_P58287253	140	14	14	0.258
GENE_A_16_P58295213	150	16	16	0.276
GENE_A_16_P58281035	159	15	15	0.293
GENE_A_18_P13885925	158	15	15	0.291
GENE_A_18_P22520790	153	14	14	0.281
GENE_A_16_P03588474	165	14	15	0.303
GENE_A_16_P03580199	153	14	14	0.281
GENE_A_16_P21228092	152	14	14	0.280
GENE_A_16_P21237995	153	15	15	0.281
GENE_A_16_P21197743	135	10	10	0.248
GENE_A_16_P58270495	140	13	13	0.257
GENE_A_16_P21213959	167	16	16	0.307

GENE_A_16_P03555232	143	14	14	0.263
GENE_A_16_P03575739	141	11	11	0.259
GENE_A_16_P21219333	138	15	15	0.254
GENE_A_16_P21248216	147	17	17	0.270
GENE_A_16_P58279263	161	19	19	0.296
GENE_A_18_P13881368	151	17	17	0.278
GENE_A_16_P58309378	177	17	17	0.325
GENE_A_16_P21193998	162	18	18	0.298
GENE_A_16_P21248364	131	15	15	0.241
GENE_A_16_P41402219	163	18	18	0.300
GENE_A_16_P03579573	149	14	14	0.274
GENE_A_16_P41457422	172	18	18	0.316
GENE_A_16_P58304685	157	12	12	0.288
GENE_A_16_P21226740	148	14	14	0.272
GENE_A_16_P21236583	148	12	12	0.272
GENE_A_16_P03568436	167	19	19	0.307
GENE_A_16_P03594994	146	16	16	0.268
GENE_A_16_P58290823	142	13	13	0.261
GENE_A_16_P21215187	143	17	17	0.262
GENE_A_18_P13937318	152	13	13	0.279
GENE_A_16_P58265602	155	17	17	0.284
GENE_A_16_P03555934	164	17	17	0.301
GENE_A_16_P21257969	158	17	17	0.290
GENE_A_16_P21228072	114	12	12	0.209
GENE_A_16_P58287529	166	19	19	0.304
GENE_A_16_P21197460	129	15	15	0.237
GENE_A_16_P41419830	141	14	14	0.259
GENE_A_16_P21232423	139	14	14	0.255
GENE_A_16_P03582845	151	12	12	0.277
GENE_A_16_P41403168	150	12	13	0.275
GENE_A_16_P58262506	156	13	13	0.286
GENE_A_18_P13891245	162	12	12	0.297
GENE_A_16_P21225121	162	15	15	0.297
GENE_A_18_P13916565	144	14	14	0.264
GENE_A_16_P58270845	139	15	15	0.255
GENE_A_16_P41426708	142	13	13	0.260
GENE_A_16_P03568924	124	12	12	0.227
GENE_A_16_P03554254	155	13	13	0.284
GENE_A_16_P03590402	156	16	16	0.286
GENE_A_16_P03592473	140	11	11	0.256
GENE_A_16_P21248520	155	15	15	0.284
GENE_A_16_P03564006	134	12	13	0.245
GENE_A_16_P41401149	165	15	15	0.302
GENE_A_16_P41395284	154	15	15	0.282
GENE_A_16_P03585212	152	15	15	0.278
GENE_A_16_P03583338	144	13	13	0.263

GENE_A_16_P21200856	174	18	18	0.318
GENE_A_16_P41466570	156	13	15	0.285
GENE_A_18_P22590704	143	16	16	0.262
GENE_A_16_P21202690	141	14	14	0.258
GENE_A_16_P21224310	149	14	14	0.272
GENE_A_16_P58308235	157	14	14	0.287
GENE_A_16_P21193803	129	12	12	0.236
GENE_A_16_P58262785	162	14	14	0.296
GENE_A_16_P58285537	136	12	12	0.249
GENE_A_16_P58277284	174	20	20	0.318
GENE_A_16_P41449516	161	13	13	0.294
GENE_A_16_P21204828	142	16	16	0.259
GENE_A_16_P34817222	152	15	15	0.278
GENE_A_16_P03593196	146	16	16	0.267
GENE_A_18_P13898665	153	14	14	0.280
GENE_A_16_P21249077	156	13	13	0.285
GENE_A_18_P13879329	145	12	13	0.265
GENE_A_16_P21246465	150	15	15	0.274
GENE_A_16_P21199808	164	16	16	0.299
GENE_A_18_P13923298	154	15	15	0.281
GENE_A_16_P03555856	167	18	18	0.305
GENE_A_16_P03588591	145	12	12	0.265
GENE_A_16_P03581593	147	15	15	0.268
GENE_A_16_P21209655	154	15	15	0.281
GENE_A_16_P03592501	166	17	17	0.303
GENE_A_16_P58277397	131	15	15	0.239
GENE_A_18_P13909849	161	17	17	0.294
GENE_A_16_P41392979	167	16	16	0.305
GENE_A_18_P13864871	160	12	12	0.292
GENE_A_16_P21212702	158	13	13	0.288
GENE_A_16_P41430062	179	21	21	0.326
GENE_A_18_P22559756	141	13	13	0.257
GENE_A_16_P58299000	159	17	17	0.290
GENE_A_16_P21227348	139	12	12	0.253
GENE_A_16_P21229166	135	14	14	0.246
GENE_A_16_P41453335	161	18	18	0.293
GENE_A_16_P21202730	144	17	17	0.262
GENE_A_18_P13876081	163	14	14	0.297
GENE_A_18_P22559208	190	18	18	0.346
GENE_A_16_P03576742	125	12	12	0.228
GENE_A_16_P58275892	164	17	17	0.299
GENE_A_16_P03558899	167	17	17	0.304
GENE_A_16_P03581376	140	13	13	0.255
GENE_A_16_P58267876	154	15	15	0.280
GENE_A_16_P58283969	145	16	16	0.264
GENE_A_16_P21228924	144	12	12	0.262

GENE_A_16_P58298216	167	14	14	0.304
GENE_A_18_P13931769	152	14	14	0.277
GENE_A_16_P03583880	164	15	15	0.298
GENE_A_16_P41445062	169	19	19	0.307
GENE_A_16_P58295285	155	19	19	0.282
GENE_A_16_P21221675	154	15	15	0.280
GENE_A_16_P41402843	150	11	11	0.273
GENE_A_16_P58289195	163	13	13	0.296
GENE_A_16_P58274275	155	16	17	0.282
GENE_A_16_P03552918	164	16	16	0.298
GENE_A_16_P34803004	128	12	12	0.233
GENE_A_16_P21203744	153	14	14	0.278
GENE_A_18_P13924157	158	15	15	0.287
GENE_A_16_P58289744	164	17	18	0.298
GENE_A_16_P03578708	139	14	14	0.253
GENE_A_16_P41471052	130	12	12	0.236
GENE_A_16_P03562050	148	13	14	0.269
GENE_A_16_P58291095	150	14	14	0.273
GENE_A_16_P21227035	137	12	12	0.249
GENE_A_16_P03552578	153	18	18	0.278
GENE_A_16_P21212439	154	14	14	0.280
GENE_A_16_P41391382	137	13	13	0.249
GENE_A_16_P21200533	156	16	16	0.283
GENE_A_18_P22558324	155	18	18	0.281
GENE_A_18_P13886290	131	10	10	0.238
GENE_A_16_P21252300	169	17	17	0.307
GENE_A_16_P03561424	175	19	19	0.318
GENE_A_16_P58290165	134	11	11	0.243
GENE_A_16_P21193283	137	16	16	0.249
GENE_A_16_P41420967	157	14	14	0.285
GENE_A_18_P13905559	168	14	14	0.305
GENE_A_16_P21197745	144	13	14	0.261
GENE_A_16_P03592837	144	14	15	0.261
GENE_A_16_P21246781	159	16	16	0.288
GENE_A_16_P21233200	148	12	12	0.268
GENE_A_16_P58265164	163	17	17	0.295
GENE_A_16_P03589477	145	11	11	0.263
GENE_A_16_P21217947	158	15	15	0.286
GENE_A_18_P13890039	149	11	11	0.270
GENE_A_18_P13900668	177	16	16	0.321
GENE_A_16_P58263769	150	13	13	0.272
GENE_A_16_P41403411	157	17	17	0.284
GENE_A_16_P41448987	149	16	16	0.270
GENE_A_16_P21196344	158	13	14	0.286
GENE_A_16_P03568539	156	19	19	0.283
GENE_A_18_P13905547	145	13	13	0.263

GENE_A_16_P03561349	151	18	18	0.273
GENE_A_16_P58296353	154	15	15	0.279
GENE_A_16_P03571124	159	16	16	0.288
GENE_A_16_P21245316	139	14	14	0.252
GENE_A_16_P21247787	155	16	16	0.281
GENE_A_16_P58275609	138	15	15	0.250
GENE_A_16_P21229671	147	13	14	0.266
GENE_A_18_P22493240	169	16	16	0.306
GENE_A_18_P13896234	142	12	12	0.257
GENE_A_16_P34821511	150	14	14	0.271
GENE_A_16_P58275410	150	14	14	0.271
GENE_A_16_P58266931	177	14	14	0.320
GENE_A_18_P22588341	166	16	16	0.300
GENE_A_16_P21221166	154	12	12	0.279
GENE_A_16_P41416935	149	12	12	0.269
GENE_A_16_P03581657	131	13	13	0.237
GENE_A_18_P13931896	148	14	14	0.268
GENE_A_18_P22587162	151	17	17	0.273
GENE_A_16_P41425294	149	15	15	0.269
GENE_A_16_P41389723	152	16	16	0.275
GENE_A_16_P21243753	160	16	16	0.289
GENE_A_18_P22587763	144	10	10	0.260
GENE_A_16_P21217141	139	15	15	0.251
GENE_A_16_P21260267	145	14	14	0.262
GENE_A_16_P21234889	168	14	14	0.304
GENE_A_16_P41394984	157	11	11	0.284
GENE_A_16_P41406144	149	17	17	0.269
GENE_A_16_P58268624	157	16	16	0.284
GENE_A_16_P21248173	169	18	18	0.305
GENE_A_16_P21221686	167	15	15	0.302
GENE_A_16_P03588846	144	14	14	0.260
GENE_A_16_P21259300	160	19	19	0.289
GENE_A_18_P22519971	148	14	14	0.267
GENE_A_16_P21207036	158	13	13	0.285
GENE_A_16_P58297996	158	18	18	0.285
GENE_A_16_P58291148	143	13	13	0.258
GENE_A_18_P13927957	131	12	12	0.237
GENE_A_16_P21219605	170	17	18	0.307
GENE_A_16_P03567437	155	15	15	0.280
GENE_A_16_P03597840	135	11	11	0.244
GENE_A_16_P21256797	155	13	13	0.280
GENE_A_16_P03556674	168	17	17	0.303
GENE_A_16_P58268511	169	17	17	0.305
GENE_A_16_P41399889	166	13	13	0.300
GENE_A_16_P58260108	140	15	15	0.253
GENE_A_16_P21196198	153	13	13	0.276

GENE_A_16_P21202186	164	13	13	0.296
GENE_A_18_P13893831	153	14	14	0.276
GENE_A_16_P58263320	162	16	16	0.292
GENE_A_16_P58270669	148	15	15	0.267
GENE_A_16_P03580766	173	15	15	0.312
GENE_A_16_P21252328	171	20	20	0.309
GENE_A_16_P58263351	157	16	16	0.283
GENE_A_16_P03562117	157	13	13	0.283
GENE_A_16_P58276937	155	15	15	0.280
GENE_A_18_P13869644	159	15	15	0.287
GENE_A_16_P21237745	151	16	16	0.272
GENE_A_16_P21195416	164	17	17	0.296
GENE_A_18_P13881731	147	13	14	0.265
GENE_A_16_P58294157	156	13	13	0.281
GENE_A_16_P41401732	136	9	9	0.245
GENE_A_16_P21214655	129	14	14	0.232
GENE_A_16_P58267909	151	15	15	0.272
GENE_A_16_P41427161	133	11	11	0.240
GENE_A_16_P03561317	175	16	16	0.315
GENE_A_18_P13945551	141	13	13	0.254
GENE_A_16_P21203878	158	14	14	0.285
GENE_A_18_P13879595	153	13	14	0.276
GENE_A_18_P13873749	160	17	17	0.288
GENE_A_16_P03572834	136	13	13	0.245
GENE_A_16_P03582347	149	14	14	0.268
GENE_A_18_P13884499	151	14	14	0.272
GENE_A_16_P21206519	161	13	13	0.290
GENE_A_16_P21229793	167	15	15	0.301
GENE_A_16_P58268877	152	12	12	0.274
GENE_A_16_P21237937	154	17	18	0.277
GENE_A_16_P21257326	165	17	17	0.297
GENE_A_16_P03570483	165	18	18	0.297
GENE_A_16_P58265205	169	17	17	0.304
GENE_A_16_P58272383	131	14	14	0.236
GENE_A_16_P21212895	155	16	17	0.279
GENE_A_16_P41456476	162	16	16	0.292
GENE_A_16_P21207186	150	14	14	0.270
GENE_A_16_P58267746	151	11	11	0.272
GENE_A_16_P58265677	146	16	16	0.263
GENE_A_16_P03585924	158	15	16	0.284
GENE_A_16_P21214769	148	14	14	0.266
GENE_A_16_P03569727	149	15	15	0.268
GENE_A_18_P13892461	149	14	14	0.268
GENE_A_16_P41457755	138	14	14	0.248
GENE_A_16_P21220215	175	18	18	0.315
GENE_A_16_P41390883	126	9	9	0.227

GENE_A_18_P22549057	121	11	11	0.217
GENE_A_16_P21255612	149	16	16	0.268
GENE_A_16_P21228579	142	14	14	0.255
GENE_A_16_P58299420	153	14	14	0.275
GENE_A_16_P41435798	148	14	14	0.266
GENE_A_16_P58294549	150	12	12	0.269
GENE_A_16_P21195539	158	16	16	0.284
GENE_A_16_P03594580	150	16	16	0.269
GENE_A_16_P58275416	148	15	15	0.266
GENE_A_16_P58271974	142	15	15	0.255
GENE_A_18_P13908404	159	16	16	0.286
GENE_A_16_P58293863	152	14	14	0.273
GENE_A_16_P21197554	137	11	11	0.246
GENE_A_16_P03593908	169	16	16	0.303
GENE_A_16_P21200371	117	13	14	0.210
GENE_A_16_P41448900	143	13	14	0.257
GENE_A_16_P58291107	164	15	15	0.294
GENE_A_16_P58308144	137	13	13	0.246
GENE_A_16_P58281503	158	18	18	0.284
GENE_A_16_P21229298	147	13	13	0.264
GENE_A_16_P21265340	152	16	16	0.273
GENE_A_16_P58274477	147	17	17	0.264
GENE_A_16_P58262765	161	18	18	0.289
GENE_A_16_P21207662	171	15	16	0.307
GENE_A_16_P58268240	127	11	11	0.228
GENE_A_16_P58284147	138	12	12	0.248
GENE_A_16_P21256043	137	13	13	0.246
GENE_A_16_P58283330	159	16	16	0.285
GENE_A_16_P58298691	163	15	15	0.292
GENE_A_16_P21219809	147	14	14	0.264
GENE_A_16_P58298647	165	12	12	0.296
GENE_A_16_P41459766	139	12	12	0.249
GENE_A_16_P58308015	140	14	14	0.251
GENE_A_16_P41438465	172	17	17	0.308
GENE_A_16_P03583715	168	13	14	0.301
GENE_A_16_P58267302	149	14	14	0.267
