# Supplementary material for: Identifying Patients with Atrioventricular Septal Defect in Down Syndrome Populations by Using Self-Normalizing Neural Networks and Feature Selection
Source: Genes (Basel). 2018 Apr 12;9(4):208. doi: 10.3390/genes9040208 (PMC5924550; doi:10.3390/genes9040208)
Supplement: Supplementary file 1 [file genes-09-00208-s001.zip › Suppl_S2.pdf]

Supplementary Material S2. Two series of MCCs and AUCs resulting from IFS method and SNN classifiers.

(1) IFS first series

**Number of features**	***MCC***	***AUC***
10	0.287	0.698
20	0.311	0.699
30	0.323	0.727
40	0.382	0.738
50	0.367	0.743
60	0.359	0.756
70	0.401	0.746
80	0.423	0.785
90	0.424	0.755
100	0.428	0.751
110	0.424	0.767
120	0.426	0.769
130	0.430	0.802
140	0.453	0.790
150	0.430	0.797
160	0.469	0.804
170	0.469	0.819
180	0.510	0.794
190	0.481	0.802
200	0.499	0.819
210	0.537	0.831
220	0.454	0.825
230	0.488	0.847
240	0.530	0.824
250	0.517	0.823
260	0.549	0.826
270	0.534	0.816
280	0.507	0.838
290	0.563	0.848
300	0.501	0.839
310	0.545	0.839
320	0.549	0.840
330	0.530	0.847
340	0.545	0.840
350	0.534	0.849

360	0.539	0.830
370	0.564	0.858
380	0.507	0.840
390	0.553	0.837
400	0.573	0.842
410	0.542	0.833
420	0.580	0.862
430	0.542	0.858
440	0.563	0.859
450	0.592	0.853
460	0.587	0.865
470	0.588	0.861
480	0.572	0.869
490	0.575	0.862
500	0.612	0.874
510	0.581	0.877
520	0.593	0.881
530	0.504	0.868
540	0.580	0.867
550	0.545	0.866
560	0.526	0.862
570	0.577	0.877
580	0.508	0.869
590	0.527	0.873
600	0.538	0.868
610	0.513	0.864
620	0.508	0.857
630	0.554	0.864
640	0.562	0.858
650	0.563	0.859
660	0.509	0.857
670	0.553	0.859
680	0.512	0.877
690	0.583	0.873
700	0.582	0.880
710	0.550	0.876
720	0.553	0.879
730	0.531	0.872
740	0.579	0.893

750	0.524	0.872
760	0.565	0.873
770	0.550	0.885
780	0.566	0.894
790	0.576	0.866
800	0.580	0.883
810	0.570	0.878
820	0.588	0.892
830	0.543	0.887
840	0.598	0.890
850	0.576	0.895
860	0.610	0.881
870	0.521	0.886
880	0.599	0.883
890	0.619	0.899
900	0.562	0.891
910	0.583	0.895
920	0.595	0.880
930	0.658	0.898
940	0.627	0.896
950	0.539	0.900
960	0.611	0.884
970	0.581	0.877
980	0.620	0.886
990	0.609	0.895
1000	0.566	0.890
1010	0.623	0.885
1020	0.616	0.899
1030	0.624	0.908
1040	0.532	0.887
1050	0.616	0.882
1060	0.618	0.889
1070	0.618	0.903
1080	0.597	0.895
1090	0.627	0.893
1100	0.563	0.887
1110	0.615	0.897
1120	0.623	0.885
1130	0.597	0.881

1140	0.630	0.903
1150	0.597	0.885
1160	0.631	0.901
1170	0.615	0.889
1180	0.612	0.899
1190	0.588	0.891
1200	0.576	0.898
1210	0.647	0.895
1220	0.626	0.887
1230	0.622	0.899
1240	0.656	0.902
1250	0.616	0.898
1260	0.592	0.895
1270	0.619	0.892
1280	0.611	0.895
1290	0.635	0.902
1300	0.639	0.904
1310	0.640	0.905
1320	0.617	0.886
1330	0.629	0.884
1340	0.644	0.895
1350	0.601	0.907
1360	0.643	0.897
1370	0.584	0.898
1380	0.617	0.894
1390	0.671	0.887
1400	0.632	0.904
1410	0.634	0.880
1420	0.589	0.889
1430	0.618	0.889
1440	0.669	0.889
1450	0.628	0.901
1460	0.644	0.910
1470	0.626	0.903
1480	0.576	0.892
1490	0.621	0.907
1500	0.605	0.919
1510	0.625	0.911
1520	0.631	0.893

1530	0.675	0.913
1540	0.647	0.903
1550	0.602	0.914
1560	0.642	0.908
1570	0.590	0.914
1580	0.633	0.913
1590	0.606	0.916
1600	0.612	0.918
1610	0.662	0.907
1620	0.643	0.917
1630	0.636	0.925
1640	0.675	0.920
1650	0.631	0.906
1660	0.659	0.927
1670	0.634	0.915
1680	0.641	0.931
1690	0.653	0.909
1700	0.652	0.919
1710	0.667	0.917
1720	0.646	0.905
1730	0.658	0.902
1740	0.668	0.923
1750	0.642	0.912
1760	0.601	0.898
1770	0.593	0.905
1780	0.665	0.908
1790	0.623	0.897
1800	0.637	0.904
1810	0.652	0.906
1820	0.653	0.904
1830	0.631	0.913
1840	0.652	0.921
1850	0.622	0.900
1860	0.666	0.911
1870	0.626	0.898
1880	0.611	0.920
1890	0.648	0.909
1900	0.656	0.914
1910	0.706	0.909

1920	0.585	0.898
1930	0.663	0.913
1940	0.655	0.897
1950	0.633	0.926
1960	0.650	0.901
1970	0.689	0.922
1980	0.675	0.921
1990	0.667	0.913
2000	0.612	0.926
2010	0.665	0.913
2020	0.670	0.918
2030	0.630	0.925
2040	0.656	0.917
2050	0.646	0.913
2060	0.645	0.922
2070	0.671	0.929
2080	0.658	0.915
2090	0.659	0.926
2100	0.647	0.917
2110	0.709	0.920
2120	0.682	0.923
2130	0.637	0.904
2140	0.617	0.921
2150	0.679	0.927
2160	0.662	0.917
2170	0.679	0.908
2180	0.661	0.924
2190	0.643	0.921
2200	0.678	0.922
2210	0.658	0.921
2220	0.627	0.928
2230	0.644	0.920
2240	0.647	0.918
2250	0.647	0.916
2260	0.667	0.912
2270	0.683	0.924
2280	0.672	0.919
2290	0.624	0.907
2300	0.685	0.920

2310	0.685	0.930
2320	0.673	0.925
2330	0.680	0.922
2340	0.650	0.913
2350	0.662	0.913
2360	0.652	0.919
2370	0.642	0.925
2380	0.644	0.920
2390	0.701	0.929
2400	0.615	0.926
2410	0.657	0.924
2420	0.697	0.927
2430	0.704	0.933
2440	0.692	0.920
2450	0.715	0.917
2460	0.689	0.919
2470	0.694	0.931
2480	0.687	0.940
2490	0.620	0.939
2500	0.708	0.936
2510	0.689	0.919
2520	0.636	0.937
2530	0.697	0.919
2540	0.711	0.929
2550	0.681	0.928
2560	0.659	0.921
2570	0.676	0.921
2580	0.667	0.933
2590	0.697	0.929
2600	0.653	0.921
2610	0.656	0.924
2620	0.715	0.919
2630	0.650	0.924
2640	0.691	0.901
2650	0.674	0.930
2660	0.675	0.922
2670	0.665	0.918
2680	0.643	0.927
2690	0.654	0.921

2700	0.670	0.926
2710	0.676	0.933
2720	0.690	0.903
2730	0.636	0.937
2740	0.698	0.926
2750	0.663	0.924
2760	0.679	0.926
2770	0.686	0.922
2780	0.695	0.920
2790	0.632	0.926
2800	0.691	0.933
2810	0.672	0.920
2820	0.701	0.922
2830	0.671	0.917
2840	0.695	0.914
2850	0.661	0.928
2860	0.683	0.928
2870	0.706	0.919
2880	0.678	0.926
2890	0.676	0.911
2900	0.653	0.910
2910	0.674	0.925
2920	0.668	0.919
2930	0.655	0.927
2940	0.646	0.911
2950	0.643	0.942
2960	0.666	0.934
2970	0.625	0.924
2980	0.670	0.934
2990	0.661	0.920
3000	0.658	0.924
3010	0.651	0.919
3020	0.655	0.938
3030	0.667	0.922
3040	0.658	0.926
3050	0.664	0.927
3060	0.643	0.919
3070	0.642	0.919
3080	0.666	0.920

3090	0.645	0.915
3100	0.662	0.926
3110	0.698	0.923
3120	0.639	0.924
3130	0.699	0.926
3140	0.677	0.921
3150	0.656	0.922
3160	0.670	0.915
3170	0.673	0.922
3180	0.692	0.922
3190	0.640	0.905
3200	0.678	0.923
3210	0.644	0.920
3220	0.656	0.920
3230	0.679	0.916
3240	0.681	0.908
3250	0.646	0.920
3260	0.616	0.921
3270	0.708	0.916
3280	0.649	0.934
3290	0.700	0.914
3300	0.644	0.926
3310	0.659	0.927
3320	0.677	0.902
3330	0.647	0.927
3340	0.649	0.931
3350	0.670	0.933
3360	0.704	0.915
3370	0.660	0.914
3380	0.700	0.922
3390	0.643	0.925
3400	0.681	0.936
3410	0.683	0.921
3420	0.696	0.917
3430	0.670	0.914
3440	0.655	0.934
3450	0.663	0.917
3460	0.657	0.929
3470	0.679	0.927

3480	0.704	0.935
3490	0.701	0.925
3500	0.681	0.918
3510	0.674	0.903
3520	0.691	0.912
3530	0.698	0.926
3540	0.676	0.926
3550	0.708	0.900
3560	0.691	0.915
3570	0.672	0.930
3580	0.705	0.913
3590	0.660	0.923
3600	0.685	0.918
3610	0.653	0.917
3620	0.699	0.915
3630	0.663	0.932
3640	0.662	0.915
3650	0.681	0.934
3660	0.688	0.924
3670	0.650	0.933
3680	0.658	0.935
3690	0.643	0.930
3700	0.650	0.934
3710	0.694	0.923
3720	0.665	0.920
3730	0.655	0.923
3740	0.618	0.925
3750	0.656	0.922
3760	0.698	0.923
3770	0.678	0.922
3780	0.689	0.932
3790	0.658	0.924
3800	0.690	0.923
3810	0.639	0.915
3820	0.643	0.917
3830	0.659	0.910
3840	0.669	0.920
3850	0.609	0.907
3860	0.655	0.925

3870	0.685	0.919
3880	0.622	0.934
3890	0.678	0.916
3900	0.631	0.918
3910	0.666	0.923
3920	0.684	0.902
3930	0.667	0.901
3940	0.629	0.916
3950	0.642	0.925
3960	0.671	0.910
3970	0.647	0.939
3980	0.686	0.921
3990	0.665	0.925
4000	0.640	0.917
4010	0.678	0.928
4020	0.661	0.926
4030	0.664	0.921
4040	0.663	0.913
4050	0.689	0.926
4060	0.700	0.920
4070	0.639	0.914
4080	0.647	0.922
4090	0.633	0.930
4100	0.662	0.907
4110	0.661	0.930
4120	0.659	0.915
4130	0.666	0.923
4140	0.672	0.925
4150	0.689	0.932
4160	0.652	0.917
4170	0.621	0.920
4180	0.689	0.925
4190	0.679	0.911
4200	0.693	0.914
4210	0.687	0.923
4220	0.700	0.923
4230	0.663	0.916
4240	0.659	0.915
4250	0.667	0.895

4260	0.670	0.920
4270	0.657	0.920
4280	0.695	0.923
4290	0.687	0.908
4300	0.689	0.918
4310	0.693	0.913
4320	0.672	0.930
4330	0.728	0.924
4340	0.647	0.897
4350	0.702	0.927
4360	0.717	0.916
4370	0.708	0.913
4380	0.701	0.925
4390	0.641	0.912
4400	0.676	0.909
4410	0.689	0.918
4420	0.633	0.925
4430	0.709	0.908
4440	0.693	0.921
4450	0.652	0.909
4460	0.671	0.923
4470	0.690	0.926
4480	0.659	0.931
4490	0.682	0.924
4500	0.693	0.919
4510	0.688	0.923
4520	0.667	0.917
4530	0.649	0.917
4540	0.675	0.908
4550	0.694	0.908
4560	0.658	0.932
4570	0.644	0.936
4580	0.628	0.912
4590	0.657	0.917
4600	0.651	0.928
4610	0.646	0.927
4620	0.662	0.912
4630	0.685	0.922
4640	0.633	0.915

4650	0.660	0.924
4660	0.667	0.918
4670	0.674	0.920
4680	0.665	0.910
4690	0.701	0.922
4700	0.695	0.920
4710	0.661	0.916
4720	0.678	0.922
4730	0.635	0.924
4740	0.643	0.920
4750	0.635	0.913
4760	0.670	0.930
4770	0.698	0.930
4780	0.663	0.918
4790	0.665	0.916
4800	0.673	0.909
4810	0.647	0.916
4820	0.639	0.922
4830	0.655	0.921
4840	0.631	0.917
4850	0.659	0.919
4860	0.663	0.912
4870	0.662	0.912
4880	0.675	0.913
4890	0.615	0.923
4900	0.666	0.911
4910	0.633	0.923
4920	0.673	0.920
4930	0.667	0.902
4940	0.700	0.903
4950	0.671	0.915
4960	0.673	0.921
4970	0.670	0.909
4980	0.682	0.922
4990	0.620	0.913
5000	0.657	0.914

(2) IFS second series

Number of features	*MCC*	*AUC*
2001	0.670	0.921
2002	0.627	0.921
2003	0.654	0.920
2004	0.689	0.935
2005	0.648	0.925
2006	0.644	0.915
2007	0.681	0.916
2008	0.654	0.915
2009	0.667	0.924
2010	0.665	0.913
2011	0.687	0.927
2012	0.668	0.930
2013	0.621	0.909
2014	0.655	0.919
2015	0.625	0.926
2016	0.618	0.925
2017	0.679	0.918
2018	0.668	0.925
2019	0.665	0.925
2020	0.670	0.918
2021	0.632	0.931
2022	0.621	0.909
2023	0.721	0.931
2024	0.638	0.932
2025	0.714	0.918
2026	0.693	0.913
2027	0.628	0.919
2028	0.655	0.915
2029	0.641	0.912
2030	0.630	0.925
2031	0.629	0.914
2032	0.674	0.930
2033	0.660	0.916
2034	0.641	0.916
2035	0.654	0.917
2036	0.687	0.925
2037	0.701	0.926

2038	0.669	0.913
2039	0.652	0.927
2040	0.656	0.917
2041	0.686	0.905
2042	0.644	0.920
2043	0.655	0.928
2044	0.729	0.920
2045	0.667	0.918
2046	0.679	0.915
2047	0.642	0.921
2048	0.639	0.912
2049	0.590	0.933
2050	0.646	0.913
2051	0.661	0.932
2052	0.639	0.907
2053	0.658	0.915
2054	0.665	0.919
2055	0.694	0.928
2056	0.640	0.909
2057	0.656	0.908
2058	0.673	0.920
2059	0.653	0.923
2060	0.645	0.922
2061	0.646	0.919
2062	0.617	0.918
2063	0.650	0.916
2064	0.618	0.926
2065	0.663	0.925
2066	0.656	0.922
2067	0.652	0.902
2068	0.650	0.923
2069	0.685	0.924
2070	0.671	0.929
2071	0.655	0.930
2072	0.654	0.919
2073	0.622	0.910
2074	0.698	0.912
2075	0.655	0.918
2076	0.655	0.918

2077	0.666	0.922
2078	0.681	0.915
2079	0.684	0.932
2080	0.658	0.915
2081	0.673	0.915
2082	0.685	0.921
2083	0.678	0.914
2084	0.659	0.914
2085	0.682	0.917
2086	0.693	0.925
2087	0.624	0.932
2088	0.680	0.921
2089	0.652	0.916
2090	0.659	0.926
2091	0.674	0.922
2092	0.668	0.923
2093	0.646	0.920
2094	0.654	0.911
2095	0.624	0.931
2096	0.722	0.925
2097	0.643	0.918
2098	0.675	0.926
2099	0.692	0.916
2100	0.647	0.917
2101	0.689	0.916
2102	0.687	0.921
2103	0.664	0.916
2104	0.628	0.913
2105	0.670	0.925
2106	0.695	0.913
2107	0.721	0.926
2108	0.716	0.909
2109	0.615	0.921
2110	0.709	0.920
2111	0.702	0.921
2112	0.639	0.920
2113	0.676	0.910
2114	0.679	0.907
2115	0.676	0.925

2116	0.686	0.915
2117	0.652	0.919
2118	0.685	0.925
2119	0.686	0.913
2120	0.682	0.923
2121	0.647	0.922
2122	0.618	0.915
2123	0.644	0.921
2124	0.686	0.919
2125	0.671	0.920
2126	0.645	0.923
2127	0.681	0.915
2128	0.693	0.922
2129	0.668	0.922
2130	0.637	0.904
2131	0.639	0.905
2132	0.651	0.920
2133	0.656	0.910
2134	0.662	0.919
2135	0.646	0.928
2136	0.690	0.917
2137	0.716	0.916
2138	0.709	0.914
2139	0.681	0.905
2140	0.617	0.921
2141	0.649	0.915
2142	0.662	0.918
2143	0.705	0.915
2144	0.644	0.923
2145	0.635	0.905
2146	0.633	0.927
2147	0.670	0.923
2148	0.653	0.925
2149	0.657	0.917
2150	0.679	0.927
2151	0.643	0.914
2152	0.637	0.914
2153	0.674	0.910
2154	0.648	0.924

2155	0.685	0.913
2156	0.704	0.921
2157	0.645	0.918
2158	0.689	0.916
2159	0.647	0.898
2160	0.662	0.917
2161	0.674	0.929
2162	0.648	0.920
2163	0.600	0.927
2164	0.697	0.915
2165	0.652	0.916
2166	0.688	0.925
2167	0.680	0.905
2168	0.637	0.926
2169	0.637	0.920
2170	0.679	0.908
2171	0.650	0.913
2172	0.627	0.916
2173	0.643	0.920
2174	0.681	0.914
2175	0.723	0.918
2176	0.678	0.914
2177	0.636	0.931
2178	0.692	0.921
2179	0.693	0.919
2180	0.661	0.924
2181	0.642	0.922
2182	0.631	0.921
2183	0.716	0.906
2184	0.631	0.919
2185	0.679	0.925
2186	0.622	0.924
2187	0.654	0.923
2188	0.680	0.931
2189	0.712	0.917
2190	0.643	0.921
2191	0.664	0.915
2192	0.663	0.922
2193	0.663	0.901

2194	0.656	0.914
2195	0.679	0.917
2196	0.678	0.920
2197	0.638	0.924
2198	0.696	0.929
2199	0.652	0.918
2200	0.678	0.922
2201	0.668	0.923
2202	0.646	0.927
2203	0.694	0.908
2204	0.681	0.911
2205	0.693	0.916
2206	0.615	0.925
2207	0.641	0.915
2208	0.646	0.914
2209	0.689	0.923
2210	0.658	0.921
2211	0.663	0.915
2212	0.653	0.914
2213	0.705	0.914
2214	0.616	0.918
2215	0.632	0.924
2216	0.682	0.931
2217	0.635	0.933
2218	0.690	0.912
2219	0.675	0.912
2220	0.627	0.928
2221	0.651	0.915
2222	0.632	0.908
2223	0.667	0.911
2224	0.674	0.919
2225	0.612	0.909
2226	0.650	0.909
2227	0.655	0.917
2228	0.673	0.925
2229	0.675	0.933
2230	0.644	0.920
2231	0.701	0.926
2232	0.679	0.921

2233	0.654	0.925
2234	0.652	0.914
2235	0.652	0.908
2236	0.708	0.927
2237	0.641	0.929
2238	0.649	0.918
2239	0.657	0.922
2240	0.647	0.918
2241	0.664	0.920
2242	0.653	0.915
2243	0.666	0.919
2244	0.639	0.921
2245	0.654	0.912
2246	0.640	0.926
2247	0.657	0.910
2248	0.664	0.923
2249	0.685	0.924
2250	0.647	0.916
2251	0.679	0.919
2252	0.666	0.916
2253	0.646	0.922
2254	0.660	0.916
2255	0.704	0.917
2256	0.648	0.921
2257	0.646	0.921
2258	0.700	0.917
2259	0.683	0.912
2260	0.667	0.912
2261	0.687	0.921
2262	0.695	0.912
2263	0.694	0.910
2264	0.665	0.922
2265	0.669	0.926
2266	0.662	0.927
2267	0.639	0.918
2268	0.673	0.914
2269	0.674	0.928
2270	0.683	0.924
2271	0.696	0.908

2272	0.681	0.919
2273	0.635	0.924
2274	0.651	0.926
2275	0.654	0.920
2276	0.629	0.911
2277	0.637	0.903
2278	0.647	0.925
2279	0.611	0.930
2280	0.672	0.919
2281	0.648	0.915
2282	0.631	0.913
2283	0.683	0.900
2284	0.666	0.913
2285	0.633	0.916
2286	0.708	0.932
2287	0.709	0.926
2288	0.660	0.911
2289	0.671	0.931
2290	0.624	0.907
2291	0.706	0.907
2292	0.625	0.907
2293	0.670	0.921
2294	0.697	0.909
2295	0.721	0.913
2296	0.650	0.924
2297	0.681	0.916
2298	0.682	0.925
2299	0.668	0.900
2300	0.685	0.920
2301	0.658	0.920
2302	0.678	0.925
2303	0.639	0.918
2304	0.693	0.919
2305	0.659	0.914
2306	0.654	0.928
2307	0.693	0.930
2308	0.677	0.908
2309	0.667	0.914
2310	0.685	0.930

2311	0.685	0.919
2312	0.685	0.909
2313	0.638	0.928
2314	0.662	0.918
2315	0.648	0.911
2316	0.664	0.926
2317	0.710	0.911
2318	0.635	0.925
2319	0.654	0.933
2320	0.673	0.925
2321	0.667	0.926
2322	0.696	0.910
2323	0.667	0.923
2324	0.688	0.936
2325	0.679	0.918
2326	0.705	0.927
2327	0.712	0.918
2328	0.697	0.913
2329	0.667	0.927
2330	0.680	0.922
2331	0.723	0.914
2332	0.672	0.915
2333	0.698	0.931
2334	0.682	0.919
2335	0.662	0.909
2336	0.669	0.918
2337	0.685	0.905
2338	0.696	0.920
2339	0.706	0.926
2340	0.650	0.913
2341	0.671	0.915
2342	0.626	0.913
2343	0.633	0.923
2344	0.682	0.924
2345	0.695	0.929
2346	0.691	0.930
2347	0.651	0.925
2348	0.650	0.915
2349	0.670	0.919

2350	0.662	0.913
2351	0.641	0.921
2352	0.723	0.910
2353	0.688	0.910
2354	0.685	0.918
2355	0.666	0.927
2356	0.687	0.917
2357	0.637	0.911
2358	0.708	0.915
2359	0.640	0.923
2360	0.652	0.919
2361	0.644	0.928
2362	0.679	0.933
2363	0.654	0.914
2364	0.616	0.935
2365	0.675	0.926
2366	0.646	0.924
2367	0.647	0.909
2368	0.667	0.913
2369	0.646	0.924
2370	0.642	0.925
2371	0.675	0.914
2372	0.705	0.926
2373	0.675	0.930
2374	0.678	0.928
2375	0.605	0.906
2376	0.629	0.920
2377	0.699	0.936
2378	0.643	0.916
2379	0.695	0.927
2380	0.644	0.920
2381	0.657	0.928
2382	0.711	0.921
2383	0.701	0.922
2384	0.665	0.910
2385	0.704	0.920
2386	0.688	0.925
2387	0.684	0.925
2388	0.620	0.933

2389	0.689	0.925
2390	0.701	0.929
2391	0.658	0.930
2392	0.701	0.918
2393	0.684	0.924
2394	0.671	0.927
2395	0.696	0.925
2396	0.698	0.937
2397	0.684	0.929
2398	0.713	0.917
2399	0.660	0.928
2400	0.615	0.926
2401	0.716	0.929
2402	0.653	0.931
2403	0.675	0.926
2404	0.666	0.934
2405	0.669	0.922
2406	0.675	0.923
2407	0.643	0.906
2408	0.708	0.923
2409	0.685	0.920
2410	0.657	0.924
2411	0.639	0.924
2412	0.666	0.931
2413	0.685	0.915
2414	0.669	0.933
2415	0.693	0.910
2416	0.723	0.926
2417	0.641	0.924
2418	0.686	0.919
2419	0.671	0.922
2420	0.697	0.927
2421	0.706	0.929
2422	0.628	0.922
2423	0.683	0.940
2424	0.677	0.917
2425	0.684	0.913
2426	0.670	0.925
2427	0.650	0.917

2428	0.649	0.925
2429	0.702	0.922
2430	0.704	0.933
2431	0.700	0.926
2432	0.712	0.920
2433	0.660	0.929
2434	0.705	0.903
2435	0.655	0.926
2436	0.640	0.926
2437	0.687	0.936
2438	0.716	0.917
2439	0.661	0.914
2440	0.692	0.920
2441	0.702	0.922
2442	0.654	0.919
2443	0.679	0.920
2444	0.712	0.916
2445	0.717	0.923
2446	0.649	0.931
2447	0.687	0.919
2448	0.701	0.927
2449	0.708	0.923
2450	0.715	0.917
2451	0.679	0.918
2452	0.699	0.926
2453	0.678	0.928
2454	0.663	0.937
2455	0.671	0.914
2456	0.682	0.912
2457	0.649	0.937
2458	0.659	0.919
2459	0.708	0.925
2460	0.689	0.919
2461	0.709	0.919
2462	0.684	0.934
2463	0.642	0.921
2464	0.660	0.923
2465	0.704	0.928
2466	0.703	0.930

2467	0.666	0.927
2468	0.679	0.916
2469	0.649	0.921
2470	0.694	0.931
2471	0.639	0.929
2472	0.713	0.921
2473	0.679	0.931
2474	0.644	0.927
2475	0.696	0.919
2476	0.667	0.931
2477	0.675	0.927
2478	0.647	0.923
2479	0.688	0.919
2480	0.687	0.940
2481	0.673	0.929
2482	0.694	0.920
2483	0.666	0.932
2484	0.670	0.919
2485	0.669	0.926
2486	0.679	0.927
2487	0.666	0.926
2488	0.694	0.918
2489	0.728	0.925
2490	0.620	0.939
2491	0.675	0.913
2492	0.712	0.933
2493	0.642	0.933
2494	0.672	0.924
2495	0.655	0.919
2496	0.667	0.919
2497	0.689	0.925
2498	0.705	0.922
2499	0.691	0.916
2500	0.708	0.936
2501	0.673	0.931
2502	0.633	0.916
2503	0.706	0.927
2504	0.708	0.920
2505	0.640	0.931

2506	0.661	0.915
2507	0.675	0.921
2508	0.679	0.926
2509	0.707	0.916
2510	0.689	0.919
2511	0.633	0.915
2512	0.693	0.925
2513	0.687	0.908
2514	0.652	0.927
2515	0.685	0.920
2516	0.698	0.944
2517	0.678	0.929
2518	0.731	0.924
2519	0.673	0.921
2520	0.636	0.937
2521	0.678	0.926
2522	0.657	0.927
2523	0.663	0.926
2524	0.678	0.920
2525	0.674	0.925
2526	0.675	0.925
2527	0.689	0.929
2528	0.705	0.928
2529	0.693	0.923
2530	0.697	0.919
2531	0.689	0.932
2532	0.671	0.929
2533	0.687	0.925
2534	0.648	0.916
2535	0.651	0.914
2536	0.654	0.922
2537	0.705	0.907
2538	0.684	0.929
2539	0.675	0.919
2540	0.711	0.929
2541	0.637	0.927
2542	0.693	0.917
2543	0.667	0.918
2544	0.663	0.929

2545	0.670	0.917
2546	0.685	0.932
2547	0.668	0.925
2548	0.696	0.918
2549	0.656	0.932
2550	0.681	0.928
2551	0.657	0.922
2552	0.679	0.927
2553	0.659	0.916
2554	0.704	0.920
2555	0.705	0.928
2556	0.640	0.926
2557	0.698	0.917
2558	0.692	0.927
2559	0.624	0.935
2560	0.659	0.921
2561	0.702	0.922
2562	0.704	0.923
2563	0.679	0.915
2564	0.722	0.926
2565	0.662	0.928
2566	0.670	0.927
2567	0.681	0.930
2568	0.654	0.924
2569	0.704	0.937
2570	0.676	0.921
2571	0.664	0.920
2572	0.678	0.922
2573	0.696	0.925
2574	0.690	0.923
2575	0.716	0.935
2576	0.639	0.932
2577	0.702	0.927
2578	0.719	0.920
2579	0.655	0.918
2580	0.667	0.933
2581	0.678	0.913
2582	0.677	0.921
2583	0.682	0.918

2584	0.679	0.923
2585	0.662	0.919
2586	0.686	0.930
2587	0.679	0.917
2588	0.694	0.926
2589	0.711	0.910
2590	0.697	0.929
2591	0.664	0.917
2592	0.670	0.925
2593	0.666	0.929
2594	0.711	0.929
2595	0.662	0.931
2596	0.688	0.911
2597	0.652	0.908
2598	0.658	0.920
2599	0.687	0.919
2600	0.653	0.921
2601	0.673	0.924
2602	0.671	0.919
2603	0.686	0.914
2604	0.661	0.927
2605	0.621	0.925
2606	0.681	0.924
2607	0.642	0.918
2608	0.664	0.920
2609	0.671	0.911
2610	0.656	0.924
2611	0.662	0.930
2612	0.689	0.932
2613	0.648	0.930
2614	0.652	0.927
2615	0.652	0.913
2616	0.687	0.923
2617	0.689	0.917
2618	0.737	0.930
2619	0.634	0.912
2620	0.715	0.919
2621	0.679	0.927
2622	0.637	0.930

2623	0.684	0.926
2624	0.666	0.932
2625	0.685	0.926
2626	0.682	0.918
2627	0.693	0.934
2628	0.699	0.922
2629	0.680	0.930
2630	0.650	0.924
2631	0.685	0.916
2632	0.709	0.932
2633	0.692	0.909
2634	0.673	0.933
2635	0.687	0.912
2636	0.650	0.915
2637	0.723	0.916
2638	0.671	0.916
2639	0.719	0.934
2640	0.691	0.901
2641	0.666	0.926
2642	0.652	0.932
2643	0.642	0.917
2644	0.685	0.917
2645	0.679	0.927
2646	0.682	0.923
2647	0.687	0.924
2648	0.681	0.920
2649	0.701	0.930
2650	0.674	0.930
2651	0.667	0.928
2652	0.708	0.927
2653	0.673	0.919
2654	0.687	0.915
2655	0.708	0.922
2656	0.654	0.922
2657	0.656	0.918
2658	0.666	0.929
2659	0.700	0.919
2660	0.675	0.922
2661	0.690	0.918

2662	0.671	0.918
2663	0.712	0.920
2664	0.670	0.922
2665	0.646	0.919
2666	0.677	0.933
2667	0.721	0.920
2668	0.681	0.928
2669	0.658	0.924
2670	0.665	0.918
2671	0.662	0.918
2672	0.678	0.914
2673	0.666	0.919
2674	0.641	0.918
2675	0.631	0.920
2676	0.662	0.908
2677	0.717	0.925
2678	0.648	0.925
2679	0.655	0.926
2680	0.643	0.927
2681	0.672	0.918
2682	0.672	0.924
2683	0.683	0.920
2684	0.647	0.925
2685	0.686	0.926
2686	0.681	0.923
2687	0.694	0.927
2688	0.694	0.926
2689	0.681	0.911
2690	0.654	0.921
2691	0.683	0.920
2692	0.710	0.916
2693	0.674	0.926
2694	0.668	0.929
2695	0.684	0.910
2696	0.682	0.917
2697	0.667	0.924
2698	0.675	0.920
2699	0.697	0.933
2700	0.670	0.926

2701	0.666	0.913
2702	0.701	0.924
2703	0.686	0.918
2704	0.686	0.925
2705	0.701	0.912
2706	0.595	0.915
2707	0.691	0.921
2708	0.650	0.916
2709	0.674	0.908
2710	0.676	0.933
2711	0.659	0.931
2712	0.652	0.919
2713	0.659	0.926
2714	0.659	0.919
2715	0.662	0.935
2716	0.685	0.919
2717	0.682	0.929
2718	0.654	0.920
2719	0.667	0.925
2720	0.690	0.903
2721	0.642	0.921
2722	0.662	0.928
2723	0.671	0.914
2724	0.640	0.916
2725	0.680	0.917
2726	0.700	0.931
2727	0.670	0.923
2728	0.629	0.923
2729	0.674	0.924
2730	0.636	0.937
2731	0.678	0.921
2732	0.691	0.939
2733	0.675	0.926
2734	0.646	0.933
2735	0.691	0.924
2736	0.693	0.921
2737	0.748	0.915
2738	0.667	0.935
2739	0.702	0.931

2740	0.698	0.926
2741	0.673	0.929
2742	0.702	0.926
2743	0.716	0.936
2744	0.673	0.920
2745	0.683	0.932
2746	0.669	0.920
2747	0.744	0.933
2748	0.697	0.930
2749	0.682	0.929
2750	0.663	0.924
2751	0.694	0.937
2752	0.650	0.921
2753	0.709	0.927
2754	0.661	0.935
2755	0.668	0.922
2756	0.716	0.922
2757	0.704	0.923
2758	0.666	0.922
2759	0.653	0.916
2760	0.679	0.926
2761	0.698	0.919
2762	0.683	0.925
2763	0.634	0.916
2764	0.665	0.922
2765	0.689	0.922
2766	0.689	0.911
2767	0.697	0.937
2768	0.670	0.928
2769	0.664	0.922
2770	0.686	0.922
2771	0.673	0.926
2772	0.667	0.930
2773	0.681	0.924
2774	0.687	0.915
2775	0.673	0.927
2776	0.679	0.906
2777	0.693	0.934
2778	0.628	0.923

2779	0.707	0.927
2780	0.695	0.920
2781	0.686	0.930
2782	0.715	0.918
2783	0.714	0.924
2784	0.702	0.918
2785	0.661	0.924
2786	0.691	0.929
2787	0.671	0.927
2788	0.683	0.924
2789	0.685	0.922
2790	0.632	0.926
2791	0.694	0.915
2792	0.636	0.924
2793	0.722	0.915
2794	0.688	0.926
2795	0.667	0.922
2796	0.677	0.926
2797	0.647	0.920
2798	0.679	0.918
2799	0.661	0.922
2800	0.691	0.933
2801	0.662	0.923
2802	0.689	0.928
2803	0.664	0.933
2804	0.590	0.928
2805	0.697	0.928
2806	0.657	0.928
2807	0.679	0.917
2808	0.687	0.923
2809	0.639	0.921
2810	0.672	0.920
2811	0.693	0.933
2812	0.628	0.919
2813	0.685	0.925
2814	0.693	0.923
2815	0.712	0.915
2816	0.663	0.920
2817	0.687	0.920

2818	0.705	0.938
2819	0.703	0.916
2820	0.701	0.922
2821	0.670	0.925
2822	0.686	0.928
2823	0.664	0.935
2824	0.712	0.920
2825	0.706	0.912
2826	0.653	0.916
2827	0.650	0.921
2828	0.671	0.922
2829	0.665	0.924
2830	0.671	0.917
2831	0.686	0.909
2832	0.689	0.925
2833	0.663	0.927
2834	0.705	0.921
2835	0.709	0.924
2836	0.706	0.926
2837	0.643	0.911
2838	0.718	0.922
2839	0.713	0.922
2840	0.695	0.914
2841	0.682	0.932
2842	0.677	0.921
2843	0.660	0.927
2844	0.726	0.935
2845	0.698	0.922
2846	0.662	0.927
2847	0.693	0.922
2848	0.705	0.926
2849	0.665	0.921
2850	0.661	0.928
2851	0.655	0.931
2852	0.712	0.916
2853	0.675	0.920
2854	0.713	0.921
2855	0.653	0.924
2856	0.720	0.923

2857	0.680	0.922
2858	0.655	0.918
2859	0.687	0.924
2860	0.683	0.928
2861	0.644	0.932
2862	0.735	0.936
2863	0.685	0.925
2864	0.659	0.921
2865	0.658	0.932
2866	0.665	0.922
2867	0.676	0.922
2868	0.718	0.918
2869	0.717	0.918
2870	0.706	0.919
2871	0.670	0.913
2872	0.643	0.921
2873	0.651	0.920
2874	0.664	0.918
2875	0.702	0.914
2876	0.693	0.922
2877	0.660	0.915
2878	0.660	0.919
2879	0.670	0.920
2880	0.678	0.926
2881	0.658	0.916
2882	0.740	0.925
2883	0.656	0.925
2884	0.647	0.929
2885	0.695	0.919
2886	0.675	0.918
2887	0.670	0.932
2888	0.641	0.923
2889	0.661	0.918
2890	0.676	0.911
2891	0.606	0.924
2892	0.663	0.922
2893	0.672	0.930
2894	0.670	0.919
2895	0.690	0.924

2896	0.674	0.932
2897	0.688	0.917
2898	0.652	0.927
2899	0.684	0.935
2900	0.653	0.910
2901	0.681	0.929
2902	0.686	0.922
2903	0.704	0.925
2904	0.670	0.921
2905	0.636	0.925
2906	0.624	0.926
2907	0.649	0.920
2908	0.652	0.926
2909	0.643	0.931
2910	0.674	0.925
2911	0.669	0.927
2912	0.662	0.908
2913	0.668	0.922
2914	0.687	0.924
2915	0.683	0.923
2916	0.667	0.917
2917	0.662	0.927
2918	0.723	0.919
2919	0.655	0.922
2920	0.668	0.919
2921	0.624	0.935
2922	0.640	0.930
2923	0.681	0.914
2924	0.674	0.929
2925	0.736	0.912
2926	0.684	0.920
2927	0.673	0.918
2928	0.693	0.929
2929	0.676	0.928
2930	0.655	0.927
2931	0.667	0.924
2932	0.652	0.930
2933	0.658	0.916
2934	0.666	0.934

2935	0.713	0.914
2936	0.664	0.924
2937	0.679	0.926
2938	0.658	0.923
2939	0.698	0.919
2940	0.646	0.911
2941	0.701	0.917
2942	0.693	0.919
2943	0.666	0.925
2944	0.661	0.927
2945	0.681	0.912
2946	0.662	0.916
2947	0.695	0.923
2948	0.676	0.926
2949	0.662	0.923
2950	0.643	0.942
2951	0.708	0.939
2952	0.626	0.919
2953	0.656	0.937
2954	0.691	0.923
2955	0.691	0.929
2956	0.744	0.921
2957	0.688	0.933
2958	0.686	0.921
2959	0.693	0.916
2960	0.666	0.934
2961	0.648	0.924
2962	0.678	0.935
2963	0.708	0.925
2964	0.687	0.932
2965	0.684	0.916
2966	0.737	0.927
2967	0.686	0.920
2968	0.648	0.922
2969	0.671	0.927
2970	0.625	0.924
2971	0.651	0.917
2972	0.648	0.921
2973	0.694	0.923

2974	0.670	0.934
2975	0.732	0.928
2976	0.684	0.924
2977	0.725	0.908
2978	0.690	0.918
2979	0.615	0.926
2980	0.670	0.934
2981	0.661	0.925
2982	0.691	0.910
2983	0.666	0.927
2984	0.635	0.925
2985	0.707	0.929
2986	0.712	0.934
2987	0.643	0.907
2988	0.695	0.921
2989	0.647	0.924
2990	0.661	0.920
2991	0.705	0.931
2992	0.677	0.934
2993	0.703	0.923
2994	0.681	0.923
2995	0.671	0.919
2996	0.645	0.921
2997	0.666	0.919
2998	0.626	0.923
2999	0.677	0.915
3000	0.658	0.924
3001	0.681	0.924
3002	0.663	0.924
3003	0.645	0.914
3004	0.666	0.917
3005	0.674	0.929
3006	0.673	0.920
3007	0.690	0.927
3008	0.681	0.917
3009	0.697	0.916
3010	0.651	0.919
3011	0.693	0.921
3012	0.679	0.919

3013	0.685	0.930
3014	0.678	0.924
3015	0.647	0.921
3016	0.665	0.935
3017	0.682	0.934
3018	0.633	0.924
3019	0.674	0.922
3020	0.655	0.938
3021	0.659	0.915
3022	0.662	0.923
3023	0.624	0.918
3024	0.670	0.934
3025	0.670	0.924
3026	0.689	0.929
3027	0.638	0.920
3028	0.639	0.931
3029	0.658	0.928
3030	0.667	0.922
3031	0.728	0.916
3032	0.686	0.921
3033	0.669	0.910
3034	0.655	0.925
3035	0.697	0.919
3036	0.653	0.922
3037	0.702	0.922
3038	0.666	0.918
3039	0.703	0.915
3040	0.658	0.926
3041	0.654	0.934
3042	0.691	0.918
3043	0.708	0.931
3044	0.652	0.930
3045	0.661	0.921
3046	0.663	0.927
3047	0.659	0.917
3048	0.682	0.925
3049	0.654	0.928
3050	0.664	0.927
3051	0.668	0.914

3052	0.695	0.917
3053	0.677	0.926
3054	0.635	0.927
3055	0.643	0.929
3056	0.663	0.923
3057	0.683	0.923
3058	0.697	0.918
3059	0.670	0.921
3060	0.643	0.919
3061	0.658	0.930
3062	0.630	0.935
3063	0.670	0.910
3064	0.713	0.915
3065	0.709	0.918
3066	0.687	0.918
3067	0.700	0.928
3068	0.702	0.920
3069	0.671	0.918
3070	0.642	0.919
3071	0.698	0.925
3072	0.702	0.919
3073	0.662	0.925
3074	0.643	0.922
3075	0.667	0.919
3076	0.674	0.923
3077	0.662	0.920
3078	0.676	0.908
3079	0.663	0.928
3080	0.666	0.920
3081	0.675	0.924
3082	0.689	0.909
3083	0.702	0.917
3084	0.690	0.919
3085	0.670	0.931
3086	0.655	0.926
3087	0.612	0.916
3088	0.667	0.918
3089	0.668	0.921
3090	0.645	0.915

3091	0.650	0.925
3092	0.693	0.923
3093	0.647	0.928
3094	0.652	0.930
3095	0.709	0.919
3096	0.664	0.923
3097	0.677	0.920
3098	0.693	0.927
3099	0.629	0.925
3100	0.662	0.926
3101	0.654	0.917
3102	0.664	0.928
3103	0.692	0.940
3104	0.668	0.924
3105	0.637	0.924
3106	0.655	0.913
3107	0.679	0.921
3108	0.673	0.927
3109	0.685	0.916
3110	0.698	0.923
3111	0.657	0.931
3112	0.694	0.925
3113	0.654	0.919
3114	0.664	0.920
3115	0.681	0.927
3116	0.663	0.920
3117	0.693	0.916
3118	0.685	0.928
3119	0.692	0.923
3120	0.639	0.924
3121	0.674	0.907
3122	0.661	0.931
3123	0.651	0.923
3124	0.705	0.927
3125	0.677	0.913
3126	0.725	0.926
3127	0.670	0.919
3128	0.678	0.932
3129	0.616	0.932

3130	0.699	0.926
3131	0.704	0.918
3132	0.672	0.921
3133	0.666	0.931
3134	0.716	0.917
3135	0.653	0.930
3136	0.656	0.925
3137	0.678	0.913
3138	0.620	0.913
3139	0.695	0.928
3140	0.677	0.921
3141	0.668	0.919
3142	0.694	0.920
3143	0.675	0.914
3144	0.662	0.927
3145	0.678	0.912
3146	0.658	0.921
3147	0.696	0.920
3148	0.690	0.919
3149	0.656	0.932
3150	0.656	0.922
3151	0.627	0.929
3152	0.689	0.928
3153	0.678	0.925
3154	0.685	0.921
3155	0.677	0.924
3156	0.695	0.929
3157	0.686	0.925
3158	0.617	0.928
3159	0.694	0.926
3160	0.670	0.915
3161	0.669	0.924
3162	0.696	0.930
3163	0.668	0.916
3164	0.716	0.930
3165	0.682	0.927
3166	0.659	0.926
3167	0.681	0.930
3168	0.708	0.921

3169	0.675	0.919
3170	0.673	0.922
3171	0.655	0.934
3172	0.629	0.925
3173	0.668	0.925
3174	0.681	0.919
3175	0.697	0.917
3176	0.729	0.921
3177	0.708	0.923
3178	0.653	0.915
3179	0.651	0.915
3180	0.692	0.922
3181	0.670	0.921
3182	0.678	0.926
3183	0.675	0.918
3184	0.669	0.920
3185	0.704	0.917
3186	0.673	0.927
3187	0.693	0.915
3188	0.689	0.934
3189	0.654	0.922
3190	0.640	0.905
3191	0.696	0.922
3192	0.701	0.926
3193	0.682	0.925
3194	0.701	0.911
3195	0.651	0.921
3196	0.693	0.927
3197	0.732	0.921
3198	0.655	0.932
3199	0.671	0.923
3200	0.678	0.923
3201	0.675	0.925
3202	0.675	0.912
3203	0.698	0.906
3204	0.683	0.931
3205	0.666	0.921
3206	0.687	0.922
3207	0.661	0.922

3208	0.705	0.924
3209	0.670	0.913
3210	0.644	0.920
3211	0.666	0.929
3212	0.682	0.923
3213	0.640	0.917
3214	0.675	0.908
3215	0.692	0.923
3216	0.670	0.921
3217	0.647	0.940
3218	0.712	0.918
3219	0.663	0.930
3220	0.656	0.920
3221	0.677	0.917
3222	0.694	0.921
3223	0.685	0.909
3224	0.671	0.919
3225	0.700	0.911
3226	0.678	0.905
3227	0.697	0.922
3228	0.689	0.923
3229	0.659	0.921
3230	0.679	0.916
3231	0.696	0.916
3232	0.712	0.921
3233	0.670	0.926
3234	0.691	0.916
3235	0.648	0.931
3236	0.660	0.920
3237	0.666	0.917
3238	0.700	0.913
3239	0.705	0.929
3240	0.681	0.908
3241	0.697	0.919
3242	0.666	0.920
3243	0.685	0.935
3244	0.658	0.930
3245	0.675	0.930
3246	0.658	0.914

3247	0.704	0.928
3248	0.660	0.938
3249	0.639	0.912
3250	0.646	0.920
3251	0.700	0.917
3252	0.697	0.916
3253	0.671	0.927
3254	0.622	0.925
3255	0.682	0.919
3256	0.651	0.913
3257	0.644	0.919
3258	0.690	0.917
3259	0.658	0.911
3260	0.616	0.921
3261	0.647	0.922
3262	0.705	0.916
3263	0.683	0.924
3264	0.686	0.922
3265	0.658	0.913
3266	0.704	0.932
3267	0.623	0.926
3268	0.636	0.928
3269	0.639	0.920
3270	0.708	0.916
3271	0.634	0.915
3272	0.689	0.929
3273	0.666	0.922
3274	0.671	0.940
3275	0.674	0.915
3276	0.681	0.909
3277	0.666	0.917
3278	0.691	0.931
3279	0.661	0.929
3280	0.649	0.934
3281	0.668	0.908
3282	0.692	0.900
3283	0.629	0.933
3284	0.728	0.927
3285	0.642	0.936

3286	0.679	0.921
3287	0.684	0.928
3288	0.635	0.925
3289	0.673	0.920
3290	0.700	0.914
3291	0.689	0.923
3292	0.684	0.921
3293	0.627	0.924
3294	0.698	0.924
3295	0.682	0.921
3296	0.689	0.934
3297	0.660	0.922
3298	0.616	0.923
3299	0.656	0.921
3300	0.644	0.926
3301	0.667	0.922
3302	0.701	0.915
3303	0.681	0.922
3304	0.685	0.930
3305	0.692	0.915
3306	0.692	0.929
3307	0.658	0.920
3308	0.623	0.930
3309	0.682	0.926
3310	0.659	0.927
3311	0.701	0.919
3312	0.669	0.920
3313	0.673	0.924
3314	0.660	0.915
3315	0.670	0.923
3316	0.679	0.926
3317	0.690	0.921
3318	0.680	0.923
3319	0.700	0.914
3320	0.677	0.902
3321	0.702	0.918
3322	0.644	0.922
3323	0.633	0.922
3324	0.654	0.920

3325	0.689	0.915
3326	0.707	0.919
3327	0.690	0.918
3328	0.683	0.915
3329	0.621	0.923
3330	0.647	0.927
3331	0.676	0.916
3332	0.665	0.921
3333	0.719	0.916
3334	0.677	0.914
3335	0.650	0.924
3336	0.707	0.924
3337	0.676	0.916
3338	0.649	0.905
3339	0.651	0.920
3340	0.649	0.931
3341	0.658	0.916
3342	0.660	0.927
3343	0.660	0.914
3344	0.658	0.910
3345	0.702	0.917
3346	0.656	0.917
3347	0.654	0.928
3348	0.705	0.926
3349	0.746	0.927
3350	0.670	0.933
3351	0.702	0.923
3352	0.636	0.909
3353	0.654	0.913
3354	0.683	0.920
3355	0.687	0.908
3356	0.651	0.931
3357	0.664	0.920
3358	0.674	0.931
3359	0.669	0.908
3360	0.704	0.915
3361	0.690	0.915
3362	0.669	0.929
3363	0.660	0.921

3364	0.649	0.918
3365	0.672	0.924
3366	0.663	0.920
3367	0.688	0.921
3368	0.682	0.925
3369	0.606	0.914
3370	0.660	0.914
3371	0.651	0.930
3372	0.666	0.915
3373	0.667	0.917
3374	0.666	0.931
3375	0.674	0.927
3376	0.657	0.921
3377	0.635	0.925
3378	0.661	0.924
3379	0.693	0.922
3380	0.700	0.922
3381	0.690	0.925
3382	0.678	0.922
3383	0.680	0.935
3384	0.661	0.914
3385	0.701	0.926
3386	0.644	0.917
3387	0.641	0.927
3388	0.667	0.919
3389	0.665	0.920
3390	0.643	0.925
3391	0.646	0.919
3392	0.654	0.915
3393	0.703	0.922
3394	0.673	0.929
3395	0.708	0.914
3396	0.681	0.930
3397	0.625	0.935
3398	0.701	0.919
3399	0.659	0.916
3400	0.681	0.936
3401	0.673	0.913
3402	0.678	0.927

3403	0.675	0.919
3404	0.678	0.916
3405	0.659	0.921
3406	0.710	0.927
3407	0.679	0.930
3408	0.693	0.919
3409	0.660	0.920
3410	0.683	0.921
3411	0.659	0.917
3412	0.638	0.920
3413	0.700	0.904
3414	0.690	0.926
3415	0.645	0.914
3416	0.687	0.921
3417	0.662	0.923
3418	0.695	0.915
3419	0.653	0.911
3420	0.696	0.917
3421	0.666	0.933
3422	0.660	0.922
3423	0.679	0.930
3424	0.680	0.920
3425	0.658	0.918
3426	0.644	0.913
3427	0.630	0.914
3428	0.679	0.940
3429	0.689	0.915
3430	0.670	0.914
3431	0.669	0.915
3432	0.692	0.926
3433	0.662	0.917
3434	0.616	0.916
3435	0.670	0.915
3436	0.663	0.930
3437	0.654	0.924
3438	0.672	0.927
3439	0.654	0.942
3440	0.655	0.934
3441	0.677	0.921

3442	0.694	0.923
3443	0.698	0.916
3444	0.666	0.924
3445	0.653	0.925
3446	0.634	0.917
3447	0.677	0.913
3448	0.632	0.923
3449	0.653	0.929
3450	0.663	0.917
3451	0.671	0.906
3452	0.703	0.909
3453	0.685	0.922
3454	0.652	0.938
3455	0.632	0.909
3456	0.659	0.931
3457	0.693	0.922
3458	0.694	0.923
3459	0.697	0.925
3460	0.657	0.929
3461	0.648	0.926
3462	0.724	0.928
3463	0.690	0.931
3464	0.655	0.925
3465	0.643	0.921
3466	0.692	0.914
3467	0.683	0.915
3468	0.670	0.922
3469	0.652	0.920
3470	0.679	0.927
3471	0.732	0.928
3472	0.669	0.924
3473	0.649	0.913
3474	0.673	0.914
3475	0.662	0.921
3476	0.693	0.919
3477	0.677	0.931
3478	0.712	0.926
3479	0.666	0.905
3480	0.704	0.935

3481	0.662	0.924
3482	0.653	0.919
3483	0.689	0.924
3484	0.675	0.928
3485	0.670	0.912
3486	0.680	0.923
3487	0.690	0.926
3488	0.666	0.918
3489	0.676	0.914
3490	0.701	0.925
3491	0.708	0.926
3492	0.697	0.925
3493	0.647	0.923
3494	0.675	0.932
3495	0.666	0.912
3496	0.670	0.923
3497	0.673	0.918
3498	0.635	0.916
3499	0.606	0.922
3500	0.681	0.918
3501	0.648	0.916
3502	0.649	0.925
3503	0.674	0.903
3504	0.675	0.918
3505	0.700	0.918
3506	0.636	0.912
3507	0.700	0.925
3508	0.622	0.928
3509	0.637	0.920
3510	0.674	0.903
3511	0.661	0.914
3512	0.690	0.915
3513	0.646	0.920
3514	0.646	0.923
3515	0.693	0.916
3516	0.685	0.925
3517	0.686	0.927
3518	0.658	0.907
3519	0.629	0.925

3520	0.691	0.912
3521	0.637	0.926
3522	0.666	0.918
3523	0.624	0.915
3524	0.669	0.924
3525	0.675	0.920
3526	0.670	0.928
3527	0.661	0.925
3528	0.647	0.911
3529	0.674	0.922
3530	0.698	0.926
3531	0.690	0.927
3532	0.626	0.928
3533	0.591	0.916
3534	0.648	0.913
3535	0.682	0.921
3536	0.676	0.916
3537	0.693	0.914
3538	0.717	0.924
3539	0.702	0.918
3540	0.676	0.926
3541	0.681	0.935
3542	0.677	0.915
3543	0.663	0.903
3544	0.667	0.919
3545	0.679	0.914
3546	0.694	0.927
3547	0.697	0.932
3548	0.642	0.921
3549	0.661	0.919
3550	0.708	0.900
3551	0.701	0.922
3552	0.665	0.922
3553	0.706	0.916
3554	0.635	0.909
3555	0.707	0.915
3556	0.711	0.916
3557	0.684	0.920
3558	0.653	0.913

3559	0.668	0.921
3560	0.691	0.915
3561	0.662	0.917
3562	0.702	0.914
3563	0.659	0.918
3564	0.702	0.932
3565	0.685	0.908
3566	0.666	0.920
3567	0.637	0.918
3568	0.682	0.913
3569	0.692	0.929
3570	0.672	0.930
3571	0.633	0.927
3572	0.657	0.922
3573	0.656	0.918
3574	0.659	0.914
3575	0.652	0.925
3576	0.669	0.924
3577	0.643	0.917
3578	0.680	0.922
3579	0.674	0.920
3580	0.705	0.913
3581	0.688	0.922
3582	0.642	0.931
3583	0.658	0.918
3584	0.698	0.906
3585	0.645	0.912
3586	0.646	0.924
3587	0.650	0.912
3588	0.650	0.926
3589	0.662	0.926
3590	0.660	0.923
3591	0.698	0.924
3592	0.664	0.923
3593	0.690	0.926
3594	0.652	0.923
3595	0.679	0.924
3596	0.638	0.921
3597	0.713	0.920

3598	0.643	0.921
3599	0.679	0.922
3600	0.685	0.918
3601	0.662	0.929
3602	0.705	0.922
3603	0.662	0.919
3604	0.651	0.923
3605	0.704	0.915
3606	0.673	0.921
3607	0.635	0.920
3608	0.639	0.926
3609	0.684	0.911
3610	0.653	0.917
3611	0.658	0.917
3612	0.640	0.911
3613	0.674	0.919
3614	0.705	0.916
3615	0.693	0.918
3616	0.682	0.910
3617	0.740	0.926
3618	0.642	0.920
3619	0.642	0.915
3620	0.699	0.915
3621	0.695	0.923
3622	0.675	0.927
3623	0.671	0.924
3624	0.639	0.915
3625	0.668	0.921
3626	0.683	0.925
3627	0.636	0.907
3628	0.640	0.926
3629	0.693	0.915
3630	0.663	0.932
3631	0.658	0.915
3632	0.663	0.912
3633	0.644	0.921
3634	0.652	0.926
3635	0.659	0.920
3636	0.693	0.927

3637	0.677	0.919
3638	0.666	0.922
3639	0.664	0.916
3640	0.662	0.915
3641	0.715	0.923
3642	0.663	0.911
3643	0.666	0.932
3644	0.667	0.912
3645	0.656	0.923
3646	0.645	0.920
3647	0.665	0.920
3648	0.663	0.924
3649	0.667	0.926
3650	0.681	0.934
3651	0.690	0.915
3652	0.666	0.925
3653	0.673	0.924
3654	0.698	0.906
3655	0.681	0.921
3656	0.655	0.917
3657	0.692	0.916
3658	0.649	0.918
3659	0.670	0.912
3660	0.688	0.924
3661	0.673	0.930
3662	0.658	0.926
3663	0.677	0.918
3664	0.663	0.902
3665	0.669	0.927
3666	0.665	0.914
3667	0.658	0.915
3668	0.655	0.919
3669	0.685	0.916
3670	0.650	0.933
3671	0.678	0.926
3672	0.672	0.918
3673	0.672	0.929
3674	0.639	0.914
3675	0.679	0.908

3676	0.675	0.926
3677	0.681	0.918
3678	0.673	0.928
3679	0.701	0.919
3680	0.658	0.935
3681	0.624	0.912
3682	0.695	0.927
3683	0.693	0.921
3684	0.702	0.905
3685	0.644	0.911
3686	0.694	0.910
3687	0.670	0.928
3688	0.671	0.914
3689	0.647	0.926
3690	0.643	0.930
3691	0.658	0.921
3692	0.672	0.912
3693	0.677	0.920
3694	0.712	0.920
3695	0.681	0.923
3696	0.687	0.924
3697	0.678	0.929
3698	0.662	0.923
3699	0.705	0.927
3700	0.650	0.934
3701	0.644	0.918
3702	0.704	0.928
3703	0.683	0.908
3704	0.710	0.911
3705	0.695	0.913
3706	0.638	0.914
3707	0.654	0.927
3708	0.651	0.915
3709	0.654	0.925
3710	0.694	0.923
3711	0.666	0.907
3712	0.647	0.919
3713	0.704	0.911
3714	0.648	0.923

3715	0.729	0.926
3716	0.702	0.911
3717	0.671	0.919
3718	0.693	0.914
3719	0.692	0.927
3720	0.665	0.920
3721	0.631	0.922
3722	0.677	0.917
3723	0.679	0.915
3724	0.665	0.903
3725	0.665	0.918
3726	0.644	0.921
3727	0.664	0.926
3728	0.625	0.924
3729	0.656	0.916
3730	0.655	0.923
3731	0.669	0.914
3732	0.653	0.915
3733	0.712	0.911
3734	0.675	0.923
3735	0.678	0.910
3736	0.674	0.921
3737	0.701	0.901
3738	0.656	0.925
3739	0.685	0.925
3740	0.618	0.925
3741	0.670	0.924
3742	0.692	0.910
3743	0.659	0.918
3744	0.701	0.916
3745	0.693	0.920
3746	0.694	0.912
3747	0.679	0.917
3748	0.641	0.914
3749	0.698	0.920
3750	0.656	0.922
3751	0.669	0.917
3752	0.643	0.932
3753	0.626	0.929

3754	0.653	0.921
3755	0.651	0.922
3756	0.665	0.926
3757	0.670	0.912
3758	0.675	0.914
3759	0.633	0.917
3760	0.698	0.923
3761	0.643	0.913
3762	0.702	0.909
3763	0.656	0.918
3764	0.652	0.925
3765	0.674	0.918
3766	0.670	0.920
3767	0.658	0.922
3768	0.656	0.906
3769	0.623	0.928
3770	0.678	0.922
3771	0.662	0.915
3772	0.647	0.926
3773	0.671	0.917
3774	0.689	0.915
3775	0.668	0.914
3776	0.655	0.930
3777	0.645	0.905
3778	0.670	0.931
3779	0.629	0.933
3780	0.689	0.932
3781	0.630	0.917
3782	0.677	0.924
3783	0.676	0.925
3784	0.656	0.922
3785	0.693	0.929
3786	0.681	0.929
3787	0.694	0.931
3788	0.666	0.928
3789	0.647	0.919
3790	0.658	0.924
3791	0.635	0.918
3792	0.679	0.922

3793	0.644	0.916
3794	0.711	0.924
3795	0.646	0.917
3796	0.681	0.917
3797	0.630	0.915
3798	0.667	0.916
3799	0.643	0.922
3800	0.690	0.923
3801	0.639	0.922
3802	0.688	0.928
3803	0.675	0.925
3804	0.688	0.928
3805	0.631	0.929
3806	0.677	0.921
3807	0.690	0.918
3808	0.661	0.931
3809	0.639	0.920
3810	0.639	0.915
3811	0.660	0.924
3812	0.647	0.929
3813	0.689	0.931
3814	0.671	0.918
3815	0.674	0.921
3816	0.654	0.912
3817	0.655	0.912
3818	0.702	0.910
3819	0.592	0.918
3820	0.643	0.917
3821	0.629	0.910
3822	0.656	0.917
3823	0.723	0.917
3824	0.672	0.920
3825	0.693	0.926
3826	0.641	0.921
3827	0.654	0.915
3828	0.650	0.912
3829	0.702	0.933
3830	0.659	0.910
3831	0.627	0.927

3832	0.674	0.913
3833	0.671	0.915
3834	0.705	0.928
3835	0.683	0.918
3836	0.687	0.924
3837	0.643	0.922
3838	0.685	0.907
3839	0.647	0.908
3840	0.669	0.920
3841	0.651	0.920
3842	0.674	0.913
3843	0.667	0.921
3844	0.705	0.893
3845	0.697	0.907
3846	0.695	0.906
3847	0.654	0.924
3848	0.667	0.915
3849	0.684	0.921
3850	0.609	0.907
3851	0.631	0.920
3852	0.655	0.920
3853	0.658	0.922
3854	0.681	0.920
3855	0.670	0.935
3856	0.680	0.920
3857	0.677	0.925
3858	0.663	0.923
3859	0.648	0.918
3860	0.655	0.925
3861	0.608	0.928
3862	0.682	0.914
3863	0.666	0.922
3864	0.639	0.914
3865	0.615	0.922
3866	0.691	0.910
3867	0.693	0.930
3868	0.626	0.914
3869	0.650	0.915
3870	0.685	0.919

3871	0.681	0.923
3872	0.662	0.917
3873	0.646	0.925
3874	0.635	0.913
3875	0.647	0.929
3876	0.671	0.932
3877	0.662	0.917
3878	0.653	0.928
3879	0.647	0.920
3880	0.622	0.934
3881	0.630	0.920
3882	0.678	0.908
3883	0.629	0.914
3884	0.658	0.920
3885	0.710	0.903
3886	0.652	0.916
3887	0.659	0.917
3888	0.652	0.922
3889	0.652	0.922
3890	0.678	0.916
3891	0.697	0.909
3892	0.678	0.900
3893	0.660	0.933
3894	0.647	0.921
3895	0.695	0.911
3896	0.683	0.912
3897	0.697	0.921
3898	0.669	0.918
3899	0.620	0.909
3900	0.631	0.918
3901	0.687	0.924
3902	0.648	0.919
3903	0.643	0.922
3904	0.702	0.915
3905	0.685	0.907
3906	0.667	0.928
3907	0.716	0.911
3908	0.671	0.911
3909	0.679	0.910

3910	0.666	0.923
3911	0.643	0.925
3912	0.647	0.921
3913	0.678	0.919
3914	0.650	0.924
3915	0.640	0.921
3916	0.681	0.918
3917	0.670	0.923
3918	0.648	0.919
3919	0.674	0.913
3920	0.684	0.902
3921	0.635	0.916
3922	0.648	0.920
3923	0.662	0.920
3924	0.689	0.929
3925	0.674	0.929
3926	0.689	0.920
3927	0.689	0.922
3928	0.679	0.916
3929	0.659	0.918
3930	0.667	0.901
3931	0.697	0.915
3932	0.677	0.923
3933	0.692	0.922
3934	0.630	0.924
3935	0.667	0.921
3936	0.678	0.920
3937	0.708	0.920
3938	0.641	0.919
3939	0.660	0.928
3940	0.629	0.916
3941	0.645	0.915
3942	0.669	0.921
3943	0.682	0.920
3944	0.690	0.915
3945	0.678	0.925
3946	0.630	0.921
3947	0.647	0.928
3948	0.677	0.935

3949	0.634	0.929
3950	0.642	0.925
3951	0.662	0.916
3952	0.641	0.920
3953	0.681	0.928
3954	0.682	0.920
3955	0.620	0.911
3956	0.648	0.916
3957	0.662	0.922
3958	0.694	0.919
3959	0.652	0.911
3960	0.671	0.910
3961	0.635	0.903
3962	0.678	0.918
3963	0.656	0.915
3964	0.646	0.916
3965	0.655	0.921
3966	0.678	0.916
3967	0.631	0.916
3968	0.694	0.916
3969	0.687	0.928
3970	0.647	0.939
3971	0.675	0.918
3972	0.714	0.904
3973	0.693	0.914
3974	0.622	0.911
3975	0.672	0.922
3976	0.681	0.920
3977	0.666	0.914
3978	0.678	0.918
3979	0.638	0.910
3980	0.686	0.921
3981	0.676	0.925
3982	0.648	0.903
3983	0.718	0.919
3984	0.685	0.927
3985	0.684	0.926
3986	0.646	0.912
3987	0.666	0.926

3988	0.659	0.927
3989	0.676	0.916
3990	0.665	0.925
3991	0.679	0.916
3992	0.691	0.917
3993	0.675	0.921
3994	0.659	0.925
3995	0.696	0.920
3996	0.643	0.921
3997	0.658	0.922
3998	0.640	0.917
3999	0.683	0.920
4000	0.640	0.917
4001	0.607	0.908
4002	0.640	0.913
4003	0.697	0.929
4004	0.724	0.925
4005	0.685	0.909
4006	0.713	0.908
4007	0.652	0.920
4008	0.662	0.928
4009	0.642	0.905
4010	0.678	0.928
4011	0.693	0.923
4012	0.663	0.910
4013	0.687	0.914
4014	0.687	0.914
4015	0.614	0.917
4016	0.652	0.914
4017	0.678	0.918
4018	0.676	0.915
4019	0.654	0.914
4020	0.661	0.926
4021	0.683	0.910
4022	0.670	0.919
4023	0.659	0.921
4024	0.675	0.930
4025	0.648	0.921
4026	0.677	0.912

4027	0.690	0.909
4028	0.603	0.913
4029	0.681	0.918
4030	0.664	0.921
4031	0.705	0.917
4032	0.689	0.921
4033	0.689	0.920
4034	0.667	0.921
4035	0.663	0.927
4036	0.673	0.919
4037	0.682	0.918
4038	0.707	0.918
4039	0.672	0.905
4040	0.663	0.913
4041	0.656	0.914
4042	0.654	0.916
4043	0.675	0.916
4044	0.662	0.913
4045	0.668	0.930
4046	0.686	0.914
4047	0.626	0.911
4048	0.652	0.921
4049	0.667	0.914
4050	0.689	0.926
4051	0.689	0.917
4052	0.643	0.933
4053	0.658	0.927
4054	0.655	0.909
4055	0.685	0.917
4056	0.685	0.929
4057	0.638	0.922
4058	0.679	0.924
4059	0.679	0.920
4060	0.700	0.920
4061	0.691	0.930
4062	0.685	0.913
4063	0.648	0.919
4064	0.670	0.916
4065	0.645	0.918

4066	0.658	0.923
4067	0.652	0.926
4068	0.668	0.922
4069	0.689	0.928
4070	0.639	0.914
4071	0.689	0.912
4072	0.683	0.911
4073	0.659	0.907
4074	0.629	0.908
4075	0.687	0.934
4076	0.659	0.938
4077	0.695	0.918
4078	0.651	0.923
4079	0.654	0.910
4080	0.647	0.922
4081	0.658	0.925
4082	0.672	0.914
4083	0.640	0.916
4084	0.608	0.907
4085	0.676	0.914
4086	0.674	0.917
4087	0.631	0.924
4088	0.671	0.915
4089	0.652	0.933
4090	0.633	0.930
4091	0.728	0.917
4092	0.682	0.911
4093	0.657	0.921
4094	0.660	0.920
4095	0.679	0.920
4096	0.671	0.918
4097	0.688	0.913
4098	0.686	0.923
4099	0.622	0.919
4100	0.662	0.907
4101	0.686	0.915
4102	0.656	0.919
4103	0.670	0.929
4104	0.639	0.921

4105	0.673	0.918
4106	0.678	0.911
4107	0.720	0.922
4108	0.648	0.930
4109	0.703	0.918
4110	0.661	0.930
4111	0.670	0.916
4112	0.666	0.924
4113	0.677	0.913
4114	0.678	0.924
4115	0.670	0.921
4116	0.666	0.917
4117	0.648	0.911
4118	0.671	0.917
4119	0.627	0.930
4120	0.659	0.915
4121	0.687	0.918
4122	0.662	0.923
4123	0.669	0.914
4124	0.668	0.921
4125	0.669	0.918
4126	0.658	0.923
4127	0.659	0.926
4128	0.649	0.916
4129	0.666	0.926
4130	0.666	0.923
4131	0.671	0.927
4132	0.698	0.908
4133	0.717	0.907
4134	0.673	0.908
4135	0.635	0.921
4136	0.678	0.921
4137	0.658	0.931
4138	0.656	0.925
4139	0.706	0.929
4140	0.672	0.925
4141	0.712	0.906
4142	0.645	0.919
4143	0.640	0.913

4144	0.652	0.910
4145	0.670	0.913
4146	0.704	0.910
4147	0.694	0.919
4148	0.638	0.922
4149	0.685	0.921
4150	0.689	0.932
4151	0.674	0.913
4152	0.679	0.924
4153	0.694	0.914
4154	0.653	0.915
4155	0.666	0.915
4156	0.650	0.924
4157	0.687	0.915
4158	0.663	0.918
4159	0.692	0.923
4160	0.652	0.917
4161	0.629	0.913
4162	0.672	0.929
4163	0.671	0.919
4164	0.706	0.920
4165	0.667	0.921
4166	0.669	0.930
4167	0.703	0.934
4168	0.637	0.923
4169	0.689	0.923
4170	0.621	0.920
4171	0.670	0.917
4172	0.647	0.921
4173	0.685	0.928
4174	0.687	0.921
4175	0.692	0.925
4176	0.662	0.929
4177	0.662	0.914
4178	0.658	0.908
4179	0.672	0.919
4180	0.689	0.925
4181	0.708	0.918
4182	0.665	0.913

4183	0.688	0.917
4184	0.670	0.929
4185	0.681	0.915
4186	0.675	0.927
4187	0.664	0.923
4188	0.662	0.927
4189	0.692	0.934
4190	0.679	0.911
4191	0.686	0.914
4192	0.674	0.915
4193	0.643	0.918
4194	0.692	0.931
4195	0.669	0.924
4196	0.643	0.918
4197	0.683	0.926
4198	0.646	0.921
4199	0.669	0.917
4200	0.693	0.914
4201	0.679	0.918
4202	0.701	0.911
4203	0.656	0.915
4204	0.656	0.909
4205	0.635	0.911
4206	0.655	0.915
4207	0.671	0.914
4208	0.647	0.922
4209	0.687	0.914
4210	0.687	0.923
4211	0.666	0.922
4212	0.663	0.916
4213	0.663	0.921
4214	0.618	0.928
4215	0.668	0.927
4216	0.672	0.913
4217	0.677	0.910
4218	0.632	0.913
4219	0.667	0.929
4220	0.700	0.923
4221	0.671	0.923

4222	0.681	0.919
4223	0.655	0.913
4224	0.703	0.911
4225	0.656	0.910
4226	0.681	0.913
4227	0.678	0.916
4228	0.674	0.917
4229	0.651	0.921
4230	0.663	0.916
4231	0.636	0.914
4232	0.660	0.932
4233	0.686	0.918
4234	0.635	0.925
4235	0.661	0.931
4236	0.626	0.914
4237	0.713	0.917
4238	0.682	0.918
4239	0.694	0.910
4240	0.659	0.915
4241	0.631	0.919
4242	0.705	0.927
4243	0.668	0.926
4244	0.719	0.922
4245	0.585	0.929
4246	0.674	0.914
4247	0.593	0.920
4248	0.658	0.918
4249	0.668	0.918
4250	0.667	0.895
4251	0.698	0.911
4252	0.686	0.913
4253	0.660	0.913
4254	0.662	0.902
4255	0.692	0.925
4256	0.662	0.915
4257	0.712	0.913
4258	0.660	0.926
4259	0.669	0.918
4260	0.670	0.920

4261	0.698	0.921
4262	0.681	0.917
4263	0.700	0.919
4264	0.655	0.925
4265	0.640	0.919
4266	0.663	0.908
4267	0.664	0.924
4268	0.660	0.919
4269	0.721	0.926
4270	0.657	0.920
4271	0.690	0.922
4272	0.685	0.924
4273	0.623	0.909
4274	0.651	0.926
4275	0.671	0.912
4276	0.620	0.920
4277	0.670	0.914
4278	0.666	0.913
4279	0.663	0.922
4280	0.695	0.923
4281	0.680	0.927
4282	0.671	0.923
4283	0.656	0.922
4284	0.659	0.923
4285	0.675	0.915
4286	0.696	0.923
4287	0.643	0.912
4288	0.700	0.922
4289	0.701	0.928
4290	0.687	0.908
4291	0.605	0.907
4292	0.670	0.924
4293	0.705	0.917
4294	0.693	0.921
4295	0.655	0.915
4296	0.664	0.926
4297	0.655	0.916
4298	0.683	0.916
4299	0.698	0.908

4300	0.689	0.918
4301	0.667	0.918
4302	0.689	0.932
4303	0.685	0.923
4304	0.693	0.918
4305	0.681	0.915
4306	0.669	0.907
4307	0.682	0.914
4308	0.640	0.928
4309	0.673	0.928
4310	0.693	0.913
4311	0.664	0.906
4312	0.624	0.929
4313	0.689	0.932
4314	0.733	0.922
4315	0.664	0.918
4316	0.667	0.915
4317	0.639	0.925
4318	0.701	0.928
4319	0.675	0.928
4320	0.672	0.930
4321	0.665	0.931
4322	0.654	0.919
4323	0.656	0.922
4324	0.696	0.925
4325	0.675	0.905
4326	0.642	0.915
4327	0.659	0.920
4328	0.662	0.918
4329	0.671	0.923
4330	0.728	0.924
4331	0.666	0.924
4332	0.680	0.918
4333	0.645	0.926
4334	0.689	0.902
4335	0.703	0.926
4336	0.663	0.930
4337	0.690	0.912
4338	0.678	0.924

4339	0.643	0.924
4340	0.647	0.897
4341	0.673	0.924
4342	0.661	0.927
4343	0.677	0.914
4344	0.635	0.922
4345	0.653	0.917
4346	0.685	0.926
4347	0.631	0.914
4348	0.659	0.920
4349	0.620	0.910
4350	0.702	0.927
4351	0.673	0.911
4352	0.621	0.918
4353	0.694	0.916
4354	0.667	0.914
4355	0.682	0.904
4356	0.711	0.920
4357	0.672	0.920
4358	0.686	0.912
4359	0.659	0.921
4360	0.717	0.916
4361	0.673	0.915
4362	0.679	0.913
4363	0.668	0.914
4364	0.654	0.926
4365	0.662	0.919
4366	0.693	0.930
4367	0.663	0.917
4368	0.669	0.915
4369	0.656	0.922
4370	0.708	0.913
4371	0.701	0.918
4372	0.636	0.921
4373	0.635	0.906
4374	0.682	0.925
4375	0.661	0.923
4376	0.679	0.902
4377	0.635	0.913

4378	0.646	0.925
4379	0.713	0.928
4380	0.701	0.925
4381	0.656	0.921
4382	0.687	0.914
4383	0.706	0.919
4384	0.695	0.931
4385	0.691	0.921
4386	0.669	0.922
4387	0.667	0.925
4388	0.633	0.927
4389	0.625	0.918
4390	0.641	0.912
4391	0.660	0.923
4392	0.682	0.936
4393	0.705	0.918
4394	0.695	0.915
4395	0.675	0.928
4396	0.637	0.919
4397	0.674	0.925
4398	0.643	0.923
4399	0.689	0.908
4400	0.676	0.909
4401	0.652	0.920
4402	0.675	0.919
4403	0.653	0.916
4404	0.643	0.922
4405	0.693	0.918
4406	0.655	0.925
4407	0.682	0.914
4408	0.682	0.920
4409	0.662	0.915
4410	0.689	0.918
4411	0.641	0.921
4412	0.678	0.919
4413	0.686	0.935
4414	0.678	0.924
4415	0.659	0.923
4416	0.660	0.909

4417	0.639	0.925
4418	0.641	0.913
4419	0.670	0.910
4420	0.633	0.925
4421	0.678	0.923
4422	0.653	0.907
4423	0.712	0.920
4424	0.626	0.915
4425	0.698	0.926
4426	0.677	0.915
4427	0.683	0.920
4428	0.644	0.917
4429	0.693	0.911
4430	0.709	0.908
4431	0.657	0.915
4432	0.721	0.918
4433	0.671	0.921
4434	0.662	0.925
4435	0.659	0.925
4436	0.664	0.924
4437	0.635	0.917
4438	0.667	0.924
4439	0.729	0.927
4440	0.693	0.921
4441	0.640	0.930
4442	0.669	0.914
4443	0.670	0.931
4444	0.714	0.926
4445	0.672	0.915
4446	0.702	0.918
4447	0.666	0.909
4448	0.664	0.926
4449	0.701	0.920
4450	0.652	0.909
4451	0.681	0.919
4452	0.636	0.933
4453	0.706	0.917
4454	0.664	0.930
4455	0.681	0.919

4456	0.656	0.920
4457	0.662	0.920
4458	0.716	0.922
4459	0.666	0.919
4460	0.671	0.923
4461	0.663	0.926
4462	0.648	0.908
4463	0.652	0.931
4464	0.628	0.921
4465	0.682	0.920
4466	0.715	0.918
4467	0.666	0.927
4468	0.693	0.930
4469	0.659	0.918
4470	0.690	0.926
4471	0.700	0.923
4472	0.666	0.918
4473	0.680	0.918
4474	0.673	0.918
4475	0.693	0.936
4476	0.661	0.922
4477	0.710	0.915
4478	0.663	0.915
4479	0.663	0.930
4480	0.659	0.931
4481	0.659	0.928
4482	0.612	0.919
4483	0.652	0.920
4484	0.661	0.922
4485	0.676	0.918
4486	0.678	0.915
4487	0.679	0.928
4488	0.689	0.922
4489	0.679	0.913
4490	0.682	0.924
4491	0.682	0.931
4492	0.687	0.929
4493	0.663	0.914
4494	0.671	0.915

4495	0.664	0.913
4496	0.681	0.908
4497	0.641	0.933
4498	0.648	0.912
4499	0.648	0.909
4500	0.693	0.919
4501	0.681	0.920
4502	0.639	0.930
4503	0.665	0.919
4504	0.690	0.915
4505	0.656	0.926
4506	0.673	0.920
4507	0.639	0.931
4508	0.635	0.922
4509	0.675	0.919
4510	0.688	0.923
4511	0.647	0.926
4512	0.685	0.904
4513	0.655	0.918
4514	0.666	0.924
4515	0.698	0.910
4516	0.622	0.918
4517	0.679	0.920
4518	0.687	0.917
4519	0.671	0.930
4520	0.667	0.917
4521	0.652	0.930
4522	0.648	0.919
4523	0.689	0.923
4524	0.681	0.922
4525	0.664	0.919
4526	0.662	0.918
4527	0.629	0.920
4528	0.667	0.914
4529	0.682	0.926
4530	0.649	0.917
4531	0.662	0.919
4532	0.662	0.911
4533	0.683	0.924

4534	0.705	0.908
4535	0.666	0.910
4536	0.683	0.912
4537	0.625	0.916
4538	0.685	0.915
4539	0.643	0.922
4540	0.675	0.908
4541	0.734	0.904
4542	0.681	0.913
4543	0.667	0.912
4544	0.651	0.910
4545	0.652	0.913
4546	0.668	0.927
4547	0.636	0.899
4548	0.639	0.919
4549	0.682	0.913
4550	0.694	0.908
4551	0.638	0.926
4552	0.625	0.930
4553	0.625	0.926
4554	0.647	0.918
4555	0.645	0.919
4556	0.695	0.913
4557	0.645	0.933
4558	0.683	0.915
4559	0.647	0.898
4560	0.658	0.932
4561	0.640	0.927
4562	0.646	0.924
4563	0.675	0.917
4564	0.684	0.923
4565	0.651	0.918
4566	0.656	0.924
4567	0.677	0.923
4568	0.670	0.916
4569	0.670	0.910
4570	0.644	0.936
4571	0.654	0.925
4572	0.703	0.927

4573	0.648	0.905
4574	0.673	0.909
4575	0.632	0.922
4576	0.658	0.908
4577	0.679	0.924
4578	0.645	0.916
4579	0.652	0.920
4580	0.628	0.912
4581	0.696	0.921
4582	0.679	0.927
4583	0.693	0.914
4584	0.679	0.913
4585	0.661	0.921
4586	0.675	0.928
4587	0.613	0.925
4588	0.612	0.915
4589	0.656	0.919
4590	0.657	0.917
4591	0.707	0.930
4592	0.677	0.933
4593	0.669	0.913
4594	0.661	0.903
4595	0.647	0.909
4596	0.616	0.924
4597	0.627	0.920
4598	0.647	0.922
4599	0.670	0.930
4600	0.651	0.928
4601	0.671	0.912
4602	0.637	0.925
4603	0.675	0.910
4604	0.681	0.916
4605	0.695	0.926
4606	0.635	0.909
4607	0.690	0.915
4608	0.641	0.911
4609	0.661	0.911
4610	0.646	0.927
4611	0.686	0.918

4612	0.673	0.906
4613	0.650	0.913
4614	0.674	0.925
4615	0.664	0.925
4616	0.692	0.919
4617	0.710	0.923
4618	0.680	0.895
4619	0.613	0.926
4620	0.662	0.912
4621	0.677	0.924
4622	0.668	0.919
4623	0.692	0.926
4624	0.652	0.916
4625	0.670	0.918
4626	0.704	0.914
4627	0.695	0.914
4628	0.649	0.911
4629	0.601	0.916
4630	0.685	0.922
4631	0.685	0.914
4632	0.674	0.925
4633	0.670	0.916
4634	0.635	0.920
4635	0.676	0.912
4636	0.646	0.903
4637	0.654	0.927
4638	0.674	0.928
4639	0.628	0.910
4640	0.633	0.915
4641	0.633	0.914
4642	0.660	0.924
4643	0.670	0.917
4644	0.675	0.904
4645	0.659	0.920
4646	0.647	0.914
4647	0.655	0.912
4648	0.640	0.900
4649	0.622	0.910
4650	0.660	0.924

4651	0.645	0.907
4652	0.643	0.915
4653	0.636	0.912
4654	0.681	0.909
4655	0.629	0.918
4656	0.674	0.917
4657	0.692	0.910
4658	0.669	0.918
4659	0.670	0.923
4660	0.667	0.918
4661	0.678	0.924
4662	0.689	0.918
4663	0.624	0.914
4664	0.682	0.924
4665	0.648	0.924
4666	0.632	0.907
4667	0.629	0.913
4668	0.632	0.924
4669	0.696	0.910
4670	0.674	0.920
4671	0.648	0.917
4672	0.644	0.915
4673	0.665	0.926
4674	0.613	0.926
4675	0.658	0.918
4676	0.587	0.929
4677	0.677	0.918
4678	0.626	0.921
4679	0.644	0.918
4680	0.665	0.910
4681	0.652	0.927
4682	0.678	0.919
4683	0.629	0.917
4684	0.670	0.916
4685	0.689	0.923
4686	0.697	0.929
4687	0.674	0.922
4688	0.655	0.915
4689	0.647	0.930

4690	0.701	0.922
4691	0.662	0.922
4692	0.663	0.905
4693	0.682	0.914
4694	0.685	0.915
4695	0.687	0.922
4696	0.617	0.914
4697	0.653	0.921
4698	0.650	0.909
4699	0.642	0.909
4700	0.695	0.920
4701	0.688	0.912
4702	0.668	0.937
4703	0.663	0.916
4704	0.679	0.923
4705	0.642	0.920
4706	0.684	0.912
4707	0.653	0.917
4708	0.662	0.913
4709	0.654	0.917
4710	0.661	0.916
4711	0.652	0.914
4712	0.674	0.922
4713	0.662	0.909
4714	0.708	0.919
4715	0.695	0.907
4716	0.657	0.914
4717	0.653	0.906
4718	0.674	0.916
4719	0.701	0.910
4720	0.678	0.922
4721	0.652	0.924
4722	0.656	0.922
4723	0.678	0.919
4724	0.665	0.924
4725	0.650	0.920
4726	0.659	0.912
4727	0.611	0.916
4728	0.654	0.920

4729	0.717	0.915
4730	0.635	0.924
4731	0.716	0.911
4732	0.705	0.923
4733	0.652	0.929
4734	0.673	0.933
4735	0.656	0.923
4736	0.623	0.918
4737	0.630	0.914
4738	0.671	0.907
4739	0.672	0.911
4740	0.643	0.920
4741	0.646	0.922
4742	0.677	0.914
4743	0.698	0.910
4744	0.688	0.910
4745	0.679	0.914
4746	0.637	0.916
4747	0.685	0.909
4748	0.695	0.919
4749	0.656	0.922
4750	0.635	0.913
4751	0.651	0.916
4752	0.653	0.918
4753	0.673	0.908
4754	0.650	0.918
4755	0.652	0.910
4756	0.687	0.904
4757	0.660	0.919
4758	0.666	0.918
4759	0.685	0.918
4760	0.670	0.930
4761	0.649	0.918
4762	0.654	0.915
4763	0.696	0.905
4764	0.655	0.918
4765	0.661	0.920
4766	0.677	0.900
4767	0.648	0.912

4768	0.655	0.907
4769	0.645	0.925
4770	0.698	0.930
4771	0.679	0.911
4772	0.678	0.916
4773	0.640	0.907
4774	0.656	0.912
4775	0.654	0.915
4776	0.662	0.919
4777	0.652	0.908
4778	0.654	0.921
4779	0.639	0.926
4780	0.663	0.918
4781	0.689	0.928
4782	0.628	0.907
4783	0.679	0.920
4784	0.644	0.918
4785	0.711	0.911
4786	0.664	0.923
4787	0.706	0.924
4788	0.671	0.920
4789	0.672	0.921
4790	0.665	0.916
4791	0.658	0.911
4792	0.696	0.923
4793	0.645	0.917
4794	0.686	0.918
4795	0.620	0.916
4796	0.659	0.922
4797	0.652	0.913
4798	0.718	0.909
4799	0.681	0.928
4800	0.673	0.909
4801	0.660	0.916
4802	0.686	0.908
4803	0.682	0.916
4804	0.673	0.919
4805	0.664	0.908
4806	0.668	0.902

4807	0.683	0.911
4808	0.655	0.922
4809	0.681	0.906
4810	0.647	0.916
4811	0.704	0.918
4812	0.677	0.915
4813	0.670	0.919
4814	0.620	0.917
4815	0.682	0.923
4816	0.614	0.920
4817	0.653	0.920
4818	0.669	0.918
4819	0.687	0.905
4820	0.639	0.922
4821	0.640	0.920
4822	0.643	0.904
4823	0.608	0.912
4824	0.721	0.927
4825	0.654	0.912
4826	0.661	0.916
4827	0.635	0.914
4828	0.649	0.922
4829	0.639	0.916
4830	0.655	0.921
4831	0.637	0.919
4832	0.653	0.913
4833	0.646	0.916
4834	0.715	0.923
4835	0.654	0.902
4836	0.685	0.922
4837	0.633	0.909
4838	0.642	0.919
4839	0.653	0.927
4840	0.631	0.917
4841	0.652	0.913
4842	0.672	0.916
4843	0.685	0.906
4844	0.670	0.918
4845	0.655	0.918

4846	0.656	0.911
4847	0.671	0.908
4848	0.636	0.902
4849	0.673	0.909
4850	0.659	0.919
4851	0.681	0.901
4852	0.655	0.905
4853	0.642	0.921
4854	0.653	0.913
4855	0.673	0.926
4856	0.603	0.908
4857	0.697	0.916
4858	0.664	0.913
4859	0.662	0.910
4860	0.663	0.912
4861	0.660	0.920
4862	0.657	0.921
4863	0.630	0.922
4864	0.701	0.909
4865	0.642	0.925
4866	0.675	0.917
4867	0.679	0.919
4868	0.642	0.928
4869	0.701	0.920
4870	0.662	0.912
4871	0.678	0.913
4872	0.635	0.909
4873	0.683	0.928
4874	0.663	0.920
4875	0.641	0.915
4876	0.644	0.914
4877	0.675	0.912
4878	0.648	0.916
4879	0.598	0.908
4880	0.675	0.913
4881	0.651	0.917
4882	0.661	0.922
4883	0.626	0.908
4884	0.640	0.916

4885	0.667	0.918
4886	0.665	0.927
4887	0.654	0.903
4888	0.714	0.911
4889	0.677	0.909
4890	0.615	0.923
4891	0.652	0.926
4892	0.666	0.917
4893	0.679	0.913
4894	0.696	0.917
4895	0.621	0.911
4896	0.620	0.914
4897	0.662	0.900
4898	0.657	0.909
4899	0.628	0.906
4900	0.666	0.911
4901	0.619	0.912
4902	0.664	0.915
4903	0.664	0.919
4904	0.632	0.925
4905	0.622	0.918
4906	0.660	0.919
4907	0.670	0.924
4908	0.673	0.909
4909	0.654	0.923
4910	0.633	0.923
4911	0.648	0.915
4912	0.652	0.922
4913	0.661	0.918
4914	0.686	0.928
4915	0.663	0.922
4916	0.666	0.908
4917	0.672	0.909
4918	0.708	0.904
4919	0.728	0.920
4920	0.673	0.920
4921	0.686	0.934
4922	0.687	0.922
4923	0.680	0.931

4924	0.662	0.914
4925	0.670	0.912
4926	0.666	0.915
4927	0.643	0.903
4928	0.732	0.919
4929	0.659	0.915
4930	0.667	0.902
4931	0.626	0.913
4932	0.689	0.917
4933	0.697	0.918
4934	0.655	0.914
4935	0.645	0.916
4936	0.702	0.922
4937	0.633	0.921
4938	0.672	0.908
4939	0.677	0.915
4940	0.700	0.903
4941	0.644	0.918
4942	0.702	0.917
4943	0.662	0.918
4944	0.641	0.907
4945	0.645	0.912
4946	0.689	0.926
4947	0.635	0.910
4948	0.629	0.923
4949	0.675	0.917
4950	0.671	0.915
4951	0.672	0.924
4952	0.674	0.916
4953	0.642	0.923
4954	0.681	0.927
4955	0.689	0.917
4956	0.646	0.911
4957	0.675	0.923
4958	0.678	0.911
4959	0.685	0.919
4960	0.673	0.921
4961	0.651	0.914
4962	0.644	0.922

4963	0.669	0.902
4964	0.602	0.918
4965	0.668	0.911
4966	0.662	0.917
4967	0.647	0.923
4968	0.653	0.923
4969	0.683	0.923
4970	0.670	0.909
4971	0.658	0.894
4972	0.651	0.916
4973	0.690	0.916
4974	0.652	0.929
4975	0.679	0.919
4976	0.670	0.914
4977	0.647	0.910
4978	0.668	0.910
4979	0.660	0.923
4980	0.682	0.922
4981	0.647	0.915
4982	0.673	0.913
4983	0.661	0.915
4984	0.664	0.918
4985	0.668	0.920
4986	0.710	0.900
4987	0.644	0.915
4988	0.658	0.915
4989	0.664	0.905
4990	0.620	0.913
4991	0.644	0.922
4992	0.664	0.927
4993	0.634	0.918
4994	0.645	0.916
4995	0.619	0.910
4996	0.668	0.909
4997	0.635	0.928
4998	0.639	0.930
4999	0.654	0.918
