# Supplementary material for: Identifying Patients with Atrioventricular Septal Defect in Down Syndrome Populations by Using Self-Normalizing Neural Networks and Feature Selection
Source: Genes (Basel). 2018 Apr 12;9(4):208. doi: 10.3390/genes9040208 (PMC5924550; doi:10.3390/genes9040208)
Supplement: Supplementary file 1 [file genes-09-00208-s001.zip › Suppl_S3.pdf]

Supplementary Material S3. MCCs and AUCs resulting from IFS method and RF classifiers.

**Number of features**	***MCC***	**AUC**
10	0.284	0.731
11	0.311	0.732
12	0.310	0.736
13	0.373	0.752
14	0.396	0.749
15	0.339	0.749
16	0.365	0.758
17	0.404	0.748
18	0.396	0.749
19	0.388	0.748
20	0.396	0.751
21	0.408	0.758
22	0.392	0.754
23	0.362	0.758
24	0.408	0.756
25	0.376	0.768
26	0.400	0.770
27	0.400	0.774
28	0.424	0.776
29	0.459	0.789
30	0.439	0.780
31	0.463	0.784
32	0.443	0.783
33	0.419	0.770
34	0.419	0.774
35	0.451	0.783
36	0.447	0.775
37	0.443	0.789
38	0.435	0.778
39	0.443	0.789
40	0.455	0.775
41	0.435	0.784
42	0.467	0.803
43	0.486	0.806
44	0.467	0.806
45	0.459	0.812
46	0.463	0.800
47	0.498	0.811
48	0.510	0.805

49	0.498	0.802
50	0.486	0.816
51	0.483	0.800
52	0.471	0.799
53	0.478	0.799
54	0.494	0.811
55	0.507	0.817
56	0.482	0.816
57	0.468	0.804
58	0.526	0.826
59	0.550	0.830
60	0.498	0.804
61	0.518	0.822
62	0.491	0.814
63	0.526	0.824
64	0.526	0.809
65	0.506	0.824
66	0.507	0.822
67	0.471	0.811
68	0.494	0.829
69	0.526	0.812
70	0.494	0.811
71	0.494	0.813
72	0.444	0.803
73	0.499	0.807
74	0.491	0.816
75	0.531	0.823
76	0.519	0.820
77	0.519	0.817
78	0.546	0.826
79	0.507	0.826
80	0.510	0.818
81	0.502	0.809
82	0.504	0.826
83	0.507	0.817
84	0.508	0.824
85	0.503	0.819
86	0.515	0.822
87	0.499	0.811
88	0.459	0.807
89	0.495	0.824
90	0.508	0.835

91	0.499	0.813
92	0.490	0.826
93	0.515	0.843
94	0.528	0.828
95	0.543	0.829
96	0.519	0.816
97	0.508	0.821
98	0.519	0.833
99	0.508	0.828
100	0.550	0.829
101	0.526	0.838
102	0.543	0.843
103	0.550	0.826
104	0.511	0.830
105	0.538	0.824
106	0.535	0.829
107	0.523	0.826
108	0.563	0.824
109	0.515	0.832
110	0.518	0.832
111	0.563	0.836
112	0.501	0.824
113	0.523	0.837
114	0.520	0.839
115	0.537	0.832
116	0.545	0.820
117	0.519	0.830
118	0.539	0.829
119	0.552	0.833
120	0.527	0.833
121	0.537	0.825
122	0.507	0.822
123	0.515	0.834
124	0.538	0.844
125	0.523	0.833
126	0.558	0.825
127	0.501	0.830
128	0.540	0.834
129	0.541	0.826
130	0.504	0.835
131	0.557	0.838
132	0.582	0.834

133	0.487	0.831
134	0.478	0.819
135	0.531	0.832
136	0.559	0.840
137	0.501	0.823
138	0.524	0.836
139	0.530	0.825
140	0.560	0.833
141	0.558	0.833
142	0.471	0.821
143	0.484	0.818
144	0.537	0.832
145	0.549	0.828
146	0.519	0.830
147	0.563	0.835
148	0.555	0.831
149	0.504	0.825
150	0.538	0.841
151	0.566	0.856
152	0.524	0.831
153	0.540	0.836
154	0.546	0.836
155	0.534	0.829
156	0.550	0.830
157	0.543	0.833
158	0.522	0.820
159	0.504	0.827
160	0.555	0.843
161	0.558	0.833
162	0.519	0.834
163	0.542	0.830
164	0.498	0.823
165	0.534	0.834
166	0.543	0.832
167	0.511	0.821
168	0.554	0.847
169	0.534	0.823
170	0.522	0.843
171	0.547	0.841
172	0.558	0.840
173	0.513	0.837
174	0.561	0.829

175	0.555	0.828
176	0.481	0.826
177	0.530	0.842
178	0.550	0.832
179	0.507	0.816
180	0.528	0.829
181	0.552	0.822
182	0.515	0.829
183	0.534	0.832
184	0.538	0.835
185	0.490	0.828
186	0.503	0.827
187	0.563	0.842
188	0.569	0.828
189	0.513	0.817
190	0.490	0.828
191	0.548	0.837
192	0.570	0.834
193	0.531	0.842
194	0.547	0.832
195	0.544	0.848
196	0.523	0.838
197	0.520	0.834
198	0.538	0.841
199	0.555	0.848
200	0.535	0.838
201	0.551	0.836
202	0.528	0.840
203	0.512	0.830
204	0.556	0.837
205	0.519	0.832
206	0.524	0.842
207	0.524	0.829
208	0.518	0.833
209	0.554	0.846
210	0.513	0.829
211	0.559	0.834
212	0.552	0.840
213	0.548	0.840
214	0.494	0.828
215	0.571	0.859
216	0.535	0.827

217	0.516	0.830
218	0.533	0.854
219	0.536	0.842
220	0.528	0.833
221	0.534	0.834
222	0.535	0.844
223	0.536	0.833
224	0.533	0.837
225	0.580	0.832
226	0.511	0.816
227	0.570	0.842
228	0.542	0.843
229	0.542	0.836
230	0.529	0.839
231	0.555	0.844
232	0.562	0.846
233	0.532	0.832
234	0.526	0.834
235	0.523	0.830
236	0.538	0.838
237	0.521	0.829
238	0.556	0.836
239	0.568	0.837
240	0.493	0.827
241	0.536	0.847
242	0.491	0.827
243	0.524	0.832
244	0.537	0.842
245	0.527	0.835
246	0.533	0.832
247	0.520	0.846
248	0.565	0.847
249	0.559	0.848
250	0.504	0.829
251	0.516	0.839
252	0.527	0.839
253	0.505	0.841
254	0.538	0.835
255	0.517	0.836
256	0.535	0.840
257	0.516	0.837
258	0.526	0.844

259	0.496	0.831
260	0.540	0.832
261	0.507	0.834
262	0.519	0.837
263	0.529	0.844
264	0.540	0.838
265	0.492	0.839
266	0.502	0.836
267	0.514	0.832
268	0.537	0.834
269	0.505	0.834
270	0.519	0.833
271	0.525	0.841
272	0.515	0.818
273	0.493	0.826
274	0.514	0.829
275	0.548	0.841
276	0.548	0.842
277	0.506	0.836
278	0.524	0.827
279	0.558	0.853
280	0.511	0.831
281	0.533	0.834
282	0.510	0.838
283	0.535	0.827
284	0.527	0.836
285	0.512	0.835
286	0.529	0.829
287	0.480	0.811
288	0.559	0.848
289	0.544	0.855
290	0.504	0.844
291	0.539	0.829
292	0.495	0.835
293	0.518	0.831
294	0.517	0.827
295	0.553	0.854
296	0.503	0.839
297	0.519	0.823
298	0.515	0.829
299	0.468	0.822
300	0.530	0.844

301	0.512	0.828
302	0.526	0.835
303	0.516	0.826
304	0.532	0.841
305	0.547	0.845
306	0.481	0.829
307	0.521	0.828
308	0.522	0.842
309	0.563	0.845
310	0.550	0.848
311	0.514	0.839
312	0.532	0.841
313	0.566	0.846
314	0.527	0.842
315	0.492	0.832
316	0.525	0.836
317	0.541	0.846
318	0.498	0.827
319	0.519	0.842
320	0.469	0.823
321	0.493	0.818
322	0.540	0.839
323	0.515	0.833
324	0.539	0.836
325	0.505	0.845
326	0.556	0.848
327	0.533	0.828
328	0.541	0.826
329	0.506	0.825
330	0.462	0.835
331	0.519	0.842
332	0.468	0.816
333	0.507	0.829
334	0.471	0.831
335	0.523	0.834
336	0.540	0.840
337	0.551	0.844
338	0.526	0.833
339	0.505	0.846
340	0.518	0.827
341	0.571	0.851
342	0.503	0.842

343	0.562	0.855
344	0.543	0.835
345	0.508	0.830
346	0.497	0.839
347	0.501	0.827
348	0.564	0.840
349	0.477	0.830
350	0.559	0.842
351	0.511	0.837
352	0.512	0.828
353	0.494	0.838
354	0.518	0.830
355	0.522	0.833
356	0.506	0.839
357	0.519	0.830
358	0.492	0.831
359	0.494	0.837
360	0.521	0.833
361	0.549	0.834
362	0.553	0.845
363	0.496	0.828
364	0.482	0.826
365	0.517	0.839
366	0.504	0.821
367	0.516	0.822
368	0.479	0.818
369	0.528	0.847
370	0.524	0.827
371	0.537	0.842
372	0.514	0.833
373	0.530	0.834
374	0.520	0.842
375	0.483	0.831
376	0.445	0.824
377	0.490	0.828
378	0.510	0.844
379	0.510	0.846
380	0.476	0.836
381	0.533	0.832
382	0.478	0.832
383	0.509	0.827
384	0.515	0.830

385	0.507	0.840
386	0.495	0.836
387	0.475	0.831
388	0.478	0.827
389	0.516	0.836
390	0.510	0.826
391	0.468	0.829
392	0.452	0.820
393	0.571	0.846
394	0.516	0.834
395	0.496	0.831
396	0.476	0.829
397	0.536	0.831
398	0.458	0.836
399	0.561	0.838
400	0.513	0.844
401	0.529	0.837
402	0.519	0.836
403	0.498	0.822
404	0.480	0.829
405	0.526	0.837
406	0.541	0.842
407	0.472	0.831
408	0.526	0.851
409	0.504	0.823
410	0.494	0.832
411	0.488	0.838
412	0.508	0.845
413	0.532	0.837
414	0.506	0.835
415	0.547	0.851
416	0.536	0.828
417	0.462	0.836
418	0.492	0.839
419	0.564	0.855
420	0.503	0.834
421	0.463	0.810
422	0.544	0.836
423	0.488	0.838
424	0.507	0.831
425	0.487	0.834
426	0.496	0.829

427	0.478	0.823
428	0.440	0.816
429	0.521	0.852
430	0.516	0.841
431	0.548	0.840
432	0.497	0.834
433	0.464	0.816
434	0.504	0.835
435	0.533	0.848
436	0.524	0.825
437	0.533	0.843
438	0.476	0.830
439	0.545	0.850
440	0.472	0.829
441	0.473	0.820
442	0.484	0.835
443	0.519	0.826
444	0.490	0.833
445	0.499	0.828
446	0.477	0.830
447	0.488	0.830
448	0.529	0.848
449	0.507	0.833
450	0.484	0.835
451	0.470	0.823
452	0.516	0.824
453	0.563	0.849
454	0.514	0.832
455	0.473	0.832
456	0.531	0.850
457	0.494	0.841
458	0.492	0.837
459	0.516	0.845
460	0.483	0.822
461	0.495	0.827
462	0.486	0.834
463	0.534	0.843
464	0.505	0.837
465	0.499	0.835
466	0.523	0.835
467	0.462	0.828
468	0.497	0.835

469	0.543	0.847
470	0.533	0.830
471	0.514	0.832
472	0.532	0.852
473	0.527	0.833
474	0.511	0.846
475	0.512	0.832
476	0.476	0.832
477	0.518	0.833
478	0.522	0.831
479	0.501	0.840
480	0.501	0.814
481	0.516	0.834
482	0.491	0.823
483	0.493	0.846
484	0.523	0.838
485	0.549	0.853
486	0.514	0.833
487	0.476	0.834
488	0.497	0.830
489	0.493	0.826
490	0.523	0.830
491	0.517	0.828
492	0.509	0.839
493	0.532	0.843
494	0.548	0.831
495	0.535	0.858
496	0.517	0.838
497	0.477	0.834
498	0.504	0.831
499	0.480	0.832
500	0.493	0.837
501	0.480	0.827
502	0.524	0.847
503	0.495	0.835
504	0.540	0.844
505	0.512	0.839
506	0.501	0.841
507	0.476	0.826
508	0.532	0.847
509	0.509	0.833
510	0.490	0.834

511	0.508	0.835
512	0.511	0.839
513	0.487	0.827
514	0.475	0.825
515	0.521	0.838
516	0.496	0.830
517	0.518	0.834
518	0.497	0.830
519	0.489	0.830
520	0.512	0.830
521	0.528	0.844
522	0.539	0.846
523	0.511	0.847
524	0.481	0.842
525	0.515	0.833
526	0.509	0.851
527	0.540	0.833
528	0.520	0.843
529	0.492	0.832
530	0.514	0.840
531	0.481	0.840
532	0.483	0.831
533	0.537	0.839
534	0.511	0.835
535	0.449	0.816
536	0.491	0.835
537	0.516	0.851
538	0.517	0.840
539	0.551	0.851
540	0.472	0.837
541	0.525	0.838
542	0.528	0.830
543	0.522	0.853
544	0.490	0.838
545	0.535	0.828
546	0.529	0.844
547	0.482	0.834
548	0.475	0.826
549	0.504	0.841
550	0.494	0.833
551	0.445	0.823
552	0.511	0.848

553	0.441	0.814
554	0.481	0.823
555	0.469	0.825
556	0.500	0.833
557	0.491	0.831
558	0.462	0.816
559	0.500	0.827
560	0.542	0.842
561	0.483	0.834
562	0.502	0.844
563	0.528	0.837
564	0.554	0.835
565	0.499	0.836
566	0.494	0.838
567	0.509	0.847
568	0.527	0.836
569	0.510	0.851
570	0.506	0.829
571	0.494	0.826
572	0.484	0.831
573	0.496	0.835
574	0.510	0.835
575	0.528	0.849
576	0.470	0.831
577	0.467	0.837
578	0.582	0.847
579	0.510	0.831
580	0.516	0.843
581	0.549	0.842
582	0.486	0.815
583	0.536	0.849
584	0.516	0.849
585	0.528	0.839
586	0.497	0.826
587	0.502	0.826
588	0.559	0.854
589	0.537	0.837
590	0.543	0.846
591	0.479	0.821
592	0.494	0.825
593	0.473	0.835
594	0.526	0.838

595	0.484	0.841
596	0.452	0.827
597	0.541	0.853
598	0.483	0.816
599	0.533	0.832
600	0.532	0.841
601	0.511	0.829
602	0.478	0.827
603	0.548	0.845
604	0.504	0.822
605	0.518	0.827
606	0.494	0.829
607	0.529	0.850
608	0.468	0.828
609	0.537	0.843
610	0.503	0.829
611	0.482	0.827
612	0.541	0.827
613	0.502	0.829
614	0.517	0.833
615	0.523	0.856
616	0.489	0.832
617	0.456	0.817
618	0.508	0.834
619	0.556	0.852
620	0.462	0.827
621	0.483	0.827
622	0.486	0.836
623	0.511	0.837
624	0.531	0.822
625	0.498	0.823
626	0.534	0.845
627	0.478	0.826
628	0.471	0.810
629	0.481	0.829
630	0.540	0.851
631	0.503	0.833
632	0.501	0.825
633	0.491	0.833
634	0.487	0.821
635	0.479	0.832
636	0.530	0.853

637	0.490	0.830
638	0.489	0.831
639	0.520	0.834
640	0.536	0.845
641	0.497	0.825
642	0.457	0.817
643	0.485	0.830
644	0.454	0.816
645	0.501	0.825
646	0.514	0.832
647	0.454	0.810
648	0.449	0.819
649	0.522	0.844
650	0.473	0.843
651	0.466	0.837
652	0.491	0.826
653	0.519	0.833
654	0.452	0.825
655	0.512	0.846
656	0.526	0.845
657	0.533	0.840
658	0.488	0.824
659	0.454	0.810
660	0.506	0.832
661	0.503	0.830
662	0.430	0.820
663	0.477	0.823
664	0.473	0.823
665	0.510	0.840
666	0.490	0.825
667	0.506	0.834
668	0.510	0.837
669	0.468	0.835
670	0.529	0.845
671	0.512	0.832
672	0.499	0.821
673	0.485	0.826
674	0.490	0.836
675	0.542	0.846
676	0.519	0.841
677	0.497	0.817
678	0.548	0.850

679	0.476	0.831
680	0.505	0.823
681	0.498	0.832
682	0.502	0.822
683	0.471	0.818
684	0.491	0.839
685	0.459	0.825
686	0.520	0.838
687	0.484	0.824
688	0.506	0.840
689	0.508	0.835
690	0.470	0.824
691	0.510	0.838
692	0.470	0.845
693	0.473	0.828
694	0.478	0.837
695	0.560	0.859
696	0.500	0.816
697	0.455	0.817
698	0.506	0.839
699	0.510	0.824
700	0.476	0.836
701	0.497	0.838
702	0.480	0.824
703	0.508	0.817
704	0.547	0.833
705	0.508	0.840
706	0.490	0.839
707	0.479	0.811
708	0.512	0.836
709	0.524	0.832
710	0.559	0.831
711	0.481	0.825
712	0.498	0.832
713	0.525	0.833
714	0.510	0.835
715	0.490	0.829
716	0.514	0.836
717	0.453	0.826
718	0.511	0.832
719	0.522	0.830
720	0.505	0.820

721	0.489	0.827
722	0.508	0.841
723	0.504	0.830
724	0.434	0.814
725	0.483	0.827
726	0.542	0.833
727	0.477	0.823
728	0.570	0.849
729	0.526	0.840
730	0.505	0.845
731	0.524	0.837
732	0.529	0.841
733	0.501	0.834
734	0.489	0.824
735	0.557	0.825
736	0.522	0.829
737	0.457	0.826
738	0.516	0.830
739	0.519	0.845
740	0.480	0.818
741	0.508	0.843
742	0.501	0.845
743	0.497	0.825
744	0.505	0.831
745	0.490	0.826
746	0.445	0.820
747	0.514	0.841
748	0.506	0.831
749	0.514	0.830
750	0.491	0.835
751	0.478	0.822
752	0.464	0.820
753	0.488	0.817
754	0.532	0.842
755	0.537	0.837
756	0.485	0.821
757	0.476	0.825
758	0.510	0.841
759	0.503	0.832
760	0.481	0.822
761	0.481	0.824
762	0.490	0.832

763	0.516	0.836
764	0.533	0.847
765	0.476	0.815
766	0.471	0.824
767	0.503	0.846
768	0.470	0.822
769	0.514	0.834
770	0.534	0.839
771	0.496	0.821
772	0.506	0.830
773	0.508	0.837
774	0.521	0.827
775	0.501	0.845
776	0.469	0.815
777	0.501	0.830
778	0.508	0.826
779	0.524	0.846
780	0.470	0.819
781	0.426	0.840
782	0.484	0.840
783	0.475	0.831
784	0.478	0.826
785	0.517	0.836
786	0.479	0.835
787	0.523	0.841
788	0.456	0.814
789	0.493	0.838
790	0.483	0.822
791	0.547	0.844
792	0.473	0.831
793	0.449	0.805
794	0.466	0.813
795	0.476	0.808
796	0.488	0.831
797	0.450	0.817
798	0.497	0.823
799	0.502	0.823
800	0.481	0.818
801	0.461	0.827
802	0.488	0.833
803	0.497	0.835
804	0.503	0.836

805	0.515	0.838
806	0.467	0.834
807	0.513	0.836
808	0.511	0.834
809	0.511	0.829
810	0.471	0.823
811	0.496	0.847
812	0.477	0.826
813	0.495	0.829
814	0.484	0.833
815	0.527	0.818
816	0.523	0.830
817	0.521	0.826
818	0.471	0.826
819	0.498	0.838
820	0.490	0.821
821	0.475	0.816
822	0.459	0.816
823	0.462	0.812
824	0.463	0.829
825	0.526	0.845
826	0.457	0.831
827	0.543	0.848
828	0.469	0.817
829	0.469	0.830
830	0.419	0.833
831	0.535	0.845
832	0.506	0.819
833	0.533	0.833
834	0.526	0.833
835	0.498	0.821
836	0.429	0.817
837	0.494	0.836
838	0.481	0.827
839	0.521	0.825
840	0.481	0.828
841	0.465	0.821
842	0.480	0.824
843	0.474	0.834
844	0.427	0.812
845	0.452	0.831
846	0.510	0.844

847	0.470	0.838
848	0.507	0.846
849	0.522	0.831
850	0.479	0.824
851	0.433	0.821
852	0.491	0.832
853	0.477	0.819
854	0.483	0.828
855	0.493	0.812
856	0.529	0.820
857	0.496	0.809
858	0.483	0.840
859	0.474	0.819
860	0.476	0.829
861	0.514	0.822
862	0.486	0.821
863	0.496	0.818
864	0.438	0.819
865	0.485	0.837
866	0.521	0.833
867	0.461	0.825
868	0.511	0.834
869	0.492	0.815
870	0.441	0.816
871	0.524	0.831
872	0.482	0.809
873	0.462	0.796
874	0.464	0.811
875	0.491	0.831
876	0.506	0.831
877	0.486	0.822
878	0.501	0.844
879	0.469	0.826
880	0.491	0.818
881	0.503	0.830
882	0.494	0.848
883	0.445	0.816
884	0.519	0.833
885	0.478	0.835
886	0.532	0.838
887	0.506	0.828
888	0.541	0.853

889	0.510	0.840
890	0.479	0.821
891	0.480	0.820
892	0.493	0.829
893	0.473	0.841
894	0.447	0.806
895	0.531	0.846
896	0.488	0.828
897	0.473	0.827
898	0.493	0.811
899	0.511	0.824
900	0.478	0.824
901	0.514	0.839
902	0.449	0.804
903	0.465	0.815
904	0.459	0.822
905	0.503	0.815
906	0.525	0.833
907	0.511	0.829
908	0.508	0.822
909	0.526	0.822
910	0.499	0.825
911	0.493	0.825
912	0.481	0.838
913	0.488	0.831
914	0.510	0.842
915	0.519	0.830
916	0.500	0.839
917	0.466	0.831
918	0.476	0.822
919	0.482	0.821
920	0.433	0.826
921	0.512	0.837
922	0.491	0.824
923	0.464	0.828
924	0.447	0.815
925	0.481	0.820
926	0.472	0.821
927	0.507	0.835
928	0.482	0.829
929	0.496	0.817
930	0.485	0.826

931	0.505	0.829
932	0.479	0.819
933	0.494	0.837
934	0.478	0.816
935	0.506	0.846
936	0.471	0.833
937	0.506	0.842
938	0.490	0.823
939	0.505	0.839
940	0.502	0.824
941	0.484	0.814
942	0.499	0.832
943	0.510	0.845
944	0.486	0.822
945	0.509	0.824
946	0.474	0.829
947	0.483	0.823
948	0.513	0.822
949	0.471	0.823
950	0.509	0.824
951	0.430	0.820
952	0.482	0.825
953	0.520	0.831
954	0.485	0.831
955	0.531	0.847
956	0.494	0.838
957	0.499	0.826
958	0.464	0.835
959	0.496	0.832
960	0.464	0.808
961	0.502	0.819
962	0.449	0.820
963	0.459	0.812
964	0.517	0.830
965	0.492	0.835
966	0.521	0.837
967	0.533	0.841
968	0.488	0.816
969	0.492	0.827
970	0.522	0.851
971	0.457	0.822
972	0.461	0.816

973	0.502	0.831
974	0.484	0.828
975	0.470	0.833
976	0.483	0.826
977	0.470	0.820
978	0.526	0.832
979	0.465	0.825
980	0.448	0.819
981	0.490	0.825
982	0.472	0.814
983	0.472	0.834
984	0.481	0.820
985	0.491	0.813
986	0.441	0.817
987	0.489	0.828
988	0.496	0.836
989	0.513	0.838
990	0.452	0.823
991	0.509	0.823
992	0.480	0.827
993	0.493	0.830
994	0.492	0.815
995	0.466	0.815
996	0.509	0.825
997	0.492	0.807
998	0.484	0.826
999	0.449	0.812
1000	0.443	0.812
1001	0.447	0.820
1002	0.461	0.817
1003	0.500	0.834
1004	0.511	0.829
1005	0.482	0.813
1006	0.489	0.820
1007	0.486	0.820
1008	0.468	0.815
1009	0.518	0.815
1010	0.451	0.823
1011	0.498	0.826
1012	0.489	0.817
1013	0.514	0.828
1014	0.473	0.819

1015	0.461	0.824
1016	0.471	0.820
1017	0.462	0.824
1018	0.506	0.825
1019	0.517	0.834
1020	0.493	0.833
1021	0.480	0.811
1022	0.522	0.831
1023	0.414	0.816
1024	0.498	0.823
1025	0.478	0.810
1026	0.476	0.833
1027	0.526	0.826
1028	0.520	0.830
1029	0.462	0.817
1030	0.449	0.820
1031	0.509	0.829
1032	0.481	0.824
1033	0.513	0.831
1034	0.484	0.824
1035	0.514	0.825
1036	0.490	0.811
1037	0.522	0.841
1038	0.528	0.832
1039	0.484	0.817
1040	0.470	0.818
1041	0.500	0.825
1042	0.462	0.824
1043	0.465	0.821
1044	0.477	0.800
1045	0.475	0.833
1046	0.508	0.838
1047	0.488	0.820
1048	0.497	0.833
1049	0.459	0.825
1050	0.454	0.813
1051	0.528	0.844
1052	0.498	0.818
1053	0.507	0.826
1054	0.504	0.816
1055	0.488	0.814
1056	0.464	0.809

1057	0.495	0.832
1058	0.495	0.817
1059	0.539	0.832
1060	0.450	0.814
1061	0.526	0.819
1062	0.498	0.833
1063	0.489	0.829
1064	0.529	0.826
1065	0.460	0.828
1066	0.498	0.836
1067	0.453	0.799
1068	0.473	0.815
1069	0.495	0.835
1070	0.518	0.833
1071	0.472	0.812
1072	0.516	0.831
1073	0.477	0.827
1074	0.469	0.813
1075	0.478	0.836
1076	0.425	0.805
1077	0.457	0.812
1078	0.486	0.830
1079	0.446	0.811
1080	0.499	0.835
1081	0.487	0.812
1082	0.496	0.818
1083	0.490	0.833
1084	0.457	0.815
1085	0.457	0.831
1086	0.485	0.825
1087	0.496	0.820
1088	0.470	0.827
1089	0.505	0.824
1090	0.506	0.829
1091	0.462	0.821
1092	0.449	0.805
1093	0.505	0.833
1094	0.510	0.825
1095	0.504	0.810
1096	0.498	0.828
1097	0.498	0.838
1098	0.466	0.820

1099	0.544	0.849
1100	0.459	0.830
1101	0.528	0.834
1102	0.494	0.812
1103	0.509	0.825
1104	0.516	0.831
1105	0.483	0.825
1106	0.512	0.825
1107	0.449	0.812
1108	0.503	0.824
1109	0.471	0.825
1110	0.520	0.820
1111	0.518	0.814
1112	0.500	0.834
1113	0.531	0.838
1114	0.486	0.834
1115	0.479	0.822
1116	0.474	0.806
1117	0.480	0.814
1118	0.484	0.811
1119	0.422	0.802
1120	0.469	0.815
1121	0.450	0.815
1122	0.520	0.843
1123	0.468	0.820
1124	0.495	0.831
1125	0.454	0.809
1126	0.502	0.834
1127	0.438	0.794
1128	0.474	0.818
1129	0.484	0.818
1130	0.514	0.822
1131	0.491	0.823
1132	0.484	0.827
1133	0.422	0.809
1134	0.480	0.824
1135	0.466	0.818
1136	0.466	0.805
1137	0.491	0.798
1138	0.491	0.818
1139	0.469	0.820
1140	0.466	0.813

1141	0.509	0.824
1142	0.491	0.829
1143	0.488	0.827
1144	0.557	0.843
1145	0.531	0.840
1146	0.425	0.810
1147	0.484	0.831
1148	0.485	0.813
1149	0.511	0.812
1150	0.501	0.825
1151	0.477	0.827
1152	0.416	0.815
1153	0.530	0.846
1154	0.517	0.823
1155	0.501	0.815
1156	0.508	0.817
1157	0.484	0.821
1158	0.462	0.827
1159	0.511	0.831
1160	0.473	0.822
1161	0.488	0.823
1162	0.488	0.823
1163	0.458	0.816
1164	0.508	0.830
1165	0.484	0.834
1166	0.482	0.810
1167	0.433	0.820
1168	0.462	0.821
1169	0.443	0.809
1170	0.462	0.815
1171	0.490	0.816
1172	0.442	0.804
1173	0.484	0.822
1174	0.462	0.832
1175	0.488	0.824
1176	0.501	0.818
1177	0.493	0.812
1178	0.504	0.821
1179	0.485	0.812
1180	0.498	0.819
1181	0.491	0.817
1182	0.416	0.811

1183	0.486	0.828
1184	0.506	0.812
1185	0.476	0.817
1186	0.517	0.829
1187	0.489	0.832
1188	0.483	0.802
1189	0.457	0.830
1190	0.482	0.827
1191	0.466	0.803
1192	0.431	0.808
1193	0.560	0.846
1194	0.485	0.824
1195	0.485	0.820
1196	0.491	0.834
1197	0.459	0.813
1198	0.465	0.818
1199	0.532	0.833
1200	0.487	0.817
1201	0.479	0.827
1202	0.405	0.809
1203	0.466	0.817
1204	0.513	0.842
1205	0.449	0.813
1206	0.436	0.799
1207	0.508	0.830
1208	0.473	0.826
1209	0.466	0.810
1210	0.481	0.822
1211	0.493	0.823
1212	0.482	0.814
1213	0.493	0.806
1214	0.468	0.809
1215	0.516	0.828
1216	0.462	0.811
1217	0.496	0.819
1218	0.474	0.823
1219	0.443	0.820
1220	0.479	0.812
1221	0.495	0.829
1222	0.507	0.829
1223	0.510	0.818
1224	0.493	0.839

1225	0.470	0.805
1226	0.496	0.810
1227	0.499	0.820
1228	0.477	0.829
1229	0.448	0.817
1230	0.443	0.808
1231	0.472	0.810
1232	0.446	0.810
1233	0.479	0.828
1234	0.486	0.835
1235	0.485	0.817
1236	0.493	0.818
1237	0.507	0.822
1238	0.451	0.816
1239	0.511	0.834
1240	0.442	0.816
1241	0.446	0.812
1242	0.510	0.829
1243	0.505	0.817
1244	0.454	0.821
1245	0.454	0.812
1246	0.490	0.810
1247	0.464	0.809
1248	0.458	0.820
1249	0.491	0.818
1250	0.511	0.830
1251	0.474	0.818
1252	0.462	0.813
1253	0.469	0.802
1254	0.458	0.810
1255	0.522	0.837
1256	0.443	0.798
1257	0.522	0.835
1258	0.495	0.829
1259	0.441	0.820
1260	0.470	0.819
1261	0.490	0.811
1262	0.413	0.808
1263	0.476	0.823
1264	0.496	0.822
1265	0.474	0.838
1266	0.506	0.828

1267	0.501	0.824
1268	0.486	0.815
1269	0.514	0.820
1270	0.500	0.822
1271	0.484	0.814
1272	0.455	0.823
1273	0.462	0.823
1274	0.528	0.831
1275	0.496	0.828
1276	0.484	0.824
1277	0.467	0.814
1278	0.444	0.804
1279	0.437	0.820
1280	0.492	0.815
1281	0.527	0.834
1282	0.470	0.819
1283	0.459	0.810
1284	0.486	0.821
1285	0.505	0.825
1286	0.481	0.816
1287	0.512	0.812
1288	0.464	0.803
1289	0.442	0.801
1290	0.490	0.804
1291	0.495	0.827
1292	0.466	0.813
1293	0.458	0.807
1294	0.473	0.827
1295	0.458	0.806
1296	0.487	0.809
1297	0.505	0.814
1298	0.501	0.825
1299	0.446	0.809
1300	0.474	0.819
1301	0.472	0.814
1302	0.379	0.802
1303	0.503	0.822
1304	0.473	0.823
1305	0.493	0.833
1306	0.446	0.798
1307	0.446	0.816
1308	0.419	0.809

1309	0.462	0.806
1310	0.440	0.809
1311	0.485	0.837
1312	0.439	0.805
1313	0.483	0.814
1314	0.509	0.805
1315	0.501	0.840
1316	0.408	0.790
1317	0.509	0.823
1318	0.430	0.802
1319	0.454	0.807
1320	0.443	0.811
1321	0.463	0.802
1322	0.430	0.806
1323	0.437	0.799
1324	0.452	0.811
1325	0.474	0.825
1326	0.498	0.825
1327	0.503	0.827
1328	0.484	0.832
1329	0.445	0.805
1330	0.485	0.823
1331	0.450	0.809
1332	0.489	0.815
1333	0.487	0.825
1334	0.476	0.821
1335	0.446	0.813
1336	0.460	0.826
1337	0.454	0.809
1338	0.469	0.829
1339	0.472	0.820
1340	0.445	0.799
1341	0.534	0.822
1342	0.468	0.814
1343	0.448	0.802
1344	0.489	0.805
1345	0.430	0.796
1346	0.520	0.842
1347	0.459	0.799
1348	0.437	0.799
1349	0.497	0.818
1350	0.469	0.815

1351	0.456	0.806
1352	0.418	0.820
1353	0.442	0.806
1354	0.479	0.818
1355	0.531	0.824
1356	0.516	0.827
1357	0.463	0.822
1358	0.440	0.817
1359	0.456	0.801
1360	0.473	0.818
1361	0.551	0.834
1362	0.430	0.804
1363	0.443	0.819
1364	0.488	0.827
1365	0.503	0.817
1366	0.450	0.794
1367	0.457	0.796
1368	0.472	0.809
1369	0.478	0.813
1370	0.469	0.804
1371	0.467	0.815
1372	0.457	0.794
1373	0.460	0.817
1374	0.484	0.820
1375	0.517	0.822
1376	0.454	0.802
1377	0.476	0.817
1378	0.458	0.814
1379	0.455	0.816
1380	0.448	0.810
1381	0.438	0.807
1382	0.481	0.808
1383	0.494	0.812
1384	0.480	0.814
1385	0.491	0.809
1386	0.444	0.799
1387	0.483	0.808
1388	0.496	0.831
1389	0.486	0.815
1390	0.473	0.815
1391	0.461	0.820
1392	0.479	0.821

1393	0.514	0.828
1394	0.501	0.827
1395	0.503	0.815
1396	0.428	0.797
1397	0.478	0.817
1398	0.494	0.817
1399	0.451	0.826
1400	0.491	0.820
1401	0.502	0.810
1402	0.437	0.796
1403	0.494	0.821
1404	0.477	0.810
1405	0.476	0.814
1406	0.450	0.812
1407	0.419	0.793
1408	0.473	0.819
1409	0.449	0.807
1410	0.458	0.819
1411	0.457	0.799
1412	0.464	0.822
1413	0.473	0.820
1414	0.453	0.804
1415	0.496	0.814
1416	0.496	0.802
1417	0.451	0.820
1418	0.454	0.808
1419	0.482	0.816
1420	0.419	0.799
1421	0.427	0.809
1422	0.449	0.816
1423	0.454	0.811
1424	0.465	0.815
1425	0.493	0.824
1426	0.501	0.807
1427	0.486	0.817
1428	0.490	0.820
1429	0.442	0.813
1430	0.431	0.814
1431	0.489	0.822
1432	0.448	0.811
1433	0.488	0.819
1434	0.476	0.797

1435	0.516	0.822
1436	0.497	0.819
1437	0.496	0.827
1438	0.460	0.822
1439	0.442	0.808
1440	0.491	0.818
1441	0.455	0.808
1442	0.505	0.823
1443	0.466	0.807
1444	0.447	0.802
1445	0.476	0.804
1446	0.471	0.803
1447	0.459	0.800
1448	0.448	0.811
1449	0.438	0.814
1450	0.485	0.821
1451	0.439	0.807
1452	0.464	0.799
1453	0.463	0.817
1454	0.464	0.800
1455	0.455	0.807
1456	0.497	0.828
1457	0.464	0.808
1458	0.457	0.823
1459	0.426	0.809
1460	0.473	0.814
1461	0.459	0.825
1462	0.465	0.822
1463	0.523	0.822
1464	0.438	0.816
1465	0.436	0.797
1466	0.491	0.814
1467	0.467	0.801
1468	0.463	0.826
1469	0.476	0.817
1470	0.471	0.808
1471	0.473	0.823
1472	0.430	0.800
1473	0.457	0.821
1474	0.467	0.814
1475	0.478	0.827
1476	0.438	0.788

1477	0.421	0.797
1478	0.426	0.822
1479	0.451	0.797
1480	0.444	0.803
1481	0.482	0.809
1482	0.494	0.811
1483	0.504	0.818
1484	0.472	0.814
1485	0.480	0.814
1486	0.507	0.823
1487	0.454	0.807
1488	0.475	0.794
1489	0.491	0.826
1490	0.456	0.802
1491	0.476	0.806
1492	0.472	0.800
1493	0.451	0.805
1494	0.496	0.820
1495	0.485	0.820
1496	0.486	0.813
1497	0.506	0.823
1498	0.484	0.804
1499	0.472	0.825
1500	0.483	0.813
1501	0.467	0.820
1502	0.421	0.806
1503	0.484	0.826
1504	0.479	0.812
1505	0.454	0.805
1506	0.476	0.817
1507	0.469	0.817
1508	0.478	0.806
1509	0.446	0.800
1510	0.457	0.799
1511	0.465	0.809
1512	0.479	0.809
1513	0.472	0.816
1514	0.452	0.802
1515	0.409	0.798
1516	0.439	0.799
1517	0.428	0.807
1518	0.454	0.796

1519	0.496	0.824
1520	0.508	0.818
1521	0.438	0.822
1522	0.445	0.790
1523	0.435	0.797
1524	0.508	0.818
1525	0.434	0.810
1526	0.492	0.813
1527	0.467	0.815
1528	0.468	0.808
1529	0.467	0.813
1530	0.414	0.815
1531	0.454	0.804
1532	0.455	0.805
1533	0.462	0.799
1534	0.429	0.795
1535	0.467	0.802
1536	0.451	0.802
1537	0.393	0.816
1538	0.454	0.804
1539	0.464	0.797
1540	0.440	0.805
1541	0.478	0.812
1542	0.506	0.831
1543	0.516	0.824
1544	0.532	0.844
1545	0.457	0.799
1546	0.502	0.816
1547	0.468	0.821
1548	0.419	0.798
1549	0.457	0.805
1550	0.499	0.822
1551	0.433	0.806
1552	0.450	0.794
1553	0.444	0.806
1554	0.459	0.819
1555	0.470	0.821
1556	0.481	0.821
1557	0.516	0.822
1558	0.491	0.825
1559	0.461	0.810
1560	0.501	0.810

1561	0.508	0.826
1562	0.485	0.825
1563	0.496	0.826
1564	0.427	0.819
1565	0.461	0.814
1566	0.442	0.806
1567	0.526	0.841
1568	0.472	0.812
1569	0.467	0.825
1570	0.474	0.821
1571	0.427	0.804
1572	0.487	0.801
1573	0.485	0.828
1574	0.484	0.803
1575	0.495	0.816
1576	0.446	0.804
1577	0.481	0.814
1578	0.418	0.813
1579	0.423	0.797
1580	0.455	0.810
1581	0.462	0.793
1582	0.431	0.802
1583	0.458	0.798
1584	0.502	0.824
1585	0.444	0.807
1586	0.455	0.805
1587	0.445	0.800
1588	0.386	0.795
1589	0.427	0.800
1590	0.532	0.830
1591	0.462	0.799
1592	0.486	0.819
1593	0.487	0.835
1594	0.459	0.815
1595	0.457	0.798
1596	0.466	0.812
1597	0.467	0.824
1598	0.499	0.819
1599	0.467	0.821
1600	0.395	0.811
1601	0.482	0.820
1602	0.441	0.798

1603	0.471	0.813
1604	0.467	0.815
1605	0.526	0.812
1606	0.486	0.818
1607	0.433	0.806
1608	0.465	0.808
1609	0.436	0.796
1610	0.519	0.813
1611	0.443	0.797
1612	0.447	0.799
1613	0.473	0.815
1614	0.443	0.829
1615	0.440	0.797
1616	0.481	0.829
1617	0.434	0.802
1618	0.480	0.815
1619	0.450	0.791
1620	0.413	0.802
1621	0.488	0.815
1622	0.447	0.797
1623	0.443	0.811
1624	0.429	0.810
1625	0.434	0.818
1626	0.465	0.814
1627	0.460	0.805
1628	0.494	0.836
1629	0.497	0.820
1630	0.471	0.809
1631	0.501	0.827
1632	0.470	0.804
1633	0.464	0.813
1634	0.408	0.812
1635	0.500	0.817
1636	0.465	0.821
1637	0.445	0.796
1638	0.473	0.815
1639	0.455	0.818
1640	0.457	0.807
1641	0.452	0.797
1642	0.450	0.825
1643	0.476	0.810
1644	0.481	0.794

1645	0.493	0.809
1646	0.433	0.813
1647	0.453	0.816
1648	0.490	0.817
1649	0.469	0.813
1650	0.458	0.828
1651	0.493	0.823
1652	0.448	0.793
1653	0.428	0.796
1654	0.424	0.800
1655	0.450	0.817
1656	0.447	0.816
1657	0.498	0.816
1658	0.476	0.804
1659	0.447	0.803
1660	0.425	0.795
1661	0.479	0.821
1662	0.451	0.801
1663	0.459	0.822
1664	0.434	0.796
1665	0.479	0.811
1666	0.440	0.791
1667	0.482	0.798
1668	0.485	0.807
1669	0.481	0.821
1670	0.447	0.807
1671	0.457	0.825
1672	0.433	0.805
1673	0.465	0.814
1674	0.473	0.819
1675	0.430	0.803
1676	0.448	0.790
1677	0.474	0.831
1678	0.503	0.817
1679	0.501	0.820
1680	0.441	0.807
1681	0.458	0.817
1682	0.465	0.807
1683	0.485	0.820
1684	0.453	0.810
1685	0.479	0.811
1686	0.455	0.813

1687	0.486	0.812
1688	0.462	0.799
1689	0.482	0.805
1690	0.435	0.805
1691	0.413	0.793
1692	0.466	0.823
1693	0.436	0.799
1694	0.471	0.808
1695	0.477	0.809
1696	0.497	0.824
1697	0.462	0.835
1698	0.506	0.823
1699	0.456	0.808
1700	0.450	0.809
1701	0.471	0.802
1702	0.457	0.794
1703	0.445	0.795
1704	0.439	0.817
1705	0.481	0.819
1706	0.457	0.821
1707	0.421	0.800
1708	0.479	0.805
1709	0.518	0.817
1710	0.484	0.801
1711	0.457	0.803
1712	0.448	0.820
1713	0.483	0.823
1714	0.475	0.813
1715	0.469	0.813
1716	0.472	0.816
1717	0.455	0.815
1718	0.466	0.795
1719	0.440	0.794
1720	0.445	0.797
1721	0.457	0.809
1722	0.401	0.794
1723	0.479	0.803
1724	0.452	0.797
1725	0.428	0.806
1726	0.451	0.801
1727	0.473	0.825
1728	0.423	0.795

1729	0.466	0.823
1730	0.444	0.808
1731	0.457	0.794
1732	0.443	0.814
1733	0.448	0.813
1734	0.468	0.820
1735	0.465	0.814
1736	0.466	0.811
1737	0.460	0.812
1738	0.471	0.799
1739	0.454	0.804
1740	0.431	0.798
1741	0.414	0.801
1742	0.464	0.804
1743	0.463	0.796
1744	0.449	0.812
1745	0.407	0.793
1746	0.482	0.790
1747	0.441	0.814
1748	0.454	0.800
1749	0.461	0.809
1750	0.462	0.803
1751	0.477	0.814
1752	0.510	0.820
1753	0.508	0.822
1754	0.477	0.807
1755	0.466	0.819
1756	0.464	0.823
1757	0.467	0.810
1758	0.461	0.811
1759	0.479	0.803
1760	0.486	0.827
1761	0.462	0.813
1762	0.493	0.823
1763	0.441	0.805
1764	0.435	0.814
1765	0.449	0.813
1766	0.434	0.796
1767	0.455	0.819
1768	0.446	0.815
1769	0.442	0.808
1770	0.451	0.803

1771	0.445	0.803
1772	0.485	0.815
1773	0.440	0.808
1774	0.467	0.825
1775	0.478	0.799
1776	0.417	0.805
1777	0.475	0.825
1778	0.466	0.819
1779	0.469	0.823
1780	0.406	0.801
1781	0.441	0.797
1782	0.445	0.793
1783	0.481	0.808
1784	0.412	0.795
1785	0.451	0.805
1786	0.487	0.820
1787	0.434	0.791
1788	0.473	0.814
1789	0.462	0.792
1790	0.468	0.821
1791	0.510	0.813
1792	0.402	0.789
1793	0.446	0.810
1794	0.460	0.803
1795	0.454	0.800
1796	0.446	0.790
1797	0.479	0.812
1798	0.479	0.804
1799	0.478	0.814
1800	0.447	0.788
1801	0.433	0.795
1802	0.412	0.804
1803	0.482	0.803
1804	0.513	0.814
1805	0.472	0.799
1806	0.482	0.801
1807	0.432	0.810
1808	0.431	0.790
1809	0.463	0.803
1810	0.475	0.800
1811	0.516	0.816
1812	0.458	0.807

1813	0.408	0.792
1814	0.519	0.806
1815	0.490	0.812
1816	0.457	0.803
1817	0.425	0.798
1818	0.461	0.809
1819	0.504	0.808
1820	0.453	0.798
1821	0.468	0.802
1822	0.453	0.794
1823	0.476	0.796
1824	0.445	0.800
1825	0.462	0.809
1826	0.451	0.789
1827	0.445	0.808
1828	0.494	0.807
1829	0.485	0.827
1830	0.494	0.803
1831	0.453	0.817
1832	0.476	0.822
1833	0.440	0.795
1834	0.522	0.833
1835	0.437	0.799
1836	0.434	0.796
1837	0.450	0.804
1838	0.463	0.796
1839	0.496	0.807
1840	0.462	0.806
1841	0.389	0.771
1842	0.423	0.801
1843	0.407	0.807
1844	0.449	0.811
1845	0.474	0.796
1846	0.467	0.811
1847	0.406	0.809
1848	0.474	0.816
1849	0.504	0.809
1850	0.438	0.799
1851	0.442	0.808
1852	0.436	0.799
1853	0.466	0.796
1854	0.468	0.792

1855	0.401	0.787
1856	0.461	0.804
1857	0.416	0.801
1858	0.459	0.778
1859	0.486	0.813
1860	0.414	0.784
1861	0.476	0.817
1862	0.466	0.808
1863	0.486	0.808
1864	0.462	0.787
1865	0.467	0.796
1866	0.464	0.799
1867	0.481	0.813
1868	0.450	0.787
1869	0.428	0.798
1870	0.515	0.818
1871	0.447	0.810
1872	0.472	0.802
1873	0.478	0.813
1874	0.451	0.804
1875	0.488	0.806
1876	0.418	0.795
1877	0.479	0.803
1878	0.503	0.813
1879	0.421	0.790
1880	0.494	0.813
1881	0.393	0.794
1882	0.409	0.778
1883	0.464	0.784
1884	0.453	0.805
1885	0.441	0.809
1886	0.406	0.800
1887	0.447	0.804
1888	0.440	0.789
1889	0.403	0.793
1890	0.483	0.806
1891	0.487	0.804
1892	0.484	0.816
1893	0.438	0.803
1894	0.375	0.781
1895	0.457	0.805
1896	0.467	0.817

1897	0.442	0.813
1898	0.418	0.794
1899	0.406	0.799
1900	0.418	0.788
1901	0.430	0.800
1902	0.442	0.788
1903	0.392	0.778
1904	0.483	0.814
1905	0.476	0.806
1906	0.474	0.827
1907	0.461	0.826
1908	0.474	0.788
1909	0.455	0.800
1910	0.442	0.799
1911	0.417	0.810
1912	0.429	0.790
1913	0.495	0.807
1914	0.484	0.804
1915	0.442	0.796
1916	0.460	0.806
1917	0.436	0.800
1918	0.453	0.798
1919	0.454	0.805
1920	0.417	0.786
1921	0.367	0.769
1922	0.468	0.810
1923	0.478	0.819
1924	0.435	0.789
1925	0.425	0.795
1926	0.405	0.787
1927	0.444	0.803
1928	0.446	0.815
1929	0.416	0.800
1930	0.431	0.810
1931	0.496	0.818
1932	0.417	0.792
1933	0.456	0.803
1934	0.511	0.812
1935	0.457	0.816
1936	0.392	0.803
1937	0.478	0.819
1938	0.440	0.796

1939	0.431	0.802
1940	0.435	0.787
1941	0.458	0.791
1942	0.458	0.812
1943	0.472	0.780
1944	0.478	0.819
1945	0.452	0.818
1946	0.463	0.811
1947	0.448	0.818
1948	0.454	0.814
1949	0.459	0.810
1950	0.425	0.797
1951	0.474	0.819
1952	0.434	0.792
1953	0.489	0.813
1954	0.440	0.789
1955	0.455	0.810
1956	0.456	0.807
1957	0.440	0.818
1958	0.458	0.794
1959	0.447	0.800
1960	0.480	0.808
1961	0.445	0.806
1962	0.496	0.814
1963	0.433	0.815
1964	0.454	0.804
1965	0.479	0.811
1966	0.442	0.805
1967	0.422	0.797
1968	0.462	0.805
1969	0.465	0.812
1970	0.457	0.810
1971	0.400	0.791
1972	0.452	0.802
1973	0.468	0.809
1974	0.458	0.806
1975	0.493	0.815
1976	0.399	0.803
1977	0.425	0.776
1978	0.448	0.804
1979	0.498	0.823
1980	0.455	0.810

1981	0.479	0.803
1982	0.481	0.816
1983	0.466	0.808
1984	0.424	0.799
1985	0.496	0.814
1986	0.426	0.797
1987	0.449	0.793
1988	0.428	0.791
1989	0.488	0.816
1990	0.471	0.815
1991	0.485	0.804
1992	0.450	0.802
1993	0.413	0.796
1994	0.422	0.788
1995	0.430	0.786
1996	0.480	0.816
1997	0.436	0.791
1998	0.476	0.811
1999	0.439	0.796
2000	0.498	0.812
2001	0.454	0.801
2002	0.467	0.813
2003	0.452	0.801
2004	0.448	0.796
2005	0.460	0.807
2006	0.466	0.801
2007	0.427	0.803
2008	0.496	0.810
2009	0.468	0.807
2010	0.423	0.796
2011	0.424	0.783
2012	0.418	0.790
2013	0.430	0.796
2014	0.493	0.809
2015	0.462	0.811
2016	0.430	0.797
2017	0.472	0.811
2018	0.419	0.799
2019	0.462	0.807
2020	0.453	0.804
2021	0.414	0.789
2022	0.468	0.815

2023	0.438	0.801
2024	0.378	0.768
2025	0.421	0.790
2026	0.493	0.811
2027	0.424	0.784
2028	0.412	0.818
2029	0.482	0.819
2030	0.468	0.797
2031	0.463	0.794
2032	0.465	0.801
2033	0.401	0.801
2034	0.483	0.812
2035	0.427	0.802
2036	0.490	0.815
2037	0.413	0.790
2038	0.474	0.800
2039	0.447	0.814
2040	0.491	0.805
2041	0.460	0.817
2042	0.456	0.827
2043	0.418	0.784
2044	0.472	0.796
2045	0.429	0.794
2046	0.421	0.788
2047	0.440	0.819
2048	0.467	0.799
2049	0.454	0.814
2050	0.439	0.815
2051	0.428	0.797
2052	0.450	0.797
2053	0.459	0.791
2054	0.473	0.809
2055	0.470	0.807
2056	0.424	0.789
2057	0.474	0.801
2058	0.430	0.789
2059	0.427	0.799
2060	0.423	0.785
2061	0.401	0.778
2062	0.472	0.813
2063	0.410	0.796
2064	0.457	0.789

2065	0.457	0.801
2066	0.491	0.801
2067	0.418	0.779
2068	0.438	0.792
2069	0.457	0.811
2070	0.466	0.804
2071	0.422	0.807
2072	0.436	0.807
2073	0.491	0.803
2074	0.504	0.826
2075	0.450	0.804
2076	0.467	0.798
2077	0.458	0.805
2078	0.416	0.789
2079	0.422	0.810
2080	0.460	0.800
2081	0.464	0.794
2082	0.476	0.813
2083	0.473	0.810
2084	0.486	0.806
2085	0.462	0.816
2086	0.478	0.814
2087	0.478	0.800
2088	0.467	0.804
2089	0.450	0.807
2090	0.436	0.796
2091	0.380	0.773
2092	0.473	0.812
2093	0.428	0.790
2094	0.445	0.804
2095	0.456	0.810
2096	0.495	0.820
2097	0.457	0.794
2098	0.463	0.813
2099	0.452	0.804
2100	0.494	0.817
2101	0.476	0.818
2102	0.439	0.800
2103	0.479	0.804
2104	0.454	0.792
2105	0.471	0.806
2106	0.439	0.806

2107	0.465	0.798
2108	0.451	0.784
2109	0.454	0.794
2110	0.423	0.799
2111	0.469	0.814
2112	0.454	0.812
2113	0.463	0.806
2114	0.419	0.795
2115	0.430	0.801
2116	0.398	0.786
2117	0.433	0.788
2118	0.450	0.809
2119	0.429	0.793
2120	0.443	0.801
2121	0.471	0.807
2122	0.436	0.800
2123	0.426	0.801
2124	0.454	0.804
2125	0.477	0.813
2126	0.469	0.803
2127	0.441	0.813
2128	0.454	0.800
2129	0.477	0.771
2130	0.466	0.804
2131	0.487	0.802
2132	0.420	0.796
2133	0.447	0.797
2134	0.417	0.803
2135	0.457	0.787
2136	0.446	0.791
2137	0.455	0.808
2138	0.429	0.787
2139	0.464	0.799
2140	0.462	0.809
2141	0.483	0.815
2142	0.429	0.805
2143	0.473	0.800
2144	0.424	0.792
2145	0.457	0.803
2146	0.399	0.795
2147	0.445	0.797
2148	0.384	0.778

2149	0.477	0.805
2150	0.480	0.802
2151	0.442	0.798
2152	0.473	0.811
2153	0.407	0.796
2154	0.424	0.809
2155	0.471	0.801
2156	0.443	0.798
2157	0.429	0.788
2158	0.396	0.788
2159	0.500	0.812
2160	0.475	0.811
2161	0.438	0.804
2162	0.482	0.800
2163	0.457	0.813
2164	0.394	0.785
2165	0.457	0.810
2166	0.489	0.815
2167	0.424	0.805
2168	0.414	0.800
2169	0.451	0.804
2170	0.441	0.799
2171	0.417	0.779
2172	0.440	0.793
2173	0.448	0.796
2174	0.443	0.790
2175	0.441	0.800
2176	0.418	0.794
2177	0.483	0.821
2178	0.455	0.797
2179	0.398	0.798
2180	0.470	0.805
2181	0.453	0.801
2182	0.458	0.799
2183	0.425	0.795
2184	0.400	0.802
2185	0.403	0.790
2186	0.484	0.803
2187	0.418	0.798
2188	0.422	0.783
2189	0.443	0.785
2190	0.412	0.795

2191	0.394	0.783
2192	0.454	0.799
2193	0.413	0.792
2194	0.447	0.803
2195	0.461	0.801
2196	0.413	0.776
2197	0.420	0.789
2198	0.438	0.800
2199	0.423	0.813
2200	0.479	0.802
2201	0.408	0.778
2202	0.447	0.810
2203	0.443	0.809
2204	0.442	0.803
2205	0.469	0.804
2206	0.422	0.785
2207	0.413	0.807
2208	0.511	0.812
2209	0.398	0.791
2210	0.471	0.794
2211	0.493	0.814
2212	0.402	0.782
2213	0.488	0.811
2214	0.455	0.790
2215	0.398	0.795
2216	0.442	0.800
2217	0.488	0.796
2218	0.430	0.804
2219	0.467	0.800
2220	0.439	0.808
2221	0.407	0.793
2222	0.396	0.787
2223	0.433	0.794
2224	0.468	0.812
2225	0.484	0.820
2226	0.440	0.807
2227	0.452	0.805
2228	0.478	0.812
2229	0.410	0.781
2230	0.446	0.812
2231	0.462	0.811
2232	0.468	0.795

2233	0.446	0.804
2234	0.440	0.806
2235	0.452	0.805
2236	0.440	0.774
2237	0.486	0.811
2238	0.473	0.800
2239	0.404	0.776
2240	0.485	0.797
2241	0.484	0.808
2242	0.462	0.816
2243	0.443	0.796
2244	0.439	0.804
2245	0.430	0.793
2246	0.437	0.782
2247	0.418	0.778
2248	0.427	0.789
2249	0.425	0.789
2250	0.445	0.804
2251	0.403	0.772
2252	0.438	0.811
2253	0.424	0.785
2254	0.430	0.790
2255	0.396	0.781
2256	0.466	0.794
2257	0.401	0.768
2258	0.424	0.794
2259	0.447	0.801
2260	0.426	0.792
2261	0.429	0.811
2262	0.476	0.805
2263	0.463	0.798
2264	0.461	0.796
2265	0.435	0.788
2266	0.473	0.798
2267	0.448	0.785
2268	0.438	0.790
2269	0.426	0.788
2270	0.433	0.796
2271	0.440	0.799
2272	0.420	0.806
2273	0.443	0.798
2274	0.440	0.799

2275	0.466	0.796
2276	0.463	0.800
2277	0.416	0.786
2278	0.436	0.799
2279	0.443	0.788
2280	0.430	0.793
2281	0.419	0.787
2282	0.490	0.806
2283	0.471	0.814
2284	0.445	0.811
2285	0.424	0.787
2286	0.437	0.800
2287	0.383	0.776
2288	0.498	0.791
2289	0.445	0.782
2290	0.446	0.800
2291	0.404	0.796
2292	0.402	0.785
2293	0.444	0.778
2294	0.395	0.802
2295	0.484	0.796
2296	0.413	0.777
2297	0.465	0.797
2298	0.419	0.803
2299	0.490	0.801
2300	0.503	0.821
2301	0.447	0.780
2302	0.468	0.803
2303	0.418	0.781
2304	0.510	0.826
2305	0.470	0.801
2306	0.442	0.790
2307	0.450	0.804
2308	0.454	0.798
2309	0.412	0.794
2310	0.451	0.797
2311	0.406	0.793
2312	0.427	0.795
2313	0.430	0.789
2314	0.457	0.793
2315	0.478	0.815
2316	0.470	0.803

2317	0.458	0.799
2318	0.439	0.793
2319	0.453	0.797
2320	0.470	0.792
2321	0.463	0.798
2322	0.443	0.789
2323	0.427	0.788
2324	0.451	0.802
2325	0.431	0.808
2326	0.432	0.803
2327	0.406	0.781
2328	0.471	0.792
2329	0.412	0.779
2330	0.420	0.799
2331	0.470	0.805
2332	0.422	0.797
2333	0.495	0.824
2334	0.434	0.809
2335	0.441	0.786
2336	0.461	0.792
2337	0.398	0.785
2338	0.460	0.802
2339	0.441	0.797
2340	0.442	0.796
2341	0.435	0.809
2342	0.483	0.801
2343	0.471	0.801
2344	0.406	0.787
2345	0.451	0.800
2346	0.430	0.793
2347	0.427	0.786
2348	0.435	0.791
2349	0.466	0.783
2350	0.436	0.782
2351	0.433	0.776
2352	0.436	0.774
2353	0.450	0.799
2354	0.421	0.796
2355	0.430	0.802
2356	0.463	0.797
2357	0.463	0.815
2358	0.427	0.791

2359	0.422	0.791
2360	0.405	0.788
2361	0.402	0.793
2362	0.458	0.796
2363	0.455	0.796
2364	0.410	0.785
2365	0.422	0.797
2366	0.426	0.789
2367	0.449	0.788
2368	0.456	0.807
2369	0.452	0.804
2370	0.420	0.800
2371	0.455	0.802
2372	0.469	0.801
2373	0.440	0.799
2374	0.473	0.810
2375	0.483	0.805
2376	0.413	0.784
2377	0.403	0.792
2378	0.458	0.801
2379	0.381	0.779
2380	0.464	0.809
2381	0.448	0.809
2382	0.465	0.784
2383	0.417	0.790
2384	0.424	0.779
2385	0.428	0.813
2386	0.476	0.805
2387	0.449	0.801
2388	0.457	0.806
2389	0.433	0.790
2390	0.454	0.792
2391	0.438	0.784
2392	0.454	0.794
2393	0.456	0.814
2394	0.463	0.799
2395	0.450	0.789
2396	0.437	0.800
2397	0.454	0.804
2398	0.418	0.804
2399	0.406	0.773
2400	0.446	0.786

2401	0.411	0.795
2402	0.417	0.786
2403	0.455	0.805
2404	0.432	0.793
2405	0.501	0.799
2406	0.424	0.811
2407	0.419	0.797
2408	0.439	0.792
2409	0.451	0.795
2410	0.441	0.799
2411	0.407	0.787
2412	0.407	0.781
2413	0.383	0.777
2414	0.407	0.808
2415	0.369	0.795
2416	0.428	0.809
2417	0.449	0.791
2418	0.445	0.784
2419	0.445	0.791
2420	0.468	0.809
2421	0.446	0.775
2422	0.503	0.823
2423	0.408	0.782
2424	0.509	0.827
2425	0.471	0.786
2426	0.403	0.798
2427	0.374	0.776
2428	0.411	0.789
2429	0.427	0.792
2430	0.479	0.807
2431	0.490	0.806
2432	0.450	0.808
2433	0.426	0.796
2434	0.466	0.809
2435	0.392	0.772
2436	0.434	0.775
2437	0.435	0.784
2438	0.452	0.787
2439	0.425	0.797
2440	0.491	0.806
2441	0.415	0.784
2442	0.382	0.777

2443	0.391	0.775
2444	0.444	0.784
2445	0.426	0.791
2446	0.423	0.791
2447	0.443	0.780
2448	0.511	0.810
2449	0.409	0.809
2450	0.418	0.797
2451	0.477	0.812
2452	0.416	0.780
2453	0.470	0.805
2454	0.432	0.794
2455	0.455	0.802
2456	0.487	0.806
2457	0.430	0.797
2458	0.463	0.795
2459	0.469	0.807
2460	0.463	0.798
2461	0.455	0.807
2462	0.464	0.823
2463	0.486	0.812
2464	0.407	0.796
2465	0.415	0.792
2466	0.422	0.778
2467	0.487	0.800
2468	0.467	0.797
2469	0.451	0.803
2470	0.430	0.763
2471	0.410	0.788
2472	0.428	0.780
2473	0.440	0.805
2474	0.434	0.803
2475	0.383	0.785
2476	0.425	0.791
2477	0.422	0.792
2478	0.449	0.807
2479	0.454	0.789
2480	0.446	0.801
2481	0.447	0.786
2482	0.455	0.804
2483	0.383	0.797
2484	0.452	0.810

2485	0.440	0.802
2486	0.419	0.789
2487	0.450	0.798
2488	0.455	0.797
2489	0.434	0.792
2490	0.416	0.792
2491	0.401	0.771
2492	0.429	0.796
2493	0.491	0.795
2494	0.458	0.803
2495	0.434	0.783
2496	0.446	0.787
2497	0.435	0.792
2498	0.440	0.812
2499	0.455	0.799
2500	0.429	0.774
2501	0.427	0.798
2502	0.434	0.786
2503	0.442	0.799
2504	0.416	0.771
2505	0.466	0.806
2506	0.463	0.801
2507	0.448	0.816
2508	0.485	0.810
2509	0.398	0.800
2510	0.427	0.799
2511	0.473	0.794
2512	0.465	0.797
2513	0.480	0.782
2514	0.424	0.803
2515	0.461	0.801
2516	0.476	0.792
2517	0.451	0.805
2518	0.429	0.778
2519	0.403	0.783
2520	0.460	0.810
2521	0.449	0.783
2522	0.433	0.798
2523	0.501	0.810
2524	0.482	0.796
2525	0.452	0.799
2526	0.439	0.785

2527	0.447	0.801
2528	0.451	0.798
2529	0.463	0.793
2530	0.458	0.796
2531	0.407	0.783
2532	0.464	0.793
2533	0.458	0.792
2534	0.454	0.791
2535	0.412	0.771
2536	0.418	0.800
2537	0.445	0.787
2538	0.445	0.781
2539	0.441	0.790
2540	0.411	0.780
2541	0.457	0.808
2542	0.430	0.797
2543	0.427	0.798
2544	0.410	0.785
2545	0.423	0.794
2546	0.425	0.794
2547	0.408	0.780
2548	0.467	0.804
2549	0.457	0.793
2550	0.426	0.790
2551	0.455	0.794
2552	0.396	0.794
2553	0.424	0.795
2554	0.442	0.796
2555	0.445	0.797
2556	0.455	0.791
2557	0.422	0.786
2558	0.444	0.787
2559	0.473	0.807
2560	0.401	0.777
2561	0.443	0.806
2562	0.435	0.789
2563	0.455	0.794
2564	0.419	0.784
2565	0.407	0.793
2566	0.490	0.793
2567	0.472	0.793
2568	0.416	0.763

2569	0.415	0.772
2570	0.472	0.805
2571	0.395	0.783
2572	0.438	0.802
2573	0.444	0.799
2574	0.449	0.795
2575	0.426	0.786
2576	0.426	0.783
2577	0.452	0.794
2578	0.444	0.801
2579	0.437	0.800
2580	0.445	0.775
2581	0.443	0.799
2582	0.431	0.784
2583	0.441	0.789
2584	0.457	0.805
2585	0.395	0.785
2586	0.444	0.810
2587	0.430	0.793
2588	0.431	0.781
2589	0.420	0.795
2590	0.451	0.793
2591	0.417	0.792
2592	0.415	0.775
2593	0.433	0.785
2594	0.449	0.804
2595	0.438	0.788
2596	0.418	0.787
2597	0.391	0.787
2598	0.396	0.781
2599	0.395	0.770
2600	0.458	0.804
2601	0.431	0.791
2602	0.438	0.800
2603	0.424	0.774
2604	0.480	0.792
2605	0.425	0.795
2606	0.437	0.791
2607	0.425	0.800
2608	0.404	0.772
2609	0.454	0.796
2610	0.427	0.781

2611	0.448	0.805
2612	0.428	0.778
2613	0.435	0.791
2614	0.430	0.788
2615	0.481	0.795
2616	0.398	0.777
2617	0.444	0.792
2618	0.424	0.775
2619	0.453	0.782
2620	0.448	0.793
2621	0.420	0.794
2622	0.419	0.769
2623	0.407	0.787
2624	0.424	0.781
2625	0.399	0.783
2626	0.387	0.791
2627	0.399	0.779
2628	0.444	0.797
2629	0.442	0.791
2630	0.442	0.785
2631	0.418	0.792
2632	0.390	0.785
2633	0.476	0.811
2634	0.456	0.805
2635	0.478	0.790
2636	0.427	0.789
2637	0.396	0.803
2638	0.422	0.776
2639	0.429	0.802
2640	0.426	0.784
2641	0.423	0.788
2642	0.448	0.815
2643	0.403	0.788
2644	0.422	0.790
2645	0.401	0.787
2646	0.429	0.782
2647	0.476	0.813
2648	0.445	0.764
2649	0.478	0.816
2650	0.437	0.801
2651	0.447	0.795
2652	0.438	0.804

2653	0.437	0.793
2654	0.400	0.778
2655	0.419	0.788
2656	0.431	0.791
2657	0.451	0.802
2658	0.426	0.782
2659	0.470	0.808
2660	0.438	0.799
2661	0.361	0.778
2662	0.428	0.796
2663	0.440	0.794
2664	0.434	0.776
2665	0.403	0.781
2666	0.458	0.805
2667	0.424	0.781
2668	0.424	0.771
2669	0.431	0.812
2670	0.425	0.778
2671	0.460	0.786
2672	0.424	0.766
2673	0.444	0.794
2674	0.405	0.775
2675	0.426	0.790
2676	0.442	0.783
2677	0.422	0.788
2678	0.406	0.781
2679	0.386	0.783
2680	0.387	0.768
2681	0.463	0.775
2682	0.452	0.796
2683	0.441	0.808
2684	0.461	0.787
2685	0.477	0.802
2686	0.420	0.785
2687	0.417	0.790
2688	0.388	0.771
2689	0.455	0.812
2690	0.407	0.774
2691	0.395	0.790
2692	0.423	0.804
2693	0.478	0.800
2694	0.446	0.796

2695	0.484	0.810
2696	0.463	0.798
2697	0.426	0.786
2698	0.465	0.796
2699	0.430	0.772
2700	0.451	0.804
2701	0.418	0.793
2702	0.450	0.793
2703	0.441	0.796
2704	0.477	0.784
2705	0.406	0.785
2706	0.379	0.781
2707	0.434	0.791
2708	0.444	0.773
2709	0.378	0.784
2710	0.420	0.782
2711	0.444	0.806
2712	0.401	0.781
2713	0.446	0.776
2714	0.421	0.783
2715	0.407	0.783
2716	0.474	0.804
2717	0.454	0.792
2718	0.358	0.781
2719	0.418	0.789
2720	0.424	0.780
2721	0.415	0.788
2722	0.462	0.794
2723	0.417	0.793
2724	0.426	0.794
2725	0.455	0.795
2726	0.383	0.783
2727	0.416	0.786
2728	0.434	0.798
2729	0.367	0.770
2730	0.457	0.797
2731	0.406	0.786
2732	0.447	0.797
2733	0.454	0.783
2734	0.420	0.771
2735	0.394	0.773
2736	0.435	0.788

2737	0.450	0.797
2738	0.403	0.783
2739	0.446	0.783
2740	0.436	0.773
2741	0.414	0.775
2742	0.436	0.773
2743	0.428	0.798
2744	0.417	0.781
2745	0.438	0.787
2746	0.450	0.804
2747	0.430	0.798
2748	0.454	0.784
2749	0.459	0.809
2750	0.424	0.774
2751	0.403	0.787
2752	0.437	0.772
2753	0.441	0.794
2754	0.400	0.762
2755	0.419	0.773
2756	0.448	0.804
2757	0.416	0.783
2758	0.411	0.779
2759	0.468	0.782
2760	0.419	0.790
2761	0.422	0.775
2762	0.457	0.796
2763	0.418	0.782
2764	0.402	0.765
2765	0.423	0.787
2766	0.401	0.798
2767	0.435	0.792
2768	0.433	0.779
2769	0.490	0.801
2770	0.426	0.782
2771	0.419	0.795
2772	0.481	0.781
2773	0.434	0.792
2774	0.407	0.776
2775	0.446	0.809
2776	0.362	0.778
2777	0.481	0.788
2778	0.429	0.789

2779	0.459	0.808
2780	0.445	0.788
2781	0.476	0.793
2782	0.399	0.780
2783	0.460	0.810
2784	0.423	0.779
2785	0.419	0.794
2786	0.407	0.794
2787	0.411	0.782
2788	0.406	0.785
2789	0.410	0.799
2790	0.406	0.776
2791	0.406	0.779
2792	0.438	0.790
2793	0.437	0.801
2794	0.405	0.791
2795	0.459	0.794
2796	0.436	0.775
2797	0.464	0.796
2798	0.470	0.784
2799	0.424	0.784
2800	0.483	0.803
2801	0.366	0.766
2802	0.413	0.780
2803	0.383	0.778
2804	0.473	0.791
2805	0.457	0.790
2806	0.467	0.813
2807	0.449	0.800
2808	0.419	0.810
2809	0.464	0.797
2810	0.407	0.767
2811	0.450	0.803
2812	0.411	0.772
2813	0.460	0.806
2814	0.383	0.769
2815	0.406	0.778
2816	0.462	0.808
2817	0.418	0.803
2818	0.420	0.780
2819	0.445	0.790
2820	0.359	0.754

2821	0.446	0.779
2822	0.412	0.782
2823	0.390	0.794
2824	0.463	0.799
2825	0.462	0.801
2826	0.431	0.779
2827	0.435	0.795
2828	0.398	0.793
2829	0.443	0.793
2830	0.452	0.779
2831	0.421	0.804
2832	0.427	0.789
2833	0.422	0.802
2834	0.470	0.805
2835	0.447	0.797
2836	0.441	0.799
2837	0.447	0.802
2838	0.401	0.785
2839	0.440	0.807
2840	0.395	0.794
2841	0.422	0.776
2842	0.408	0.789
2843	0.543	0.812
2844	0.508	0.795
2845	0.422	0.783
2846	0.462	0.792
2847	0.390	0.785
2848	0.434	0.792
2849	0.440	0.778
2850	0.386	0.785
2851	0.435	0.794
2852	0.393	0.766
2853	0.412	0.786
2854	0.467	0.804
2855	0.441	0.793
2856	0.451	0.792
2857	0.423	0.771
2858	0.373	0.762
2859	0.422	0.784
2860	0.379	0.771
2861	0.395	0.767
2862	0.416	0.778

2863	0.459	0.808
2864	0.454	0.794
2865	0.463	0.802
2866	0.451	0.791
2867	0.406	0.770
2868	0.440	0.787
2869	0.401	0.774
2870	0.428	0.799
2871	0.419	0.796
2872	0.445	0.791
2873	0.419	0.789
2874	0.387	0.775
2875	0.419	0.780
2876	0.435	0.798
2877	0.442	0.783
2878	0.403	0.786
2879	0.438	0.792
2880	0.450	0.807
2881	0.443	0.785
2882	0.429	0.792
2883	0.425	0.789
2884	0.408	0.773
2885	0.481	0.800
2886	0.406	0.797
2887	0.445	0.802
2888	0.427	0.773
2889	0.401	0.772
2890	0.421	0.789
2891	0.444	0.791
2892	0.432	0.795
2893	0.436	0.804
2894	0.420	0.769
2895	0.415	0.782
2896	0.438	0.790
2897	0.465	0.806
2898	0.460	0.791
2899	0.420	0.770
2900	0.383	0.773
2901	0.385	0.760
2902	0.383	0.759
2903	0.423	0.802
2904	0.399	0.776

2905	0.423	0.779
2906	0.467	0.784
2907	0.410	0.786
2908	0.406	0.789
2909	0.383	0.773
2910	0.468	0.784
2911	0.330	0.785
2912	0.429	0.786
2913	0.415	0.776
2914	0.492	0.813
2915	0.433	0.787
2916	0.411	0.768
2917	0.447	0.790
2918	0.377	0.779
2919	0.393	0.768
2920	0.418	0.785
2921	0.411	0.789
2922	0.467	0.804
2923	0.443	0.781
2924	0.425	0.778
2925	0.411	0.798
2926	0.410	0.787
2927	0.434	0.807
2928	0.439	0.800
2929	0.463	0.790
2930	0.416	0.776
2931	0.402	0.771
2932	0.438	0.802
2933	0.422	0.779
2934	0.497	0.795
2935	0.423	0.786
2936	0.448	0.788
2937	0.413	0.781
2938	0.395	0.787
2939	0.402	0.789
2940	0.402	0.773
2941	0.409	0.773
2942	0.463	0.783
2943	0.430	0.775
2944	0.388	0.775
2945	0.440	0.792
2946	0.406	0.769

2947	0.462	0.788
2948	0.431	0.780
2949	0.427	0.788
2950	0.388	0.783
2951	0.403	0.793
2952	0.423	0.794
2953	0.378	0.782
2954	0.401	0.772
2955	0.462	0.811
2956	0.415	0.779
2957	0.394	0.774
2958	0.471	0.796
2959	0.411	0.782
2960	0.435	0.788
2961	0.469	0.795
2962	0.445	0.790
2963	0.423	0.790
2964	0.441	0.805
2965	0.412	0.780
2966	0.378	0.764
2967	0.445	0.790
2968	0.448	0.790
2969	0.413	0.779
2970	0.418	0.782
2971	0.374	0.774
2972	0.479	0.809
2973	0.426	0.785
2974	0.441	0.784
2975	0.411	0.775
2976	0.471	0.793
2977	0.441	0.805
2978	0.436	0.783
2979	0.382	0.785
2980	0.436	0.784
2981	0.456	0.804
2982	0.458	0.794
2983	0.420	0.792
2984	0.357	0.757
2985	0.498	0.790
2986	0.440	0.796
2987	0.487	0.794
2988	0.374	0.772

2989	0.414	0.775
2990	0.366	0.765
2991	0.410	0.762
2992	0.425	0.791
2993	0.377	0.772
2994	0.427	0.781
2995	0.401	0.790
2996	0.424	0.796
2997	0.428	0.785
2998	0.499	0.805
2999	0.402	0.770
3000	0.476	0.774
3001	0.471	0.800
3002	0.467	0.800
3003	0.419	0.792
3004	0.418	0.786
3005	0.409	0.786
3006	0.433	0.785
3007	0.431	0.777
3008	0.386	0.784
3009	0.427	0.782
3010	0.427	0.789
3011	0.433	0.786
3012	0.475	0.783
3013	0.439	0.793
3014	0.458	0.785
3015	0.411	0.791
3016	0.383	0.772
3017	0.409	0.771
3018	0.441	0.784
3019	0.432	0.779
3020	0.394	0.763
3021	0.374	0.768
3022	0.449	0.783
3023	0.405	0.774
3024	0.445	0.789
3025	0.477	0.799
3026	0.361	0.764
3027	0.408	0.789
3028	0.446	0.785
3029	0.433	0.788
3030	0.437	0.798

3031	0.430	0.782
3032	0.454	0.773
3033	0.420	0.789
3034	0.451	0.787
3035	0.433	0.800
3036	0.428	0.795
3037	0.366	0.771
3038	0.379	0.774
3039	0.401	0.764
3040	0.389	0.762
3041	0.382	0.782
3042	0.413	0.793
3043	0.427	0.777
3044	0.429	0.770
3045	0.433	0.777
3046	0.420	0.784
3047	0.459	0.801
3048	0.493	0.800
3049	0.370	0.765
3050	0.426	0.788
3051	0.421	0.777
3052	0.475	0.778
3053	0.396	0.782
3054	0.431	0.796
3055	0.405	0.778
3056	0.442	0.775
3057	0.404	0.785
3058	0.454	0.786
3059	0.419	0.791
3060	0.429	0.794
3061	0.384	0.770
3062	0.447	0.784
3063	0.443	0.787
3064	0.445	0.804
3065	0.451	0.786
3066	0.446	0.782
3067	0.450	0.778
3068	0.388	0.779
3069	0.423	0.783
3070	0.382	0.776
3071	0.447	0.798
3072	0.415	0.779

3073	0.462	0.798
3074	0.424	0.782
3075	0.438	0.799
3076	0.438	0.797
3077	0.415	0.783
3078	0.497	0.809
3079	0.425	0.790
3080	0.410	0.778
3081	0.424	0.808
3082	0.434	0.786
3083	0.433	0.803
3084	0.369	0.758
3085	0.427	0.777
3086	0.374	0.784
3087	0.403	0.762
3088	0.427	0.779
3089	0.453	0.796
3090	0.427	0.777
3091	0.431	0.780
3092	0.424	0.772
3093	0.397	0.778
3094	0.361	0.764
3095	0.456	0.779
3096	0.439	0.781
3097	0.370	0.783
3098	0.422	0.783
3099	0.419	0.780
3100	0.439	0.786
3101	0.449	0.778
3102	0.446	0.799
3103	0.401	0.781
3104	0.397	0.768
3105	0.402	0.774
3106	0.454	0.804
3107	0.457	0.805
3108	0.445	0.800
3109	0.429	0.792
3110	0.475	0.803
3111	0.458	0.762
3112	0.430	0.791
3113	0.434	0.794
3114	0.418	0.773

3115	0.422	0.783
3116	0.396	0.779
3117	0.411	0.781
3118	0.397	0.773
3119	0.428	0.785
3120	0.436	0.790
3121	0.419	0.788
3122	0.423	0.790
3123	0.419	0.765
3124	0.417	0.784
3125	0.401	0.774
3126	0.428	0.767
3127	0.397	0.785
3128	0.432	0.798
3129	0.432	0.789
3130	0.409	0.771
3131	0.429	0.799
3132	0.403	0.782
3133	0.390	0.766
3134	0.419	0.798
3135	0.428	0.784
3136	0.361	0.761
3137	0.374	0.762
3138	0.440	0.784
3139	0.431	0.800
3140	0.403	0.771
3141	0.418	0.802
3142	0.432	0.779
3143	0.395	0.789
3144	0.417	0.786
3145	0.415	0.784
3146	0.428	0.778
3147	0.450	0.784
3148	0.428	0.768
3149	0.444	0.788
3150	0.408	0.791
3151	0.412	0.761
3152	0.417	0.793
3153	0.460	0.780
3154	0.426	0.788
3155	0.389	0.775
3156	0.436	0.783

3157	0.418	0.793
3158	0.437	0.782
3159	0.423	0.787
3160	0.406	0.778
3161	0.474	0.797
3162	0.418	0.780
3163	0.442	0.786
3164	0.443	0.796
3165	0.413	0.794
3166	0.401	0.795
3167	0.454	0.773
3168	0.418	0.772
3169	0.414	0.760
3170	0.402	0.762
3171	0.417	0.770
3172	0.440	0.780
3173	0.424	0.773
3174	0.392	0.774
3175	0.433	0.788
3176	0.388	0.791
3177	0.417	0.765
3178	0.434	0.783
3179	0.355	0.774
3180	0.401	0.790
3181	0.429	0.774
3182	0.459	0.805
3183	0.450	0.786
3184	0.442	0.791
3185	0.472	0.806
3186	0.431	0.793
3187	0.427	0.777
3188	0.473	0.785
3189	0.445	0.785
3190	0.437	0.792
3191	0.385	0.760
3192	0.440	0.777
3193	0.400	0.787
3194	0.428	0.784
3195	0.381	0.785
3196	0.457	0.806
3197	0.473	0.799
3198	0.436	0.795

3199	0.406	0.769
3200	0.459	0.787
3201	0.411	0.789
3202	0.392	0.773
3203	0.423	0.782
3204	0.399	0.774
3205	0.429	0.792
3206	0.387	0.771
3207	0.373	0.744
3208	0.419	0.778
3209	0.406	0.770
3210	0.400	0.763
3211	0.464	0.803
3212	0.427	0.782
3213	0.428	0.780
3214	0.431	0.773
3215	0.378	0.787
3216	0.464	0.793
3217	0.466	0.790
3218	0.450	0.787
3219	0.423	0.785
3220	0.443	0.780
3221	0.436	0.786
3222	0.439	0.798
3223	0.430	0.778
3224	0.413	0.795
3225	0.435	0.802
3226	0.438	0.793
3227	0.391	0.783
3228	0.378	0.767
3229	0.418	0.787
3230	0.384	0.782
3231	0.428	0.776
3232	0.394	0.763
3233	0.370	0.763
3234	0.414	0.782
3235	0.428	0.789
3236	0.420	0.778
3237	0.425	0.779
3238	0.424	0.796
3239	0.403	0.791
3240	0.376	0.765

3241	0.457	0.809
3242	0.413	0.782
3243	0.397	0.781
3244	0.436	0.796
3245	0.399	0.781
3246	0.373	0.767
3247	0.367	0.763
3248	0.458	0.797
3249	0.424	0.776
3250	0.411	0.785
3251	0.433	0.781
3252	0.412	0.794
3253	0.452	0.771
3254	0.447	0.806
3255	0.410	0.785
3256	0.385	0.778
3257	0.355	0.750
3258	0.366	0.767
3259	0.403	0.775
3260	0.415	0.770
3261	0.400	0.772
3262	0.401	0.785
3263	0.427	0.783
3264	0.414	0.780
3265	0.442	0.793
3266	0.383	0.785
3267	0.419	0.771
3268	0.448	0.789
3269	0.399	0.779
3270	0.464	0.793
3271	0.447	0.784
3272	0.420	0.794
3273	0.419	0.780
3274	0.406	0.776
3275	0.423	0.781
3276	0.483	0.799
3277	0.392	0.779
3278	0.383	0.777
3279	0.430	0.795
3280	0.398	0.763
3281	0.436	0.796
3282	0.408	0.789

3283	0.418	0.794
3284	0.376	0.766
3285	0.440	0.792
3286	0.395	0.768
3287	0.420	0.787
3288	0.442	0.774
3289	0.424	0.776
3290	0.394	0.772
3291	0.349	0.750
3292	0.411	0.776
3293	0.440	0.782
3294	0.454	0.763
3295	0.408	0.787
3296	0.377	0.776
3297	0.410	0.779
3298	0.350	0.764
3299	0.434	0.778
3300	0.413	0.792
3301	0.454	0.798
3302	0.394	0.787
3303	0.403	0.797
3304	0.380	0.771
3305	0.425	0.769
3306	0.457	0.790
3307	0.396	0.784
3308	0.442	0.795
3309	0.443	0.804
3310	0.392	0.778
3311	0.357	0.758
3312	0.446	0.777
3313	0.443	0.797
3314	0.425	0.784
3315	0.435	0.804
3316	0.440	0.788
3317	0.400	0.784
3318	0.465	0.776
3319	0.422	0.794
3320	0.430	0.792
3321	0.402	0.782
3322	0.419	0.771
3323	0.431	0.785
3324	0.401	0.764

3325	0.424	0.797
3326	0.426	0.790
3327	0.371	0.758
3328	0.468	0.790
3329	0.423	0.782
3330	0.353	0.766
3331	0.397	0.774
3332	0.369	0.768
3333	0.410	0.776
3334	0.445	0.805
3335	0.391	0.774
3336	0.393	0.772
3337	0.411	0.791
3338	0.453	0.768
3339	0.428	0.770
3340	0.384	0.762
3341	0.376	0.777
3342	0.434	0.798
3343	0.361	0.772
3344	0.503	0.798
3345	0.434	0.793
3346	0.417	0.772
3347	0.454	0.808
3348	0.366	0.770
3349	0.415	0.795
3350	0.472	0.810
3351	0.401	0.783
3352	0.400	0.800
3353	0.442	0.793
3354	0.378	0.781
3355	0.471	0.797
3356	0.419	0.771
3357	0.464	0.809
3358	0.383	0.779
3359	0.427	0.778
3360	0.381	0.777
3361	0.427	0.779
3362	0.428	0.778
3363	0.377	0.760
3364	0.417	0.771
3365	0.473	0.783
3366	0.418	0.771

3367	0.454	0.790
3368	0.415	0.777
3369	0.419	0.793
3370	0.404	0.771
3371	0.403	0.781
3372	0.453	0.790
3373	0.422	0.794
3374	0.392	0.772
3375	0.388	0.763
3376	0.399	0.780
3377	0.445	0.782
3378	0.391	0.760
3379	0.324	0.759
3380	0.417	0.791
3381	0.419	0.784
3382	0.462	0.791
3383	0.428	0.792
3384	0.409	0.781
3385	0.367	0.759
3386	0.412	0.762
3387	0.419	0.759
3388	0.431	0.777
3389	0.410	0.768
3390	0.441	0.788
3391	0.387	0.766
3392	0.403	0.763
3393	0.402	0.784
3394	0.402	0.773
3395	0.381	0.789
3396	0.383	0.764
3397	0.448	0.783
3398	0.394	0.768
3399	0.392	0.784
3400	0.441	0.788
3401	0.459	0.777
3402	0.390	0.774
3403	0.422	0.782
3404	0.417	0.774
3405	0.472	0.799
3406	0.390	0.783
3407	0.424	0.784
3408	0.409	0.795

3409	0.442	0.764
3410	0.416	0.776
3411	0.479	0.799
3412	0.478	0.796
3413	0.425	0.773
3414	0.439	0.770
3415	0.391	0.750
3416	0.463	0.785
3417	0.452	0.787
3418	0.407	0.775
3419	0.442	0.789
3420	0.426	0.775
3421	0.398	0.788
3422	0.394	0.777
3423	0.390	0.761
3424	0.387	0.774
3425	0.409	0.795
3426	0.452	0.782
3427	0.415	0.766
3428	0.415	0.780
3429	0.422	0.768
3430	0.377	0.768
3431	0.422	0.775
3432	0.466	0.785
3433	0.459	0.788
3434	0.381	0.783
3435	0.437	0.802
3436	0.398	0.769
3437	0.391	0.771
3438	0.417	0.776
3439	0.483	0.806
3440	0.395	0.777
3441	0.393	0.761
3442	0.404	0.767
3443	0.390	0.782
3444	0.346	0.779
3445	0.345	0.748
3446	0.415	0.782
3447	0.434	0.764
3448	0.421	0.782
3449	0.443	0.774
3450	0.376	0.751

3451	0.433	0.783
3452	0.415	0.766
3453	0.399	0.773
3454	0.417	0.791
3455	0.409	0.787
3456	0.412	0.797
3457	0.422	0.784
3458	0.389	0.773
3459	0.382	0.778
3460	0.392	0.788
3461	0.396	0.784
3462	0.373	0.768
3463	0.462	0.786
3464	0.406	0.767
3465	0.428	0.754
3466	0.405	0.763
3467	0.436	0.790
3468	0.374	0.773
3469	0.406	0.776
3470	0.422	0.774
3471	0.422	0.776
3472	0.424	0.781
3473	0.383	0.743
3474	0.438	0.789
3475	0.406	0.767
3476	0.450	0.776
3477	0.409	0.787
3478	0.409	0.785
3479	0.424	0.774
3480	0.424	0.781
3481	0.394	0.776
3482	0.348	0.771
3483	0.396	0.764
3484	0.375	0.767
3485	0.338	0.740
3486	0.396	0.765
3487	0.411	0.774
3488	0.388	0.766
3489	0.382	0.773
3490	0.418	0.791
3491	0.411	0.794
3492	0.415	0.776

3493	0.422	0.766
3494	0.390	0.764
3495	0.412	0.782
3496	0.486	0.801
3497	0.420	0.786
3498	0.403	0.765
3499	0.447	0.779
3500	0.392	0.768
3501	0.434	0.780
3502	0.419	0.756
3503	0.377	0.769
3504	0.462	0.797
3505	0.351	0.756
3506	0.395	0.785
3507	0.404	0.771
3508	0.403	0.774
3509	0.402	0.775
3510	0.359	0.761
3511	0.377	0.774
3512	0.431	0.786
3513	0.420	0.784
3514	0.419	0.787
3515	0.427	0.774
3516	0.412	0.776
3517	0.385	0.764
3518	0.334	0.753
3519	0.398	0.775
3520	0.418	0.748
3521	0.404	0.777
3522	0.384	0.769
3523	0.412	0.766
3524	0.402	0.777
3525	0.440	0.784
3526	0.437	0.798
3527	0.441	0.781
3528	0.371	0.762
3529	0.421	0.766
3530	0.417	0.783
3531	0.433	0.789
3532	0.455	0.781
3533	0.439	0.790
3534	0.395	0.784

3535	0.419	0.781
3536	0.434	0.790
3537	0.373	0.751
3538	0.395	0.771
3539	0.450	0.778
3540	0.379	0.786
3541	0.422	0.770
3542	0.402	0.771
3543	0.403	0.786
3544	0.391	0.786
3545	0.382	0.760
3546	0.375	0.765
3547	0.428	0.794
3548	0.414	0.761
3549	0.441	0.775
3550	0.366	0.759
3551	0.403	0.784
3552	0.417	0.771
3553	0.415	0.779
3554	0.376	0.764
3555	0.424	0.770
3556	0.397	0.773
3557	0.398	0.757
3558	0.347	0.752
3559	0.407	0.777
3560	0.441	0.771
3561	0.374	0.758
3562	0.433	0.789
3563	0.430	0.792
3564	0.387	0.778
3565	0.433	0.788
3566	0.367	0.780
3567	0.394	0.769
3568	0.385	0.778
3569	0.417	0.781
3570	0.381	0.772
3571	0.422	0.765
3572	0.455	0.803
3573	0.426	0.791
3574	0.426	0.771
3575	0.428	0.785
3576	0.397	0.765

3577	0.429	0.761
3578	0.326	0.752
3579	0.439	0.782
3580	0.418	0.768
3581	0.410	0.772
3582	0.378	0.768
3583	0.378	0.764
3584	0.449	0.777
3585	0.383	0.755
3586	0.355	0.746
3587	0.483	0.798
3588	0.427	0.790
3589	0.436	0.785
3590	0.412	0.767
3591	0.453	0.785
3592	0.422	0.765
3593	0.443	0.785
3594	0.467	0.792
3595	0.418	0.775
3596	0.362	0.763
3597	0.325	0.753
3598	0.349	0.751
3599	0.394	0.774
3600	0.436	0.785
3601	0.419	0.764
3602	0.422	0.783
3603	0.354	0.777
3604	0.387	0.761
3605	0.474	0.800
3606	0.429	0.770
3607	0.370	0.756
3608	0.404	0.774
3609	0.438	0.780
3610	0.391	0.772
3611	0.385	0.752
3612	0.394	0.787
3613	0.353	0.753
3614	0.390	0.775
3615	0.429	0.767
3616	0.399	0.776
3617	0.366	0.784
3618	0.426	0.786

3619	0.432	0.795
3620	0.448	0.772
3621	0.412	0.798
3622	0.384	0.765
3623	0.409	0.778
3624	0.427	0.779
3625	0.412	0.790
3626	0.403	0.774
3627	0.424	0.779
3628	0.409	0.766
3629	0.409	0.752
3630	0.457	0.790
3631	0.401	0.775
3632	0.379	0.781
3633	0.412	0.792
3634	0.400	0.773
3635	0.395	0.789
3636	0.448	0.767
3637	0.422	0.780
3638	0.371	0.761
3639	0.394	0.770
3640	0.393	0.774
3641	0.436	0.785
3642	0.400	0.780
3643	0.448	0.794
3644	0.417	0.771
3645	0.358	0.743
3646	0.395	0.770
3647	0.428	0.794
3648	0.421	0.785
3649	0.436	0.773
3650	0.361	0.770
3651	0.401	0.769
3652	0.399	0.784
3653	0.371	0.758
3654	0.423	0.778
3655	0.432	0.771
3656	0.445	0.791
3657	0.401	0.776
3658	0.406	0.780
3659	0.438	0.769
3660	0.383	0.769

3661	0.443	0.791
3662	0.425	0.794
3663	0.406	0.764
3664	0.397	0.775
3665	0.422	0.776
3666	0.394	0.764
3667	0.433	0.780
3668	0.399	0.776
3669	0.399	0.761
3670	0.383	0.782
3671	0.388	0.775
3672	0.422	0.786
3673	0.368	0.754
3674	0.440	0.781
3675	0.364	0.765
3676	0.393	0.759
3677	0.420	0.780
3678	0.367	0.763
3679	0.376	0.772
3680	0.366	0.761
3681	0.415	0.783
3682	0.410	0.775
3683	0.406	0.788
3684	0.372	0.765
3685	0.454	0.795
3686	0.374	0.761
3687	0.342	0.753
3688	0.430	0.793
3689	0.383	0.782
3690	0.393	0.768
3691	0.430	0.771
3692	0.340	0.760
3693	0.386	0.766
3694	0.414	0.791
3695	0.398	0.778
3696	0.387	0.775
3697	0.413	0.766
3698	0.426	0.780
3699	0.438	0.787
3700	0.363	0.757
3701	0.407	0.774
3702	0.427	0.781

3703	0.419	0.780
3704	0.416	0.770
3705	0.334	0.732
3706	0.374	0.775
3707	0.432	0.797
3708	0.387	0.769
3709	0.362	0.752
3710	0.408	0.766
3711	0.354	0.771
3712	0.367	0.754
3713	0.456	0.805
3714	0.394	0.774
3715	0.426	0.788
3716	0.428	0.787
3717	0.431	0.781
3718	0.421	0.758
3719	0.410	0.775
3720	0.419	0.772
3721	0.435	0.769
3722	0.407	0.777
3723	0.408	0.773
3724	0.347	0.757
3725	0.427	0.763
3726	0.407	0.770
3727	0.413	0.774
3728	0.413	0.780
3729	0.418	0.768
3730	0.368	0.773
3731	0.404	0.792
3732	0.461	0.775
3733	0.445	0.775
3734	0.466	0.796
3735	0.385	0.768
3736	0.474	0.791
3737	0.422	0.759
3738	0.401	0.786
3739	0.438	0.780
3740	0.413	0.787
3741	0.388	0.766
3742	0.377	0.780
3743	0.446	0.782
3744	0.402	0.782

3745	0.370	0.767
3746	0.424	0.769
3747	0.367	0.745
3748	0.396	0.781
3749	0.410	0.774
3750	0.447	0.787
3751	0.422	0.794
3752	0.399	0.785
3753	0.419	0.779
3754	0.338	0.749
3755	0.384	0.767
3756	0.406	0.766
3757	0.350	0.749
3758	0.417	0.778
3759	0.442	0.781
3760	0.439	0.802
3761	0.426	0.787
3762	0.403	0.756
3763	0.462	0.776
3764	0.373	0.751
3765	0.420	0.779
3766	0.438	0.776
3767	0.403	0.769
3768	0.399	0.785
3769	0.390	0.757
3770	0.460	0.789
3771	0.444	0.784
3772	0.429	0.779
3773	0.376	0.754
3774	0.410	0.773
3775	0.398	0.766
3776	0.438	0.784
3777	0.404	0.772
3778	0.368	0.747
3779	0.387	0.767
3780	0.373	0.774
3781	0.450	0.777
3782	0.419	0.778
3783	0.364	0.763
3784	0.366	0.763
3785	0.442	0.781
3786	0.341	0.762

3787	0.367	0.762
3788	0.429	0.765
3789	0.405	0.773
3790	0.408	0.767
3791	0.420	0.773
3792	0.409	0.762
3793	0.417	0.785
3794	0.418	0.767
3795	0.430	0.770
3796	0.389	0.786
3797	0.445	0.801
3798	0.386	0.746
3799	0.411	0.768
3800	0.397	0.778
3801	0.425	0.793
3802	0.383	0.756
3803	0.443	0.774
3804	0.424	0.760
3805	0.383	0.770
3806	0.456	0.778
3807	0.400	0.767
3808	0.409	0.776
3809	0.415	0.759
3810	0.427	0.780
3811	0.342	0.745
3812	0.379	0.758
3813	0.404	0.777
3814	0.433	0.798
3815	0.438	0.765
3816	0.409	0.779
3817	0.399	0.758
3818	0.385	0.776
3819	0.431	0.781
3820	0.370	0.763
3821	0.437	0.778
3822	0.363	0.756
3823	0.412	0.784
3824	0.424	0.783
3825	0.399	0.769
3826	0.413	0.779
3827	0.417	0.775
3828	0.403	0.777

3829	0.403	0.759
3830	0.387	0.775
3831	0.396	0.759
3832	0.371	0.782
3833	0.415	0.760
3834	0.414	0.779
3835	0.385	0.786
3836	0.379	0.753
3837	0.357	0.759
3838	0.407	0.771
3839	0.325	0.751
3840	0.378	0.761
3841	0.402	0.768
3842	0.412	0.765
3843	0.367	0.765
3844	0.392	0.777
3845	0.394	0.767
3846	0.383	0.769
3847	0.452	0.766
3848	0.374	0.781
3849	0.387	0.764
3850	0.392	0.764
3851	0.406	0.759
3852	0.415	0.780
3853	0.394	0.769
3854	0.405	0.778
3855	0.388	0.763
3856	0.399	0.792
3857	0.439	0.789
3858	0.389	0.778
3859	0.445	0.757
3860	0.413	0.771
3861	0.394	0.761
3862	0.431	0.772
3863	0.427	0.778
3864	0.447	0.791
3865	0.425	0.771
3866	0.379	0.764
3867	0.405	0.773
3868	0.391	0.761
3869	0.363	0.758
3870	0.366	0.760

3871	0.375	0.771
3872	0.414	0.771
3873	0.372	0.758
3874	0.378	0.767
3875	0.416	0.778
3876	0.439	0.768
3877	0.385	0.777
3878	0.412	0.762
3879	0.391	0.771
3880	0.404	0.760
3881	0.405	0.778
3882	0.427	0.780
3883	0.387	0.752
3884	0.424	0.787
3885	0.395	0.764
3886	0.436	0.778
3887	0.448	0.764
3888	0.388	0.782
3889	0.380	0.771
3890	0.407	0.764
3891	0.433	0.776
3892	0.391	0.757
3893	0.416	0.761
3894	0.437	0.784
3895	0.414	0.772
3896	0.419	0.787
3897	0.425	0.788
3898	0.414	0.774
3899	0.363	0.757
3900	0.407	0.770
3901	0.378	0.744
3902	0.382	0.769
3903	0.375	0.763
3904	0.406	0.782
3905	0.410	0.789
3906	0.400	0.770
3907	0.396	0.770
3908	0.381	0.756
3909	0.458	0.785
3910	0.404	0.774
3911	0.370	0.765
3912	0.405	0.773

3913	0.380	0.769
3914	0.407	0.764
3915	0.369	0.760
3916	0.406	0.759
3917	0.441	0.789
3918	0.360	0.750
3919	0.425	0.791
3920	0.427	0.785
3921	0.444	0.787
3922	0.365	0.753
3923	0.436	0.782
3924	0.427	0.780
3925	0.399	0.770
3926	0.362	0.767
3927	0.419	0.789
3928	0.426	0.792
3929	0.431	0.783
3930	0.438	0.770
3931	0.440	0.787
3932	0.377	0.768
3933	0.362	0.763
3934	0.377	0.753
3935	0.444	0.788
3936	0.390	0.764
3937	0.350	0.756
3938	0.438	0.772
3939	0.345	0.764
3940	0.417	0.790
3941	0.406	0.760
3942	0.422	0.773
3943	0.403	0.766
3944	0.395	0.770
3945	0.398	0.767
3946	0.322	0.740
3947	0.429	0.774
3948	0.425	0.782
3949	0.353	0.765
3950	0.442	0.777
3951	0.398	0.764
3952	0.393	0.762
3953	0.414	0.774
3954	0.361	0.771

3955	0.407	0.779
3956	0.394	0.774
3957	0.404	0.788
3958	0.343	0.758
3959	0.378	0.758
3960	0.321	0.752
3961	0.433	0.776
3962	0.388	0.770
3963	0.409	0.778
3964	0.412	0.767
3965	0.380	0.762
3966	0.397	0.758
3967	0.365	0.743
3968	0.390	0.779
3969	0.380	0.760
3970	0.448	0.803
3971	0.409	0.772
3972	0.390	0.763
3973	0.375	0.765
3974	0.404	0.776
3975	0.383	0.773
3976	0.362	0.758
3977	0.406	0.770
3978	0.405	0.768
3979	0.368	0.757
3980	0.435	0.783
3981	0.407	0.779
3982	0.405	0.772
3983	0.371	0.762
3984	0.442	0.784
3985	0.385	0.756
3986	0.423	0.765
3987	0.401	0.779
3988	0.431	0.773
3989	0.427	0.784
3990	0.411	0.775
3991	0.352	0.780
3992	0.400	0.769
3993	0.387	0.760
3994	0.385	0.782
3995	0.419	0.777
3996	0.433	0.774

3997	0.398	0.750
3998	0.387	0.776
3999	0.401	0.786
4000	0.427	0.775
4001	0.362	0.765
4002	0.378	0.761
4003	0.455	0.781
4004	0.403	0.771
4005	0.400	0.777
4006	0.385	0.782
4007	0.398	0.769
4008	0.388	0.766
4009	0.370	0.765
4010	0.383	0.765
4011	0.395	0.768
4012	0.413	0.781
4013	0.336	0.756
4014	0.370	0.768
4015	0.395	0.766
4016	0.390	0.772
4017	0.401	0.763
4018	0.469	0.790
4019	0.349	0.768
4020	0.394	0.777
4021	0.411	0.766
4022	0.425	0.765
4023	0.405	0.789
4024	0.405	0.779
4025	0.411	0.776
4026	0.373	0.775
4027	0.377	0.774
4028	0.354	0.750
4029	0.381	0.783
4030	0.443	0.785
4031	0.381	0.769
4032	0.368	0.762
4033	0.412	0.786
4034	0.412	0.770
4035	0.408	0.783
4036	0.455	0.776
4037	0.397	0.762
4038	0.406	0.754

4039	0.393	0.776
4040	0.406	0.750
4041	0.454	0.772
4042	0.378	0.768
4043	0.358	0.764
4044	0.423	0.783
4045	0.347	0.743
4046	0.430	0.798
4047	0.374	0.770
4048	0.423	0.777
4049	0.341	0.755
4050	0.400	0.778
4051	0.402	0.773
4052	0.440	0.782
4053	0.407	0.780
4054	0.338	0.767
4055	0.368	0.761
4056	0.428	0.769
4057	0.374	0.769
4058	0.425	0.791
4059	0.416	0.765
4060	0.427	0.789
4061	0.374	0.761
4062	0.408	0.773
4063	0.370	0.765
4064	0.387	0.764
4065	0.374	0.763
4066	0.380	0.777
4067	0.368	0.759
4068	0.373	0.763
4069	0.388	0.770
4070	0.405	0.778
4071	0.383	0.761
4072	0.429	0.768
4073	0.429	0.777
4074	0.411	0.772
4075	0.402	0.765
4076	0.384	0.762
4077	0.399	0.753
4078	0.423	0.783
4079	0.446	0.770
4080	0.390	0.765

4081	0.374	0.741
4082	0.446	0.786
4083	0.424	0.759
4084	0.347	0.769
4085	0.424	0.770
4086	0.415	0.750
4087	0.363	0.755
4088	0.418	0.768
4089	0.396	0.739
4090	0.350	0.749
4091	0.379	0.771
4092	0.351	0.754
4093	0.409	0.768
4094	0.404	0.769
4095	0.400	0.778
4096	0.353	0.752
4097	0.345	0.739
4098	0.387	0.763
4099	0.420	0.776
4100	0.373	0.740
4101	0.381	0.749
4102	0.427	0.781
4103	0.420	0.748
4104	0.404	0.779
4105	0.378	0.746
4106	0.416	0.773
4107	0.382	0.770
4108	0.399	0.779
4109	0.365	0.756
4110	0.428	0.779
4111	0.362	0.760
4112	0.377	0.763
4113	0.441	0.770
4114	0.354	0.749
4115	0.408	0.774
4116	0.403	0.766
4117	0.383	0.779
4118	0.447	0.785
4119	0.337	0.740
4120	0.404	0.774
4121	0.466	0.776
4122	0.393	0.770

4123	0.409	0.772
4124	0.377	0.764
4125	0.397	0.769
4126	0.427	0.762
4127	0.424	0.790
4128	0.361	0.768
4129	0.406	0.759
4130	0.438	0.773
4131	0.403	0.784
4132	0.370	0.755
4133	0.422	0.782
4134	0.426	0.776
4135	0.424	0.772
4136	0.426	0.782
4137	0.378	0.762
4138	0.411	0.763
4139	0.383	0.764
4140	0.356	0.748
4141	0.418	0.754
4142	0.390	0.780
4143	0.381	0.749
4144	0.382	0.774
4145	0.424	0.786
4146	0.384	0.779
4147	0.457	0.782
4148	0.397	0.775
4149	0.338	0.768
4150	0.425	0.774
4151	0.357	0.772
4152	0.422	0.776
4153	0.425	0.754
4154	0.397	0.778
4155	0.370	0.757
4156	0.389	0.756
4157	0.396	0.765
4158	0.386	0.754
4159	0.378	0.755
4160	0.408	0.771
4161	0.334	0.751
4162	0.367	0.767
4163	0.436	0.771
4164	0.426	0.780

4165	0.382	0.757
4166	0.452	0.771
4167	0.334	0.736
4168	0.447	0.803
4169	0.462	0.800
4170	0.407	0.757
4171	0.395	0.779
4172	0.424	0.764
4173	0.426	0.794
4174	0.414	0.775
4175	0.382	0.763
4176	0.370	0.765
4177	0.333	0.749
4178	0.406	0.754
4179	0.425	0.773
4180	0.382	0.779
4181	0.411	0.760
4182	0.366	0.767
4183	0.422	0.774
4184	0.383	0.761
4185	0.351	0.763
4186	0.350	0.740
4187	0.394	0.762
4188	0.400	0.777
4189	0.387	0.755
4190	0.416	0.754
4191	0.434	0.764
4192	0.399	0.776
4193	0.440	0.760
4194	0.425	0.771
4195	0.409	0.770
4196	0.412	0.783
4197	0.304	0.727
4198	0.373	0.769
4199	0.390	0.763
4200	0.391	0.776
4201	0.418	0.759
4202	0.400	0.755
4203	0.405	0.770
4204	0.359	0.763
4205	0.408	0.757
4206	0.393	0.766

4207	0.366	0.770
4208	0.351	0.730
4209	0.440	0.759
4210	0.408	0.746
4211	0.375	0.772
4212	0.429	0.775
4213	0.378	0.754
4214	0.412	0.761
4215	0.370	0.739
4216	0.345	0.755
4217	0.371	0.756
4218	0.421	0.769
4219	0.397	0.775
4220	0.418	0.767
4221	0.398	0.764
4222	0.384	0.765
4223	0.431	0.775
4224	0.420	0.785
4225	0.446	0.761
4226	0.404	0.766
4227	0.406	0.774
4228	0.445	0.784
4229	0.426	0.767
4230	0.375	0.760
4231	0.426	0.783
4232	0.435	0.767
4233	0.347	0.775
4234	0.385	0.755
4235	0.423	0.778
4236	0.362	0.763
4237	0.419	0.779
4238	0.457	0.787
4239	0.372	0.767
4240	0.418	0.778
4241	0.422	0.783
4242	0.390	0.778
4243	0.399	0.775
4244	0.406	0.765
4245	0.446	0.781
4246	0.372	0.746
4247	0.398	0.759
4248	0.420	0.787

4249	0.415	0.762
4250	0.406	0.769
4251	0.374	0.762
4252	0.418	0.775
4253	0.411	0.767
4254	0.367	0.779
4255	0.376	0.755
4256	0.413	0.787
4257	0.420	0.783
4258	0.371	0.758
4259	0.376	0.780
4260	0.400	0.748
4261	0.365	0.757
4262	0.398	0.772
4263	0.383	0.768
4264	0.422	0.778
4265	0.397	0.757
4266	0.369	0.779
4267	0.404	0.785
4268	0.431	0.784
4269	0.420	0.753
4270	0.366	0.765
4271	0.424	0.782
4272	0.473	0.781
4273	0.364	0.761
4274	0.414	0.778
4275	0.455	0.796
4276	0.379	0.753
4277	0.415	0.766
4278	0.398	0.769
4279	0.359	0.761
4280	0.422	0.781
4281	0.396	0.764
4282	0.404	0.775
4283	0.414	0.754
4284	0.390	0.754
4285	0.417	0.767
4286	0.371	0.763
4287	0.386	0.745
4288	0.407	0.775
4289	0.384	0.761
4290	0.342	0.767

4291	0.425	0.768
4292	0.367	0.766
4293	0.390	0.752
4294	0.432	0.771
4295	0.383	0.756
4296	0.364	0.755
4297	0.404	0.762
4298	0.394	0.773
4299	0.377	0.779
4300	0.399	0.774
4301	0.386	0.782
4302	0.393	0.770
4303	0.387	0.760
4304	0.392	0.762
4305	0.362	0.759
4306	0.387	0.766
4307	0.428	0.764
4308	0.436	0.755
4309	0.387	0.750
4310	0.394	0.748
4311	0.342	0.749
4312	0.398	0.754
4313	0.350	0.763
4314	0.381	0.764
4315	0.383	0.764
4316	0.370	0.739
4317	0.411	0.754
4318	0.398	0.760
4319	0.397	0.764
4320	0.365	0.752
4321	0.395	0.757
4322	0.374	0.762
4323	0.445	0.785
4324	0.374	0.766
4325	0.317	0.747
4326	0.341	0.743
4327	0.381	0.755
4328	0.426	0.766
4329	0.412	0.781
4330	0.370	0.771
4331	0.387	0.765
4332	0.422	0.768

4333	0.350	0.748
4334	0.428	0.778
4335	0.381	0.759
4336	0.467	0.765
4337	0.406	0.781
4338	0.403	0.758
4339	0.410	0.773
4340	0.408	0.759
4341	0.410	0.762
4342	0.381	0.759
4343	0.436	0.796
4344	0.412	0.774
4345	0.406	0.761
4346	0.363	0.770
4347	0.391	0.758
4348	0.384	0.760
4349	0.382	0.747
4350	0.366	0.752
4351	0.431	0.778
4352	0.346	0.747
4353	0.415	0.771
4354	0.410	0.759
4355	0.396	0.777
4356	0.406	0.767
4357	0.387	0.770
4358	0.364	0.757
4359	0.357	0.762
4360	0.415	0.784
4361	0.460	0.783
4362	0.425	0.777
4363	0.427	0.764
4364	0.358	0.749
4365	0.409	0.756
4366	0.362	0.748
4367	0.372	0.747
4368	0.377	0.750
4369	0.405	0.775
4370	0.392	0.749
4371	0.421	0.764
4372	0.428	0.760
4373	0.461	0.787
4374	0.369	0.743

4375	0.441	0.773
4376	0.358	0.749
4377	0.407	0.766
4378	0.439	0.784
4379	0.367	0.757
4380	0.423	0.786
4381	0.442	0.773
4382	0.360	0.766
4383	0.400	0.771
4384	0.358	0.745
4385	0.440	0.773
4386	0.363	0.750
4387	0.409	0.749
4388	0.412	0.751
4389	0.355	0.752
4390	0.371	0.759
4391	0.386	0.775
4392	0.373	0.768
4393	0.350	0.747
4394	0.413	0.786
4395	0.424	0.766
4396	0.397	0.773
4397	0.346	0.745
4398	0.405	0.774
4399	0.406	0.766
4400	0.435	0.773
4401	0.439	0.762
4402	0.396	0.772
4403	0.401	0.773
4404	0.416	0.749
4405	0.378	0.750
4406	0.410	0.760
4407	0.373	0.759
4408	0.434	0.781
4409	0.374	0.767
4410	0.392	0.756
4411	0.415	0.775
4412	0.440	0.772
4413	0.375	0.769
4414	0.415	0.767
4415	0.382	0.773
4416	0.362	0.762

4417	0.355	0.748
4418	0.421	0.767
4419	0.387	0.765
4420	0.400	0.765
4421	0.378	0.753
4422	0.407	0.764
4423	0.365	0.757
4424	0.391	0.785
4425	0.407	0.777
4426	0.387	0.759
4427	0.395	0.757
4428	0.338	0.746
4429	0.361	0.760
4430	0.354	0.745
4431	0.397	0.758
4432	0.396	0.775
4433	0.425	0.773
4434	0.428	0.774
4435	0.380	0.773
4436	0.402	0.773
4437	0.382	0.754
4438	0.376	0.768
4439	0.374	0.760
4440	0.408	0.768
4441	0.424	0.791
4442	0.438	0.782
4443	0.350	0.748
4444	0.419	0.771
4445	0.425	0.779
4446	0.350	0.747
4447	0.429	0.763
4448	0.402	0.769
4449	0.414	0.781
4450	0.325	0.775
4451	0.417	0.764
4452	0.386	0.772
4453	0.406	0.757
4454	0.430	0.774
4455	0.358	0.755
4456	0.411	0.767
4457	0.433	0.793
4458	0.409	0.767

4459	0.392	0.770
4460	0.403	0.768
4461	0.440	0.768
4462	0.400	0.784
4463	0.347	0.742
4464	0.336	0.759
4465	0.352	0.756
4466	0.348	0.752
4467	0.395	0.770
4468	0.417	0.783
4469	0.378	0.757
4470	0.401	0.764
4471	0.346	0.751
4472	0.416	0.762
4473	0.392	0.770
4474	0.384	0.798
4475	0.372	0.742
4476	0.451	0.774
4477	0.422	0.776
4478	0.350	0.762
4479	0.321	0.740
4480	0.374	0.769
4481	0.341	0.757
4482	0.365	0.760
4483	0.383	0.764
4484	0.415	0.769
4485	0.359	0.751
4486	0.359	0.753
4487	0.382	0.766
4488	0.385	0.778
4489	0.375	0.755
4490	0.358	0.748
4491	0.404	0.758
4492	0.350	0.753
4493	0.387	0.758
4494	0.410	0.763
4495	0.391	0.764
4496	0.382	0.772
4497	0.432	0.781
4498	0.375	0.749
4499	0.389	0.755
4500	0.410	0.785

4501	0.460	0.772
4502	0.425	0.772
4503	0.381	0.749
4504	0.361	0.750
4505	0.370	0.752
4506	0.392	0.770
4507	0.416	0.765
4508	0.420	0.790
4509	0.346	0.749
4510	0.396	0.751
4511	0.416	0.765
4512	0.384	0.753
4513	0.397	0.770
4514	0.394	0.762
4515	0.441	0.763
4516	0.395	0.768
4517	0.370	0.749
4518	0.350	0.773
4519	0.432	0.776
4520	0.399	0.756
4521	0.379	0.752
4522	0.388	0.765
4523	0.382	0.758
4524	0.361	0.744
4525	0.356	0.747
4526	0.416	0.775
4527	0.415	0.773
4528	0.385	0.756
4529	0.318	0.760
4530	0.362	0.751
4531	0.382	0.757
4532	0.345	0.770
4533	0.356	0.740
4534	0.432	0.762
4535	0.369	0.747
4536	0.390	0.767
4537	0.377	0.754
4538	0.402	0.758
4539	0.403	0.774
4540	0.417	0.777
4541	0.399	0.771
4542	0.392	0.753

4543	0.429	0.767
4544	0.430	0.771
4545	0.382	0.766
4546	0.348	0.725
4547	0.383	0.745
4548	0.368	0.784
4549	0.424	0.781
4550	0.394	0.761
4551	0.417	0.793
4552	0.349	0.750
4553	0.435	0.760
4554	0.424	0.777
4555	0.358	0.749
4556	0.360	0.766
4557	0.373	0.754
4558	0.336	0.752
4559	0.419	0.752
4560	0.378	0.765
4561	0.394	0.775
4562	0.375	0.770
4563	0.430	0.762
4564	0.355	0.756
4565	0.418	0.760
4566	0.399	0.767
4567	0.404	0.768
4568	0.387	0.764
4569	0.384	0.758
4570	0.360	0.760
4571	0.419	0.743
4572	0.387	0.740
4573	0.379	0.765
4574	0.380	0.749
4575	0.363	0.745
4576	0.381	0.778
4577	0.393	0.765
4578	0.360	0.749
4579	0.439	0.782
4580	0.444	0.766
4581	0.400	0.762
4582	0.391	0.768
4583	0.358	0.745
4584	0.416	0.781

4585	0.389	0.762
4586	0.375	0.771
4587	0.385	0.753
4588	0.364	0.739
4589	0.396	0.761
4590	0.456	0.784
4591	0.441	0.789
4592	0.412	0.787
4593	0.426	0.781
4594	0.386	0.763
4595	0.444	0.777
4596	0.368	0.765
4597	0.366	0.751
4598	0.334	0.730
4599	0.421	0.766
4600	0.409	0.783
4601	0.437	0.771
4602	0.320	0.740
4603	0.432	0.760
4604	0.419	0.770
4605	0.387	0.747
4606	0.364	0.758
4607	0.368	0.750
4608	0.397	0.756
4609	0.397	0.780
4610	0.352	0.759
4611	0.419	0.775
4612	0.388	0.787
4613	0.368	0.758
4614	0.417	0.774
4615	0.366	0.743
4616	0.370	0.754
4617	0.409	0.770
4618	0.359	0.738
4619	0.390	0.750
4620	0.416	0.757
4621	0.358	0.743
4622	0.341	0.763
4623	0.398	0.749
4624	0.385	0.762
4625	0.399	0.761
4626	0.384	0.763

4627	0.419	0.772
4628	0.350	0.746
4629	0.372	0.763
4630	0.412	0.774
4631	0.398	0.769
4632	0.358	0.765
4633	0.406	0.771
4634	0.325	0.744
4635	0.382	0.761
4636	0.412	0.766
4637	0.359	0.766
4638	0.383	0.761
4639	0.358	0.762
4640	0.432	0.774
4641	0.373	0.770
4642	0.435	0.757
4643	0.366	0.749
4644	0.394	0.773
4645	0.397	0.746
4646	0.402	0.775
4647	0.333	0.756
4648	0.393	0.759
4649	0.350	0.758
4650	0.381	0.761
4651	0.412	0.760
4652	0.446	0.755
4653	0.383	0.785
4654	0.445	0.780
4655	0.394	0.759
4656	0.361	0.757
4657	0.413	0.769
4658	0.378	0.763
4659	0.398	0.759
4660	0.371	0.752
4661	0.375	0.761
4662	0.368	0.745
4663	0.403	0.778
4664	0.389	0.775
4665	0.378	0.729
4666	0.330	0.732
4667	0.383	0.751
4668	0.360	0.761

4669	0.378	0.757
4670	0.407	0.764
4671	0.387	0.767
4672	0.376	0.743
4673	0.419	0.772
4674	0.365	0.742
4675	0.300	0.752
4676	0.363	0.767
4677	0.402	0.772
4678	0.420	0.757
4679	0.377	0.748
4680	0.413	0.765
4681	0.387	0.771
4682	0.369	0.778
4683	0.354	0.769
4684	0.363	0.760
4685	0.373	0.762
4686	0.318	0.753
4687	0.383	0.769
4688	0.383	0.768
4689	0.382	0.757
4690	0.345	0.753
4691	0.393	0.752
4692	0.413	0.775
4693	0.406	0.761
4694	0.385	0.783
4695	0.353	0.757
4696	0.389	0.754
4697	0.416	0.771
4698	0.392	0.764
4699	0.399	0.768
4700	0.369	0.749
4701	0.383	0.763
4702	0.401	0.780
4703	0.452	0.790
4704	0.337	0.750
4705	0.373	0.758
4706	0.329	0.741
4707	0.446	0.778
4708	0.397	0.761
4709	0.310	0.740
4710	0.368	0.752

4711	0.373	0.758
4712	0.417	0.770
4713	0.359	0.755
4714	0.413	0.767
4715	0.411	0.762
4716	0.345	0.743
4717	0.336	0.738
4718	0.404	0.783
4719	0.427	0.766
4720	0.405	0.770
4721	0.405	0.752
4722	0.380	0.743
4723	0.397	0.760
4724	0.402	0.768
4725	0.393	0.753
4726	0.385	0.749
4727	0.352	0.746
4728	0.362	0.754
4729	0.387	0.758
4730	0.410	0.777
4731	0.411	0.760
4732	0.417	0.747
4733	0.387	0.751
4734	0.418	0.755
4735	0.403	0.768
4736	0.400	0.770
4737	0.362	0.742
4738	0.396	0.772
4739	0.380	0.764
4740	0.414	0.765
4741	0.408	0.769
4742	0.334	0.753
4743	0.375	0.783
4744	0.402	0.768
4745	0.401	0.762
4746	0.371	0.756
4747	0.335	0.744
4748	0.410	0.766
4749	0.416	0.762
4750	0.375	0.743
4751	0.332	0.732
4752	0.388	0.756

4753	0.407	0.778
4754	0.394	0.759
4755	0.402	0.759
4756	0.412	0.797
4757	0.317	0.737
4758	0.407	0.772
4759	0.384	0.765
4760	0.371	0.766
4761	0.458	0.775
4762	0.405	0.771
4763	0.420	0.773
4764	0.409	0.771
4765	0.403	0.754
4766	0.363	0.752
4767	0.354	0.735
4768	0.349	0.753
4769	0.388	0.747
4770	0.380	0.758
4771	0.447	0.772
4772	0.353	0.775
4773	0.408	0.765
4774	0.389	0.762
4775	0.410	0.776
4776	0.425	0.763
4777	0.427	0.786
4778	0.447	0.758
4779	0.357	0.742
4780	0.369	0.752
4781	0.387	0.780
4782	0.411	0.784
4783	0.385	0.765
4784	0.409	0.753
4785	0.392	0.758
4786	0.383	0.770
4787	0.367	0.745
4788	0.352	0.742
4789	0.418	0.759
4790	0.363	0.744
4791	0.394	0.744
4792	0.401	0.751
4793	0.359	0.746
4794	0.357	0.758

4795	0.342	0.736
4796	0.415	0.772
4797	0.408	0.766
4798	0.395	0.749
4799	0.430	0.768
4800	0.364	0.745
4801	0.431	0.762
4802	0.379	0.770
4803	0.395	0.760
4804	0.387	0.757
4805	0.367	0.761
4806	0.382	0.760
4807	0.386	0.749
4808	0.406	0.757
4809	0.403	0.759
4810	0.438	0.769
4811	0.375	0.758
4812	0.445	0.765
4813	0.395	0.754
4814	0.413	0.769
4815	0.373	0.756
4816	0.361	0.763
4817	0.395	0.764
4818	0.431	0.779
4819	0.372	0.744
4820	0.341	0.750
4821	0.395	0.776
4822	0.338	0.745
4823	0.366	0.746
4824	0.398	0.765
4825	0.413	0.767
4826	0.382	0.761
4827	0.330	0.755
4828	0.417	0.770
4829	0.406	0.734
4830	0.398	0.765
4831	0.385	0.764
4832	0.409	0.760
4833	0.383	0.754
4834	0.415	0.770
4835	0.409	0.780
4836	0.345	0.750

4837	0.382	0.769
4838	0.383	0.761
4839	0.372	0.741
4840	0.402	0.788
4841	0.395	0.764
4842	0.362	0.760
4843	0.411	0.769
4844	0.416	0.759
4845	0.341	0.746
4846	0.393	0.767
4847	0.395	0.755
4848	0.403	0.745
4849	0.372	0.737
4850	0.400	0.767
4851	0.358	0.749
4852	0.385	0.762
4853	0.395	0.751
4854	0.390	0.760
4855	0.370	0.753
4856	0.375	0.761
4857	0.416	0.762
4858	0.386	0.763
4859	0.383	0.747
4860	0.401	0.760
4861	0.374	0.775
4862	0.407	0.770
4863	0.403	0.749
4864	0.433	0.773
4865	0.459	0.782
4866	0.386	0.759
4867	0.440	0.776
4868	0.408	0.766
4869	0.382	0.778
4870	0.424	0.779
4871	0.379	0.762
4872	0.374	0.750
4873	0.411	0.770
4874	0.416	0.765
4875	0.389	0.765
4876	0.348	0.764
4877	0.370	0.764
4878	0.321	0.742

4879	0.345	0.733
4880	0.373	0.758
4881	0.358	0.751
4882	0.405	0.765
4883	0.410	0.753
4884	0.351	0.747
4885	0.361	0.740
4886	0.457	0.777
4887	0.392	0.762
4888	0.390	0.747
4889	0.341	0.738
4890	0.370	0.747
4891	0.337	0.755
4892	0.388	0.761
4893	0.366	0.743
4894	0.409	0.767
4895	0.383	0.765
4896	0.426	0.757
4897	0.440	0.761
4898	0.413	0.780
4899	0.430	0.776
4900	0.411	0.797
4901	0.342	0.744
4902	0.353	0.738
4903	0.389	0.763
4904	0.359	0.760
4905	0.384	0.754
4906	0.409	0.752
4907	0.393	0.753
4908	0.373	0.753
4909	0.406	0.771
4910	0.345	0.724
4911	0.334	0.733
4912	0.371	0.752
4913	0.386	0.772
4914	0.426	0.777
4915	0.378	0.760
4916	0.401	0.766
4917	0.398	0.781
4918	0.381	0.769
4919	0.337	0.734
4920	0.404	0.739

4921	0.415	0.777
4922	0.321	0.738
4923	0.383	0.770
4924	0.378	0.762
4925	0.341	0.746
4926	0.375	0.754
4927	0.341	0.762
4928	0.386	0.756
4929	0.420	0.752
4930	0.363	0.784
4931	0.387	0.760
4932	0.441	0.773
4933	0.389	0.770
4934	0.382	0.747
4935	0.375	0.785
4936	0.379	0.761
4937	0.358	0.753
4938	0.429	0.777
4939	0.440	0.768
4940	0.367	0.733
4941	0.399	0.771
4942	0.413	0.755
4943	0.378	0.765
4944	0.396	0.755
4945	0.387	0.747
4946	0.377	0.752
4947	0.412	0.771
4948	0.445	0.779
4949	0.368	0.750
4950	0.369	0.748
4951	0.364	0.762
4952	0.438	0.780
4953	0.381	0.762
4954	0.403	0.756
4955	0.425	0.755
4956	0.353	0.737
4957	0.412	0.764
4958	0.397	0.749
4959	0.384	0.753
4960	0.369	0.752
4961	0.403	0.768
4962	0.421	0.769

4963	0.449	0.774
4964	0.418	0.750
4965	0.351	0.744
4966	0.382	0.741
4967	0.408	0.774
4968	0.350	0.749
4969	0.428	0.774
4970	0.344	0.758
4971	0.383	0.767
4972	0.437	0.787
4973	0.390	0.761
4974	0.383	0.744
4975	0.296	0.741
4976	0.288	0.731
4977	0.367	0.749
4978	0.368	0.757
4979	0.349	0.735
4980	0.383	0.763
4981	0.393	0.776
4982	0.374	0.762
4983	0.403	0.775
4984	0.419	0.780
4985	0.386	0.755
4986	0.355	0.755
4987	0.370	0.742
4988	0.444	0.784
4989	0.367	0.771
4990	0.379	0.753
4991	0.418	0.777
4992	0.382	0.774
4993	0.325	0.763
4994	0.394	0.776
4995	0.386	0.750
4996	0.376	0.760
4997	0.425	0.769
4998	0.378	0.765
4999	0.330	0.742
5000	0.390	0.774
